# Adaptive venom evolution and toxicity in octopods is driven by extensive novel gene formation, expansion, and loss

**DOI:** 10.1093/gigascience/giaa120

**Published:** 2020-11-10

**Authors:** Brooke L Whitelaw, Ira R Cooke, Julian Finn, Rute R da Fonseca, Elena A Ritschard, M T P Gilbert, Oleg Simakov, Jan M Strugnell

**Affiliations:** Centre for Sustainable Tropical Fisheries and Aquaculture, James Cook University, 1 James Cook Dr, Douglas QLD 4811 , Australia; Sciences, Museum Victoria, 11 Nicholson St, Carlton, Victoria 3053, Australia; College of Public Health, Medical and Vet Sciences, James Cook University,1 James Cook Dr, Douglas QLD 4811 , Australia; La Trobe Institute of Molecular Science, La Trobe University, Plenty Rd &, Kingsbury Dr, Bundoora, Melbourne, Victoria 3086, Australia; Sciences, Museum Victoria, 11 Nicholson St, Carlton, Victoria 3053, Australia; Center for Macroecology, Evolution and Climate (CMEC), GLOBE Institute, University of Copenhagen, Universitetsparken 15, 2100 Copenhagen, Denmark; Department of Neurosciences and Developmental Biology, University of Vienna,Universitätsring 1, 1010 Wien, Vienna, Austria; Department of Biology and Evolution of Marine Organisms, Stazione Zoologica Anton Dohrn, Naples, Italy; Center for Evolutionary Hologenomics, GLOBE Institute, University of Copenhagen, Øster Voldgade 5–7, 1350 Copenhagen, Denmark; Department of Neurosciences and Developmental Biology, University of Vienna,Universitätsring 1, 1010 Wien, Vienna, Austria; Centre for Sustainable Tropical Fisheries and Aquaculture, James Cook University, 1 James Cook Dr, Douglas QLD 4811 , Australia; Department of Ecology, Environment and Evolution, La Trobe University, Plenty Rd &, Kingsbury Dr, Bundoora, Melbourne, Victoria 3086, Australia

**Keywords:** cephalopod genome, comparative genomics, gene family expansions, transposable elements, venom evolution

## Abstract

**Background:**

Cephalopods represent a rich system for investigating the genetic basis underlying organismal novelties. This diverse group of specialized predators has evolved many adaptations including proteinaceous venom. Of particular interest is the blue-ringed octopus genus (*Hapalochlaena*), which are the only octopods known to store large quantities of the potent neurotoxin, tetrodotoxin, within their tissues and venom gland.

**Findings:**

To reveal genomic correlates of organismal novelties, we conducted a comparative study of 3 octopod genomes, including the Southern blue-ringed octopus (*Hapalochlaena maculosa*). We present the genome of this species and reveal highly dynamic evolutionary patterns at both non-coding and coding organizational levels. Gene family expansions previously reported in *Octopus bimaculoides* (e.g., zinc finger and cadherins, both associated with neural functions), as well as formation of novel gene families, dominate the genomic landscape in all octopods. Examination of tissue-specific genes in the posterior salivary gland revealed that expression was dominated by serine proteases in non–tetrodotoxin-bearing octopods, while this family was a minor component in *H. maculosa*. Moreover, voltage-gated sodium channels in *H. maculosa* contain a resistance mutation found in pufferfish and garter snakes, which is exclusive to the genus. Analysis of the posterior salivary gland microbiome revealed a diverse array of bacterial species, including genera that can produce tetrodotoxin, suggestive of a possible production source.

**Conclusions:**

We present the first tetrodotoxin-bearing octopod genome *H. maculosa*, which displays lineage-specific adaptations to tetrodotoxin acquisition. This genome, along with other recently published cephalopod genomes, represents a valuable resource from which future work could advance our understanding of the evolution of genomic novelty in this family.

## Background

Reconstructing the evolution of novelties at the genomic level is becoming an increasingly viable approach to elucidate their origin. The recent publication of octopod genomes provides an opportunity to investigate the link between genomic and organismal evolution in this unique lineage for which genomic resources have been lacking [1]. From their emergence 275 million years ago (mya) [2], octopods have diversified into >300 species, inhabiting tropical to polar regions, from the deep sea to shallow intertidal zones [3]. As a highly diverse group, octopods show remarkable variation in body form and function. They are specialized soft-bodied predators that are well adapted to their environment with prehensile limbs lined with chemosensory suckers [4], the ability to manipulate skin texture and colour using specialized chromatophores [5], the largest invertebrate nervous systems (excluding those of other cephalopods) [6], and a relatively large circumesophageal brain allowing for complex problem solving and retention of information [7]. Furthermore, the cephalopods have independently evolved proteinaceous venom, which is produced and stored within a specialized gland known as the posterior salivary gland (PSG). All octopods are believed to possess a form of proteinaceous venom used to subdue prey [8–10]. Serine proteases are a common component of cephalopod venoms and have been observed in the PSG of squids, cuttlefish, and octopods [10–13]. Convergent recruitment of serine proteases has been observed between many vertebrate (Squamata [14–16] and Monotremata [17]) and invertebrate (Hymenoptera [18], Arachnida [19], Gastropoda [20], Remipedia [21], and Cnidarian [22]) venomous lineages.

In addition to these proteinaceous venoms, the blue-ringed octopus (genus *Hapalochlaena*) is the only group that also contains the potent non-proteinaceous neurotoxin tetrodotoxin (TTX) [12, 23]. The mechanism of TTX resistance, which allows for safe sequestration of TTX, has been attributed to several substitutions in the p-loop regions of voltage-gated sodium channels (Na_v_) in *Hapalochlaena lunulata* [24]. However, these channels have yet to be examined in *Hapalochlaena maculosa* and *Hapalochlaena fasciata*. TTX resistance has also been studied in a range of other genera including pufferfish [25], newts [26, 27], arachnids [28], snakes [29], and gastropods [30].

The blue-ringed octopus is easily identified by iridescent blue rings, which advertise its toxicity in an aposematic display [31–33]. Sequestration of the TTX within bodily tissues is unique to this genus among cephalopods [32, 34]. While other unrelated TTX-bearing species primarily use TTX for defense, *Hapalochlaena* is the only known taxon to utilize TTX in venom [23, 35]. The effect of TTX inclusion on venom composition and function has been previously investigated in the southern blue-ringed octopus (*H. maculosa*) [9]. Relative to the non–TTX-bearing species *Octopus kaurna, H. maculosa* exhibited greater expression of putative dispersal factors such as hyaluronidase, which serve to aid in the dispersal of toxic venom components [9]. Conversely, tachykinins—neurotoxins known from other octopods [36, 37]—were absent from the *H. maculosa* PSG [9]. Further investigation into the broader impact of TTX on the evolutionary trajectory of the species has yet to be addressed owing to the absence of a genome.

This study presents the genome of the southern blue-ringed octopus (*H. maculosa*, NCBI:txid61716; marinespecies.org: taxname:342334), the first from the genus *Hapalochlaena*. By using a comparative genomic approach we are able to examine the emergence of octopod novelties, at a molecular level between *H. maculosa* and the 2 non–TTX-bearing octopods: the California 2-spot octopus (*Octopus bimaculoides*) and the long-armed octopus (*Callistoctopus minor*). We also address unique features of venom evolution in octopods while also addressing the species-specific evolution of tetrodotoxin acquisition and resistance in *H. maculosa*.

## Data Description

### Genome assembly and annotation

The southern blue-ringed octopus genome was sequenced using Illumina paired-end and Dovetail sequencing from a single female collected at Beaumaris Sea Scout Boat Shed, Beaumaris, Port Phillip Bay, Victoria, Australia. The assembly was composed of 48,285 scaffolds with an N50 of 0.93 Mb and total size of 4.08 Gb. A total of 29,328 inferred protein-coding genes were predicted using a PASA [38] and an Augustus [39] pipeline and supplemented with zinc finger and cadherin genes obtained from aligning *H. maculosa* transcripts to *O. bimaculoides* gene models (Supplementary Note S1.1–S1.4). Completeness of the genome was estimated using BUSCO [40], which identified 87.7% complete and 7.5% fragmented genes against the metazoan database of 978 groups (Supplementary Note S3.2).

*H. maculosa* has a highly heterozygous genome (0.95%), similar to *Octopus vulgaris* (1.1%) [41] but far higher than *O. bimaculoides* (0.08%) [42]. While the low heterozygosity of *O. bimaculoides* is surprising, other molluscs also have highly heterozygous genomes in accordance with *H. maculosa*, including the gastropods (1–3.66%) [43, 44] and bivalves (0.51–3%) [45–51] (Supplementary Table S5).

### PSMC and mutation rate

The mutation rate for *H. maculosa* was estimated to be 2.4 × 10^−9^ per site per generation on the basis of analysis of synonymous differences with *O. bimaculoides* (Supplementary Note S1.5). The mutation rate is comparable to the average mammalian mutation rate of 2.2 × 10^−9^ per site per generation, and *Drosophila*, 2.8 × 10^−9^ [52, 53]. Owing to the unavailability of a suitable closely related and comprehensive genome until the publication of *O. bimaculoides* in 2015 [42], this is the first genome-wide mutation rate estimated for any cephalopod genome.

The historic effective population size (*N_e_*) of *H. maculosa* was estimated using the pairwise sequentially Markovian coalescent (PSMC) model (Supplementary Fig. S2). Population size was found to initially increase during the early Pleistocene, followed by a steady decline that slows slightly at ∼100 kya. It should be noted that PSMC estimates are not reliable at very recent times owing to a scarcity of genomic blocks that share a recent common ancestor in this highly heterozygous genome. A decline in population size started during the mid-Pleistocene ∼1 mya, a time of unstable environmental conditions with fluctuations in both temperature and glaciation events [54–56]. Corals in the genus *Acropora* show a similar pattern of expansion and contraction attributed to niche availability after the mass extinction of shallow-water marine organisms 2–3 mya, followed by the unstable mid-Pleistocene climate [57, 58]. A similar pattern of expansion and decline in effective population size has also been observed in the Antarctic icefish among other marine organisms distributed in the Southern Hemisphere [59].

### Phylogenomics

**Figure 1:**
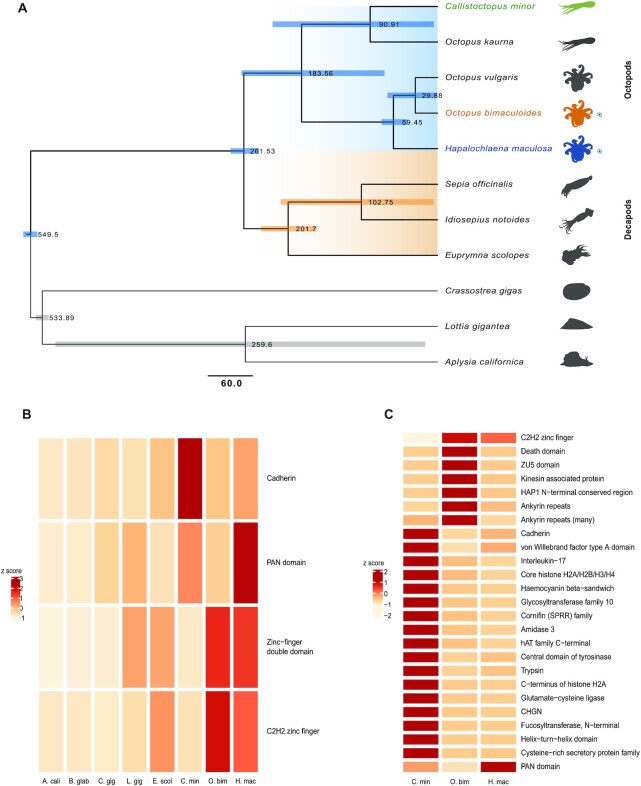
Comparisons of molluscan genomes and gene families. (**A)** Time-calibrated maximum likelihood phylogeny of 7 molluscan genomes (*Aplysia californica, Lottia gigantea, Crassostrea gigas, Euprymna scolopes, Octopus bimaculoides, Callistoctopus minor*,and*Hapalochlaena maculosa*) and 4 transcriptomes (*Octopus kaurna, Octopus vulgaris, Sepia officinalis*, and *Idiosepius notoides*) using 2,108 single-copy orthologous sequence clusters. Node labels show divergence times in millions of years (mya); blue (divergence to octopods) and orange bars (decapods) represent standard error within a 95% confidence interval. Octopodiformes lineages are highlighted in blue and decapod orange. Scale bar represents mya. (**B)** Expansions of octopod gene families relative to molluscan genomes *Aplysia californica* (A. cali), *Biomphalaria glabrata* (B. glab), *C. gigas* (C. gig), *L. gigantea* (L. gig), *E. scolopes* (E. scol), *C. minor* (C. min), *O. bimaculoides* (O. bim), and *H. maculosa* (H. mac). (**C)** Lineage-specific gene expansions in the octopod genomes *C. minor* (C. min), *O. bimaculoides* (O. bim), and *H. maculosa* (H. mac). CHGN: chondroitin N-acetylgalactosaminyltransferase; C2H2: Cys2-His2; SPRR: small proline-rich proteins.

A total of 2,108 (single copy/1-to-1) orthologous clusters were identified between the molluscan genomes and transcriptomes of 11 species and used to construct a time-calibrated maximum likelihood tree (Fig. 1a). The phylogenetic reconstruction estimated the divergence time between *H. maculosa* and its nearest relative, *O. bimaculoides*, to be ∼59 mya. *C. minor* diverged from this clade much earlier at ∼183 mya. Previous phylogenies using a combination of a small number of mitochondrial and nuclear genes [60–62] and orthologs derived from transcriptomes [63] support this topology. Likewise, estimates by Tanner et al. 2017, using a concatenated alignment of 197 genes with a Bayesian approach, placed divergence of *H. maculosa* from *Abdopus aculeatus* at ∼59 mya [2].

Inference of “shared” phenotypic traits can be difficult to resolve with the current literature. For example, false eye spots/ocelli observed in both *O. bimaculoides* and *H. maculosa* are structurally very different. Each ocellus in *H. maculosa* is composed of a continuous single blue ring [33], while *O. bimaculoides* has a blue ring composed of multiple small rings. Morphological variations of ocelli structure and colour, in conjunction with the taxonomically sporadic occurrence of this trait across species within *Octopus* and *Amphioctopus*, limit our interpretation as to the evolutionary history of this trait in octopods [3]. Large gaps remain in the literature between phenotypic traits in cephalopods and their genomic source [1]. This study aims to provide a genomic framework to enable resolution of these features by profiling changes in several genomic characters: (i) gene duplications, (ii) novel gene formation, and (iii) non-coding element evolution.

### Organismal impact of novel genes and gene family expansions

Gene family expansions between octopods (*O. bimaculoides, C. minor*, and *H. maculosa)* and 3 other molluscan genomes (*Aplysia californica, Lottia gigantea*, and *Crassostrea gigas*) were examined using Pfam annotations. A total of 5,565 Pfam domains were identified among 6 molluscan genomes. *H. maculosa* and *C. minor* exhibit expansions in the cadherin gene family, characteristic of other octopod genomes, including *O. bimaculoides* (Fig. 1B) [42, 64]*. C. minor*, in particular, shows the greatest expansion of this family within octopods. Expansions of protocadherins, a subset of the cadherin family, have also occurred independently in squid [42], with the octopod expansions occurring after divergence ∼135 mya [42]. The shared ancestry of octopod cadherins was also documented by Styfhals et al. [64] using phylogenetic inference between *O. bimaculoides* and *O. vulgaris*. Cadherins, specifically protocadherins, play crucial roles in synapse formation, elimination, and axon targeting within mammals and are essential mediators of short-range neuronal connections [65–68]. It should be noted that octopods lack a myelin sheath; as a result short-range connections are integral to maintaining signal fidelity over distance [6]. The independent expansions of protocadherins within chordate and cephalopod lineages are believed to be associated with increased neuronal complexity [42, 64]. Elevated expression of protocadherins within neural tissues has been observed in *O. vulgaris* and *O. bimaculoides* by Styfhals et al. [64] and Albertin et al. [42], respectively. In particular Styfhals et al. [64] noted differential expression across neural tissues including supra-esophageal mass, sub-esophageal mass, optic lobe, and the stellate ganglion [64]. However, functional implications of observed expression patterns remain speculative without further study.

*H. maculosa* also shows expansions in the C2H2-type zinc finger family. Zinc fingers form an ancient family of transcription factors, which among other roles serve to regulate transposon splicing, as well as embryonic and neural development [69, 70]. Expansion of this type of zinc finger in *O. bimaculoides* has been associated with neural tissues. It should be noted that due to the inherent difficulty in fully annotating the zinc finger family, alternative methods were used to examine the number of exons in *C. minor* with high similarity to annotated zinc finger genes in *O. bimaculoides* (Supplementary Note S5.1). A total of 609 exons (not captured by published gene models) from *C. minor* were found with high similarity to accepted zinc finger genes in *O. bimaculoides*, suggesting that this family is larger than that which the genome annotation infers.

**Figure 2:**
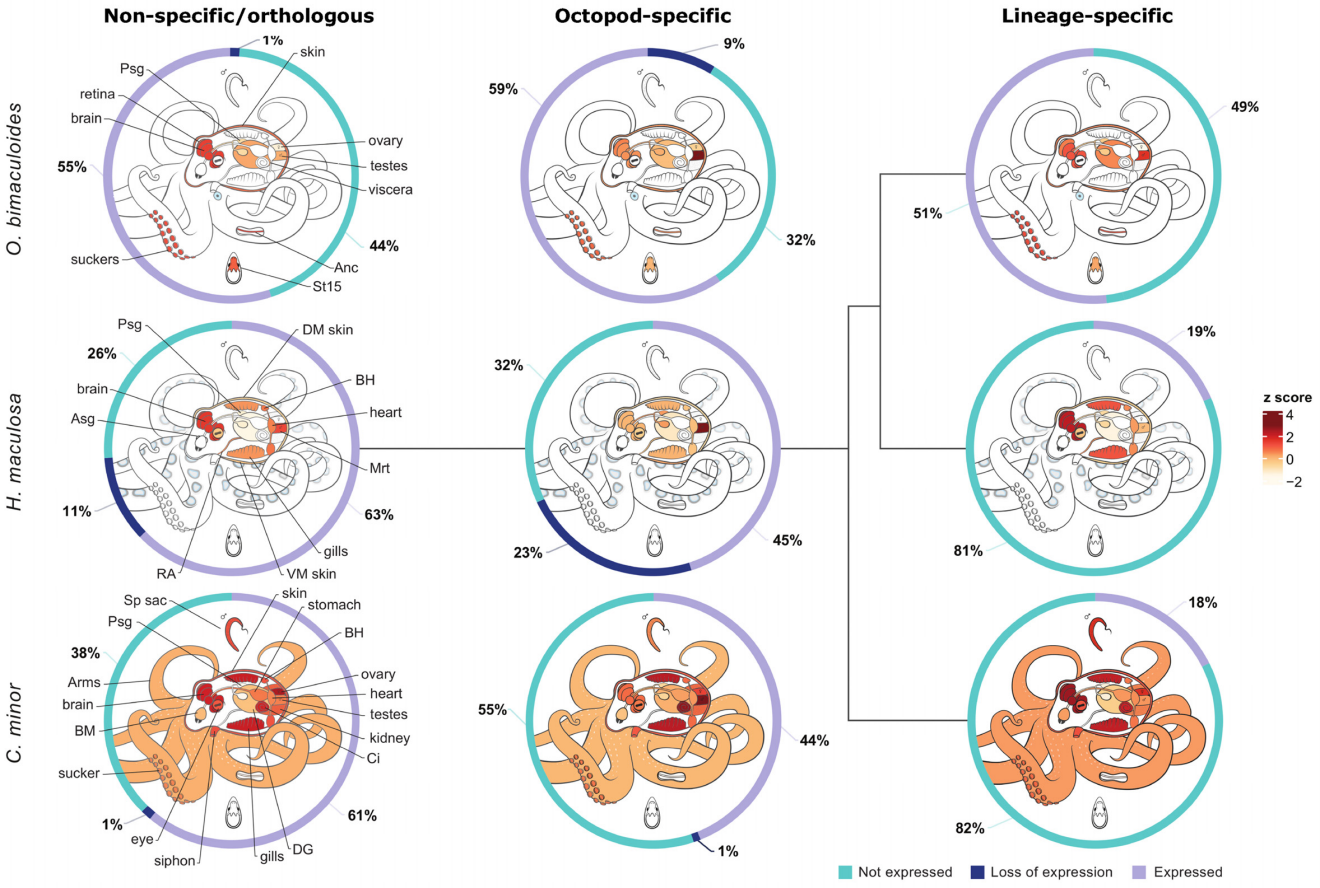
Dynamics of gene expression in octopod genomes. Proportion of gene expression across levels of specificity from not specific to octopods or an octopus species (left) to octopod-specific (middle) and lineage-specific (right). Donut plots show gene expression as some expression in any tissue (purple), no expression (blue), or expression that has been lost (dark blue). Loss of expression requires an ortholog of the gene to be expressed in ≥1 species and not expressed in the other species. Heatmaps at each specificity level show average expression of genes within their respective tissues, low expression (cream) to high expression (dark red).

Examination of genes specifically expressed within neural tissues found that cadherins were among the most highly expressed gene families of all octopod species. Particularly in *C. minor*, relative to the other octopods, such a trend reflects the gene family expansions found in this species (Fig. 2). Zinc fingers were less pronounced, representing 1.1% of overall expression in *C. minor* compared to cadherins at 11.3%. Overall, neural tissues express a large diversity of Pfams with each species, exhibiting a similar profile and proportion of orthologous to lineage-specific genes.

### Novel patterns of gene expression

High-level examination of gene dynamics (expression, loss of expression, and absence of expression) between octopods across different levels of orthology provides insight into large-scale expression patterns and highlights lineage-specific loss of expression.

The greatest proportion of genes in each species examined were not specific to octopods or an octopus lineage (ancient genes) (Fig. 2). Expression of these genes was enriched in neural tissues across all species, indicating the core conservation of neural development and function. However, we also find that genes specific to each octopod species also show this expression pattern. The overall elevated expression of genes within neural tissues could be reflective of the extensive neural network present in cephalopods, which comprises ∼520 million nerve cells [71], rivalling vertebrates/mammals in size [6]. Expression of many novel genes in the nervous system may also indicate contribution of those genes to lineage-specific neural network evolution. In contrast, genes that date back to the shared octopod ancestor show highest expression in male reproductive tissues in all species.

Loss of expression between octopod genomes is exhibited most clearly in *H. maculosa*, with 11% (1,993 genes) of all ancient genes having no expression, compared to 1% in both *O. bimaculoides* and *C. minor*. Absence of gene expression for genes whose orthologs have retained expression in ≥1 other species suggests a unique evolutionary trajectory from other octopods. It should be noted that differences in tissue sampling may in part influence these values and owing to the limited sampling of species, loss of expression cannot be inferred at a species level and may have occurred at any point in the lineage. To fully understand the implications of the gene family contractions and loss of expression in *H. maculosa*, relative to other octopods, further investigation is required.

### Evolution of the octopod non-coding genome

Similar to other cephalopod genomes, the *H. maculosa* genome has a high repeat content of 37.09% (bases masked). *O. bimaculoides* and *C. minor* are also highly repetitive, with 46% and 44% of their genomes composed of transposable elements (TE), respectively. Of the repetitive elements, LINEs dominate the decapodiform *Euprymna scolopes* genome, accounting for its larger genome size [72], while SINEs are expanded in all 4 octopod genomes. SINEs have been previously documented in *O. bimaculoides* (7.86%) [42], comparable with *H. maculosa* (7.53%), while fewer SINEs were previously reported for *C. minor* (4.7%) [73]. SINE elements also dominate the *O. vulgaris* genome, with an expansion occurring after divergence from *O. bimaculoides* [41]. Rolling circle elements are a prominent minor component in octopods, particularly in *H. maculosa*. Rolling circle transposons have been isolated from plant (*Zea mays*) and mammalian genomes. They depend greatly on proteins used in host DNA replication and are the only known class of eukaryotic mobile element (transposon) to have this dependence [74]. TE elements in cephalopod lineages show differing expansions between most of the genomes currently available, suggesting that they are highly active and play a strong role in cephalopod evolution.

Enrichment of transposable elements associated with genes (flanking regions 10 kb up- and downstream) was not observed compared to the whole genome for any species examined. More notable were differences between species; in particular *C. minor* shows a greater proportion of LINE to SINE elements relative to both *O. bimaculoides* and *H. maculosa*.

Together, this highlights a very dynamic evolutionary composition of repeats in cephalopods that requires further study to test for any potential association with changes in gene expression or genome evolution.

### Dynamics of gene expression in the PSG

The PSG is the primary venom-producing gland in octopods. Venom composition in the majority of octopods is primarily composed of proteinaceous toxins. *Hapalochlaena* is an exception, containing an additional non-proteinaceous neurotoxin, TTX, within their venom. We hypothesize that the *Hapalochlaena* PSG will exhibit a loss of redundant proteinaceous toxins due to the presence of TTX.

**Figure 3:**
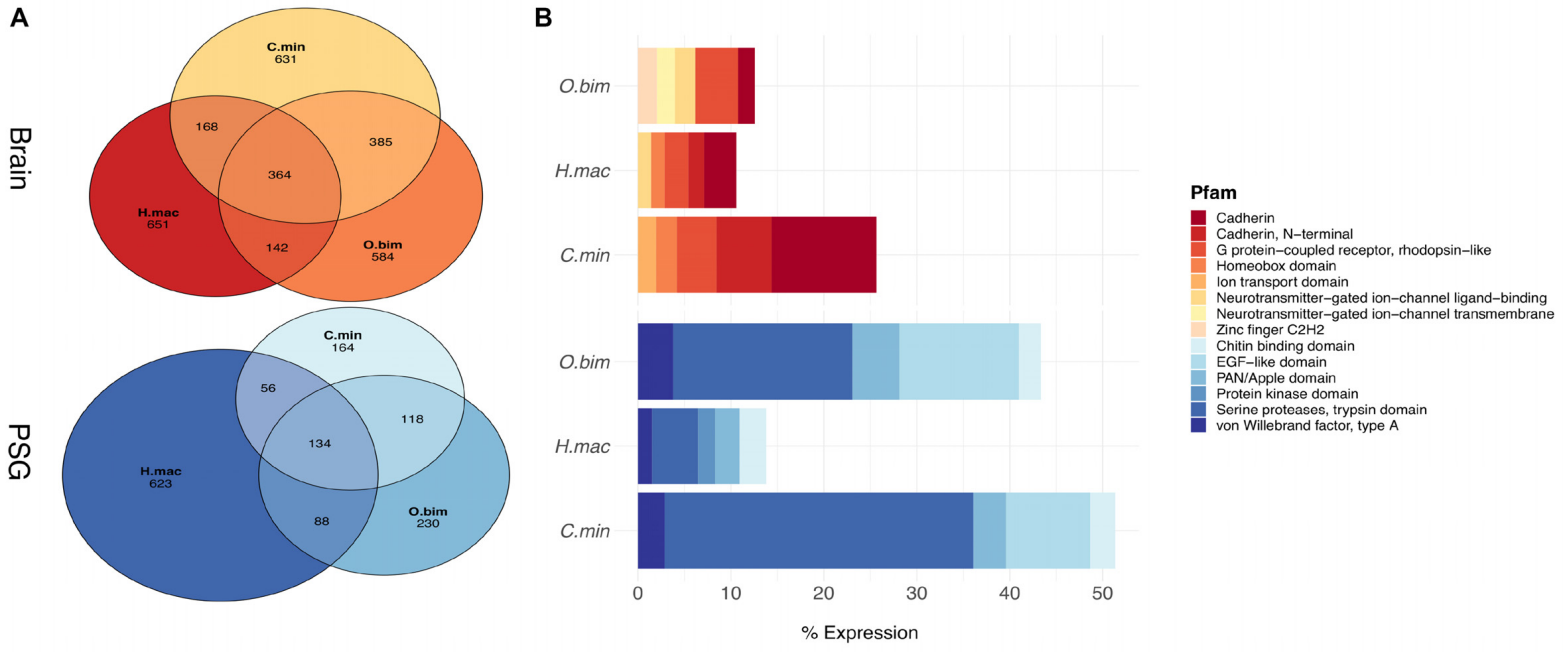
Dynamics of gene expression in neural and venom-producing tissues of octopods. Tissue-specific expression of genes within the brain (red) and posterior salivary gland (PSG) (blue) of *H. maculosa* (H.mac), *O. bimaculoides* (O.bim), and *C. minor* (C.min). A) Venn diagram shows numbers of shared and exclusive genes between species (left). B) Bar chart of the top 5 Pfams and their contribution to overall expression in the brain (right).

**Figure 4:**
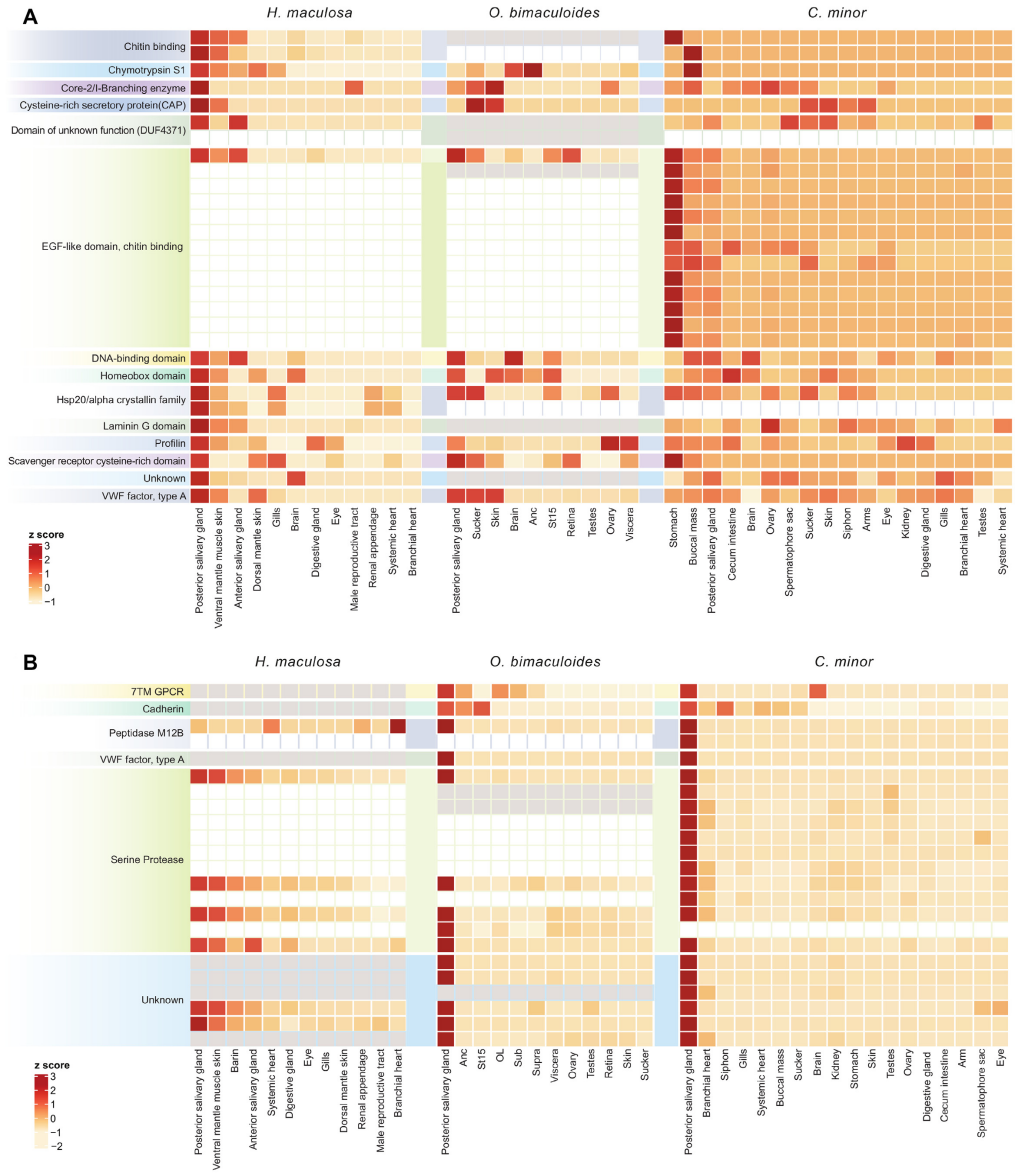
Examination of posterior salivary gland (PSG) gene expression between 3 octopod genomes. **(A)** Heat map of genes expressed specifically in the PSG of *H. maculosa* (τ > 0.8) and their orthologs in *O. bimaculoides* and *C. minor* lacking specific expression to the PSG (τ < 0.8). Genes with an ortholog lacking expression are coloured in grey while the absence of an ortholog is white. **(B)** Heat map of genes expressed specifically in the PSG of both *O. bimaculoides* and *C. minor* (τ > 0.8) and their orthologs in *H. maculosa* lacking specific expression to the PSG.

Examination of all PSG-specific genes from the 3 octopods revealed a disproportionate number of genes exclusive to *H. maculosa* (Fig. 3A). A total of 623 genes were exclusive to *H. maculosa* PSG compared with only 230 and 164 exclusive to *O. bimaculoides* and *C. minor* PSGs, respectively. Additionally, we predict that the *H. maculosa* PSG is functionally more diverse on the basis of the number of Pfam families detected, 532 in total. Comparatively, the PSG genes in *O. bimaculoides* and *C. minor* are fewer and more specialized. Gene family expansions of serine proteases dominate expression, comprising >30% of total PSG-specific expression in *C. minor* and 17–20% in *O. bimaculoides* (Fig. 3B). Serine proteases were also among genes whose expression seems to have shifted between octopod species. Several serine proteases show specific expression to the PSG of *O. bimaculoides* and *C. minor* while being expressed in a non-specific pattern among brain, skin, muscle, and anterior salivary gland tissues in *H. maculosa* (Fig. 4B). Most notable is the absence of many paralogs in both *H. maculosa* and *O. bimaculoides*, suggesting a lineage-specific expansion of this cluster in *C. minor*. Fewer serine protease genes can also be observed in *H. maculosa* (Fig. 4). Similarly, reprolysin (M12B) exhibits shifting expression in *H. maculosa*, presumably from the PSG to the branchial heart, and a complete loss of paralogs from the genome. While the function of this protein has not been assessed in octopus, members of this protein family exhibit anticoagulant properties in snake venom [75–78].

Serine proteases have been previously documented in cephalopod venom and are prime candidates for conserved toxins in octopods. Cephalopod-specific expansions have been identified with strong association to the PSG in 11 cephalopods (7 octopus, 2 squid, and 2 cuttlefish) [8, 13]. All serine proteases identified from the PSG of these species were found to belong to the cephalopod-specific clade. Functionally, cephalopod venom serine proteases have yet to be assessed. However, octopod venom has been observed to have strong digestive and hemolytic properties, which may be in part due to this crucial protein family [79–81]. The reduced number and expression of serine proteases in *H. maculosa* suggests a change in function of the PSG for this species. These results support the hypothesis of toxin redundancy in the *H. maculosa* PSG due to the incorporation of tetrodotoxin. Previous proteomic analysis of the *H. maculosa* PSG revealed high expression of hyaluronidase, which often serves as a dispersal factor within snake venom, facilitating the spread of toxin while not being directly toxic to their prey [9, 82]. While further investigation is required, the incorporation of TTX within *H. maculosa* venom may have contributed to a shift in function, with proteins present acting to support the spread of venom and digestion of tissues.

### TTX resistance of the Na_v_ channels

To identify the mechanism of TTX resistance in *H. maculosa*, the voltage-gated sodium channel (Na_v_) sequences were compared between susceptible (human) and resistant (pufferfish, salamanders, and garter snakes) species. TTX binds to the p-loop regions of sodium channels, inhibiting the flow of sodium ions in neurons, resulting in paralysis [83, 84]. Inhibition of TTX binding has been observed in species that either ingest TTX via prey, such as garter snakes [85], and in those that retain TTX within their tissues like pufferfish [86].

**Figure 5:**
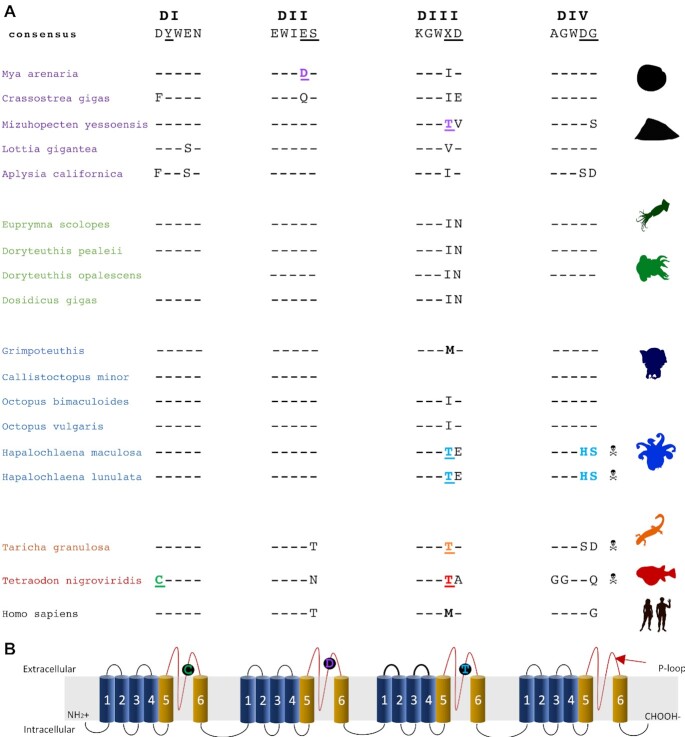
Mechanism of tetrodotoxin resistance within the posterior salivary gland of *H. maculosa* (PSG). (**A)** Alignment of voltage-gated sodium channel α-subunits (DI, DII, DIII, and DIV) p-loop regions. Mutations conferring resistance are coloured in green (pufferfish), orange (salamander), purple (clam), and blue (octopus). Susceptible mutations at the same site are black and boldface. Sites that may be involved with resistance are in boldface. (**B)** Schematic of voltage-gated sodium channel (Na_v_) α-subunits (DI, DII, DIII, and DIV). Each unit is composed of 6 subunits, 1–4 (blue) and 5–6 (yellow). Alternating extra- and intracellular loops are shown in black with the p-loops between subunits 5 and 6 highlighted in red. Mutations conferring resistance are shown within black circles on p-loops.

Two Na_v_ genes were identified in the *H. maculosa* genome (Na_v_1 and Na_v_2); this is congruent with the recent identification of 2 Na_v_ isoforms in *H. lunulata* [24] (Supplementary Figs S8 and S9). Among cephalopods with sequenced Na_v_1 channels, p-loop regions are highly conserved, with both DI and DII shared between all species. The regions DIII and DIV closer to the C-terminal end of the protein in *Hapalochlaena* sp. contain mutations, which may affect TTX binding and differ between families and species as follows. Similar to the pufferfish (*Arothron, Canthigaster, Takifugu*, and *Tetraodon*) [87] and garter snake *Thamnophis couchii* [88], *H. maculosa* Nav1 has a mutation within the third p-loop at site (DIII) from M1406T, while all other cephalopods have an Ile(I) at this position (Fig. 5A). The dumbo octopus (*Grimpoteuthis*) is the only exception, retaining the susceptible M at this site similar to humans and other non-resistant mammals [83]. Additionally, the fourth p-loop (DIV) in *H. maculosa* exhibits 2 substitutions at known TTX binding sites: D1669H and H1670S. In a previous study a Met to Thr substitution into a TTX-sensitive Nav1.4 channel decreased binding affinity to TTX by 15-fold [87]. Likewise, a 10-fold increase in sensitivity was observed from a T1674M substitution in a mite (*Varroa destructor*) channel VdNav1 [28]. However, resistance is often a result of multiple substitutions and when I1674T/D1967S occur together in VdNav1, resistance is multiplicative, resulting in “super-resistant” channels with binding inhibition of 1,000-fold. The combination of M1406T/D1669H in *H. maculosa* also occurs in the turbellarian flatworm *Bdelloura candida* (BcNav1) [87, 89]. While it has yet to be assessed for TTX resistance, the replacement of aspartic acid in *B. candida* with a neutral amino acid has been predicted to disrupt TTX binding by preventing formation of a salt bridge or hydrogen bond [89, 90]. These 3 substitutions (M1406T, D1669H, and H1670S) in *H. maculosa*, with the potential to inhibit TTX binding, have also been identified by Geffeney et al. [24] in *H. lunulata*. It has yet to be established whether these mutations are derived from a shared ancestor or have occurred independently.

**Figure 6:**
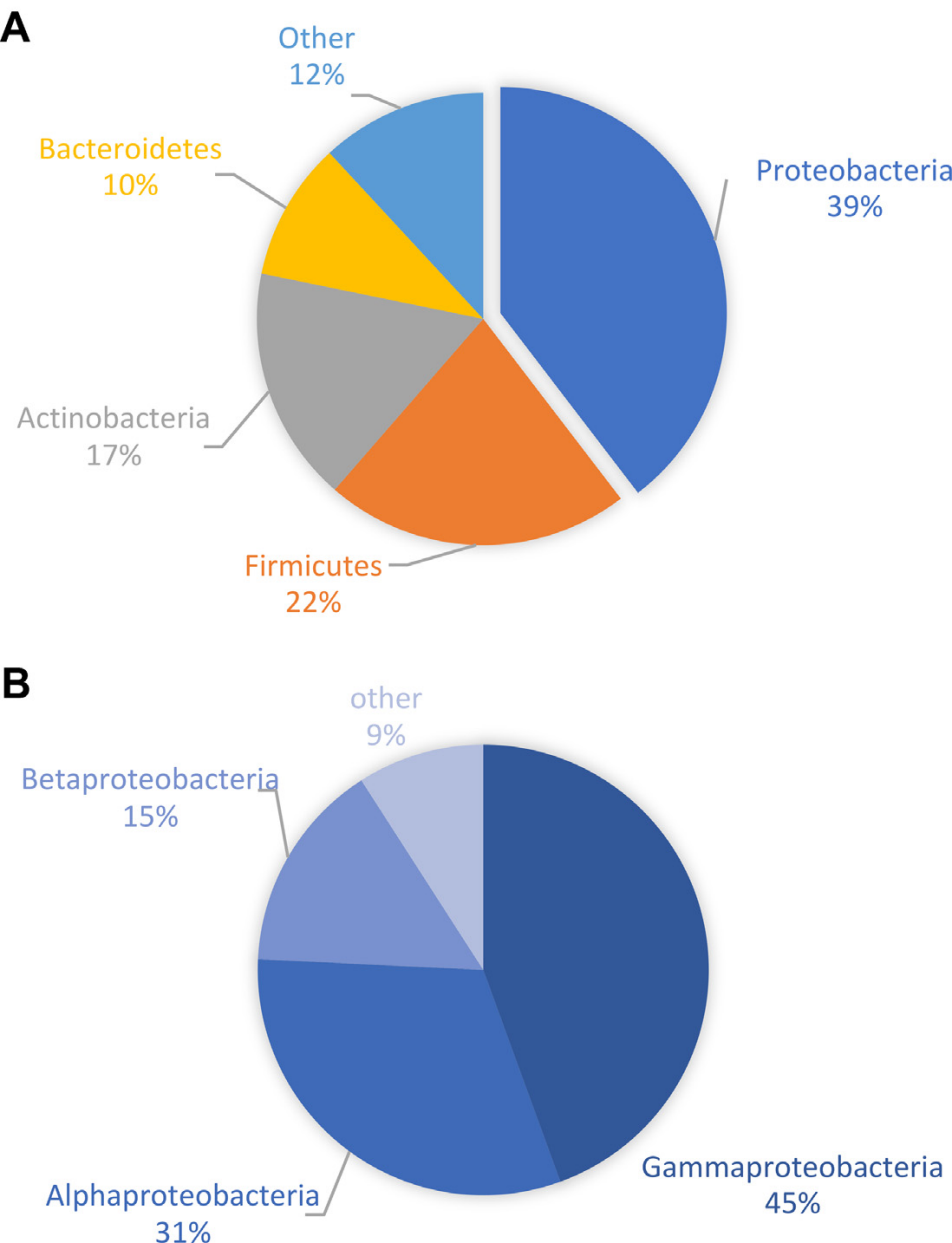
Assessment of bacteria within the posterior salivary gland of *H. maculosa* (PSG). (**A)** Bacterial composition at the phylum level of an*H. maculosa* posterior salivary/venom gland. (**B)** Composition of the largest phylum, Protobacteria, of an *H. maculosa* posterior salivary/venom gland.

While *Hapalochlaena* remains the best-documented example of TTX resistance among cephalopods, other species may contain some level of TTX resistance (e.g., *O. vulgaris*) [91, 92]. Saxitoxin (STX) is a similar toxin in structure and function, and mutations resistant to TTX are often also STX inhibiting [93]. *O. vulgaris* has been observed consuming STX-contaminated bivalves with no negative effects and as such is believed to be resistant [92]. However, no mutations known to reduce TTX/STX binding affinity occur in its Nav1 [92, 94]. The selective pressure facilitating the evolution of STX/TTX resistance in these shallow-water benthic octopods may be toxic prey, similar to garter snakes. STX is also known as a paralytic shellfish poison. Produced by photosynthetic dinoflagellates and bioaccumulated in bivalves [95], this toxin contaminates a common octopus food source. Pelagic squids such as the Humboldt (*Doryteuthis gigas*) and longfin inshore squid (*Doryteuthis pealeii*) do not appear to be TTX/STX resistant; mass strandings of Humboldt squid have been associated with ingestion of STX-contaminated fish [96]. Likewise, no evidence of resistance was found in the sodium channel of the dumbo octopus (*Grimpoteuthis*). This species typically inhabits depths of 2,000–5,000 m and is unlikely to encounter STX-contaminated food sources [97].

### Microbiome of the PSG

TTX is produced through a wide variety of bacteria, which are common in marine sediments and have been isolated from organisms such as pufferfish [25, 98, 99]. Sequestration of TTX is not exclusive to the blue-ringed octopus among molluscs. Gastropods such as *Pleurobranchaea maculata* [100] and *Niotha clathrata* [30], as well as some bivalves, are also capable of sequestering TTX [95]. The commonly held hypothesis for TTX acquisition within *Hapalochlaena* is that it is bacterial in origin and is either ingested or endosymbiotic [100, 101]. Analysis of a ribo-depleted RNA sample from the PSG of *H. maculosa* revealed a highly diverse composition of bacterial genera with Simpson and Shannon diversity indices of 4.77 and 0.94, respectively. The dominant phyla were Proteobacteria and Firmicutes, composing, respectively, 41% and 22% of overall bacterial species detected (Fig. 6). To date, 151 strains of TTX-producing bacteria have been identified from 31 genera. Of these, 104 are members of Proteobacteria [102]. The genera *Pseudomonas* and *Bacillus* belonging to the phyla Proteobacteria and Firmicutes, respectively, have been previously identified in the PSG of *Hapalochlaena* sp. (*Octopus maculosus*) [101]. Examination of these bacterial strains revealed TTX production, and extracts injected into mice proved to be lethal [101]. A more recent study on the bacterial composition of *H. maculosa* PSG did not identify TTX-producing strains [100]. However, only a small subset of the many strains identified were tested. Congruent with our findings the diversity of bacterial genera was high and this may complicate identification of species responsible for TTX production. The biosynthetic pathway of TTX has yet to be elucidated, and as a result, only culturable bacterial species can be tested for TTX production.

## Conclusions

This work describes the genome of a unique TTX-bearing mollusc, the southern blue-ringed octopus (*H. maculosa*). Much of cephalopod evolution is barely understood owing to sparseness of genomic data. Our analysis provides the first glimpse into genomic changes underlying genome evolution of closely related octopod species. While the size, heterozygosity, and repetitiveness of the blue ring genome is congruent with previously published octopod genomes, we find similar yet independent expansions of key neuronal gene families across all 3 species and show evidence for the involvement of gene novelty in the evolution of key neuronal, reproductive, and sensory tissues. The evolution of venom in octopods also differs between species, with *H. maculos*a showing a reduction in the number and expression of serine proteases in their venom gland relative to the other octopods in this study. Inclusion of TTX in *H. maculosa* distinguishes this species from related octopods and is believed to affect toxin recruitment and retention because the highly potent TTX is sufficient to subdue common octopod prey without additional toxins.

## Methods

### Genome sequencing and assembly

DNA was extracted from a single *H. maculosa* female collected at Port Phillip Bay, Victoria, Australia. Two types of Illumina libraries were constructed, standard paired end and Illumina mate pairs (Supplementary Data S2). Dovetail sequencing, Chicago libraries improved upon original sequencing,c resulting in an overall coverage of 71×. Assembly-stats [103] was used to ascertain the quality of the assembly and relevant metrics (Supplementary Note S1).

### Transcriptome sequencing

The *H. maculosa* transcriptome was generated using 12 tissues (brain, anterior salivary gland, digestive gland, renal, brachial heart, male reproductive tract, systemic heart, eyeballs, gills, posterior salivary gland, dorsal mantle, and ventral mantle tissue). RNA was extracted using the Qiagen RNeasy kit. Construction of complementary DNA libraries was outsourced to AGRF (Australian Genome Research Facility), Melbourne, and conducted using their TruSeq mRNA Library Prep with polyA selection and unique dual indexing method. Libraries were constructed using 3 μg of RNA at a concentration of >100 ng/μL. Each tissue was sequenced on 1/12th of an Illumina HiSeq2000 lane with 1 lane used in total.

### De novo transcriptome assembly

*De novo* assembly of the *H. maculosa* transcriptome was conducted using sequencing data from 11 tissues (as listed above) and Trinity v10.11.201 (Trinity, RRID:SCR_013048) [104]. Default parameters were used aside from *k*-mer coverage, which was set to 3 to account for the large data volume. Protein-coding sequences were identified using Trinotate (Trinotate, RRID:SCR_018930) [105] and domains assigned by Interpro v72.0 (InterPro, RRID:SCR_006695) [106].

### Genome annotation

Genes were annotated using a *de novo* predictor supplemented with transcriptomic evidence. Training models were produced by PASA (PASA, RRID:SCR_014656) [38] using a transcriptome composed of 12 tissues (as listed above) and supplied to the *de novo* predictor Augustus (Augustus, RRID:SCR_008417) [39] along with intron, exon, and repeat hints (generated by repeatmasker). Alternative splicing of gene models was also predicted using PASA (PASA, RRID:SCR_014656). Methods used for annotation have been documented in an online git repository [107]. Additional genes were predicted by mapping raw expressed reads against the genome. Functional annotation of gene models was achieved using InterPro v72.0 (InterPro, RRID:SCR_006695) [106]. Completeness of genes was assessed using BUSCO v3 Metazoan database (BUSCO, RRID:SCR_015008) [40].

### Heterozygosity

JELLYFISH v2.2.1 (Jellyfish, RRID:SCR_005491) was used in conjunction with GenomeScope (GenomeScope, RRID:SCR_017014) [108] to calculate heterozygosity in *H. maculosa* using a *k*-mer frequency of 21 (Supplementary Table S5).

### Repetitive and transposable elements

Repetitive and transposable elements were annotated using RepeatModeler v1.0.9 (RepeatScout) (RepeatModeler, RRID:SCR_015027) and masking performed with RepeatMasker v4.0.8 (RepeatMasker, RRID:SCR_012954) [109] (Supplementary Note S3.3). Analysis of gene-associated TEs was conducted by extracting TEs within flanking regions 10 kb upstream and downstream of genes using Bedtools v2.27.1 (BEDTools, RRID:SCR_006646) [110].

### Calibration of sequence divergence with respect to time

Divergence times between the molluscan genomes (*C. gigas, L. gigantea, A. californica, E. scolopes, O. bimaculoides, C. minor*, and *H. maculosa*) and transcriptomes (*Sepia officinalis, Idiosepius notoides, O. kaurna*, and *O. vulgaris*) was obtained using a mutual best hit approach. Bioprojects for each genome used are as follows: *C. gigas* (PRJNA629593 and PRJEB3535), *L. gigantea* (PRJNA259762 and PRJNA175706), *A. californica* (PRJNA629593 and PRJNA13635), and *E. scolopes* (PRJNA47095). *O. bimaculoides* was obtained from [111]. The *I. notoides*(BioProject: PRJNA302677) transcriptome was sequenced and assembled using the same method previously described for the *H. maculosa* transcriptome. Whole genomes and transcriptomes were BLASTed against *O. bimaculoides*. The resulting hits were filtered, and alignments shared between all species extracted. A maximum likelihood phylogeny was generated using RAxML v8.0 (RAxML, RRID:SCR_006086) [112]. Phylobayes v3.3 (PhyloBayes, RRID:SCR_006402) [113] was used to calculate divergence times (Supplementary NoteS4.1).

### Effective population size (PSMC)

Historical changes in effective population size were estimated using PSMC implemented in the software MSMC [114, 115]. To generate inputs for MSMC we selected a subset of the reads used for genome assembly corresponding to 38× coverage of reads from libraries with short (500 bp) insert sizes. These were pre-processed according to GATK best practices; briefly, adapters were marked with Picard 2.2.1, reads were mapped to the *H. maculosa* genome using bwa mem v 0.7.17 (BWA, RRID:SCR_010910) [116], and PCR duplicates identified using Picard v2.2.1. To avoid inaccuracies due to poor coverage or ambiguous read mapping we masked regions where short reads would be unable to find unique matches using SNPable [117] and where coverage was more than double or less than half the genome-wide average of 38×. Variant sites were called within unmasked regions and results converted to MSMC input format using msmc-tools [118]. All data for *H. maculosa* scaffolds of length >1 Mb were then used to generate 100 bootstrap replicates by dividing data into 500-kb chunks and assembling them into 20 chromosomes with 100 chunks each. We then ran msmc2 on each bootstrap replicate and assembled and imported the resulting data into R for plotting. A mutation rate of 2.4e−9 per base per year and a generation time of 1 year were assumed in order to set a timescale in years and convert coalescence rates to effective population size.

### Mutation rate

Mutation rate was calculated by extracting orthologous genes from *O. bimaculoides* and *H. maculosa*. Neutrality was assumed for genes with very low expression (<10 TPM across all tissues). Neutral genes were aligned using MAFFT v7.407 [119] and codeml (PAML, RRID:SCR_014932) [120] was used to calculate substitution metrics (dS). Per base neutral substitution between lineages was determined using the mean dS value divided by divergence time (refer to "Calibration of sequence divergence with respect to time") over the number of generations. Because octopus are diploid the rate was divided by 2. Divergence between species was calculated using Phylobayes v3.3 (PhyloBayes, RRID:SCR_006402) [113].

### Quantifying gene expression/specificity

Gene expression (as TPM) within individual tissues was calculated using Kallisto (kallisto, RRID:SCR_016582) [121] for the transcriptomic data sets of *H. maculosa, O. bimaculoides*, and *C. minor*. Defaults were used and counts of specific genes were calculated as TPM defined as any gene with τ > 0.80.

### Gene model expression dynamics

Patterns of gene expression and loss were assessed across octopod genomes at differing taxonomic/organismal levels. Gene models were classified as lineage-specific, octopod specific, or non-specific (orthologous to a gene outside of octopods). Expression at each level was determined using whole transcriptomes from all tissues of each species. Genes with expression within ≥1 tissue were determined to be expressed; loss of expression was classified as a gene with a single ortholog in each species, which is expressed in ≥1 species and not expressed in the remaining species.

### Dynamics of PSG gene expression

To identify patterns of PSG-specific gene expression (losses and shifts) between the 3 available octopod genomes, genes with expression specific to the PSG of each species were examined separately. Specific gene expression was defined as τ > 0.8. Orthologous groups were identified between species using Orthovenn2 [122] and sequences that were identified as lineage specific were confirmed using BLAST. Types of expressions were categorized as follows: a loss of expression requires a gene to be present in all 3 octopods and expressed in ≥1 species while having no detectable expression in ≥1 species. A shift in expression occurs when an ortholog present in all species is expressed in different tissues.

### The role of the Na_v_ in TTX resistance

Sodium channels for the 3 octopus genomes along with all available in-house cephalopod transcriptomes were extracted manually using a series of BLAST searches against the nr database. Annotation was achieved using Interpro v72.0 (InterPro, RRID:SCR_006695) [106] and identification and extraction of p-loop regions of the sodium channel α-subunit were manually performed. Where sodium channels were incomplete, alignment against related complete channels was used to extract the p-loop regions. Individual mutations with potential to confer resistance were identified manually in Geneious v10.1 [123].

### Microbiome of PSG

A single ribo-depleted RNA sample of *H. maculosa* PSG was examined using the SAMSA2 pipeline [124] to identify the bacterial composition and corresponding molecular functions. Two databases were used, Subsys and NCBI RefBac. The Krona package [125] was used to produce visualizations of each dataset.

## Availability of Source Code and requirements

Project name: BRO_annotation

Project home page: https://github.com/blwhitelaw/BRO_annotation

Operating system(s): Linux

Programming language: Unix/Bash

Other requirements: high-performance computing

License: GPL-2.0 License

Any restrictions to use by non-academics: none

RRID:SCR_019072

## Availability of Supporting Data and Materials

Genomic and transcriptomic data produced and used in this article have been made available in the NCBI BioProject: PRJNA602771 under the following accession numbers: raw transcriptome (SAMN13930963–SAMN13930975), genome assembly (SAMN13906985), raw genome reads (SAMN13906958), and gene models (SAMN13942395). Voucher specimen for the transcriptome is stored at Melbourne Museum. All supporting data and materials are available in the *GigaScience* GigaDB database [126]. This includes expression data for the transcriptome, raw transcriptome reads, gene models, gene annotation gff and assembled genome, as well as files used in figure generation (i.e., trees, heat maps).

## Additional Files

Supplementary Note S1.GENOME SEQUENCING, ASSEMBLY AND ANALYSES

Supplementary Note S2. TRANSCRIPTOME SEQUENCING AND ANALYSIS

Supplementary Note S3. ANNOTATION OF TRANSPOSABLE ELEMENTS AND PROTEIN CODING GENES

Supplementary Note S4. MULTI-GENE PHYLOGENY AND GENE FAMILY EXPANSION ANALYSES

Supplementary Note S5. ANALYSIS OF NEURAL ASSOCIATED GENE FAMILIES

Supplementary Note S6. EVOLUTION OF THE VENOM/POSTERIOR SALIVARY GLAND IN OCTOPODS

Supplementary Note S7. EVOLUTION OF TETRODOTOXIN RESISTANCE IN H. MACULOSA

Supplementary Note S8. MICROBIOME OF THE H. MACULOSA POSTERIOR SALIVARY GLAND

Supplementary Table S1.Summary for Illumina libraries

Supplementary Table S2. Comparison of original Illumina and Dovetail augmented assemblies.

Supplementary Table S3. Statistical comparisons between original Illumina and Dovetail augmented assemblies

Supplementary Table S4. Assembly statistics for the three octopod genomes used in this study

Supplementary Table S5. GenomeScope version 1.0 H. maculosa results

Supplementary Table S6. Heterozygosity for published molluscan genomes

Supplementary Table S7. H. maculosa assembly assessed for completeness against the BUSCO Metazoan database.

Supplementary Table S8. Summary for H. maculosa repeat annotation

Supplementary Figure S1. Comparison of assembly continuity between original Illumina (input scaffolds) and Dovetail (Final scaffolds) augmented assemblies

Supplementary Figure S2. PSMC estimation of effective population size in H. maculosa

Supplementary Figure S3. QI-TREE Maximum-likelihood tree

Supplementary Figure S4. Phylogenetic tree of cadherins in H. maculosa (blue), O. bimaculoides (orange) and C. minor (green)

Supplementary Figure S5. Phylogenetic tree of protocadherins in H. maculosa (blue), O. bimaculoides (orange) and C. minor (green)

Supplementary Figure S6. Distribution of tau values for genes in H. maculosa, C. minor and O. bimaculoides

Supplementary Figure S7. Orthologous genes specifically expressed in the PSG of O. bimaculoides and C. minor which have no ortholog in H. maculosa

Supplementary Figure S8. Alignment of Na_v_1 p-loop regions

Supplementary Figure S9. Alignment of Nav2 p-loop regions

Supplementary Figure S10. Expression of Nav1 and Nav2 channels across shared tissues of H. maculosa (Hmac), O. bimaculoides (Obim) and C. minor (Cmin)

Supplementary Data 1 : Table of genomic Illumina library insert sizes

## Abbreviations

BLAST: Basic Local Alignment Search Tool; bp: base pairs; BUSCO: Benchmarking Universal Single-Copy Orthologs; BWA: Burrows-Wheeler Aligner; CHGN: chondroitin N-acetylgalactosaminyltransferase, C2H2: Cys2-His2; GATK: Genome Analysis Toolkit; Gb: gigabase pairs; kb: kilobase pairs; LINE: long interspersed nuclear element; MAFFT: Multiple Alignment using Fast Fourier Transform; Mb: megabase pairs; MSMC: multiple sequentially Markovian coalescent; mya: million years ago; NCBI: National Center for Biotechnology Information: PASA: Program to Assemble Spliced Alignments; PSG: posterior salivary gland; PSMC: pairwise sequentially Markovian coalescent; RAxML: Randomized Axelerated Maximum Likelihood; SINE: short interspersed nuclear element; SPRR: small proline-rich proteins; STX: saxitoxin; TE: transposable element; TPM: transcripts per million; TTX: tetrodotoxin.

## Ethics Declaration

Animal Ethics Approval: field collection of fishes, cephalopods (nautiluses, squids, cuttlefishes, and octopuses), and decapod crustaceans (crabs, lobsters, crayfishes, and their allies) was conducted for Museums Victoria (Animal Ethics Committee: Museums Victoria; AEC Approval No. 10006).

## Competing Interests

The authors declare that they have no competing interests.

## Funding

This work was supported by an Australian Biological Resources Study (ABRS) grant (ref: RF211–41). O.S. was supported by the Austrian Science Fund (FWF) grant P30686-B29.

## Supplementary Material

giaa120_GIGA-D-20-00135_Original_Submission

giaa120_GIGA-D-20-00135_Revision_1

giaa120_GIGA-D-20-00135_Revision_2

giaa120_Response_to_Reviewer_Comments_Original_Submission

giaa120_Response_to_Reviewer_Comments_Revision_1

giaa120_Reviewer_1_Report_Original_Submission Frank Anderson -- 6/3/2020 Reviewed

giaa120_Reviewer_1_Report_Revision_1 Frank Anderson -- 8/21/2020 Reviewed

giaa120_Reviewer_2_Report_Original_Submission Shana Geffeney -- 6/8/2020 Reviewed

giaa120_Reviewer_2_Report_Revision_1 Shana Geffeney -- 8/25/2020 Reviewed

giaa120_Reviewer_3_Report_Original_Submission Bryan Fry -- 6/10/2020 Reviewed

giaa120_Supplemental_Files
