# Supplementary material for: Adaptive venom evolution and toxicity in octopods is driven by extensive novel gene formation, expansion, and loss
Source: Gigascience. 2020 Nov 10;9(11):giaa120. doi: 10.1093/gigascience/giaa120 (PMC7656900; doi:10.1093/gigascience/giaa120)
Supplement: giaa120_GIGA-D-20-00135_Original_Submission [file giaa120_giga-d-20-00135_original_submission.pdf]

GigaScience

Adaptive venom evolution and toxicity in octopods is driven by extensive novel gene formation, expansion and loss

--Manuscript Draft--

**Manuscript Number:**	GIGA-D-20-00135
**Full Title:**	Adaptive venom evolution and toxicity in octopods is driven by extensive novel gene formation, expansion and loss
**Article Type:**	Research
**Funding Information:**	Australian Biological Resources Study (ref:RF211-41) — Assoc Prof Jan M. Strugnell
**Abstract:**	Background<br><br>Cephalopods represent a rich system for investigating the genetic basis underlying organismal novelties. This diverse group of specilised predators have evolved many unique adaptations including proteinaceous venom. Of particular interest is the blue-ringed-octopus genus (Hapalochlaena), which are the only octopods known to store large quantities of the potent neurotoxin, tetrodotoxin, within their tissues and venom gland.<br><br>Findings<br><br>To reveal genomic correlates of organismal novelties, we conducted a comparative study of three octopod genomes, including the Southern blue-ringed octopus (Hapalochlaena maculosa). We present the genome of this species and reveal highly dynamic evolutionary patterns at both non-coding and coding organizational levels. Gene family expansions previously reported in Octopus bimaculoides (e.g., zinc finger and cadherins, both associated with neural functions), as well as formation of novel gene families, dominate the genomic landscape in all octopods. Examination of tissue-specific genes in the posterior salivary gland (PSG) revealed that expression was dominated by serine proteases in non- tetrodotoxin bearing octopods, while this family was a minor component in H. maculosa . Moreover, voltage-gated sodium channels in H. maculosa contain a resistance mutation found in pufferfish and garter snakes, which is absent in other octopods. Analysis of the PSG microbiome revealed a diverse array of bacterial species, including genera that can produce tetrodotoxin, suggestive of a possible production source.<br><br>Conclusions<br><br>We present the first tetrodotoxin-bearing octopod genome H. maculosa, which displays species-specific adaptations to tetrodotoxin acquisition. This genome along with other recently published cephalopod genomes represent a valuable resource from which future work could advance the evolution of genomic novelty in this family.
**Corresponding Author:**	Brooke Lauren Whitelaw, Bachelor with Honours and current PhD candidate<br>James Cook University College of Science and Engineering<br>Townsville, QLD AUSTRALIA
**Corresponding Author Secondary Information:**	
**Corresponding Author's Institution:**	James Cook University College of Science and Engineering
**Corresponding Author's Secondary Institution:**	
**First Author:**	Brooke Lauren Whitelaw
**First Author Secondary Information:**	
**Order of Authors:**	Brooke Lauren Whitelaw
	Ira R. Cooke

Powered by Editorial Manager® and ProduXion Manager® from Aries Systems Corporation

	Julian Finn
	Rute R. da Fonseca
	Elena A. Ritschard
	M. T P. Gilbert
	Oleg Simakov
	Jan M. Strugnell
**Order of Authors Secondary Information:**	
**Additional Information:**	
**Question**	**Response**
Are you submitting this manuscript to a special series or article collection?	No
**Experimental design and statistics**<br><br>Full details of the experimental design and statistical methods used should be given in the Methods section, as detailed in our Minimum Standards Reporting Checklist. Information essential to interpreting the data presented should be made available in the figure legends.<br><br>Have you included all the information requested in your manuscript?	Yes
**Resources**<br><br>A description of all resources used, including antibodies, cell lines, animals and software tools, with enough information to allow them to be uniquely identified, should be included in the Methods section. Authors are strongly encouraged to cite Research Resource Identifiers (RRIDs) for antibodies, model organisms and tools, where possible.<br><br>Have you included the information requested as detailed in our Minimum Standards Reporting Checklist?	Yes
**Availability of data and materials**<br><br>All datasets and code on which the	Yes

conclusions of the paper rely must be either included in your submission or deposited in publicly available repositories (where available and ethically appropriate), referencing such data using a unique identifier in the references and in the "Availability of Data and Materials" section of your manuscript.<br><br>Have you have met the above requirement as detailed in our Minimum Standards Reporting Checklist?	

Manuscript
Click here to download Manuscript Manuscript_Bwhitelaw_GS.docx

Adaptive venom evolution and toxicity in octopods is driven by extensive novel gene formation, expansion and loss

Brooke L. Whitelaw[1,2], Ira R. Cooke[3,4], Julian Finn[2], Rute R. da Fonseca[5], Elena A. Ritschard[6], M. T P. Gilbert[7] Oleg Simakov[6], Jan M. Strugnell[1,8]

[1]Centre for Sustainable Tropical Fisheries and Aquaculture, James Cook University, Townsville, Queensland, 4811, Australia

[2]Sciences, Museum Victoria, Carlton, Victoria 3053, Australia

[3]College of Public Health, Medical and Vet Sciences, James Cook University, Townsville, Queensland, 4811, Australia

[4]La Trobe Institute of Molecular Science, La Trobe University, Melbourne, Victoria 3086, Australia

[5]Center for Macroecology, Evolution and Climate (CMEC), GLOBE Institute, University of Copenhagen, Universitetsparken 15, 2100 Copenhagen, Denmark;

[6]Department of Molecular Evolution and Development, University of Vienna, Universitätsring 1, 1010 Wien, Austria

[7]Center for Evolutionary Hologenomics, GLOBE Institute, University of Copenhagen, Øster Voldgade 5-7, 1350 Copenhagen, Denmark;

[8]Department of Ecology, Environment and Evolution, La Trobe University, Melbourne, Victoria, 3086 Australia

Abstract

Background

Cephalopods represent a rich system for investigating the genetic basis underlying organismal novelties. This diverse group of specilised predators have evolved many unique adaptations including proteinaceous venom. Of particular interest is the blue-ringed-octopus genus (*Hapalochlaena*), which are the only octopods known to store large quantities of the potent neurotoxin, tetrodotoxin, within their tissues and venom gland.

Findings

To reveal genomic correlates of organismal novelties, we conducted a comparative study of three octopod genomes, including the Southern blue-ringed octopus (*Hapalochlaena maculosa*). We present the genome of this species and reveal highly

dynamic evolutionary patterns at both non-coding and coding organizational levels. Gene family expansions previously reported in *Octopus bimaculoides* (e.g., zinc finger and cadherins, both associated with neural functions), as well as formation of novel gene families, dominate the genomic landscape in all octopods. Examination of tissue-specific genes in the posterior salivary gland (PSG) revealed that expression was dominated by serine proteases in non- tetrodotoxin bearing octopods, while this family was a minor component in *H. maculosa*. Moreover, voltage-gated sodium channels in *H. maculosa* contain a resistance mutation found in pufferfish and garter snakes, which is absent in other octopods. Analysis of the PSG microbiome revealed a diverse array of bacterial species, including genera that can produce tetrodotoxin, suggestive of a possible production source.

Conclusions

We present the first tetrodotoxin-bearing octopod genome *H. maculosa,* which displays species-specific adaptations to tetrodotoxin acquisition. This genome along with other recently published cephalopod genomes represent a valuable resource from which future work could advance the evolution of genomic novelty in this family.

Background

Reconstructing the evolution of novelties at the genomic level is becoming an increasingly viable approach to understand their origin. The recent publication of octopod genomes provides an opportunity to investigate the link between genomic and organismal evolution in this unique and underrepresented lineage[1]. From their emergence 275 mya[2], octopods have diversified into > 300 species, inhabiting tropical to polar regions, from the deep sea to shallow intertidal zones (FAO,)[3]. As a highly diverse group, octopods show remarkable variation in body form and function. They are specialised soft bodied predators that are well-adapted to their environment with prehensile limbs lined with chemosensory suckers[4], the ability to manipulate skin texture and colour using specialised chromatophores[5], the largest invertebrate nervous systems (excluding those of other cephalopods)[6], and a relatively large circumesophageal brain allowing for complex problem solving and retention of information[7]. Furthermore, proteinaceous venom is produced and stored within a specialised gland in cephalopods known as the posterior salivary gland (PSG). All octopods are believed to possess a form of proteinaceous venom used to subdue prey[8-

[10]. In addition to these proteinaceous venoms, the blue-ringed octopus (genus *Hapalochlaena*) is the only group that also contains the potent non-proteinaceous neurotoxin, tetrodotoxin (TTX)[11,12]. How resistance to TTX has been acquired at the genetic level, remains a large unknown, with TTX resistance studied in only in a few select species (i.e. pufferfish[13], newts[14,15] and gastropods[16]).

The blue-ringed octopus is easily identified by iridescent blue rings, which advertise its toxicity in an aposematic display [17-19]. Sequestration of the TTX within bodily tissues is unique to this genus among cephalopods[18,20]. Primarily used for defense in other unrelated TTX-bearing species, *Hapalochlaena* is the only known taxa to utilise TTX in venom[11,21]. The impact of TTX inclusion on venom composition and function has been previously investigated in the southern blue-ringed octopus (*H. maculosa*)[22]. Relative to the non-TTX bearing species *Octopus kaurna, H. maculosa* exhibited greater expression of putative dispersal factors such as hyaluronidase, which serve to aid in the dispersal of toxic venom components[22]. Conversely, tachykinins-neurotoxins known from other octopods [23,24] were absent from the *H. maculosa* PSG [22]. Further investigation into the broader impact of TTX on the evolutionary trajectory of the species has yet to be addressed due to the absence of a genome.

This study presents the genome of the southern blue-ringed octopus (*H. maculosa*), the first from the genus *Hapalochlaena.* By using a comparative genomic approach we are able to examine the emergence of octopod novelties, for example at the evolution of venoms, at a molecular level between *H. maculosa* and the two non-TTX bearing octopods: the California two-spot octopus (*O. bimaculoides*) and the long-armed octopus (*Callistoctopus minor*), while also addressing the species-specific evolution of tetrodotoxin acquisition and resistance in *H. maculosa.*

Keywords: cephalopod genome, comparative genomics, gene family expansions, transposable elements, venom evolution

Data Description

Genome assembly and annotation

The southern blue-ringed octopus genome was sequenced using Illumina paired end and Dovetail sequencing from a single female collected at Beaumaris Sea Scout

Boat Shed, Beaumaris, Port Phillip bay, Victoria, Australia. The assembly was composed of 48,285 scaffolds with an N50 of 0.93 Mb and total size of 4.08 GB. A total of 29,328 inferred protein coding genes were predicted using a PASA[25] and an Augustus[26] pipeline and supplemented with zinc finger and cadherin genes obtained from aligning *H. maculosa* transcripts to *O. bimaculoides* gene models(Supplementary notes 1.1-1.4). Completeness of the genome was estimated using BUSCO[27], which identified 87.7% complete and 7.5% fragmented genes against the metazoan database of 978 groups (Supplementary notes 3.2).

H. maculosa has a highly heterozygous genome (0.95%), similar to *O. vulgaris* (1.1%)[28] but far higher than *O. bimaculoides* (0.08%) [29]. While the low heterozygosity of *O. bimaculoides* is surprising, other molluscs also have highly heterozygous genomes in accordance with *H. maculosa*, including the gastropods (1-3.66%)[30,31] and bivalves (0.51-3%)[32-37] (Supplementary table 5).

PSMC (Pairwise Sequentially Markovian Coalescent) and mutation rate

The mutation rate for *H. maculosa* was estimated to be 2.4 x 10^{-9} per site per generation based on analysis of synonymous differences with *O. bimaculoides*

(Supplementary note 1.5). The mutation rate is comparable to the average mammalian mutation rate of 2.2 x 10^{-9} per site per generation, and *Drosophila*, 2.8 x 10^{-9} [38,39]. Due to the unavailability of a suitable closely related and comprehensive genome until the publication of *O. bimaculoides* in 2015[29], this is the first genome-wide mutation rate estimated for any cephalopod genome.

The historic effective population size (Ne) of *H. maculosa* was estimated using the pairwise sequentially Markovian coalescent (PSMC) model (Supplementary Fig 2). Population size was found to initially increase during the early Pleistocene, followed by a steady decline which slows slightly around 100kya. Note that PSMC estimates are not reliable at very recent times due to a scarcity of genomic blocks that share a recent common ancestor in this highly heterozygous genome. A decline in population size started during the mid-Pleistocene approximately 1mya, a time of unstable environmental conditions with fluctuations in both temperature and glaciation events[40-42]. Corals in the genus *Acropora* show a similar pattern of expansion and contraction attributed to niche availability post mass extinction of shallow water marine organisms 2-3 mya, followed by the unstable mid-Pleistocene climate[43,44]. A similar pattern of expansion and decline in effective population size has also been observed in the

Antarctic ice fish among other marine organisms distributed in the southern hemisphere[45].

Phylogenomics

A total of 2,108 (single copy/ 1-to-1) orthologous clusters were identified between the molluscan genomes and transcriptomes of 11 species and used to construct a time-calibrated maximum likelihood tree (Fig 1a). The phylogenetic reconstruction estimated the divergence time between *H. maculosa* and its nearest relative, *O. bimaculoides*, to be ~59 mya. *C. minor* diverged from this clade much earlier ~183 mya. Previous phylogenies using a combination of a small number of mitochondrial and nuclear genes[46-48] and orthologs derived from transcriptomes[49] support this topology. Likewise, divergence of the *H. maculosa from Abdopus aculeatus* has been previously estimated to be ~59 mya[2].

Inference of "shared" phenotypic traits can be difficult to resolve with the current literature. For example, false eye spots/ocelli observed in both *O. bimaculoides* and *H. maculosa* are structurally very different. Each ocellus in *H. maculosa* is composed of a continuous single blue ring[11], while *O. bimaculoides* has a blue ring composed of

multiple small rings. Morphological variations of ocelli structure and colour, along with their sporadic occurrence within *Octopus* and *Amphioctopus*[3], limits our interpretation as to the evolutionary history of this trait in octopods. Large gaps remain in the literature between phenotypic traits in cephalopods and their genomic source[1]. This study aims to provide a genomic framework to enable resolution of these features by profiling changes in several genomic characters: (i) gene duplications, (ii) novel gene formation, and (iii) non-coding element evolution.

Fig 1. Comparisons of molluscan genomes and gene families a) Time-calibrated maximum likelihood phylogeny of seven molluscan genomes (*Aplysia californica, Lottia gigantea, Crassostrea gigas, Euprymna scolopes, Octopus bimaculoides, Callistoctopus minor and Hapalochlaena maculosa*) and four transcriptomes (*Octopus kaurna, Octopus vulgaris, Sepia officinalis* and *Idiosepius notoides*) using 2,108 single copy orthologous sequence clusters. Node labels show divergence times in millions of years (mya), blue (divergence to octopods) and orange bars (decopods) represent standard error within a 95% confidence interval. Octopodiformes lineages are highlighted in blue and decapod orange. Scale bar represents millions of year (mya). **b)** Expansions of octopod gene families relative to molluscan genomes *Aplysia californica* (A. cali), *Biomphalaria glabrata* (B. glab), *Crassostrea gigas* (C. gig), *Lottia gigantea* (L. gig), *Euprymna scolopes* (E. scol) **c)** Species specific gene expansions in the octopod genomes *Callistoctopus*

minor (C. min), *Octopus bimaculoides* (O. bim) and *Hapalochlaena maculosa* (H. mac). Domains abbreviated: Chondroitin N-acetylgalactosaminyltransferase (CHGN), C2H2(Cys2-His2) zinc finger and Cornifin SPRR(small proline-rich proteins).

Organismal impact of novel genes and gene family expansions

Gene family expansions between octopods (*O. bimaculoides*, *C. minor* and *H. maculosa)* and three other molluscan genomes (*Aplysia californica*, *Lottia gigantea* and *Crassostrea gigas)* were examined using Pfam annotations. A total of 5565 Pfam domains were identified among six molluscan genomes. *H. maculosa* and *C. minor* exhibit expansions in the cadherin gene family, characteristic of other octopod genomes, including *O. bimaculoides* (Fig1b). *C. minor,* in particular, shows the greatest expansion of this family within octopods. Expansions of protocadherins, a subset of the cadherin family, have also occurred independently in squid [20], with the octopod expansions occuring post divergence ~135 mya [20]. Cadherins, specifically protocadherins, are essential mediators of short-range neuronal connections in

mammals[42] [43]. Due to the absence of a myelin sheath in octopods, short-range connections are integral to maintaining signal fidelity over distance[44].

H. maculosa also shows expansions in the C2H2-type zinc finger family. Zinc fingers form an ancient family of transcription factors, which among other roles serve to regulate transposon splicing, embryonic and neural development [45,46]. Expansion of this type of zinc fingers in *O. bimaculoides* has been associated with neural tissues. It should be noted that due to the inherent difficulty in fully annotating the zinc finger family, alternative methods were used to examine the number of exons in *C. minor* with high similarity to annotated zinc finger genes in *O. bimaculoides* (Supplementary notes 5.1). A total of 609 exons (not captured by published gene models) from *C. minor* were found with high similarity to accepted zinc finger genes in *O. bimaculoides,* suggesting this family is larger than that which the genome annotation infers.

Examination of genes specifically expressed within neural tissues found that cadherins were among the most highly expressed gene families of all octopod species. Particularly in *C. minor*, relative to the other octopods, such a trend reflects the gene family expansions found in this species (Fig2c). Zinc fingers were less pronounced, representing 1.1% of overall expression in *C. minor* compared to cadherins at 11.3%.

Overall, neural tissues express a large diversity of Pfams with each species, exhibiting a similar profile and proportion of orthologous to species-specific genes.

Novel patterns of gene expression

High level examination of gene dynamics (expression, loss of expression and absence of expression) between octopods across different levels of orthology provides insight into large scale expression patterns and highlights lineage specific loss of expression.

The greatest proportion of genes in each species examined were not specific to octopods or an octopus species (ancient genes) (Fig 2a). Expression of these genes were enriched in neural tissues across all species, indicating the core conservation of neural development and function. However, we also find that, genes specific to each octopod species also show this expression pattern. The overall elevated expression of genes within neural tissues could be reflective of the extensive neural network present in cephalopods, which comprises around 520 million nerve cells[47], rivalling vertebrates/mammals in size[44]. Expression of many novel genes in the nervous system

may also indicate contribution of those genes to species-specific neural network evolution. In contrast, genes that date back to the shared octopod ancestor show highest expression in male reproductive tissues in all species.

Loss of expression between octopod genomes is exhibited most clearly in *H. maculosa* with 11% (1993 genes) of all ancient genes having no expression, compared to 1% in both *O. bimaculoides* and *C. minor*. Absence of gene expression for genes whose orthologs have retained expression in one or more other species suggests a unique evolutionary trajectory from other octopods. It should be noted that differences in tissue sampling may in part influence these values. In order to understand the implications of gene expression loss, in tandem with overall reduction in genes number relative to the octopods, further investigation is required.

Fig2. Dynamics of gene expression in octopod genomes. Proportion of gene expression across levels of specificity from not specific to octopods or an octopus species (left) to octopod specific (middle) and species specific (right). Donut plots show gene expression as some expression in any tissue (purple), no expression (blue) or expression that has been lost (dark blue). Loss of expression requires an ortholog of

the gene to be expressed in one or more species and not expressed in the other species. Heatmaps at each specificity level show average expression of genes within their respective tissues, low expression (cream) to high expression (dark red).

Fig3. Dynamics of gene expression in neural and venom producing tissues of octopods. a) Tissue specific expression of genes within the brain of *H. maculosa, O. bimaculoides* and *C. minor* (red). Venn diagram shows numbers of shared and exclusive genes between species (Left). Bar chart of the top 5 Pfams and their contribution to overall expression in the brain (right). b) Tissue specific expression of genes within the posterior salivary gland (PSG) of *H. maculosa, O. bimaculoides* and *C. minor* (Blue). Venn diagram shows numbers of shared and exclusive genes between species (left). Bar chart of the top 5 Pfams and their contribution to overall expression in the PSG (right).

Evolution of the octopod non-coding genome

Similar to other cephalopod genomes, the *H. maculosa* genome has a high repeat content of 37.09% (bases masked). *O bimaculoides* and *C. minor* are also highly repetitive with 46.39% and 44% of their genomes composed of transposable elements (TE) respectively. Of the repetitive elements, LINEs dominate the decapodiform

Euprymna scolopes genome accounting for its larger genome size [48], while SINEs are expanded in all four octopod genomes. SINEs have been previously documented in *O. bimaculoides* (7.86%)[20], comparable with *H. maculosa* (7.53%), while fewer SINEs were previously reported for *C. minor* (4.7%)[49]. SINE elements also dominate the *O. vulgaris* genome with an expansion occurring post divergence from *O. bimaculoides*[19]. Rolling circle (RC) elements are a prominent minor component in octopods, particularly in *H. maculosa*. RC transposons have been isolated from plant (*Zea mays*) and mammalian genomes. They depend greatly on proteins used in host DNA replication and are the only known class of eukaryotic mobile element (transposon) to have this dependence[50]. TE elements in cephalopod lineages show differing expansions between most of the genomes currently available, suggesting they are highly active and play a strong role in cephalopod evolution.

Enrichment of transposable elements associated with genes (flanking regions 10kb up- and downstream) was not observed compared to the whole genome for any species examined. More notable, were differences between species, in particular *C. minor* shows a greater proportion of LINE to SINE elements relative to both *O. bimaculoides* and *H. maculosa*.

Together, this highlights a very dynamic evolutionary composition of repeats in cephalopods, that requires further study to test for any potential association with changes in gene expression or genome evolution.

Dynamics of gene expression in the posterior salivary gland (PSG)

The posterior salivary gland is the primary venom producing gland in octopods. Venom composition in the majority of octopods is primarily composed of proteinaceous toxins. *Hapalochlaena* is an exception containing an additional non-proteinaceous neurotoxin, TTX, within their venom. It has been hypothesized that the Hapalochlaena PSG will exhibit a loss of redundant proteinaceous toxins due to the presence of TTX.

Examination of all PSG-specific genes from the three octopods revealed a disproportionate number of genes exclusive to *H. maculosa* (Fig 3a). A total of 623 genes were exclusive to *H. maculosa* PSG compared to only 230 and 164 exclusive to *O. bimaculoides* and *C. minor*, respectively. Additionally, *H. maculosa* PSG is predicted to be functionally more diverse based on the number of Pfam families detected, 532 in total. Comparatively, the PSG genes in *O. bimaculoides* and *C. minor* are fewer and more

specialised. Gene family expansions of serine proteases dominate expression comprising over 30% of total PSG-specific expression in *C. minor* and 17-20% in *O. bimaculoides* (Fig 3b). Serine proteases were also among genes whose expression appears to have shifted between octopod species. Several serine proteases show specific expression to the PSG of *O. bimaculoides* and *C. minor* while being expressed in a non-specific pattern among brain, skin, muscle and anterior salivary gland tissues in *H. maculosa* (Fig 4b). Most notable is the absence of many paralogs in both *H. maculosa* and *O. bimaculoides* suggesting a species-specific expansion of this cluster in *C. minor*. Loss of serine protease genes can also be observed in *H. maculosa* (Fig 4c). Similarly, reprolysin (M12B) exhibits shifting expression in *H. maculosa*, presumably from the PSG to the branchial heart, and a complete loss of orthologs from the genome. While the function of this protein has not been assessed in octopus, members of this protein family exhibit anticoagulant properties in snake venom[50].

Serine proteases have been previously documented in cephalopod venom and are prime candidates for conserved toxins in octopods. Cephalopod-specific expansions have been identified with strong association to the PSG in 11 cephalopods (seven octopus, two squid and two cuttlefish)[8,51]. All serine proteases identified from the PSG

of these species were found to belong to the cephalopod specific clade. Functionally, cephalopod venom serine proteases have yet to be assessed. However, octopod venom has been observed to have strong digestive and hemolytic properties, which may be in part due to this crucial protein family[52-54]. The reduced number and expression of serine proteases in *H. maculosa* suggests a change in function of the PSG for this species. These results support the hypothesis of toxin redundancy in the *H. maculosa* PSG due to the incorporation of tetrodotoxin. Previous proteomic analysis of the *H. maculosa* PSG revealed high expression of hyaluronidase, which often serve as dispersal factors within snake venom, facilitating the spread of toxin while not being directly toxic to their prey[22,55]. While further investigation is required, the incorporation of TTX within *H. maculosa* venom may have contributed to a shift in function, with proteins present acting to support the spread of venom and digestion of tissues.

Fig 4. Examination of posterior salivary gland (PSG) gene expression between three octopod genomes. a) Heatmap of genes expressed specifically in the PSG of *H. maculosa* (tau $>$ 0.8) and their orthologs in *O. bimaculoides* and *C. minor* lacking specific expression to the PSG (tau $<$ 0.8). Genes with an ortholog lacking expression are coloured in grey while the absence of an ortholog is white. **b)**

Heatmap of genes expressed specifically in the (PSG) of both *O. bimaculoides* and *C. minor* (tau >0.8) and their orthologs in *H. maculosa* lacking specific expression to the PSG.

TTX resistance of the Na_v channels

To identify the mechanism of TTX resistance in *H. maculosa,* the voltage gated sodium channel (Na_v) sequences were compared between susceptible (human) and resistant (pufferfish, salamanders and garter snakes) species. TTX binds to the p-loop regions of sodium channels, inhibiting the flow of sodium ions in neurons, resulting in paralysis[56,57]. Inhibition of TTX binding has been observed in species which either ingest TTX via prey, such as garter snakes[58], and in those which retain TTX within their tissues like pufferfish[59].

Two Nav genes were identified in the *H. maculosa* genome (Na_v1 and Na_v2), this is congruent with the recent identification of two Na_v isoforms in *H. lunulata*[60](Supplementary Fig 8 & 9). Among cephalopods with sequenced Na_v1 channels, p-loop regions are highly conserved with both DI and DII shared between all species. The latter regions in DIII and DIV in *Hapalochlaena* sp. contain mutations,

which may impact TTX binding and differ between families and species as follows. Similar to the pufferfish (*Arothron, Canthigaster, Takifugu* and *Tetraodon*)[61] and garter snake *Thamnophis couchii* [62], *H. maculosa* Nav1 has a mutation within the third p-loop at site (DIII) from M1406T, while all other cephalopods have an Ile(I) at this position (Fig 5a). The dumbo octopus (*Grimpoteuthis*) is the only exception retaining the susceptible M at this site similar to humans and other non-resistant mammals[56]. Additionally, the fourth p-loop (DIV) in *H. maculosa* exhibits two substitutions at known TTX binding sites: D1669H and H1670S. In previous studies, when examined individually, the Met-Thr substitution in a TTX sensitive Nav1.4 rat channel decreased binding affinity in pufferfish by 15-fold[61]. Likewise, a 10-fold increase in sensitivity was observed from a T1674M substitution in a mite (*Varroa destructor*) channel VdNav1[63]. However, resistance is often a result of multiple substitutions and when I1674T/D1967S occur together in VdNav1, resistance is multiplicative resulting in “super resistant” channels with binding inhibition of 1000-fold. The combination of M1406T/ D1669H in *H. maculosa* also occurs in the turbellarian flatworm *Bdelloura candida*(BcNav1)[61,64]. While it has yet to be assessed for TTX resistance, the replacement of Asp in *B. candida* with a neutral amino acid has been predicted to disrupt TTX binding by preventing formation

of a hydrogen bond[65]. These three substitutions (M1406T, D1669H and H1670S) in *H. maculosa,* with the potential to inhibit TTX binding, have also been identified by Geffeney et al (2019) in *H. lunulata*[60]. It has yet to be established if these mutations are derived from a shared ancestor or have occurred independently.

While *Hapalochlaena* remain the best documented example of TTX resistance among cephalopods, other species may contain some level of TTX resistance (e.g. *Octopus vulgaris*)[66,67]. Saxitoxin (STX) is a similar toxin in structure and function, and mutations resistant to TTX are often also STX inhibiting[68]. *O. vulgaris* has been observed consuming STX contaminated bivalves with no negative impacts and as such is believed to be resistant[67]. However, no mutations known to reduce TTX/STX binding affinity occur in its Nav1[67,69]. The selective pressure facilitating the evolution of STX/TTX resistance in these shallow water benthic octopods may be toxic prey, similar to garter snakes. STX is also known as a paralytic shellfish poison (PSP). Produced by photosynthetic dinoflagellates and bioaccumulated in bivalves[70], this toxin contaminates a common octopus food source. Pelagic squids such as the humboldt (*D. gigas*) and longfin inshore squid (*D. pealeii*) do not appear to be TTX/STX resistant; mass strandings of humboldt squid have been associated with ingestion of STX

contaminated fish[71]. Likewise, no evidence of resistance was found in the sodium channel of the dumbo octopus (*Grimpoteuthis*). This species typically inhabits depths of 2000-5000m and is unlikely to encounter STX contaminated food sources[72].

Fig 5. Mechanism of tetrodotoxin resistance within the posterior salivary gland of *H. maculosa* (PSG) a) Alignment of voltage gated sodium channel alpha subunits (DI, DII,DIII & DIV) p-loop regions. Mutations conferring resistance are coloured in green (pufferfish), orange (salamander), purple (clam) and blue (octopus). Susceptible mutations at the same site are Black and bolded. Sites which may be involved with resistance are in bold. **b)** Schematic of voltage-gated sodium channel (Na_v) alpha subunits (DI, DII, DIII and DIV). Each unit is composed of six subunits 1-4 (blue) and 5-6 (yellow). Alternating extra and intercellular loops are shown in black with the p-loops between subunits 5 and 6 highlighted in red. Mutations conferring resistance are shown within black circles on p-loops.

Microbiome of the PSG

TTX is produced through a wide variety of bacteria, which are common in marine sediments and have been isolated from organisms such as pufferfish[13,73,74].

Sequestration of TTX is not exclusive to the blue-ringed octopus among molluscs. Gastropods such as *Pleurobranchaea maculata*[75] and *Niotha clathrata*[16], as well as some bivalves, are capable of sequestering the similar toxin STX[70].

The commonly held hypothesis for TTX acquisition within *Hapalochlaena* is that it is bacterial in origin, and is either ingested or endosymbiotic[75,76]. Analysis of a ribo-depleted RNA sample from the PSG of *H. maculosa* revealed a highly diverse composition of genera with Simpson's and Shannon's diversity indices of 4.77 and 0.94, respectively. The dominant phyla were Proteobacteria and Firmicutes, composing respectively 41% and 22% of overall bacterial species detected (Fig 5a-b). To date, 151 strains of TTX producing bacteria have been identified from 31 genera. Of these, 104 are members of Proteobacteria[77]. The genera *Pseudomonas* and *Bacillus* belonging to the phyla Proteobacteria and Firmicutes, respectively, have been previously identified in the PSG of *Hapalochlaena* sp *(Octopus maculosus)*[76]. Examination of these bacterial strains revealed TTX production, and extracts injected into mice proved to be lethal[76]. A more recent study on the bacterial composition of *H. maculosa* PSG did not identify TTX producing strains[75]. However, only a small subset of the many strains identified were tested. Diversity of bacterial genera much like the *H. maculosa* in this study was

high and this may complicate identification of species responsible for TTX production. The biosynthetic pathway of TTX has yet to be elucidated, and as a result, only culturable bacterial species can be tested for TTX production.

Fig 6. Assessment of bacteria within the posterior salivary gland of *H. maculosa* (PSG). **a)** Bacterial composition at the phylum level of a *H. maculosa* posterior salivary/venom gland. **b)** Composition of the largest Phylum, Protobacteria of a *H. maculosa* posterior salivary/venom gland.

Conclusions

This work describes the genome of a unique TTX bearing mollusc genome, the southern blue-ringed octopus (*Hapalochlaena maculosa*). Much of cephalopod evolution is barely understood due to sparseness of genomic data. Our analysis provides the first glimpse into genomic changes underlying genome evolution of closely related octopod species. While the size, heterozygosity and repetitiveness of the blue ring genome is congruent with previously published octopod genomes, we find similar yet independent expansions of key neuronal gene families across all three species and show evidence for the involvement of gene novelty in the evolution of key neuronal, reproductive, and

sensory tissues. The evolution of venom in octopods also differs between species, with *H. maculos*a showing a reduction in the number and expression of serine proteases in their venom gland relative to the other octopods in this study. Inclusion of TTX in *H. maculosa* distinguishes this species from related octopods and is believed to impact toxin recruitment and retention, as the highly potent TTX is sufficient to subdue common octopod prey without additional toxins.

Acknowledgements

We thank Dr Mark Norman and Colin Silvey for aiding Dr Julian Finn in collection of the two blue-ringed octopus specimens. We thank Jacqui Stuart for her brilliant illustrations and work on Fig beautification. This work was supported by an Australian Biological Resources Study (ABRS) grant (ref:RF211-41).

Ethics declaration

Animal Ethics Approval

Field collection of fishes, cephalopods (nautiluses, squids, cuttlefishes and octopuses} and decapod crustaceans (crabs, lobsters, crayfishes and their allies) in for Museum Victoria” (Animal Ethics Committee: Museums Victoria; AEC Approval Number: 10006)

Competing interests

Authors have no conflicts/competing interests to declare.

Data availability statement:

Genomic and transcriptomic data produced and used in this paper have been made available in the NCBI BioProject: PRJNA602771 under the following accession numbers: raw transcriptome(SAMN13930963 - SAMN13930975), genome assembly(to be provided). Voucher specimen for the transcriptome is stored at Melbourne museum (number to be provided). (Data is not yet available om NCBI as we would like to release it on publication).

Code availability statement:

Custom scripts and pipelines used in all of the analyses are published in GitHub(https://github.com/blwhitelaw/BRO_annotation).

Abbreviations

TTX: Tetrodotoxin, STX: Saxitoxin, PSG: Posterior Salivary Gland, CHGN:Chondroitin N-acetylgalactosaminyltransferase, C2H2(Cys2-His2) zinc finger, Cornifin SPRR:Small Proline-Rich Proteins, LINE: Long Interspersed Nuclear Element, SINE: Short Interspersed Nuclear Element, Mya: Million Years Ago, BUSCO: Benchmarking Universal Single-Copy Orthologs, PSMC: *Pairwise Sequentially Markovian Coalescent* and MSMC:multiple sequentially Markovian coalescent

Methods

Genome sequencing and assembly

DNA was extracted from a single *H. maculosa* female collected at Port Phillip Bay, Victoria, Australia. Two types of Illumina libraries were constructed, standard paired end and Illumina mate pairs (Supplementary data 2). Dovetail sequencing, Chicago

libraries improved upon original sequencing resulting in an overall coverage of 71X. Assembly-stats (https://github.com/sanger-pathogens/assembly-stats) was used to ascertain the quality of the assembly and relevant metrics (Supplementary notes 1).

Transcriptome sequencing

The *H. maculosa* transcriptome was generated using 12 tissues (brain, anterior salivary gland, digestive gland, renal, brachial heart, male reproductive tract, systemic heart, eyeballs, gills, posterior salivary gland, dorsal mantle and ventral mantle tissue). RNA was extracted using the Qiagen RNeasy kit. Libraries were constructed using 3 μg of RNA at a concentration of >100 ng/μ L. Each tissue was sequenced on 1/12th of an Illumina HiSeq2000 lane with one lane used in total.

De novo transcriptome assembly

De novo assembly of the *H. maculosa* transcriptome was conducted using sequencing data from 11 tissues (as listed above) and Trinity (v10.11.201). Default parameters were used aside from kmer coverage, which was set to three to account for the large

data volume. Protein coding sequences were identified using Trinotate[78] and domains assigned by Interpro[79].

Genome annotation

Genes were annotated using a *de novo* predictor supplemented with transcriptomic evidence. Training models were produced by PASA[25] using a transcriptome composed of 12 tissues (as listed above) and supplied to the *de novo* predictor Augustus[26] along with intron, exon and repeat hints (generated by repeatmasker). Alternative splicing of gene models was also predicted using PASA. Methods used for annotation have been documented in the git https://github.com/blwhitelaw/BRO_annotation. Additional genes were predicted by mapping raw expressed reads against the genome. Functional annotation of gene models was achieved using InterPro v72.0[79]. Completeness of genes was assessed using BUSCO v3 Metazoan database[27].

Heterozygosity

JELLYFISH v2.2.1 was used in conjunction with GenomeScope [80] to calculate heterozygosity in *H. maculosa* using a kmer frequency of 21 (Supplementary table 5).

Repetitive and transposable elements

Repetitive and transposable elements were annotated using RepeatModeler v1.0.9 (RepeatScout) and masking performed with RepeatMasker v4.0.8[81](Supplementary notes 3.3). Analysis of gene associated TE was conducted by extracting TE within flanking regions 10K upstream and downstream of genes using Bedtools v2.27.1.

Calibration of sequence divergence with respect to time

Divergence times between the molluscan genomes (*Crassostrea gigas, Lottia gigantea, Aplysia californica, Euprymna scolopes, Octopus bimaculoides, Callistoctopus minor* and *Hapalochlaena maculosa*) and transcriptomes (*Sepia officinalis, idiosepius notoides, Octopus kaurna* and *Octopus vulgaris*) was obtained using a mutual best hit (MBH) approach. Whole genomes and transcriptomes were BLASTed against *Octopus bimaculoides*. The resulting hits were filtered, and alignments shared between all species extracted. A maximum likelihood phylogeny was generated using RAxML v8.0[82]. Phylobayes v3.3[83] was used to calculate divergence times (Supplementary 4.1)

Effective population size (PSMC)

Historical changes in effective population size were estimated using PSMC implemented in the software MSMC[84,85]. To generate inputs for MSMC we selected a subset of the reads used for genome assembly corresponding to 38x coverage of reads from libraries with short (500bp) insert sizes. These were pre-processed according to GATK best practices; briefly, adapters were marked with Picard 2.2.1, reads were mapped to the *H. maculosa* genome using bwa mem v 0.7.17 and PCR duplicates identified using Picard v2.2.1. In order to avoid inaccuracies due to poor coverage or ambiguous read mapping we masked regions where short reads would be unable to find unique matches using SNPable (http://lh3lh3.users.sourceforge.net/snpable.shtml) and where coverage was more than double or less than half the genome wide average of 38x. Variant sites were called within unmasked regions and results converted to MSMC input format using msmc-tools https://github.com/stschiff/msmc-tools. All data for *H. maculosa* scaffolds of length greater than 1Mb was then used to generate 100 bootstrap replicates by dividing data into 500kb chunks and assembling them into 20 chromosomes with 100 chunks each. We then ran msmc2 on each bootstrap replicate and assembled imported the resulting data into R for plotting. A mutation rate of 2.4e-

9 per base per year and a generation time of 1 year were assumed in order to set a timescale in years and convert coalescence rates to effective population size.

Mutation rate

Mutation rate was calculated by extracting orthologous genes from *O. bimaculoides* and *H. maculosa.* Neutrality was assumed for genes with very low expression (> 10 TMP across all tissues). Neutral genes were aligned using MAFFT v7.407[86] and codeml[87] was used to calculate substitution metrics (dS). Per base neutral substitution between lineages was determined using the mean dS value divided by divergence time (refer to *Calibration of sequence divergence with respect to time*) usually over number of generations, however *H. maculosa* is a single generation species. As octopus are diploid the rate was divided by two. Divergence between species was calculated using Phylobayes v3.3[83].

Quantifying gene expression/ specificity

Gene expression within individual tissues was calculated using Kallisto[88] for the transcriptomic data sets of *H. maculosa*, *O. bimaculoides* and *C. minor*. Defaults were

used and counts were calculated as TPM. Gene specificity was defined as any gene with a tau value > 0.80.

Gene model expression dynamics

Patterns of gene expression and loss were assessed across octopod genomes at differing taxonomic/organismal levels. Gene models were classified as species specific, octopod specific or non-specific (orthologous to a gene outside of octopods). Expression at each level was determined using whole transcriptomes from all tissues of each species. Genes with expression within one or more tissues was determined to be expressed, loss of expression was classified as a gene with a single ortholog in each species, which is expressed in one or more species and not expressed in the remaining species.

Dynamics of PSG gene expression

In order to identify patterns of PSG specific gene expression (losses and shifts) between the three available octopod genomes, genes with expression specific to the PSG of each species were examined separately. Specific gene expression was defined as a tau value > 0.8. Orthologous groups were identified between species using Orthovenn2[89] and

sequences which were identified as species specific were confirmed using BLAST. Types of expressions were categorized as follows: A loss of expression requires a gene to be present in all three octopods with and expressed in one or more species while having no detectable expression in at least one species. A shift in expression occurs when an ortholog present in all species is expressed in different tissues.

The role of the Nav in TTX resistance

Sodium channels for the three octopus genomes along with all available in-house cephalopod transcriptomes were extracted manually using a series of BLAST searches against the nr database. Annotation was achieved using Interpro vv72.0[79] and identification and extraction of p-loop regions of the sodium channel alpha subunit were manually performed. Where sodium channels were incomplete alignment against related complete channels were used to extract the p-loop regions. Individual mutation with potential to confer resistance were identified manually in Geneious v10.1 (https://www.geneious.com).

Microbiome of PSG

A single ribo-depleted RNA sample of *H. maculosa* PSG was examined using the SAMSA2 pipeline[90] to identify the bacterial composition and corresponding molecular functions. Two databases were used Subsys and NCBI RefBac. The Krona package[91] was used to produce visualizations of each dataset.

Supplementary Information

Supplementary Notes 1-8, Supplementary Tables 1-8, Supplementary Figs 1-10

Supplementary Data 2: Table of genomic Illumina library insert sizes

References

1 Ritschard, E. A. *et al.* Coupled Genomic Evolutionary Histories as Signatures of Organismal Innovations in Cephalopods. *BioEssays* (2019).

2 Tanner, A. R. *et al.* Molecular clocks indicate turnover and diversification of modern coleoid cephalopods during the Mesozoic Marine Revolution. *Proceedings of the Royal Society B: Biological Sciences* **284**, 20162818 (2017).

3 Jereb, P., Roper, C. F. E., Norman, M. D., and Finn, J. K. Cephalopods of the world. An annotated and illustrated catalogue of cephalopod species known to date," in Octopods and Vampire Squids, Vol. 3,. *Rome: FAO* (2014).

4 Graziadei, P. & Gagne, H. Sensory innervation in the rim of the octopus sucker. *Journal of morphology* **150**, 639-679 (1976).

5 Froesch, D. Projection of chromatophore nerves on the body surface of Octopus vulgaris. *Marine Biology* **19**, 153-155 (1973).

6 Budelmann, B. in *The nervous systems of invertebrates: An evolutionary and comparative approach* 115-138 (Springer, 1995).

7 Gray, E. & Young, J. Electron microscopy of synaptic structure of Octopus brain. *The Journal of cell biology* **21**, 87-103 (1964).
8 Fingerhut, L. C. *et al.* Shotgun proteomics analysis of saliva and salivary gland tissue from the common octopus Octopus vulgaris. *Journal of proteome research* **17**, 3866-3876 (2018).
9 Ghiretti, F. Toxicity of octopus saliva against Crustacea. *Annals of the New York Academy of Sciences* **90**, 726-741 (1960).
10 Cooke, I. R., Whitelaw, B., Norman, M., Caruana, N. & Strugnell, J. M. Toxicity in cephalopods. *Evolution of Venomous Animals and Their Toxins*, 1-15 (2015).
11 Crone, H., Leake, B., Jarvis, M. & Freeman, S. E. On the nature of "Maculotoxin", a toxin from the blue-ringed octopus (Hapalochlaena maculosa). *Toxicon* **14**, 423-426 (1976).
12 Fry, B., Roelants, K. & Norman, J. Tentacles of venom: toxic protein convergence in the Kingdom Animalia. *Journal of molecular evolution* **68**, 311-321 (2009).
13 Wu, Z. *et al.* Toxicity and distribution of tetrodotoxin-producing bacteria in puffer fish Fugu rubripes collected from the Bohai Sea of China. *Toxicon* **46**, 471-476 (2005).
14 Tsuruda, K. *et al.* Secretory glands of tetrodotoxin in the skin of the Japanese newt Cynops pyrrhogaster. *Toxicon* **40**, 131-136 (2002).
15 Wakely, J. F., Fuhrman, G. J., Fuhrman, F. A., Fischer, H. G. & Mosher, H. S. The occurrence of tetrodotoxin (tarichatoxin) in Amphibia and the distribution of the toxin in the organs of newts (Taricha). *Toxicon* **3**, 195-203 (1966).
16 Cheng, C. *et al.* Microflora and tetrodotoxin-producing bacteria in a gastropod, Niotha clathrata. *Food and Chemical Toxicology* **33**, 929-934 (1995).
17 Williams, B. L. & Caldwell, R. L. Intra-organismal distribution of tetrodotoxin in two species of blue-ringed octopuses (Hapalochlaena fasciata and H. lunulata). *Toxicon* **54**, 345-353 (2009).
18 Yotsu-Yamashita, M., Mebs, D. & Flachsenberger, W. Distribution of tetrodotoxin in the body of the blue-ringed octopus (Hapalochlaena maculosa). *Toxicon* **49**, 410-412 (2007).
19 Mäthger, L. M., Bell, G. R., Kuzirian, A. M., Allen, J. J. & Hanlon, R. T. How does the blue-ringed octopus (Hapalochlaena lunulata) flash its blue rings? *Journal of Experimental Biology* **215**, 3752-3757 (2012).
20 Williams, B. L., Stark, M. R. & Caldwell, R. L. Microdistribution of tetrodotoxin in two species of blue-ringed octopuses (Hapalochlaena lunulata and Hapalochlaena fasciata) detected by fluorescent immunolabeling. *Toxicon* **60**, 1307-1313 (2012).
21 Savage, I. & Howden, M. Hapalotoxin, a second lethal toxin from the octopus Hapalochlaena maculosa. *Toxicon* **15**, 463-466 (1977).
22 Whitelaw, B. L. *et al.* Combined transcriptomic and proteomic analysis of the posterior salivary gland from the southern blue-ringed octopus and the southern sand octopus. *Journal of proteome research* **15**, 3284-3297 (2016).
23 Kanda, A., Iwakoshi-Ukena, E., Takuwa-Kuroda, K. & Minakata, H. Isolation and characterization of novel tachykinins from the posterior salivary gland of the common octopus Octopus vulgaris. *Peptides* **24**, 35-43 (2003).
24 Ruder, T. *et al.* Functional characterization on invertebrate and vertebrate tissues of tachykinin peptides from octopus venoms. *Peptides* **47**, 71-76 (2013).
25 Haas, B. J. *et al.* Improving the Arabidopsis genome annotation using maximal transcript alignment assemblies. *Nucleic acids research* **31**, 5654-5666 (2003).
26 Stanke, M. *Gene prediction with a hidden Markov model*, University of Göttingen, (2004).
27 Waterhouse, R. M. *et al.* BUSCO applications from quality assessments to gene prediction and phylogenomics. *Molecular biology and evolution* **35**, 543-548 (2017).
28 Zarrella, I. *et al.* The survey and reference assisted assembly of the Octopus vulgaris genome. *Scientific data* **6**, 13 (2019).

29 Albertin, C. B. *et al.* The octopus genome and the evolution of cephalopod neural and morphological novelties. *Nature* **524**, 220-224, doi:10.1038/nature14668 (2015).
30 Cai, H. *et al.* A draft genome assembly of the solar-powered sea slug Elysia chlorotica. *Scientific data* **6**, 190022 (2019).
31 Liu, C. *et al.* The genome of the golden apple snail Pomacea canaliculata provides insight into stress tolerance and invasive adaptation. *GigaScience* **7**, giy101 (2018).
32 Du, X. *et al.* The pearl oyster Pinctada fucata martensii genome and multi-omic analyses provide insights into biomineralization. *GigaScience* **6**, gix059 (2017).
33 Sun, J. *et al.* Adaptation to deep-sea chemosynthetic environments as revealed by mussel genomes. *Nature ecology & evolution* **1**, 0121 (2017).
34 Uliano-Silva, M. *et al.* A hybrid-hierarchical genome assembly strategy to sequence the invasive golden mussel, Limnoperna fortunei. *GigaScience* **7**, gix128 (2017).
35 Zhang, G. *et al.* The oyster genome reveals stress adaptation and complexity of shell formation. *Nature* **490**, 49 (2012).
36 Jiao, W. *et al.* High-resolution linkage and quantitative trait locus mapping aided by genome survey sequencing: building up an integrative genomic framework for a bivalve mollusc. *DNA research* **21**, 85-101 (2013).
37 Garton, D. W. & Haag, W. R. Heterozygosity, shell length and metabolism in the European mussel, Dreissena polymorpha, from a recently established population in Lake Erie. *Comparative Biochemistry and Physiology Part A: Physiology* **99**, 45-48 (1991).
38 Simmons, M. J. & Crow, J. F. Mutations affecting fitness in Drosophila populations. *Annual review of genetics* **11**, 49-78 (1977).
39 Kumar, S. & Subramanian, S. Mutation rates in mammalian genomes. *Proceedings of the National Academy of Sciences* **99**, 803-808 (2002).
40 Hayward, B. W., Kawagata, S., Grenfell, H. R., Sabaa, A. T. & O'Neill, T. Last global extinction in the deep sea during the mid-Pleistocene climate transition. *Paleoceanography and Paleoclimatology* **22** (2007).
41 Hofreiter, M. & Stewart, J. Ecological change, range fluctuations and population dynamics during the Pleistocene. *Current biology* **19**, R584-R594 (2009).
42 Reynolds, T. V., Matthee, C. A. & Von Der Heyden, S. The influence of Pleistocene climatic changes and ocean currents on the phylogeography of the southern African barnacle, Tetraclita serrata (Thoracica; Cirripedia). *PloS one* **9**, e102115 (2014).
43 Huber, C. D., DeGiorgio, M., Hellmann, I. & Nielsen, R. Detecting recent selective sweeps while controlling for mutation rate and background selection. *Molecular ecology* **25**, 142-156 (2016).
44 Mao, Y., Economo, E. P. & Satoh, N. The roles of introgression and climate change in the rise to dominance of Acropora Corals. *Current Biology* **28**, 3373-3382. e3375 (2018).
45 Kim, B.-M. *et al.* Antarctic blackfin icefish genome reveals adaptations to extreme environments. *Nature ecology & evolution* **3**, 469 (2019).
46 Guzik, M. T., Norman, M. D. & Crozier, R. H. Molecular phylogeny of the benthic shallow-water octopuses (Cephalopoda: Octopodinae). *Molecular Phylogenetics and Evolution* **37**, 235-248, doi:10.1016/j.ympev.2005.05.009 (2005).
47 Strugnell, J., Jackson, J., Drummond, A. J. & Cooper, A. Divergence time estimates for major cephalopod groups: evidence from multiple genes. *Cladistics* **22**, 89-96 (2006).
48 Strugnell, J. M., Rogers, A. D., Prodöhl, P. A., Collins, M. A. & Allcock, A. L. The thermohaline expressway: the Southern Ocean as a centre of origin for deep-sea octopuses. *Cladistics* **24**, 853-860 (2008).
49 Lindgren, A. R. & Anderson, F. E. Assessing the utility of transcriptome data for inferring phylogenetic relationships among coleoid cephalopods. *Molecular phylogenetics and evolution* **118**, 330-342 (2018).

50 Fox, J. W. & Serrano, S. M. Structural considerations of the snake venom metalloproteinases, key members of the M12 reprolysin family of metalloproteinases. *Toxicon* **45**, 969-985 (2005).
51 Ruder, T. *et al.* Molecular phylogeny and evolution of the proteins encoded by coleoid (cuttlefish, octopus, and squid) posterior venom glands. *Journal of molecular evolution* **76**, 192-204 (2013).
52 Grisley, M. & Boyle, P. Bioassay and proteolytic activity of digestive enzymes from octopus saliva. *Comparative Biochemistry and Physiology Part B: Comparative Biochemistry* **88**, 1117-1123 (1987).
53 Key, L., Boyle, P. & Jaspars, M. Novel activities of saliva from the octopus Eledone cirrhosa (Mollusca; Cephalopoda). *Toxicon* **40**, 677-683 (2002).
54 McDonald, N. & Cottrell, G. Purification and mode of action of toxin from Eledone cirrosa. *Comparative and general pharmacology* **3**, 243-248 (1972).
55 Bordon, K. C., Perino, M. G., Giglio, J. R. & Arantes, E. C. Isolation, enzymatic characterization and antiedematogenic activity of the first reported rattlesnake hyaluronidase from Crotalus durissus terrificus venom. *Biochimie* **94**, 2740-2748 (2012).
56 Frank, H. Y. & Catterall, W. A. Overview of the voltage-gated sodium channel family. *Genome biology* **4**, 207 (2003).
57 How, C.-K., Chern, C.-H., Huang, Y.-C., Wang, L.-M. & Lee, C.-H. Tetrodotoxin poisoning. *The American journal of emergency medicine* **21**, 51-54 (2003).
58 Brodie III, E. D. & Brodie Jr, E. D. Tetrodotoxin resistance in garter snakes: an evolutionary response of predators to dangerous prey. *Evolution* **44**, 651-659 (1990).
59 Nakamura, M. & Yasumoto, T. Tetrodotoxin derivatives in puffer fish. *Toxicon* **23**, 271-276 (1985).
60 Geffeney, S. L. *et al.* Convergent and parallel evolution in a voltage-gated sodium channel underlies TTX-resistance in the Greater Blue-ringed Octopus: Hapalochlaena lunulata. *Toxicon* (2019).
61 Jost, M. C. *et al.* Toxin-resistant sodium channels: parallel adaptive evolution across a complete gene family. *Molecular biology and evolution* **25**, 1016-1024 (2008).
62 Feldman, C. R., Brodie, E. D. & Pfrender, M. E. Constraint shapes convergence in tetrodotoxin-resistant sodium channels of snakes. *Proceedings of the National Academy of Sciences* **109**, 4556-4561 (2012).
63 Du, Y., Nomura, Y., Liu, Z., Huang, Z. Y. & Dong, K. Functional expression of an arachnid sodium channel reveals residues responsible for tetrodotoxin resistance in invertebrate sodium channels. *Journal of Biological Chemistry* **284**, 33869-33875 (2009).
64 Jeziorski, M., Greenberg, R. & Anderson, P. Cloning of a putative voltage-gated sodium channel from the turbellarian flatworm Bdelloura candida. *Parasitology* **115**, 289-296 (1997).
65 Geffeney, S. L., Fujimoto, E., Brodie III, E. D., Brodie Jr, E. D. & Ruben, P. C. Evolutionary diversification of TTX-resistant sodium channels in a predator–prey interaction. *Nature* **434**, 759 (2005).
66 Flachsenberger, W. & Kerr, D. Lack of effect of tetrodotoxin and of an extract from the posterior salivary gland of the blue-ringed octopus following injection into the octopus and following application to its brachial nerve. *Toxicon* **23**, 997-999 (1985).
67 Lopes, V. M., Baptista, M., Repolho, T., Rosa, R. & Costa, P. R. Uptake, transfer and elimination kinetics of paralytic shellfish toxins in common octopus (Octopus vulgaris). *Aquatic toxicology* **146**, 205-211 (2014).
68 Ulbricht, W., Wagner, H. H. & Schmidtmayer, J. Kinetics of TTX-STX Block of Sodium Channels. *Annals of the New York Academy of Sciences* **479**, 68-83 (1986).
69 Monteiro, A. & Costa, P. R. Distribution and selective elimination of paralytic shellfish toxins in different tissues of Octopus vulgaris. *Harmful Algae* **10**, 732-737 (2011).

70 Li, S.-C., Wang, W.-X. & Hsieh, D. Feeding and absorption of the toxic dinoflagellate Alexandriumtamarense by two marine bivalves from the South China Sea. *Marine Biology* **139**, 617-624 (2001).
71 Braid, H. E. *et al.* Preying on commercial fisheries and accumulating paralytic shellfish toxins: a dietary analysis of invasive Dosidicus gigas (Cephalopoda Ommastrephidae) stranded in Pacific Canada. *Marine biology* **159**, 25-31 (2012).
72 Shea, E. K., Ziegler, A., Faber, C. & Shank, T. M. Dumbo octopod hatchling provides insight into early cirrate life cycle. *Current Biology* **28**, R144-R145 (2018).
73 Lee, M.-J. *et al.* A tetrodotoxin-producing Vibrio strain, LM-1, from the puffer fish Fugu vermicularis radiatus. *Appl. Environ. Microbiol.* **66**, 1698-1701 (2000).
74 Yotsu, M. *et al.* Production of tetrodotoxin and its derivatives by Pseudomonas sp. isolated from the skin of a pufferfish. *Toxicon* **25**, 225-228 (1987).
75 Chau, R., Kalaitzis, J., Wood, S. & Neilan, B. Diversity and biosynthetic potential of culturable microbes associated with toxic marine animals. *Marine drugs* **11**, 2695-2712 (2013).
76 Hwang, D. *et al.* Tetrodotoxin-producing bacteria from the blue-ringed octopus Octopus maculosus. *Marine Biology* **100**, 327-332 (1989).
77 Magarlamov, T., Melnikova, D. & Chernyshev, A. Tetrodotoxin-producing bacteria: Detection, distribution and migration of the toxin in aquatic systems. *Toxins* **9**, 166 (2017).
78 Bryant, D. M. *et al.* A tissue-mapped axolotl de novo transcriptome enables identification of limb regeneration factors. *Cell reports* **18**, 762-776 (2017).
79 Jones, P. *et al.* InterProScan 5: genome-scale protein function classification. *Bioinformatics* **30**, 1236-1240 (2014).
80 Vurture, G. W. *et al.* GenomeScope: fast reference-free genome profiling from short reads. *Bioinformatics* **33**, 2202-2204 (2017).
81 Smit, A., Hubley, R & Green, P. *RepeatMasker Open-4.0.*, <<http://www.repeatmasker.org>. > (2013-2015).
82 Stamatakis, A. RAxML-VI-HPC: maximum likelihood-based phylogenetic analyses with thousands of taxa and mixed models. *Bioinformatics* **22**, 2688-2690 (2006).
83 Guindon, S. & Gascuel, O. A simple, fast, and accurate algorithm to estimate large phylogenies by maximum likelihood. *Systematic biology* **52**, 696-704 (2003).
84 Li, H. & Durbin, R. Inference of human population history from whole genome sequence of a single individual. *Nature* **475**, 493 (2012).
85 Schiffels, S. & Durbin, R. Inferring human population size and separation history from multiple genome sequences. *Nature genetics* **46**, 919 (2014).
86 Rozewicki, J., Li, S., Amada, K. M., Standley, D. M. & Katoh, K. MAFFT-DASH: integrated protein sequence and structural alignment. *Nucleic acids research* **47**, W5-W10 (2019).
87 Adachi, J. & Hasegawa, M. *MOLPHY version 2.3: programs for molecular phylogenetics based on maximum likelihood*. (Institute of Statistical Mathematics Tokyo, 1996).
88 Bray, N. L., Pimentel, H., Melsted, P. & Pachter, L. Near-optimal probabilistic RNA-seq quantification. *Nature biotechnology* **34**, 525-527 (2016).
89 Xu, L. *et al.* OrthoVenn2: a web server for whole-genome comparison and annotation of orthologous clusters across multiple species. *Nucleic acids research* **47**, W52-W58 (2019).
90 Westreich, S. T., Korf, I., Mills, D. A. & Lemay, D. G. SAMSA: a comprehensive metatranscriptome analysis pipeline. *BMC bioinformatics* **17**, 399 (2016).
91 Ondov, B. D., Bergman, N. H. & Phillippy, A. M. Interactive metagenomic visualization in a Web browser. *BMC bioinformatics* **12**, 385 (2011).

Figure1

Click here to download Figure Fig1.pdf

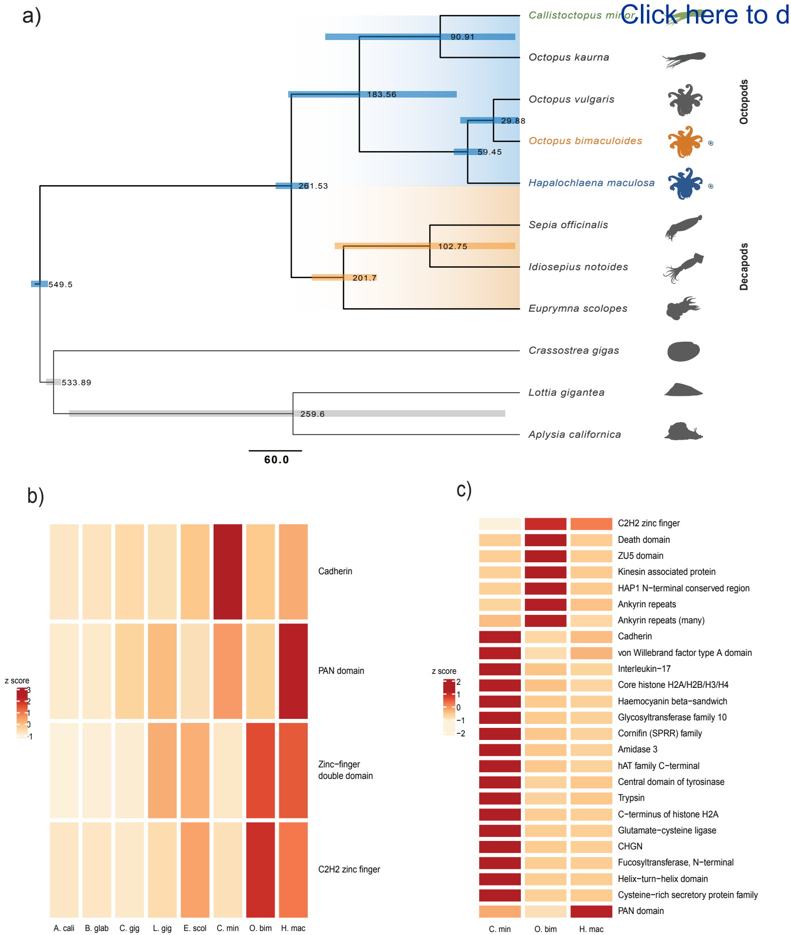

Figure2

Click here to download Figure Fig2.pdf

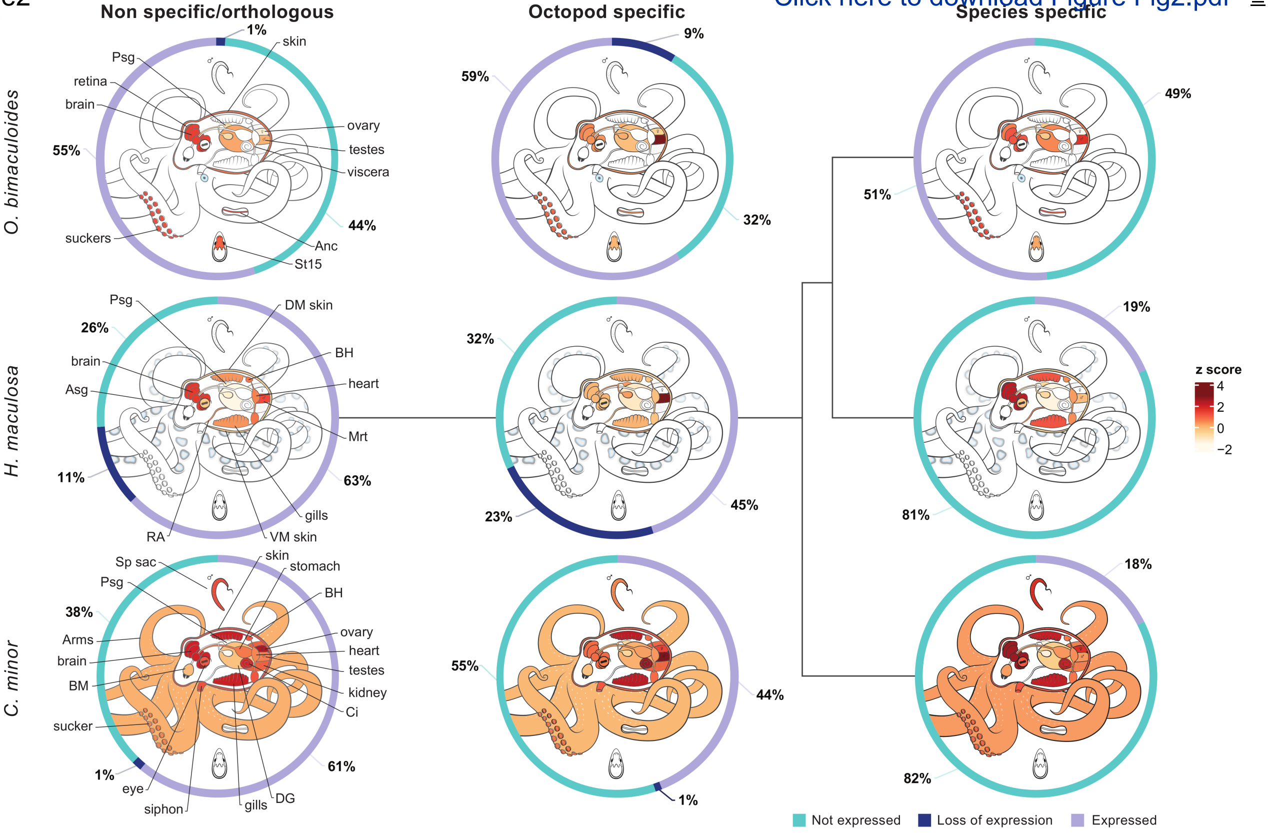

Figure3

Click here to download Figure Fig3_V2.pdf

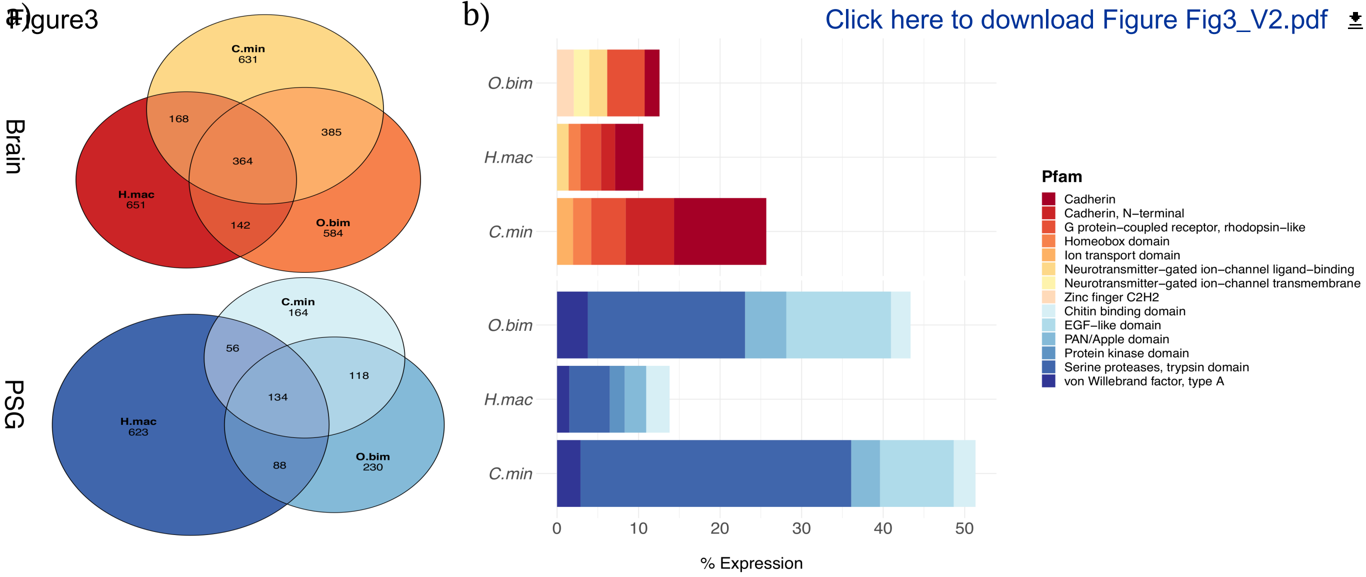

Figure4

Click here to download Figure Fig4.pdf

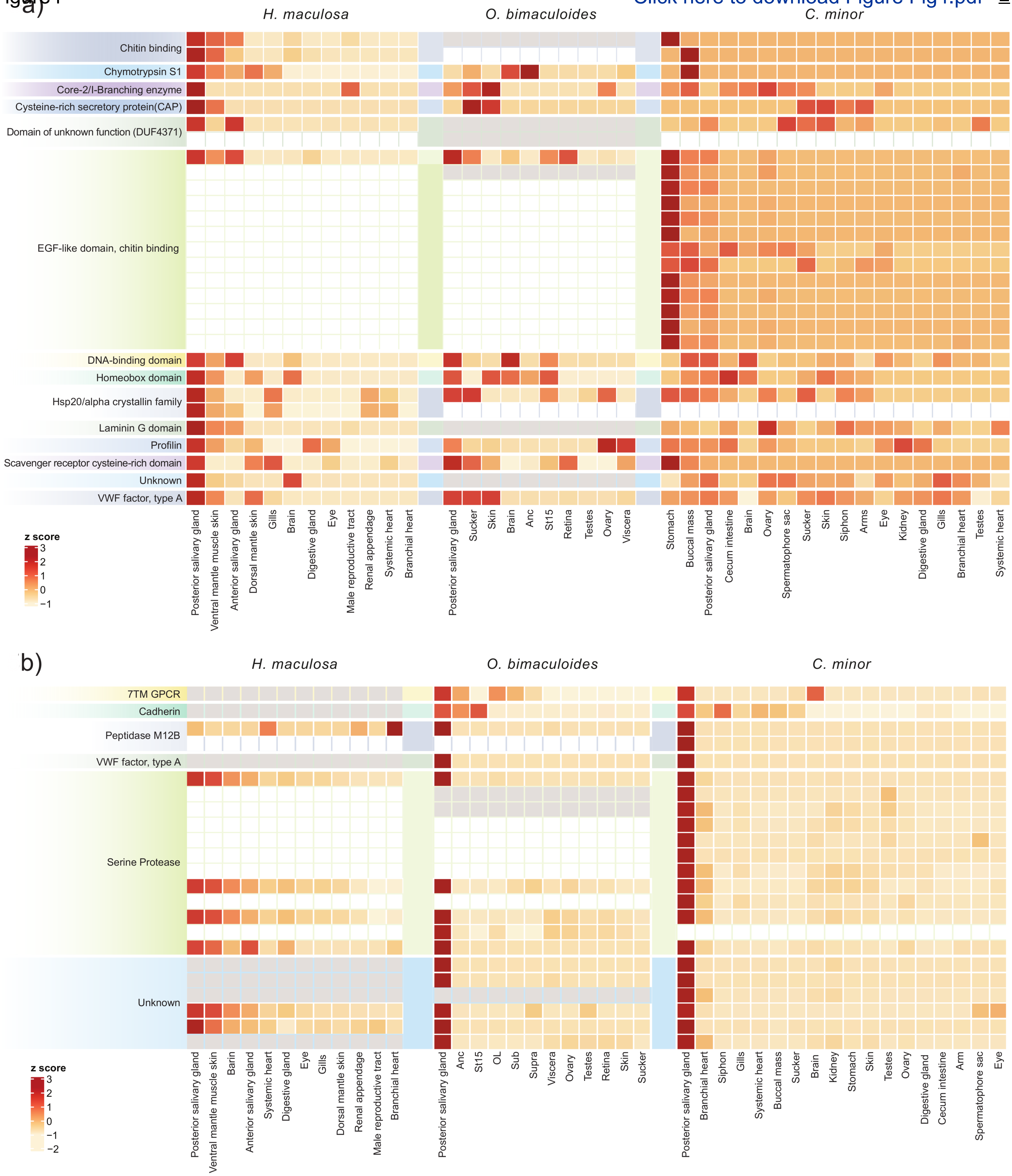

Figure5

Click here to download Figure Fig5_V2.pdf

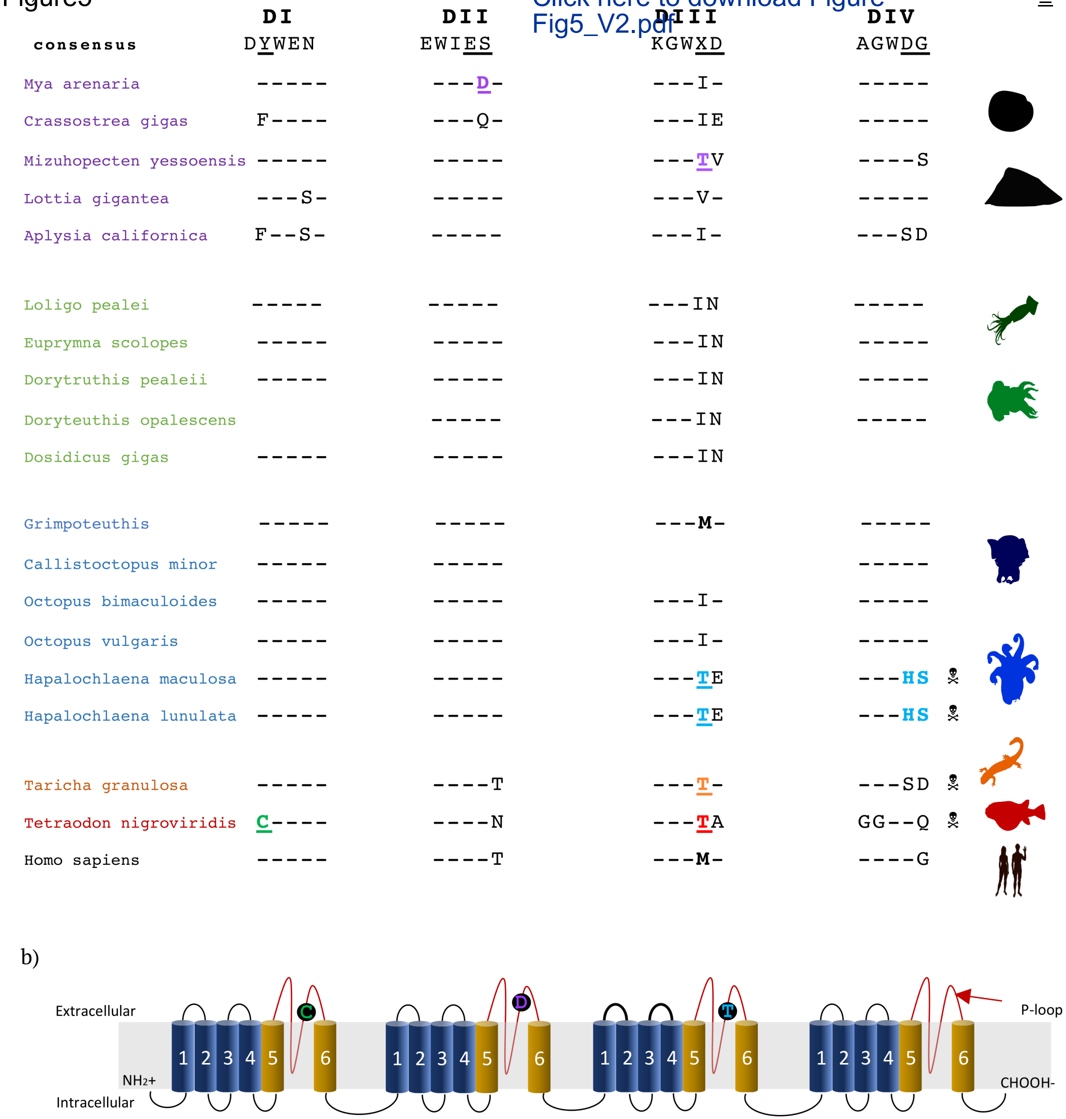

Figure6

Click here to download Figure Fig6_V2.pdf

a)

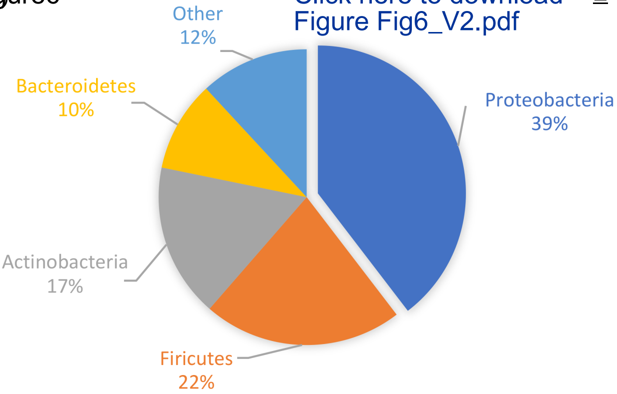

b)

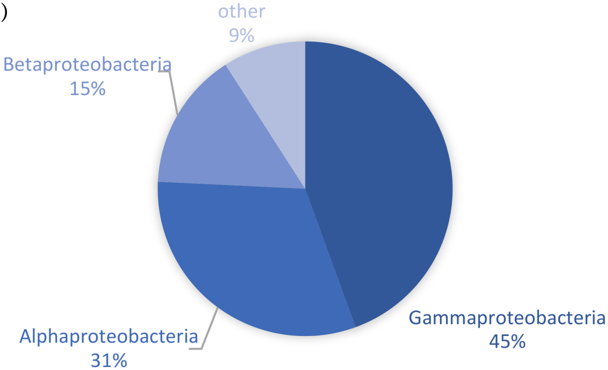

Supplementary Notes

Click here to access/download
Supplementary Material
Supplementry_Notes.docx

Supplementary Material(insert sizes)

Click here to access/download
Supplementary Material
BRO Sequencing Insert Sizes.xlsx

Cairns
Singapore
Townsville

JAMES COOK UNIVERSITY
AUSTRALIA

Dear Dr. Goodman

I am pleased to submit an original research piece titled "**Adaptive venom evolution and toxicity in octopods is driven by extensive novel gene formation, expansion and loss** " for consideration to be published in *GigaScience*.

Much of cephalopod evolution remains unknown due to sparseness of their genomic sampling. Cephalopod genomes are some of the largest and most repetitive animal genomes and exhibit drastically different evolutionary trajectories relative to other better documented lineages. A more focused genomic study to reveal how individual genomic changes are associated with the evolution of novel organs, tissues, or adaptations, within a single group of cephalopods has been missing so far. We present such a study, focussing on adaptations in the toxic blue-ringed octopus the *Hapalochlaena maculosa*, for which we provide a high quality genome assembly based on multiple technologies. Members of the genus *Hapalochlaena* are the only octopods to contain the lethal neurotoxin, tetrodotoxin (TTX), within their venom and tissues and are a prime example of the origin of evolutionary novelties within octopods.

Using global comparative genomics approaches and focused study on TTX evolution, we report key findings:

- Gene family expansions crucial for the development of complex neural networks are present in cephalopods, yet are differentially expanding in all three octopod species
- Novel gene formation at different phylogenetic levels can be associated with evolution in a specific set of cephalopod tissues
- Changes in Posterior Salivary Gland composition (PSG) between TTX bearing and non-TTX bearing species
- Convergently evolved mutations consistent with TTX resistance detected in *H. maculosa*

We firmly believe that our manuscript is suited for publication by *GigaScience* as one of the first to explore the evolutionary genomic basis for novelties within octopods and cephalopods in general. Our whole genomic comparisons provide insight into the defining structure/features of octopod genomes at the species-specific level. Additionally, we

examine the impact of TTX on the evolution of venom in *H. maculosa* relative to non-TTX bearing octopods.

Yours sincerely,

Brooke L. Whitelaw
James Cook University,
Townsville, QLD 4810
Mobile: 0424642621
brooke.whitelaw@my.jcu.edu.au

Assoc. Prof. Jan Strugnell
James Cook University,
Townsville, QLD 4810
jan.strugnell.jcu.edu.au

and

Prof. Oleg Simakov
University of Vienna
Universitätsring 1, 1010 Wien, Austria
oleg.simakov@univie.ac.at
