# Supplementary material for: Adaptive venom evolution and toxicity in octopods is driven by extensive novel gene formation, expansion, and loss
Source: Gigascience. 2020 Nov 10;9(11):giaa120. doi: 10.1093/gigascience/giaa120 (PMC7656900; doi:10.1093/gigascience/giaa120)
Supplement: giaa120_GIGA-D-20-00135_Revision_1 [file giaa120_giga-d-20-00135_revision_1.pdf]

// GigaScience

GigaScience

Adaptive venom evolution and toxicity in octopods is driven by extensive novel gene formation, expansion and loss

--Manuscript Draft--

**Manuscript Number:**	GIGA-D-20-00135R1
**Full Title:**	Adaptive venom evolution and toxicity in octopods is driven by extensive novel gene formation, expansion and loss
**Article Type:**	Research
**Funding Information:**	Australian Biological Resources Study (ref:RF211-41) — Assoc Prof Jan M. Strugnell<br>Austrian Science Fund (FWF) grant (P30686-B29) — Prof Oleg Simakov
**Abstract:**	Background<br><br>Cephalopods represent a rich system for investigating the genetic basis underlying organismal novelties. This diverse group of specialized predators has evolved many adaptations including proteinaceous venom. Of particular interest is the blue-ringed-octopus genus (Hapalochlaena), which are the only octopods known to store large quantities of the potent neurotoxin, tetrodotoxin, within their tissues and venom gland.<br><br>Findings<br><br>To reveal genomic correlates of organismal novelties, we conducted a comparative study of three octopod genomes, including the Southern blue-ringed octopus (Hapalochlaena maculosa). We present the genome of this species and reveal highly dynamic evolutionary patterns at both non-coding and coding organizational levels. Gene family expansions previously reported in Octopus bimaculoides (e.g., zinc finger and cadherins, both associated with neural functions), as well as formation of novel gene families, dominate the genomic landscape in all octopods. Examination of tissue-specific genes in the posterior salivary gland (PSG) revealed that expression was dominated by serine proteases in non- tetrodotoxin bearing octopods, while this family was a minor component in H. maculosa . Moreover, voltage-gated sodium channels in H. maculosa contain a resistance mutation found in pufferfish and garter snakes, which is exclusive to the genus. Analysis of the PSG microbiome revealed a diverse array of bacterial species, including genera that can produce tetrodotoxin, suggestive of a possible production source.<br><br>Conclusions<br><br>We present the first tetrodotoxin-bearing octopod genome H. maculosa, which displays lineage-specific adaptations to tetrodotoxin acquisition. This genome, along with other recently published cephalopod genomes, represents a valuable resource from which future work could advance our understanding of the evolution of genomic novelty in this family.
**Corresponding Author:**	Brooke Lauren Whitelaw, Bachelor with Honours and current PhD candidate<br>James Cook University College of Science and Engineering<br>Townsville, QLD AUSTRALIA
**Corresponding Author Secondary Information:**	
**Corresponding Author's Institution:**	James Cook University College of Science and Engineering
**Corresponding Author's Secondary Institution:**	
**First Author:**	Brooke Lauren Whitelaw
**First Author Secondary Information:**	
**Order of Authors:**	Brooke Lauren Whitelaw

Powered by Editorial Manager® and ProduXion Manager® from Aries Systems Corporation

	Ira R. Cooke
	Julian Finn
	Rute R. da Fonseca
	Elena A. Ritschard
	M. T P. Gilbert
	Oleg Simakov
	Jan M. Strugnell
**Order of Authors Secondary Information:**	
**Response to Reviewers:**	Dear Dr. Hongling Zhou

On behalf of my co-authors, I would like to thank you for the opportunity to revise and resubmit our research piece titled "Adaptive venom evolution and toxicity in octopods is driven
by extensive novel gene formation, expansion and loss" (GIGA-D-20-00135).

We would also like thank all three reviewers for their constructive criticism and advice on
how to improve our manuscript. All suggestions have been taken into account and incorporated resulting in a much-improved manuscript.

A response to reviewers has been provided in which we address each point providing the
original text followed by the revised text, including line numbers where changes have been
implemented. We also included a low resolution version of all corrected figures in the response.

Thank you again for your consideration of our revised manuscript

Yours sincerely,
Brooke L. Whitelaw

Reviewer reports:

Reviewer #1: The manuscript describes the genome of the southern blue-ringed octopus and provides comparisons between this genome and previously published octopus genomes, with a particular focus on venom. I did not see any major flaws in the paper, which I think will be a valuable contribution to the cephalopod genomics literature. The blue-ringed octopuses are of major interest due to their highly toxic venom, and I found the comparisons between this species and others in terms of venom production and resistance to be quite illuminating (though somewhat unsurprising, given what we already knew about the use of TTX by this species).

I found the paper to be fairly well written, though I do have several questions and comments that I hope will help clarify some issues. I will list those below, in the order I encountered them as I read the manuscript and supplementary materials. My only somewhat substantive concern first struck me as I read page 17 of the manuscript: "…suggesting a species-specific expansion of this cluster in C. minor". I think the authors should be a bit more careful with how they use the phrase "species-specific". They have included only three octopod species out of 300+ species in this study. Yes, any differences they detect between these species could be species specific, but I think it is more likely that the differences arose in ancestral lineages. For example, expansion of the serine protease cluster may have occurred only in C. minor, but it could also have occurred in the ancestor of Callistoctopus, or in some other ancestor. At present, the authors do not have sufficient sampling to know if any of the expansions, losses, shifts in expression, etc., they are seeing are truly species specific. Similarly, sentences like "Loss of serine protease genes can also be observed in H.

maculosa". The authors can certainly state that H. maculosa has fewer serine protease genes than O. bimaculoides and C. minor, but the *loss* of these genes may have occurred in H. maculosa *or* in any ancestral lineage after the divergence of Hapalochlaena from Octopus. I urge the authors to go through their manuscript carefully to find instances where they have evidence of differences among these species and to check that their descriptions of differences among these species are clear.

We agree with the reviewer and have carefully read through the manuscript and corrected sections where species-specific inferences were made to prevent miscommunication of the findings.

Original text:
“Loss of serine protease genes can also be observed in H. maculosa”

Revised text: (pg. 18, line: 315)
“Fewer serine protease genes can also be observed in H. maculosa”

Original text:
“The greatest proportion of genes in each species examined were not specific to octopods or an octopus species (ancient genes) (Fig 2a). Expression of these genes were enriched in neural tissues across all species, indicating the core conservation of neural development and function. However, we also find that genes specific to each octopod species also show this expression pattern”

Revised text: (pg. 13-14 , lines: 231-235)
“The greatest proportion of genes in each species examined were not specific to octopods or an octopus lineage (ancient genes) (Fig 2a). Expression of these genes were enriched in neural tissues across all species, indicating the core conservation of neural development and function. However, we also find that genes specific to each octopod lineage also show this expression pattern”

Original text:
“Absence of gene expression for genes whose orthologs have retained expression in one or more other species suggests a unique evolutionary trajectory from other octopods. It should be noted that differences in tissue sampling may in part influence these values.”

Revised text: (pg. 14, lines: 244-249)
“Absence of gene expression for genes whose orthologs have retained expression in one or more other species suggests a unique evolutionary trajectory from other octopods. It should be noted that differences in tissue sampling may in part influence these values and due to the limited sampling of species, loss of expression cannot be inferred at a species level and may have occurred at any point in the lineage.”

We have also removed the term ‘species-specific’ and replaced it with the more accurate term ‘lineage-specific’ to avoid confusion. : (pg. 2, line: 45) , (pg. 10, line: 178), (pg. 13, line:229, (pg. 13, line: 224), (pg. 14, line: 239), (pg. 15, line: 255), (pg. 18, line: 314), (pg. 34, line: 589) & (pg. 35, line: 601)

Figure 2 has also been corrected to replace ‘species specific’ with ‘lineage specific’. Low quality version shown here. The corrected version has been uploaded to replace the original

Minor points and suggestions

Check for subject-verb agreement in the abstract. For example, it should be "This diverse group of specilised (sic) predators has evolved…" (the subject is "group", not "predators").

We have corrected the spelling of ‘specialised’ and have replaced ‘have’ with ‘has’ (pg

1, lines: 24).

Abstract: Last sentence might be better as "This genome, along with other recently published cephalopod genomes, represents a valuable resource from which future work could advance our understanding of the evolution of genomic novelty in this family"

We agree that this sentence would fit better at the end of the abstract and have corrected this.

Original sentence:
“This genome along with other recently published cephalopod genomes represent a valuable resource from which future work could advance the evolution of genomic novelty within the family.“

Revised sentence (pg. 2, lines: 45-48):
"This genome, along with other recently published cephalopod genomes, represents a valuable resource from which future work could advance our understanding of the evolution of genomic novelty in this family"

Pg. 3: "underrepresented" - Underrepresented in what sense? In terms of genomic resources?

When we used the term underrepresented, we were referring to the lack of published genomes for cephalopods. This has been slowly changing since the publication of the first cephalopod genome in 2015 by Albertin et al. The sentence has been modified to clarify this.

Original sentence:
“The recent publication of octopod genomes provides an opportunity to investigate the link between genomic and organismal evolution in this unique and underrepresented lineage”

Revised sentence: (pg. 3 ,lines: 52-55).
“The recent publication of octopod genomes provides an opportunity to investigate the link between genomic and organismal evolution in this unique lineage for which genomic resources have been lacking.1”

Pg. 3: (FAO,) - Looks like a typo? Or incomplete switch to a different citation format?

Corrected (pg. 3 ,line: 57)

Pg. 3: "soft bodied" should be "soft-bodied"

Corrected (pg. 3 ,line: 58)

Pg. 3: Should be "that are well adapted" (no hyphen)

Corrected (pg. 3 ,line: 58)

Pg. 4: "How resistance to TTX" - This is not totally clear as written. Resistance in what? I think the authors are referring to how Hapalochlaena avoids being killed by its own TTX, but this could be rephrased to make it crystal clear. Also "remains a large unknown" is a little awkward…maybe "remains largely unknown" would be better?

We agree that this sentence was unclear. Reviewer 3 also suggested that due the recent publication by Geffeney et al (2019) this sentence was no longer reflective of the current literature. In order to correct this we have updated this sentence to reflect the latest literature and taken care to ensure the sentences are clear.

Original sentence:
“How resistance to TTX has been acquired at the genetic level, remains a large unknown, with TTX resistance studied in only in a few select species (i.e. pufferfish13, newts14,15 and gastropods16).”

Powered by Editorial Manager® and ProduXion Manager® from Aries Systems Corporation

Revised sentences (pg. 4, lines: 74-79):
“The mechanism of TTX resistance, which allows for safe sequestration of TTX, has been attributed to several substitutions in the p-loop regions of voltage-gated sodium channels(Nav) in H. lunulata24. However, these channels have yet to be examined in H. maculosa and H. fasciata. TTX resistance has also been studied in a range of other genera including, pufferfish25, newts26,27 arachnids28, snakes29 and gastropods30”

Pg. 4: "Primarily used for defense…" - Awkward sentence with a dangling modifier, which makes it read as though Hapalochlaena is primarily used for defense in other species.

We agree that this was a poor choice of wording and this sentence has been clarified as follows:

Original sentence:
“Primarily used for defense in other unrelated TTX-bearing species, Hapalochlaena is the only known taxa to utilise TTX in venom”

Revised sentence (pg. 4-5,lines: 82-84):
“While other unrelated TTX-bearing species primarily use TTX for defense, Hapalochlaena is the only known taxa to utilise TTX in venom23,35.”

Pg. 5: "for example at the evolution of venoms" - Somewhat awkward, I think?

We agree and have modified the sentence to improve flow as follows:

Original sentence:
“By using a comparative genomic approach we are able to examine the emergence of octopod novelties, for example at the evolution of venoms, at a molecular level between H. maculosa and the two non-TTX bearing octopods: the California two-spot octopus (O. bimaculoides) and the long-armed octopus (Callistoctopus minor), while also addressing the species-specific evolution of tetrodotoxin acquisition and resistance in H. maculosa”

Revised sentence (pg. 5 ,lines: 93-98):
“By using a comparative genomic approach we are able to examine the emergence of octopod novelties, at a molecular level between H. maculosa and the two non-TTX bearing octopods: the California two-spot octopus (O. bimaculoides) and the long-armed octopus (Callistoctopus minor). We also address unique features of venom evolution in octopods while also addressing the species-specific evolution of tetrodotoxin acquisition and resistance in H. maculosa”

Pg. 6: Capitalize "bay"? (Port Phillip Bay)

Corrected (pg. 6, line: 108)

Pg. 7: Should be "shallow-water marine organisms".

Corrected (pg. 8, lines: 139)

Pg. 8: Should be "Southern Hemisphere".

Corrected (pg. 8, line: 142-143)

Pg. 8: Also here, how did they do their divergence time estimation?

Tanner et al. (2017) used a Bayesian approach to estimate divergence times, analyzing a concatenated alignment of 197 genes with Phylobayes. These details have been incorporated into the manuscript.

Original sentence:
“Previous phylogenies using a combination of a small number of mitochondrial and

nuclear genes46-48 and orthologs derived from transcriptomes49 support this topology. Likewise, divergence of the H. maculosa from Abdopus aculeatus has been previously estimated to be ~59 mya2”

Modified sentence (pg. 9, lines: 151-155) :
“Previous phylogenies using a combination of a small number of mitochondrial and nuclear genes49–51 and orthologs derived from transcriptomes52 support this topology. Likewise, estimates by Tanner et al.2, using a concatenated alignment of 197 genes with a Bayesian approach, placed divergence of H. maculosa from Abdopus aculeatus at ~59 mya2.”

Pg. 8: "maculosa from Abdopus" - "from" should not be italicized.

Corrected (pg. 9, line: 154)

Pg. 9: "sporadic occurrence" - I think the authors mean sporadic taxonomically here (i.e., some species have them, some do not), but this should be clarified (surely the authors don't mean that sometimes a given species has them and sometimes they don't!).

In order to prevent confusion the sentence has been modified to include the term “taxonomically sporadic”.

Original sentence
“Morphological variations of ocelli structure and colour, along with their sporadic occurrence within Octopus and Amphioctopus3, limits our interpretation as to the evolutionary history of this trait in octopods”

Revised sentence: (pg. 9, lines: 160-163):

“Morphological variations of ocelli structure and colour, in conjunction with the taxonomically sporadic occurrence of this trait across species within Octopus and Amphioctopus, limits our interpretation as to the evolutionary history of this trait in octopods.”

Pg. 11: Just a suggestion here: "splicing, embryonic and neural development" is clear, but it looks odd. How about "splicing and embryonic and neural development" or "splicing as well as embryonic and neural development".

We have modified the sentence as suggested:

Original sentence:
“H. maculosa also shows expansions in the C2H2-type zinc finger family. Zinc fingers form an ancient family of transcription factors, which among other roles serve to regulate transposon splicing, embryonic and neural development 45,46.”

Revised sentence: (pg. 12 ,lines: 207-09)
“H. maculosa also shows expansions in the C2H2-type zinc finger family. Zinc fingers form an ancient family of transcription factors, which among other roles serve to regulate transposon splicing as well as embryonic and neural development69,70.”

Pg. 11: Should be "this type of zinc finger in O. bimaculoides"

Corrected (pg. 12, line: 210)

Pg. 12: "High level examination" should be "High-level examination", "large scale expression patterns" should be "large-scale expression patterns", and "lineage specific loss" should be "lineage-specific loss".

Corrected (pg. 13, lines: 227 & 230)

Pg. 12: Unnecessary comma in "we also find that, genes specific to each octopod"

Corrected (pg.13, line: 234)

Powered by Editorial Manager® and ProduXion Manager® from Aries Systems Corporation

Pg. 13: "in tandem with overall reduction in genes number relative to the octopods" - This is unclear and poorly worded. I assume this is referring still to H. maculosa relative to other octopods?

In order to improve the clarity and wording of this sentence it has been rephrased as follows:

Original sentence:
“In order to understand the implications of gene expression loss, in tandem with overall reduction in genes number relative to the octopods, further investigation is required.”

Revised sentence: (pg. 14, lines: 249-251)
“In order to fully understand the implications of the gene family contractions and loss of expression in H. maculosa, relative to other octopods, further investigation is required.”

Pg. 15: Unnecessary comma in "More notable, were differences"

Corrected (pg. 17, line: 288)

Pg. 16: Should be "primary venom-producing gland".

Corrected (pg. 17, line: 296)

Pg. 16: Haplochlaena should be italicized in "hypothesized that the Hapalochlaena PSF…" Also, who has hypothesized that the Hapalochlaena PSF will exhibit a loss of redundant proteinaceous toxins? This seems to call for a citation.

This statement was poorly worded and has been corrected to reflect that we proposed the hypothesis. Hapalochlaena has also been italicized.

Original sentence:
“It has been hypothesized that the Hapalochlaena PSG will exhibit a loss of redundant proteinaceous toxins due to the presence of TTX.”

Revised sentence: (pg. 17, lines: 299-300)
“We hypothesize that the Hapalochlaena PSG will exhibit a loss of redundant proteinaceous toxins due to the presence of TTX.”

Pg. 16: "A total of 623 genes were exclusive to H. maculosa PSF…exclusive to O. bimaculoides and C. minor, respectively". Should this be "exclusive to the O. bimaculoides and C. minor PSGs, respectively"?

Yes the reviewer is correct. This sentence has been corrected as suggested.

Original sentence:
“A total of 623 genes were exclusive to H. maculosa PSG compared to only 230 and 164 exclusive to O. bimaculoides and C. minor, respectively”

Revised sentence (pg.17-18, lines 302-304)
“A total of 623 genes were exclusive to H. maculosa PSG compared to only 230 and 164 exclusive to O. bimaculoides and C. minor PSGs, respectively.”

Pg. 16: "Additionally, H. maculosa PSG is predicted to be" - Predicted by whom? The authors? I think so, so they should make that clear, e.g., "we predict that the H. maculosa PSG is functionally more diverse…"

This sentence has been corrected as suggested:

Revised sentence (pg. 18, lines: 304-306)
“Additionally, we predict that the H. maculosa PSG is functionally more diverse based on the number of Pfam families detected, 532 in total.”

Pg. 17: The sentence about reprolysin doesn't make sense to me. The authors

	describe shifting expression in this species (see my comment above) but then note that there is a complete loss<br>of orthologs from the genome. This latter comment suggests that reprolysin doesn't even exist in the H. maculosa genome, but it must, if it is showing different expression patterns than the other species. Can the authors clarify this?<br><br>The sentence that “there is a complete loss of orthologs from the genome” was incorrect. It should have read “there is a complete loss of paralogs from the genome”. This has been corrected in the sentence below.<br><br>Revised sentence (pg. 18, lines: 316-318)<br>“Similarly, reprolysin (M12B) exhibits shifting expression in H. maculosa, presumably from the PSG to the branchial heart, and a complete loss of paralogs from the genome.”<br><br>Pg. 18: "the cephalopod specific clade" should be "cephalopod-specific clade".<br><br>Corrected (pg. 19 ,line: 324)<br><br>Pg. 18: "…hyaluronidase, which often serve as dispersal factors" seems odd. Should this be "hyaluronidase, which often serves as a dispersal factor"?<br><br>Yes and we have modified the sentence as suggested.<br><br>Original sentence:<br>“Previous proteomic analysis of the H. maculosa PSG revealed high expression of hyaluronidase, which often serve as dispersal factors within snake venom, facilitating the spread of toxin while not being directly toxic to their prey9,72”<br><br>Revised sentence: (pg. 19, lines: 330-333)<br>“Previous proteomic analysis of the H. maculosa PSG revealed high expression of hyaluronidase, which often serves as a dispersal factor within snake venom, facilitating the spread of toxin while not being directly toxic to their prey9,72.”<br><br>Pg. 19: "Two Nav genes"…should the "v" be a subscript here?<br><br>Corrected (pg. 20, line: 352)<br><br>Pg. 19: "latter regions in DIII and DIV" - Is "latter" the best adjective here?<br><br>“Latter” is not an ideal word choice and the sentence has been modified.<br><br>Original sentence:<br>“The latter regions in DIII and DIV”<br><br>Revised sentence: (pg. 21, line: 356)<br>“The regions DIII and DIV closer to the C-terminal end of the protein”<br><br>Pg. 20: "In previous studies, when examined individually, the Met- Thr substitution in a TTX sensitive Nav1.4 rat channel decreased binding affinity in pufferfish by 15-fold" - This could be more clear. A "rat channel"? As in, a channel in rats? But the sentence says "in pufferfish". Please clarify this.<br><br>Jost et al 2008 found a Met-Thr substitution in the third p-loop region of a pufferfish sodium channel. This substitution was induced in a TTX sensitive rat channel through site-directed mutagenesis and the rat channel was then expressed in the oocytes of the African clawed frog (Xenopus). In our original sentence some of these taxonomical/methodological details obscured the ultimate finding which is the introduction of a Met-Thr substitution, in an otherwise TTX-sensitive channel, inhibits TTX binding by 15-fold. Our revised sentence has removed reference to taxonomy to convey the main finding more clearly.<br><br>Original sentence:<br>"In previous studies, when examined individually, the Met- Thr substitution in a TTX

sensitive Nav1.4 rat channel decreased binding affinity in pufferfish by 15-fold"

Revised sentence (pg. 21,lines: 365-366):
“In a previous study a Met to Thr substitution into a TTX sensitive Nav1.4 channel decreased binding affinity to TTX by 15-fold87.”

Pg. 21: "It has yet to be established if these mutations are derived from a shared ancestor or have occurred independently" - Excellent. This is exactly the clarity I think the authors can bring to their other statements about gains, losses, etc. that I pointed out in a previous comment.

As detailed in the first response to reviewers we have clarified these statements throughout.

Pg. 21: I would write "While Hapalochlaena remains" here.

Corrected (pg. 22 ,line: 379)

Pg. 21: Should be "STX-contaminated bivalves" (and "STX-contaminated fish" and "STX-contaminated food sources" below…and "TTX-producing bacteria" and "TTX-producing strains" on pg. 23).

Corrected (pg. 22-24, lines: 383, 391-392, 394, 416, 422)

Pg. 21: "Humboldt" should be capitalized, as it is a proper name.

Corrected (pg. 18, line: 389, 391)

Pg. 23: "Sequestration of TTX is not exclusive to the blue-ringed octopus among molluscs. Gastropods such as Pleurobranchaea maculata and Niotha clathrata, as well as some bivalves, are capable of sequestering the similar toxin STX " - TTX and STX are similar, but not the same. Are there other examples of actual TTX sequestration in molluscs, outside of Hapalochlaena? If not, this should be rephrased.

Yes, sequestration of TTX does occur in molluscs aside from Hapalochlaena, including but not necessarily limited to the gastropods Pleurobranchaea maculata and Niotha clathrata. In the original sentence we mistakenly said these species were capable of sequestering STX as opposed to TTX. This has been corrected in the revised sentence.

Original sentence:
“Gastropods such as Pleurobranchaea maculata and Niotha clathrata, as well as some bivalves, are capable of sequestering the similar toxin STX”

Revised sentence: (pg. 24 ,lines: 409-420)
“Gastropods such as Pleurobranchaea maculata and Niotha clathrata, as well as some bivalves, are also capable of sequestering TTX”

Pg. 23: "highly diverse composition of genera" may be better as "highly diverse composition of bacterial genera"

Corrected (pg. 24, lines: 413)

Pg. 23: "Diversity of bacterial genera much like the H. maculosa in this study was high" - This seems awkward and unclear."

This sentence was unclear and has been modified to:

Original sentence:
“Diversity of bacterial genera much like the H. maculosa in this study was high and this may complicate identification of species responsible for TTX production”

Revised sentence (pg. 25 , lines: 423-425):
“Congruent with our findings the diversity of bacterial genera was high and this may

	complicate identification of species responsible for TTX production"<br><br>Pg. 24: "TTX bearing mollusk genome" - Hmm…does the genome bear TTX? The mollusk does, I suppose, but it's produced by bacteria as described above. This could be rephrased.<br><br>The sentence has been rephrased to improve clarity to:<br><br>Original sentence:<br>"This work describes the genome of a unique TTX bearing mollusc genome, the southern blue-ringed octopus (Hapalochlaena maculosa)."<br><br>Revised sentence (pg. 25 ,lines: 434-435):<br>"This work describes the genome of a unique TTX bearing mollusc, the southern blue-ringed octopus (Hapalochlaena maculosa)."<br><br>Pg. 28: What kit or method was used to construct the cDNA libraries for transcriptome sequencing?<br><br>Construction of cDNA libraries was outsourced to AGRF (Australian Genome Research Facility), Melbourne, and conducted using their TruSeq mRNA Library Prep with polyA selection and unique dual indexing method. This information has been included in the methods section "Transcriptome sequencing"<br><br>Revised text: (pg. 29, lines: 499-504)<br>"Construction of cDNA libraries was outsourced to AGRF (Australian Genome Research Facility), Melbourne and conducted using their TruSeq mRNA Library Prep with polyA selection and unique dual indexing method. Libraries were constructed using 3 µg of RNA at a concentration of >100 ng/µ L. Each tissue was sequenced on 1/12th of an Illumina HiSeq2000 lane with one lane used in total."<br><br>Pg. 30: Which assemblies of the molluscan genomes (Crassostrea, etc.) were used? Where did the transcriptomes for Sepia and Idiosepius come from? Were reads downloaded from the NCBI SRA and assembled in Trinity? If so, what were the BioProejct numbers for the transcriptome data?<br><br>We have modified the methods section "Calibration of sequence divergence with respect to time" to include details as to the origin of both the transcriptomes and the genomes used in this study.<br><br>Revised text: (pg. 31-32, lines: 539-546)<br>Bioprojects for each genome used are as follows:Crassostera gigas (PRJNA629593 & PRJEB3535), Lottia gigantea (PRJNA259762 & PRJNA175706), Aplysia californica (PRJNA629593 & PRJNA13635) and (Euprymna scolopes PRJNA47095). Octopus bimaculoides was obtained from http://octopus.unit.oist.jp/OCTDATA/BASIC/Metazome/Obimaculoides_280.fa.gz. The , Idiosepius notoides (BioProject: PRJNA302677) transcriptome was sequenced and assembled using the same method previously described for the H. maculosa transcriptome.<br><br>Pg. 32: I think when the authors write "H. maculosa is a single generation species", they mean that it is semelparous, but I don't see why that is relevant for mutation rate calculations.<br><br>We completely agree that this has little impact on mutation rate (measured per generation). This was included in an early draft (that discussed the coalescent demographic analyses in more detail) and was kept in by mistake. The text has been revised to remove "H. maculosa is a single generation species"<br><br>Original sentence:<br>"Per base neutral substitution between lineages was determined using the mean dS value divided by divergence time (refer to Calibration of sequence divergence with respect to time) usually over number of generations, however H. maculosa is a single generation species"

Revised sentence: (pg. 34, lines: 575-577)
“Per base neutral substitution between lineages was determined using the mean dS value divided by divergence time (refer to Calibration of sequence divergence with respect to time) over the number of generations.”

Pg. 33: "genes with expression within one or more tissues was determined" should be "…were determined".

Corrected (pg. 35, line: 592).

Pg. 34: Something is strange in this sentence - "A loss of expression requires a gene to be present in all three octopods with and expressed in one or more species while having no detectable expression in at least one species" (an extra "with"?)

Corrected (pg. 35, lines: 602-604).

Pg. 34: Individual mutation with potential" should be "mutations"

Corrected (pg. 36, line: 613-614).

Figure 5: Loligo pealei and Doryteuthis pealeii are the same species. The latter name is the correct one.

Figure 5 has been corrected by removing the duplicate sequence and retaining the correct name Doryteuthis pealeii

Low quality version shown here. The corrected version has been uploaded to replace the original

Supplementary Material

4.1: a)What models and settings were used in RAxML and PhyloBayes?
b)How were those models chosen?
c)What calibrations were used for the divergence time analysis?
d)How was convergence inferred for the PhyloBayes run?
e)Also, Supplementary Figure 3 is a a "QITREE" tree…do the authors mean IQ-TREE? If so, why is IQ-TREE not mentioned in the text? This tree also differs slightly from the tree presented in Figure 1a, though I doubt the difference is of any consequence for this paper.

In order to clearly address the points raised by Reviewer1, we have answered each question separately.

a)The models chosen for RaxML and Phylobayes were GTR+G+I and strict clock with a mixture model of F81 + G respectively.
b) These models were selected based on results from JmodelTest.
c) Calibrations were used on two nodes for the Phylobayes run : divergence between H. maculosa and E. scolopes 275mya & divergence between C. gigas and E. scolopes 500mya
d) Convergence was ascertained using tracecomp from the Phylobayes package
e) Two trees were run using the same alignment, the first was run with RAXML and the resulting tree used to inform Phylobayes, the second was run with IQTREE and included as a supplementary as it does not differ from the previous tree in any significant capacity. We have corrected the typo QITREE in the text.

We have added the details mentioned above to the supplementary materials section “4.1 Multi-gene cephalopod phylogeny and dating”.

Original text:
“A total of 2,108 clusters were obtained. Phylogenies were constructed using RAxML31 and Phylobayes32. Divergence times were calculated using Phylobayes, calibrations, setting and model used”

Revised text: (pg. 15-16, lines: 222-232)
“A total of 2,108 clusters were obtained. Phylogenies were constructed using RAxML v8.031 and divergence times estimated by Phylobayes v4.132. RAxML v8.031 was run using the GTR+G+I model ascertained from JmodelTest v2.1.10. using the cAIC criterion for 100 bootstraps. Phylobayes estimated divergence times under a strict clock with a mixture model of F81+G with a burn-in of 10%. Calibrations were used as follows : divergence between H. maculosa and E. scolopes 275mya & divergence between C. gigas and E. scolopes 500mya. Two runs were performed and convergence verified using bpcomp, which confirmed a maximum difference of < 0.1 and tracecomp, which also indicated convergence with an effective sample size(EES) of > 200 for all parameters. Both programs used were from the Phylobayes package.”

Reviewer #2: Review
Manuscript Number: GIGA-D-20-00135
Title: Adaptive venom evolution and toxicity in octopods is driven by extensive novel gene formation, expansion and loss
submitted to: GigaScience

This manuscript presents interesting data sets of both the genomic sequence of the TTX-bearing octopus Hapalochlaena maculosa as well as transcriptomes from twelve different tissues. The methods used were appropriate for the aims of the study including the use of two different methods to prepare (Illumina and Chicago), sequence (Illumina HiSeq 2000 and Dovetail) and assemble the genome (Illumina and HiRise). The authors do a good job reporting the statistical analysis of their assembly and comparing their statistics to two other octopus genomes, Callistoctopus minor and Octopus bimaculoides. Their methods of transcriptome sequencing, analysis and assembly were appropriate. Finally, their analysis of the completeness of their genome was appropriate and indicate that their genome is well constructed.
Their further analysis of the assembled genome and transcriptome are interesting and appropriate including the examination of the expansion of the zinc finger and cadherin/protocadherin gene families that have previously been identified in octopuses. Their analysis of expression differences in genes expressed in the posterior salivary gland between non-TTX bearing octopuses and the TTX-bearing H. maculosa is informative and suggests that the expression of serine protease venoms found in non TTX-bearing octopuses is reduced in H. maculosa. Finally, the authors confirm that H. maculosa has the same set of amino acid substitutions that are found in the voltage-gated sodium channel NaV1 of Hapalochlaena lunalata. In both species, these changes in channel structure are likely to impart TTX resistance and explain the genetic mechanism underlying TTX resistance in the genus.
The authors appear to have met the minimum standard of reporting for the journal. However, the authors have not done an adequate job of reviewing the scientific literature that would contextualize their work and this has led to inaccurate statements in the manuscript. The manuscript requires editing for clarity. I will highlight several of the problem sections below.

1) In the abstract/background the authors state "Cephalopods represent a rich system for investigating the genetic basis underlying organismal novelties. This diverse group of specilised predators have evolved many unique adaptations including proteinaceous venom." Proteinaceous venoms are not unique to cephalopods. Snakes have evolved the use of proteinaceous venoms that function as enzymes including serine proteases that the authors suggest are unique to cephalopods. For an example, see a review in Toxicon from 2013 by Solange and Serrano. The authors could strengthen this manuscript by discussing their work in the context of the independent evolution in vertebrates and invertebrate lineages of the use of this enzyme class. The author's interesting report that serine protease expression is reduced in a tetrodotoxin (TTX) bearing cephalopod compared to non-TTX bearing cephalopods is overshadowed by this mischaracterization of the uniqueness of this character in cephalopods.

It was not our intention to convey that cephalopods are the only taxa to have evolved proteinaceous venom and to rectify this we have modified the sentence in the abstract to more accurately represent the literature. Additionally, we clarified the independent evolution between invertebrates and vertebrates in the introduction.

Powered by Editorial Manager® and ProduXion Manager® from Aries Systems Corporation

Abstract:
Original text:
"Cephalopods represent a rich system for investigating the genetic basis underlying organismal novelties. This diverse group of specilised predators have evolved many unique adaptations including proteinaceous venom."

Revised text: (pg.1, lines:23-25)
“Cephalopods represent a rich system for investigating the genetic basis underlying organismal novelties. This diverse group of specialized predators has evolved many adaptations including proteinaceous venom.”

Background:
Original text:
“Furthermore, proteinaceous venom is produced and stored within a specialised gland in cephalopods known as the posterior salivary gland (PSG)”

Revised text: (pg.3-4, lines:63-71)
“Furthermore, the cephalopods have independently evolved proteinaceous venom, which is produced and stored within a specialised gland in known as the posterior salivary gland (PSG). All octopods are believed to possess a form of proteinaceous venom used to subdue prey8–10. Serine proteases are a common component of cephalopod venoms and have been observed in the PSG of squids, cuttlefish and octopods10–13. Convergent recruitment of serine proteases has been observed between many vertebrate (Squamata14–16 and Monotremata17) and invertebrate (Hymenoptera18, Arachnida19, Gastropoda20, Remipedia21 and Cnidarian22) venomous lineages.”

2)In the abstract/findings description the authors state "…voltage-gated sodium channels in H. maculosa contain a resistance mutation found in pufferfish and garter snakes, which is absent in other octopods." Hapalochlaena maculosa has the same amino acid sequences encoded in the voltage-gated sodium channel genes NaV1 and NaV2 as previously reported for the Greater Blue-ringed octopus Hapalochlaena lunulata, in Toxicon from 2019 by Geffeney and colleagues.

This sentence has been corrected to reflect the recent finding of the resistance mutations by Gefferny et al 2019 in H. lunulata.

Original sentence:
“Moreover, voltage-gated sodium channels in H. maculosa contain a resistance mutation found in pufferfish and garter snakes, which is absent in other octopods”

Modified sentence: (pg.2, lines:38-40)
“Moreover, voltage-gated sodium channels in H. maculosa contain a resistance mutation found in pufferfish and garter snakes, which is exclusive to the genus.”

3) In the background section of the main body the authors state "How resistance to TTX has been acquired at the genetic level, remains a large unknown, with TTX resistance studied in only in a few select species (i.e. pufferfish13, newts14,15 and gastropods16)." The changes in voltage-gated sodium channel genes that lead to TTX-resistance are well understood. Genetic changes that lead to TTX resistance have been examined in groups not included in the authors list including other invertebrates (e.g. insects and blue-ringed octopuses) as well as snakes. The authors statement mischaracterizes the body of literature examining the evolution of TTX resistance.

This section has been modified to more accurately depict the current literature by providing more examples of taxa which exhibit resistance to TTX. We now included an example of an arachnid whose channels have been examined for TTX resistance, however we were unable to find an example of an insect as suggested above by reviewer 2. We would be happy to include an example of an insect if the reference could be provided.

Original text:

Powered by Editorial Manager® and ProduXion Manager® from Aries Systems Corporation

"How resistance to TTX has been acquired at the genetic level, remains a large unknown, with TTX resistance studied in only in a few select species (i.e. pufferfish13, newts14,15 and gastropods16)."

Revised text: (pg. 4, lines: 74-89)
“The mechanism of TTX resistance, which allows for safe sequestration of TTX, has been attributed to several substitutions in the p-loop regions of voltage-gated sodium channels(Nav) in H. lunulata24. However, these channels have yet to be examined in H. maculosa and H. fasciata. TTX resistance has also been studied in a range of other genera including, pufferfish25, newts26,27 arachnids28, snakes29 and gastropods30.”

4)In the data description the authors discuss their work to identify the expansion of genes in the cadherin/protocadherin gene family. This section requires citations as well as correction of existing citations.

a.The authors state "H. maculosa and C. minor exhibit expansions in the cadherin gene family, characteristic of other octopod genomes, including O. bimaculoides (Fig1b)." without including a reference. This statement requires a reference and the discussion of their data would be improved by comparing their findings to other articles that have examined the expansion of the cadherin gene family and specifically protocadherins, for example Styfhals et al. (2019) in Frontiers in Physiology.

We agree with the reviewer that further discussion was required for this section and have restructured the paragraph to the following:

Original text:
“Organismal impact of novel genes and gene family expansions
Gene family expansions between octopods (O. bimaculoides, C. minor and H. maculosa) and three other molluscan genomes (Aplysia californica, Lottia gigantea and Crassostrea gigas) were examined using Pfam annotations. A total of 5565 Pfam domains were identified among six molluscan genomes. H. maculosa and C. minor exhibit expansions in the cadherin gene family, characteristic of other octopod genomes, including O. bimaculoides (Fig1b). C. minor, in particular, shows the greatest expansion of this family within octopods. Expansions of protocadherins, a subset of the cadherin family, have also occurred independently in squid 20, with the octopod expansions occuring post divergence ~135 mya 20. Cadherins, specifically protocadherins, are essential mediators of short-range neuronal connections in mammals42 43. Due to the absence of a myelin sheath in octopods, short-range connections are integral to maintaining signal fidelity over distance44.”

Revised text: (pg. 10-12, lines: 183-206)
“Organismal impact of novel genes and gene family expansions
Gene family expansions between octopods (O. bimaculoides, C. minor and H. maculosa) and three other molluscan genomes (Aplysia californica, Lottia gigantea and Crassostrea gigas) were examined using Pfam annotations. A total of 5565 Pfam domains were identified among six molluscan genomes. H. maculosa and C. minor exhibit expansions in the cadherin gene family, characteristic of other octopod genomes, including O. bimaculoides (Fig1b)42,64. C. minor, in particular, shows the greatest expansion of this family within octopods. Expansions of protocadherins, a subset of the cadherin family, have also occurred independently in squid42, with the octopod expansions occurring post divergence ~135 mya42. The shared ancestry of octopod cadherins was also documented by Styfhals et al64 using phylogenetic inference between O. bimaculoides and O. vulgaris.Cadherins, specifically protocadherins, play crucial roles in synapse formation, elimination and axon targeting within mammals and are essential mediators of short-range neuronal connections65–68. It should be noted that octopods lack a myelin sheath, as a result short-range connections are integral to maintaining signal fidelity over distance6. The independent expansions of protocadherins within chordate and cephalopod lineages are believed to be associated with increased neuronal complexity42,64. Elevated expression of protocadherins within neural tissues have been observed in O. vulgaris and O. bimaculoides by both Styfhals et al64 and Albertin et al42 respectively. In particular Styfhals et al64 noted differential expression across neural tissues including supra-esophageal mass, sub-esophageal mass, optic lobe and the stellate ganglion64.

Powered by Editorial Manager® and ProduXion Manager® from Aries Systems Corporation

	However, functional implications of observed expression patterns remain speculative without further study."<br><br>b.The next sentence ("Expansions of protocadherins, a subset of the cadherin family, have also occurred independently in squid 20, with the octopod expansions occuring post divergence ~135 mya 20.") incorrectly references Williams et al. (2012, reference 20) but should reference Albertin et al. (2015, reference 29).<br><br>This reference was corrected as suggested: (pg. 11, line:190-192)<br><br>"Expansions of protocadherins, a subset of the cadherin family, have also occurred independently in squid42, with the octopod expansions occurring post divergence ~135 mya42"<br><br>c.The authors state "Cadherins, specifically protocadherins, are essential mediators of short-range neuronal connections in mammals42 43. Due to the absence of a myelin sheath in octopods, short-range connections are integral to maintaining signal fidelity over distance44." None of the citations in these two sentences are correct and no correct references can be found in the list of citations. These final statements should include the fact that expansion in the number of protocadherin genes also occurs in chordates (for example, Hulpiau & van Roy, 2010 from Molecular Biology and Evolution). There is good evidence that protocadherins have roles in multiple aspects of proper synapse formation in mammals including synapse generation, synapse elimination and axon targeting (for example see reviews by de Wit and Ghosh from 2016 in Nature Reviews Neuroscience as well as Peek et al. from 2017 in Cellular and Molecular Life Sciences). Though synapses are "short-range connections", proper synapse formation is important for vertebrates and invertebrates with complex nervous systems whether or not that have myelinated axons. The expansion of protocadherin genes in both cephalopods and chordates independently is thought to be linked to increased neuronal circuit complexity.<br><br>We have corrected the miscitations and restructured the paragraph to incorporate the reviewers suggestions and more accurately describe the evolution and role of protocadherins in vertebrates and invertebrates.<br><br>Original text:<br>"Cadherins, specifically protocadherins, are essential mediators of short-range neuronal connections in mammals42 43. Due to the absence of a myelin sheath in octopods, short-range connections are integral to maintaining signal fidelity over distance44."<br><br>Revised text: (pg. 11-12, lines: 194-206)<br>"Cadherins, specifically protocadherins, play crucial roles in synapse formation, elimination and axon targeting within mammals and are essential mediators of short-range neuronal connections65–68. It should be noted that octopods lack a myelin sheath, as a result short-range connections are integral to maintaining signal fidelity over distance6. The independent expansions of protocadherins within chordate and cephalopod lineages are believed to be associated with increased neuronal complexity42,64. Elevated expression of protocadherins within neural tissues have been observed in O. vulgaris and O. bimaculoides by both Styfhals et al64 and Albertin et al42 respectively. In particular Styfhals et al64 noted differential expression across neural tissues including supra-esophageal mass, sub-esophageal mass, optic lobe and the stellate ganglion64. However, functional implications of observed expression patterns remain speculative without further study."<br><br>5)In the data description the authors discuss their work to identify the expansion of genes in the zinc finger gene family. The author state "H. maculosa also shows expansions in the C2H2-type zinc finger family. Zinc fingers form an ancient family of transcription factors, which among other roles serve to regulate transposon splicing, embryonic and neural development 45,46." These references are not correct for this statement. The manuscript would be strengthened by proper citations in this section, for example Fedotova and colleagues (2017) have a review in Acta Naturae. Additionally, there is evidence that these proteins have roles in both transposon suppression and alternative splicing.

Citations were corrected as suggested: (pg. 12, lines: 207-209)

“H. maculosa also shows expansions in the C2H2-type zinc finger family. Zinc fingers form an ancient family of transcription factors, which among other roles serve to regulate transposon splicing as well as embryonic and neural development69,70.”

6)The authors state "It has been hypothesized that the Hapalochlaena PSG will exhibit a loss of redundant proteinaceous toxins due to the presence of TTX." This sentence should have a citation or the authors should explain that this statement is their hypothesis.

This statement is a hypothesis by the authors and the sentence has been modified to reflect this.

Original sentence:
“It has been hypothesized that the Hapalochlaena PSG will exhibit a loss of redundant proteinaceous toxins due to the presence of TTX.”

Revised sentence: (pg.17, lines: 299-300)
“We hypothesize that the Hapalochlaena PSG will exhibit a loss of redundant proteinaceous toxins due to the presence of TTX.”

7)There are minor errors in the sequences presented in Figure 5. In multiple invertebrate species, phenylalanine (F) replaces tyrosine (Y) in the D1 pore. In pufferfish cysteine (C) replaces tyrosine (Y). The figure is constructed in a way that suggests that these amino acids replace a neighboring aspartic acid (D).

Figure 5 has been modified so all sequences align correctly.
Low quality version shown here. The corrected version has been uploaded to replace the original

Reviewer #3
A truly excellent paper that was a pleasure to read. My comments are very minor:
- TTX resistance in Thamnophis species of snakes should be referenced in the sentence "How resistance to TTX has been acquired at the genetic level, remains a large unknown, with TTX resistance studied in only in a few select species (i.e. pufferfish13, newts14,15 and gastropods16)". This is cited later (ref 58) but it would be appropriate for inclusion in this sentence too.
This citation has been added as suggested to a modified version of this sentence as suggested by reviewer 1:

Original text:
"How resistance to TTX has been acquired at the genetic level, remains a large unknown, with TTX resistance studied in only in a few select species (i.e. pufferfish13, newts14,15 and gastropods16)"
Revised text: (pg. 4, lines: 74-79)
“The mechanism of TTX resistance, which allows for safe sequestration of TTX, has been attributed to several substitutions in the p-loop regions of voltage-gated sodium channels(Nav) in H. lunulata13. However, these channels have yet to be examined in H. maculosa and H. fasciata. TTX resistance has also been studied in a range of other genera including, pufferfish14, newts15,16 arachnids17, snakes18 and gastropods19.

- For the PSG specific genes, calculations of the relative rates of evolution would be informative as this would be suggestive of adaptive evolution eg are the abundant serine proteases C. minor showing signs of accelerated evolution seen in other venomous lineages such as snakes? Previous work has shown that the sites on the molecular surface are undergoing episodic diversification when compared across a wide range of lineages. In this case, it would be interesting to see what the evolutionary patterns are for C. minor, in that is the extensive duplication accompanied by signs of diversification?
We investigated the potential of positive selection within serine proteases with a focus

	on genes specifically expressed in the posterior salivary gland (venom gland). Unfortunately, we did not find strong evidence of accelerated evolution in these genes with the method described below. In the future we look forward to conducting a more in-depth analysis of this interesting family with a more comprehensive sampling across coleoid cephalopods.<br>This section below, describing our additional analyses, has been added to the supplementary materials: (pg. 24-25, lines: 355-382)<br>6.4 Examination of selection and evolutionary rates in octopod serine proteases<br>Gene models (aa) from the three octopod genomes (H. maculosa, O. bimaculoides and C. minor) were annotated with Interproscan and serine proteases with the Pfam PF00089 extracted for examination. Gene models and their corresponding CDS sequences were imported into Geneious v10.2.6 and selected for a single trypsin (PF00089) domain greater than 200aa/600bp long. The region containing the trypsin domain was then extracted from the nucleic acid sequences and MAFFT v7.407 was used to align sequences using Translation align in Geneious v10.2.6, which interpreted the first codon as the start of the codon region and used the first translation frame. The resulting alignment was tested for an appropriate substitution model in jModelTest v2.2.10 and a tree was generated with RAxML v8.0 using the GTR +G+I model and 100 bootstraps. The resulting tree and alignment were examined using codeml via EasyCodeml v1.21 from the PAML package to examine non-synonymous to synonymous substitution rates for evidence of positive selection. We first used a site-based model which allows for ω values to vary between sites along the protein. Comparison of the nested models (M1a-M2a) and (M7-M8) did not reveal any sites under positive selection ($p > 0.05$). In order to access the potential for different rates of evolution within specific lineages we used a branch site model which allows for ω values to vary between sites and branches. For the foreground a large clade of genes, majority of which were specifically expressed in the posterior salivary gland (PSG) was selected and compared to all other non-PSG specific genes. No sites among the foreground branches were significantly accelerated relative to the background. The last method implemented is similar to the branch site model, however, the rate along sites is constant and the rate between the background and foreground can differ. This also found no evidence of positive selection between the background and foreground lineages. It should be noted that serine proteases are a large and complex family and are due a more in-depth analysis in coleoid cephalopods, which could form a complete stand-alone study.<br>- The M12B metalloprotease type in snake venom has a wide range of demonstrated activities, both anticoagulant (fibrinogenolytic) but also procoagulant (Factor X activating [Atractaspis and Daboia venoms] and prothrombin activating (Bothrops, Echis, and Dispholidus/Thelatornis venoms]) prothrombin activating metalloproteases from Dispholidus typus (boomslang) and Thelotornis mossambicanus (twig snake). " Comp Biochem Physiol C Toxicol Pharmacol: 108625. Oulion, B., J. S. Dobson, C. N. Zdenek, K. Arbuckle, C. Lister, F. C. P. Coimbra, B. Op den Brouw, J. Debono, A. Rogalski, A. Violette, R. Fourmy, N. Frank and B. G. Fry (2018). "Factor X activating Atractaspis snake venoms and the relative coagulotoxicity neutralising efficacy of African antivenoms." Toxicol Lett 288: 119-128.<br>Rogalski, A., C. Soerensen, B. Op den Brouw, C. Lister, D. Dashevsky, K. Arbuckle, A. Gloria, C. N. Zdenek, N. R. Casewell, J. M. Gutierrez, W. Wuster, S. A. Ali, P. Masci, P. Rowley, N. Frank and B. G. Fry (2017). "Differential procoagulant effects of saw-scaled viper (Serpentes: Viperidae: Echis) snake venoms on human plasma and the narrow taxonomic ranges of antivenom efficacies." Toxicol Lett 280: 159-170.<br>Sousa, L. F., C. N. Zdenek, J. S. Dobson, B. Op den Brouw, F. Coimbra, A. Gillett, T. H. M. Del-Rei, H. M. Chalkidis, S. Sant'Anna, M. M. Teixeira-da-Rocha, K. Grego, S. R. Travaglia Cardoso, A. M. Moura da Silva and B. G. Fry (2018). "Coagulotoxicity of Bothrops (Lancehead Pit-Vipers) Venoms from Brazil: Differential Biochemistry and Antivenom Efficacy Resulting from Prey-Driven Venom Variation." Toxins (Basel) 10(10): 411.<br>We agree that these references should be included in the manuscript and have added them as appropriate. (pg. 18, lines:316-319)<br><br>“Similarly, reprolysin (M12B) exhibits shifting expression in H. maculosa, presumably from the PSG to the branchial heart, and a complete loss of paralogs from the genome. While the function of this protein has not been assessed in octopus, members of this protein family exhibit anticoagulant properties in snake venom75–78.
**Additional Information:**	

Question	Response
Are you submitting this manuscript to a special series or article collection?	No
**Experimental design and statistics**<br><br>Full details of the experimental design and statistical methods used should be given in the Methods section, as detailed in our Minimum Standards Reporting Checklist. Information essential to interpreting the data presented should be made available in the figure legends.<br><br>Have you included all the information requested in your manuscript?	Yes
**Resources**<br><br>A description of all resources used, including antibodies, cell lines, animals and software tools, with enough information to allow them to be uniquely identified, should be included in the Methods section. Authors are strongly encouraged to cite Research Resource Identifiers (RRIDs) for antibodies, model organisms and tools, where possible.<br><br>Have you included the information requested as detailed in our Minimum Standards Reporting Checklist?	Yes
**Availability of data and materials**<br><br>All datasets and code on which the conclusions of the paper rely must be either included in your submission or deposited in publicly available repositories (where available and ethically appropriate), referencing such data using a unique identifier in the references and in the "Availability of Data and Materials" section of your manuscript.	Yes

Have you have met the above requirement as detailed in our Minimum Standards Reporting Checklist?	

Powered by Editorial Manager® and ProduXion Manager® from Aries Systems Corporation

Revised Manuscript

Click here to access/download;Manuscript;Bwhitelaw_Manuscript_5_8_2020J

Adaptive venom evolution and toxicity in octopods is driven by extensive novel gene formation, expansion and loss

Brooke L. Whitelaw[1,2], Ira R. Cooke[3,4], Julian Finn[2], Rute R. da Fonseca[5], Elena A. Ritschard[6], M. T P. Gilbert[7] Oleg Simakov[6], Jan M. Strugnell[1,8]

[1]Centre for Sustainable Tropical Fisheries and Aquaculture, James Cook University, Townsville, Queensland, 4811, Australia

[2]Sciences, Museum Victoria, Carlton, Victoria 3053, Australia

[3]College of Public Health, Medical and Vet Sciences, James Cook University, Townsville, Queensland, 4811, Australia

[4]La Trobe Institute of Molecular Science, La Trobe University, Melbourne, Victoria 3086, Australia

[5]Center for Macroecology, Evolution and Climate (CMEC), GLOBE Institute, University of Copenhagen, Universitetsparken 15, 2100 Copenhagen, Denmark;

[6]Department of Molecular Evolution and Development, University of Vienna, Universitätsring 1, 1010 Wien, Austria

[7]Center for Evolutionary Hologenomics, GLOBE Institute, University of Copenhagen, Øster Voldgade 5-7, 1350 Copenhagen, Denmark;

[8]Department of Ecology, Environment and Evolution, La Trobe University, Melbourne, Victoria, 3086 Australia

Abstract

Background

Cephalopods represent a rich system for investigating the genetic basis underlying organismal novelties. This diverse group of specialized predators has evolved many adaptations including proteinaceous venom. Of particular interest is the blue-ringed-octopus genus (*Hapalochlaena*), which are the only octopods known to store large quantities of the potent neurotoxin, tetrodotoxin, within their tissues and venom gland.

Findings

To reveal genomic correlates of organismal novelties, we conducted a comparative study of three octopod genomes, including the Southern blue-ringed octopus (*Hapalochlaena maculosa*). We present the genome of this species and reveal highly dynamic evolutionary patterns at both non-coding and coding organizational levels.

Gene family expansions previously reported in *Octopus bimaculoides* (e.g., zinc finger and cadherins, both associated with neural functions), as well as formation of novel gene families, dominate the genomic landscape in all octopods. Examination of tissue-specific genes in the posterior salivary gland (PSG) revealed that expression was dominated by serine proteases in non- tetrodotoxin bearing octopods, while this family was a minor component in *H. maculosa*. Moreover, voltage-gated sodium channels in *H. maculosa* contain a resistance mutation found in pufferfish and garter snakes, which is exclusive to the genus. Analysis of the PSG microbiome revealed a diverse array of bacterial species, including genera that can produce tetrodotoxin, suggestive of a possible production source.

Conclusions

We present the first tetrodotoxin-bearing octopod genome *H. maculosa,* which displays lineage-specific adaptations to tetrodotoxin acquisition. This genome, along with other recently published cephalopod genomes, represents a valuable resource from which future work could advance our understanding of the evolution of genomic novelty in this family.

Background

Reconstructing the evolution of novelties at the genomic level is becoming an increasingly viable approach to understand their origin. The recent publication of octopod genomes provides an opportunity to investigate the link between genomic and organismal evolution in this unique lineage for which genomic resources have been lacking[1]. From their emergence 275 mya[2], octopods have diversified into > 300 species, inhabiting tropical to polar regions, from the deep sea to shallow intertidal zones[3]. As a highly diverse group, octopods show remarkable variation in body form and function. They are specialised soft-bodied predators that are well adapted to their environment with prehensile limbs lined with chemosensory suckers[4], the ability to manipulate skin texture and colour using specialised chromatophores[5], the largest invertebrate nervous systems (excluding those of other cephalopods)[6], and a relatively large circumesophageal brain allowing for complex problem solving and retention of information[7]. Furthermore, the cephalopods have independently evolved proteinaceous venom, which is produced and stored within a specialised gland in known as the posterior salivary gland (PSG). All octopods are believed to possess a form of

proteinaceous venom used to subdue prey[8–10]. Serine proteases are a common component of cephalopod venoms and have been observed in the PSG of squids, cuttlefish and octopods[10–13]. Convergent recruitment of serine proteases has been observed between many vertebrate (Squamata[14–16] and Monotremata[17]) and invertebrate (Hymenoptera[18], Arachnida[19], Gastropoda[20], Remipedia[21] and Cnidarian[22]) venomous lineages.

In addition to these proteinaceous venoms, the blue-ringed octopus (genus *Hapalochlaena*) is the only group that also contains the potent non-proteinaceous neurotoxin, tetrodotoxin (TTX)[12,23]. The mechanism of TTX resistance, which allows for safe sequestration of TTX, has been attributed to several substitutions in the p-loop regions of voltage-gated sodium channels(Na_v) in *H. lunulata*[24]. However, these channels have yet to be examined in *H. maculosa* and *H. fasciata*. TTX resistance has also been studied in a range of other genera including, pufferfish[25], newts[26,27] arachnids[28], snakes[29] and gastropods[30].

The blue-ringed octopus is easily identified by iridescent blue rings, which advertise its toxicity in an aposematic display[31–33]. Sequestration of the TTX within bodily tissues is unique to this genus among cephalopods[32,34]. While other unrelated

TTX-bearing species primarily use TTX for defense, *Hapalochlaena* is the only known taxa to utilise TTX in venom[23,35]. The impact of TTX inclusion on venom composition and function has been previously investigated in the southern blue-ringed octopus (*H. maculosa*)[9]. Relative to the non-TTX bearing species *Octopus kaurna*, *H. maculosa* exhibited greater expression of putative dispersal factors such as hyaluronidase, which serve to aid in the dispersal of toxic venom components[9]. Conversely, tachykinins-neurotoxins known from other octopods[36,37] were absent from the *H. maculosa* PSG[9]. Further investigation into the broader impact of TTX on the evolutionary trajectory of the species has yet to be addressed due to the absence of a genome.

This study presents the genome of the southern blue-ringed octopus (*H. maculosa*), the first from the genus *Hapalochlaena.* By using a comparative genomic approach we are able to examine the emergence of octopod novelties, at a molecular level between *H. maculosa* and the two non-TTX bearing octopods: the California two-spot octopus (*O. bimaculoides*) and the long-armed octopus (*Callistoctopus minor*). We also address unique features of venom evolution in octopods while also addressing the species-specific evolution of tetrodotoxin acquisition and resistance in *H. maculosa*.

Keywords: cephalopod genome, comparative genomics, gene family expansions, transposable elements, venom evolution

Data Description

Genome assembly and annotation

The southern blue-ringed octopus genome was sequenced using Illumina paired end and Dovetail sequencing from a single female collected at Beaumaris Sea Scout Boat Shed, Beaumaris, Port Phillip Bay, Victoria, Australia. The assembly was composed of 48,285 scaffolds with an N50 of 0.93 Mb and total size of 4.08 GB. A total of 29,328 inferred protein coding genes were predicted using a PASA[38] and an Augustus[39] pipeline and supplemented with zinc finger and cadherin genes obtained from aligning *H. maculosa* transcripts to *O. bimaculoides* gene models(Supplementary notes 1.1-1.4). Completeness of the genome was estimated using BUSCO[40], which identified 87.7% complete and 7.5% fragmented genes against the metazoan database of 978 groups (Supplementary notes 3.2).

H. maculosa has a highly heterozygous genome (0.95%), similar to *O. vulgaris* (1.1%)[41] but far higher than *O. bimaculoides* (0.08%)[42]. While the low heterozygosity of *O. bimaculoides* is surprising, other molluscs also have highly heterozygous genomes in accordance with *H. maculosa*, including the gastropods (1-3.66%)[43,44] and bivalves (0.51-3%)[45–51](Supplementary table 5).

PSMC (Pairwise Sequentially Markovian Coalescent) and mutation rate

The mutation rate for *H. maculosa* was estimated to be 2.4 x 10^{-9} per site per generation based on analysis of synonymous differences with *O. bimaculoides* (Supplementary note 1.5). The mutation rate is comparable to the average mammalian mutation rate of 2.2 x 10^{-9} per site per generation, and *Drosophila*, 2.8 x 10^{-9}[52,53]. Due to the unavailability of a suitable closely related and comprehensive genome until the publication of *O. bimaculoides* in 2015[42], this is the first genome-wide mutation rate estimated for any cephalopod genome.

The historic effective population size (Ne) of *H. maculosa* was estimated using the pairwise sequentially Markovian coalescent (PSMC) model (Supplementary Fig 2). Population size was found to initially increase during the early Pleistocene, followed by

a steady decline which slows slightly around 100kya. Note that PSMC estimates are not reliable at very recent times due to a scarcity of genomic blocks that share a recent common ancestor in this highly heterozygous genome. A decline in population size started during the mid-Pleistocene approximately 1mya, a time of unstable environmental conditions with fluctuations in both temperature and glaciation events[54–56]. Corals in the genus *Acropora* show a similar pattern of expansion and contraction attributed to niche availability post mass extinction of shallow-water marine organisms 2-3 mya, followed by the unstable mid-Pleistocene climate[57,58]. A similar pattern of expansion and decline in effective population size has also been observed in the Antarctic ice fish among other marine organisms distributed in the Southern Hemisphere[59].

Phylogenomics

A total of 2,108 (single copy/ 1-to-1) orthologous clusters were identified between the molluscan genomes and transcriptomes of 11 species and used to construct a time-calibrated maximum likelihood tree(Fig 1a). The phylogenetic reconstruction estimated the divergence time between *H. maculosa* and its nearest relative, *O.*

bimaculoides, to be ~59 mya. *C. minor* diverged from this clade much earlier ~183 mya. Previous phylogenies using a combination of a small number of mitochondrial and nuclear genes[60–62] and orthologs derived from transcriptomes[63] support this topology. Likewise, estimates by Tanner et al.[2], using a concatenated alignment of 197 genes with a Bayesian approach, placed divergence of *H. maculosa* from *Abdopus aculeatus* at ~59 mya[2].

Inference of "shared" phenotypic traits can be difficult to resolve with the current literature. For example, false eye spots/ocelli observed in both *O. bimaculoides* and *H. maculosa* are structurally very different. Each ocellus in *H. maculosa* is composed of a continuous single blue ring[33], while *O. bimaculoides* has a blue ring composed of multiple small rings. Morphological variations of ocelli structure and colour, in conjunction with the taxonomically sporadic occurrence of this trait across species within *Octopus* and *Amphioctopus,* limits our interpretation as to the evolutionary history of this trait in octopods[3] . Large gaps remain in the literature between phenotypic traits in cephalopods and their genomic source[1]. This study aims to provide a genomic framework to enable resolution of these features by profiling changes in

several genomic characters: (i) gene duplications, (ii) novel gene formation, and (iii) non-coding element evolution.

Fig 1. Comparisons of molluscan genomes and gene families a) Time-calibrated maximum likelihood phylogeny of seven molluscan genomes (*Aplysia californica, Lottia gigantea, Crassostrea gigas, Euprymna scolopes, Octopus bimaculoides, Callistoctopus minor and Hapalochlaena maculosa*) and four transcriptomes (*Octopus kaurna, Octopus vulgaris, Sepia officinalis* and *Idiosepius notoides*) using 2,108 single copy orthologous sequence clusters. Node labels show divergence times in millions of years (mya), blue (divergence to octopods) and orange bars (decopods) represent standard error within a 95% confidence interval. Octopodiformes lineages are highlighted in blue and decapod orange. Scale bar represents millions of year (mya). **b)** Expansions of octopod gene families relative to molluscan genomes *Aplysia californica* (A. cali), *Biomphalaria glabrata* (B. glab), *Crassostrea gigas* (C. gig), *Lottia gigantea* (L. gig), *Euprymna scolopes* (E. scol) **c)** Lineage-specific gene expansions in the octopod genomes *Callistoctopus minor* (C. min), *Octopus bimaculoides* (O. bim) and *Hapalochlaena maculosa* (H. mac). Domains abbreviated: Chondroitin N-acetylgalactosaminyltransferase (CHGN), C2H2(Cys2-His2) zinc finger and Cornifin SPRR(small proline-rich proteins).

Organismal impact of novel genes and gene family expansions

Gene family expansions between octopods (*O. bimaculoides*, *C. minor* and *H. maculosa)* and three other molluscan genomes (*Aplysia californica*, *Lottia gigantea* and *Crassostrea gigas)* were examined using Pfam annotations. A total of 5565 Pfam domains were identified among six molluscan genomes. *H. maculosa* and *C. minor* exhibit expansions in the cadherin gene family, characteristic of other octopod genomes, including *O. bimaculoides* (Fig1b)[42,64]. *C. minor,* in particular, shows the greatest expansion of this family within octopods. Expansions of protocadherins, a subset of the cadherin family, have also occurred independently in squid[42], with the octopod expansions occurring post divergence ~135 mya[42]. The shared ancestry of octopod cadherins was also documented by Styfhals et al[64] using phylogenetic inference between *O. bimaculoides* and *O. vulgaris*.Cadherins, specifically protocadherins, play crucial roles in synapse formation, elimination and axon targeting within mammals and are essential mediators of short-range neuronal connections[65–68]. It should be noted that octopods lack a myelin sheath, as a result short-range connections are integral to maintaining signal fidelity over distance[6]. The independent expansions of protocadherins within chordate and cephalopod lineages are believed to be associated with increased neuronal complexity[42,64]. Elevated expression of

protocadherins within neural tissues have been observed in *O. vulgaris* and *O. bimaculoides* by both Styfhals et al[64] and Albertin et al[42] respectively. In particular Styfhals et al[64] noted differential expression across neural tissues including supra-esophageal mass, sub-esophageal mass, optic lobe and the stellate ganglion[64]. However, functional implications of observed expression patterns remain speculative without further study.

H. maculosa also shows expansions in the C2H2-type zinc finger family. Zinc fingers form an ancient family of transcription factors, which among other roles serve to regulate transposon splicing as well as embryonic and neural development[69,70]. Expansion of this type of zinc finger in *O. bimaculoides* has been associated with neural tissues. It should be noted that due to the inherent difficulty in fully annotating the zinc finger family, alternative methods were used to examine the number of exons in *C. minor* with high similarity to annotated zinc finger genes in *O. bimaculoides* (Supplementary notes 5.1). A total of 609 exons (not captured by published gene models) from *C. minor* were found with high similarity to accepted zinc finger genes in *O. bimaculoides,* suggesting this family is larger than that which the genome annotation infers.

Examination of genes specifically expressed within neural tissues found that cadherins were among the most highly expressed gene families of all octopod species. Particularly in *C. minor*, relative to the other octopods, such a trend reflects the gene family expansions found in this species (Fig2c). Zinc fingers were less pronounced, representing 1.1% of overall expression in *C. minor* compared to cadherins at 11.3%. Overall, neural tissues express a large diversity of Pfams with each species, exhibiting a similar profile and proportion of orthologous to lineage-specific genes.

Novel patterns of gene expression

High-level examination of gene dynamics (expression, loss of expression and absence of expression) between octopods across different levels of orthology provides insight into large-scale expression patterns and highlights lineage-specific loss of expression.

The greatest proportion of genes in each species examined were not specific to octopods or an octopus lineage (ancient genes) (Fig 2a). Expression of these genes were enriched in neural tissues across all species, indicating the core conservation of neural development and function. However, we also find that genes specific to each octopod

species also show this expression pattern. The overall elevated expression of genes within neural tissues could be reflective of the extensive neural network present in cephalopods, which comprises around 520 million nerve cells[71], rivalling vertebrates/mammals in size[6]. Expression of many novel genes in the nervous system may also indicate contribution of those genes to lineage-specific neural network evolution. In contrast, genes that date back to the shared octopod ancestor show highest expression in male reproductive tissues in all species.

Loss of expression between octopod genomes is exhibited most clearly in *H. maculosa* with 11% (1993 genes) of all ancient genes having no expression, compared to 1% in both *O. bimaculoides* and *C. minor*. Absence of gene expression for genes whose orthologs have retained expression in one or more other species suggests a unique evolutionary trajectory from other octopods. It should be noted that differences in tissue sampling may in part influence these values and due to the limited sampling of species, loss of expression cannot be inferred at a species level and may have occurred at any point in the lineage. In order to fully understand the implications of the gene family contractions and loss of expression in *H. maculosa*, relative to other octopods, further investigation is required.

Fig2. Dynamics of gene expression in octopod genomes. Proportion of gene expression across levels of specificity from not specific to octopods or an octopus species (left) to octopod-specific (middle) and lineage-specific (right). Donut plots show gene expression as some expression in any tissue (purple), no expression (blue) or expression that has been lost (dark blue). Loss of expression requires an ortholog of the gene to be expressed in one or more species and not expressed in the other species. Heatmaps at each specificity level show average expression of genes within their respective tissues, low expression (cream) to high expression (dark red).

Fig3. Dynamics of gene expression in neural and venom producing tissues of octopods. a) Tissue specific expression of genes within the brain of *H. maculosa, O. bimaculoides* and *C. minor* (red). Venn diagram shows numbers of shared and exclusive genes between species (Left). Bar chart of the top 5 Pfams and their contribution to overall expression in the brain (right). b) Tissue specific expression of genes within the posterior salivary gland (PSG) of *H. maculosa, O. bimaculoides* and *C. minor* (Blue). Venn diagram shows numbers of shared and exclusive genes between species (left). Bar chart of the top 5 Pfams and their contribution to overall expression in the PSG (right).

Evolution of the octopod non-coding genome

Similar to other cephalopod genomes, the *H. maculosa* genome has a high repeat content of 37.09% (bases masked). *O bimaculoides* and *C. minor* are also highly repetitive with 46.39% and 44% of their genomes composed of transposable elements (TE) respectively. Of the repetitive elements, LINEs dominate the decapodiform *Euprymna scolopes* genome accounting for its larger genome size[72], while SINEs are expanded in all four octopod genomes. SINEs have been previously documented in *O. bimaculoides* (7.86%)[42], comparable with *H. maculosa* (7.53%), while fewer SINEs were previously reported for *C. minor* (4.7%)[73]. SINE elements also dominate the *O. vulgaris* genome with an expansion occurring post divergence from *O. bimaculoides*[41]. Rolling circle (RC) elements are a prominent minor component in octopods, particularly in *H. maculosa*. RC transposons have been isolated from plant (*Zea mays*) and mammalian genomes. They depend greatly on proteins used in host DNA replication and are the only known class of eukaryotic mobile element (transposon) to have this dependence[74]. TE elements in cephalopod lineages show differing expansions between most of the genomes currently available, suggesting they are highly active and play a strong role in cephalopod evolution.

Enrichment of transposable elements associated with genes (flanking regions 10kb up- and downstream) was not observed compared to the whole genome for any species examined. More notable were differences between species, in particular *C. minor* shows a greater proportion of LINE to SINE elements relative to both *O. bimaculoides* and *H. maculosa*.

Together, this highlights a very dynamic evolutionary composition of repeats in cephalopods, that requires further study to test for any potential association with changes in gene expression or genome evolution.

Dynamics of gene expression in the posterior salivary gland (PSG)

The posterior salivary gland is the primary venom-producing gland in octopods. Venom composition in the majority of octopods is primarily composed of proteinaceous toxins. *Hapalochlaena* is an exception containing an additional non-proteinaceous neurotoxin, TTX, within their venom. We hypothesize that the *Hapalochlaena* PSG will exhibit a loss of redundant proteinaceous toxins due to the presence of TTX.

Examination of all PSG-specific genes from the three octopods revealed a disproportionate number of genes exclusive to *H. maculosa* (Fig 3a). A total of 623

genes were exclusive to *H. maculosa* PSG compared to only 230 and 164 exclusive to *O. bimaculoides* and *C. minor* PSGs, respectively. Additionally, we predict that the *H. maculosa* PSG is functionally more diverse based on the number of Pfam families detected, 532 in total. Comparatively, the PSG genes in *O. bimaculoides* and *C. minor* are fewer and more specialised. Gene family expansions of serine proteases dominate expression comprising over 30% of total PSG-specific expression in *C. minor* and 17-20% in *O. bimaculoides* (Fig 3b). Serine proteases were also among genes whose expression appears to have shifted between octopod species. Several serine proteases show specific expression to the PSG of *O. bimaculoides* and *C. minor* while being expressed in a non-specific pattern among brain, skin, muscle and anterior salivary gland tissues in *H. maculosa* (Fig 4b). Most notable is the absence of many paralogs in both *H. maculosa* and *O. bimaculoides* suggesting a lineage-specific expansion of this cluster in *C. minor*. Fewer serine protease genes can also be observed in *H. maculosa* (Fig 4c). Similarly, reprolysin (M12B) exhibits shifting expression in *H. maculosa*, presumably from the PSG to the branchial heart, and a complete loss of paralogs from the genome. While the function of this protein has not been assessed in octopus, members of this protein family exhibit anticoagulant properties in snake venom[75–78].

Serine proteases have been previously documented in cephalopod venom and are prime candidates for conserved toxins in octopods. Cephalopod-specific expansions have been identified with strong association to the PSG in 11 cephalopods (seven octopus, two squid and two cuttlefish)[8,13]. All serine proteases identified from the PSG of these species were found to belong to the cephalopod-specific clade. Functionally, cephalopod venom serine proteases have yet to be assessed. However, octopod venom has been observed to have strong digestive and hemolytic properties, which may be in part due to this crucial protein family[79–81]. The reduced number and expression of serine proteases in *H. maculosa* suggests a change in function of the PSG for this species. These results support the hypothesis of toxin redundancy in the *H. maculosa* PSG due to the incorporation of tetrodotoxin. Previous proteomic analysis of the *H. maculosa* PSG revealed high expression of hyaluronidase, which often serves as a dispersal factor within snake venom, facilitating the spread of toxin while not being directly toxic to their prey[9,82]. While further investigation is required, the incorporation of TTX within *H. maculosa* venom may have contributed to a shift in function, with proteins present acting to support the spread of venom and digestion of tissues.

Fig 4. Examination of posterior salivary gland (PSG) gene expression between three octopod genomes. a) Heatmap of genes expressed specifically in the PSG of *H. maculosa* (tau > 0.8) and their orthologs in *O. bimaculoides* and *C. minor* lacking specific expression to the PSG (tau < 0.8). Genes with an ortholog lacking expression are coloured in grey while the absence of an ortholog is white. **b)** Heatmap of genes expressed specifically in the (PSG) of both *O. bimaculoides* and *C. minor* (tau >0.8) and their orthologs in *H. maculosa* lacking specific expression to the PSG.

TTX resistance of the Na_v channels

To identify the mechanism of TTX resistance in *H. maculosa,* the voltage gated sodium channel (Na_v) sequences were compared between susceptible (human) and resistant (pufferfish, salamanders and garter snakes) species. TTX binds to the p-loop regions of sodium channels, inhibiting the flow of sodium ions in neurons, resulting in paralysis[83,84]. Inhibition of TTX binding has been observed in species which either ingest TTX via prey, such as garter snakes[85], and in those which retain TTX within their tissues like pufferfish[86].

Two Na_v genes were identified in the *H. maculosa* genome (Na_v1 and Na_v2), this is congruent with the recent identification of two Na_v isoforms in *H.*

lunulata[24](Supplementary Fig 8 & 9). Among cephalopods with sequenced Na$_v$1 channels, p-loop regions are highly conserved with both DI and DII shared between all species. The regions DIII and DIV closer to the C-terminal end of the protein in *Hapalochlaena* sp. contain mutations, which may impact TTX binding and differ between families and species as follows. Similar to the pufferfish (*Arothron, Canthigaster, Takifugu* and *Tetraodon*)[87]and garter snake *Thamnophis couchii*[88], *H. maculosa* Nav1 has a mutation within the third p-loop at site (DIII) from M1406T, while all other cephalopods have an Ile(I) at this position (Fig 5a). The dumbo octopus (*Grimpoteuthis*) is the only exception retaining the susceptible M at this site similar to humans and other non-resistant mammals[83]. Additionally, the fourth p-loop (DIV) in *H. maculosa* exhibits two substitutions at known TTX binding sites: D1669H and H1670S. In a previous study a Met to Thr substitution into a TTX sensitive Nav1.4 channel decreased binding affinity to TTX by 15-fold[87]. Likewise, a 10-fold increase in sensitivity was observed from a T1674M substitution in a mite (*Varroa destructor*) channel VdNav1[28]. However, resistance is often a result of multiple substitutions and when I1674T/D1967S occur together in VdNav1, resistance is multiplicative resulting in “super resistant” channels with binding inhibition of 1000-fold. The combination of

M1406T/ D1669H in *H. maculosa* also occurs in the turbellarian flatworm *Bdelloura candida*(BcNav1)[87,89]. While it has yet to be assessed for TTX resistance, the replacement of Asp in *B. candida* with a neutral amino acid has been predicted to disrupt TTX binding by preventing formation of a hydrogen bond[90]. These three substitutions (M1406T, D1669H and H1670S) in *H. maculosa*, with the potential to inhibit TTX binding, have also been identified by Geffeney et al[24] in *H. lunulata*. It has yet to be established if these mutations are derived from a shared ancestor or have occurred independently.

While *Hapalochlaena* remains the best documented example of TTX resistance among cephalopods, other species may contain some level of TTX resistance (e.g. *Octopus vulgaris*)[91,92]. Saxitoxin (STX) is a similar toxin in structure and function, and mutations resistant to TTX are often also STX inhibiting[93] *O. vulgaris* has been observed consuming STX-contaminated bivalves with no negative impacts and as such is believed to be resistant[92]. However, no mutations known to reduce TTX/STX binding affinity occur in its Nav1[92,94]. The selective pressure facilitating the evolution of STX/TTX resistance in these shallow water benthic octopods may be toxic prey, similar to garter snakes. STX is also known as a paralytic shellfish poison (PSP). Produced by

photosynthetic dinoflagellates and bioaccumulated in bivalves[95], this toxin contaminates a common octopus food source. Pelagic squids such as the Humboldt (*D. gigas*) and longfin inshore squid (*D. pealeii*) do not appear to be TTX/STX resistant; mass strandings of Humboldt squid have been associated with ingestion of STX-contaminated fish[96]. Likewise, no evidence of resistance was found in the sodium channel of the dumbo octopus (*Grimpoteuthis*). This species typically inhabits depths of 2000-5000m and is unlikely to encounter STX-contaminated food sources[97].

Fig 5. Mechanism of tetrodotoxin resistance within the posterior salivary gland of *H. maculosa* (PSG) a) Alignment of voltage gated sodium channel alpha subunits (DI, DII,DIII & DIV) p-loop regions. Mutations conferring resistance are coloured in green (pufferfish), orange (salamander), purple (clam) and blue (octopus). Susceptible mutations at the same site are Black and bolded. Sites which may be involved with resistance are in bold. **b)** Schematic of voltage-gated sodium channel (Na_v) alpha subunits (DI, DII, DIII and DIV). Each unit is composed of six subunits 1-4 (blue) and 5-6 (yellow). Alternating extra and intercellular loops are shown in black with the p-loops between subunits 5 and 6 highlighted in red. Mutations conferring resistance are shown within black circles on p-loops.

Microbiome of the PSG

TTX is produced through a wide variety of bacteria, which are common in marine sediments and have been isolated from organisms such as pufferfish[25,98,99]. Sequestration of TTX is not exclusive to the blue-ringed octopus among molluscs. Gastropods such as *Pleurobranchaea maculata*[100] and *Niotha clathrata*[30], as well as some bivalves, are also capable of sequestering TTX[95]. The commonly held hypothesis for TTX acquisition within *Hapalochlaena* is that it is bacterial in origin, and is either ingested or endosymbiotic[100,101]. Analysis of a ribo-depleted RNA sample from the PSG of *H. maculosa* revealed a highly diverse composition of bacterial genera with Simpson's and Shannon's diversity indices of 4.77 and 0.94, respectively. The dominant phyla were Proteobacteria and Firmicutes, composing respectively 41% and 22% of overall bacterial species detected (Fig 5a-b). To date, 151 strains of TTX-producing bacteria have been identified from 31 genera. Of these, 104 are members of Proteobacteria[102]. The genera *Pseudomonas* and *Bacillus* belonging to the phyla Proteobacteria and Firmicutes, respectively, have been previously identified in the PSG of *Hapalochlaena* sp *(Octopus maculosus)*[101]. Examination of these bacterial strains revealed TTX production, and extracts injected into mice proved to be lethal[101]. A more recent study on the bacterial composition of *H. maculosa* PSG did not identify TTX-producing strains[100].

However, only a small subset of the many strains identified were tested. Congruent with our findings the diversity of bacterial genera was high and this may complicate identification of species responsible for TTX production. The biosynthetic pathway of TTX has yet to be elucidated, and as a result, only culturable bacterial species can be tested for TTX production.

Fig 6. Assessment of bacteria within the posterior salivary gland of *H. maculosa* (PSG). **a)** Bacterial composition at the phylum level of a *H. maculosa* posterior salivary/venom gland. **b)** Composition of the largest Phylum, Protobacteria of a *H. maculosa* posterior salivary/venom gland.

Conclusions

This work describes the genome of a unique TTX bearing mollusc, the southern blue-ringed octopus (*Hapalochlaena maculosa*). Much of cephalopod evolution is barely understood due to sparseness of genomic data. Our analysis provides the first glimpse into genomic changes underlying genome evolution of closely related octopod species. While the size, heterozygosity and repetitiveness of the blue ring genome is congruent with previously published octopod genomes, we find similar yet independent expansions of key neuronal gene families across all three species and show evidence for

the involvement of gene novelty in the evolution of key neuronal, reproductive, and sensory tissues. The evolution of venom in octopods also differs between species, with *H. maculosa* showing a reduction in the number and expression of serine proteases in their venom gland relative to the other octopods in this study. Inclusion of TTX in *H. maculosa* distinguishes this species from related octopods and is believed to impact toxin recruitment and retention, as the highly potent TTX is sufficient to subdue common octopod prey without additional toxins.

Acknowledgements

We thank Dr Mark Norman and Colin Silvey for aiding Dr Julian Finn in collection of the two blue-ringed octopus specimens. We thank Jacqui Stuart for her brilliant illustrations and work on Fig beautification. This work was supported by an Australian Biological Resources Study (ABRS) grant (ref:RF211-41). O. Simakov was supported by the Austrian Science Fund (FWF) grant P30686-B29.

Ethics declaration

Animal Ethics Approval

Field collection of fishes, cephalopods (nautiluses, squids, cuttlefishes and octopuses} and decapod crustaceans (crabs, lobsters, crayfishes and their allies) in for Museum Victoria" (Animal Ethics Committee: Museums Victoria; AEC Approval Number: 10006)

Competing interests

Authors have no conflicts/competing interests to declare.

Data availability statement:

Genomic and transcriptomic data produced and used in this paper have been made available in the NCBI BioProject: PRJNA602771 under the following accession numbers: raw transcriptome(SAMN13930963 - SAMN13930975), genome assembly(to be provided). Voucher specimen for the transcriptome is stored at Melbourne museum (number to be provided). (Data is not yet available om NCBI as we would like to release it on publication).

Code availability statement:

Custom scripts and pipelines used in all of the analyses are published in GitHub(https://github.com/blwhitelaw/BRO_annotation).

Abbreviations

TTX: Tetrodotoxin, STX: Saxitoxin, PSG: Posterior Salivary Gland, CHGN:Chondroitin N-acetylgalactosaminyltransferase, C2H2(Cys2-His2) zinc finger, Cornifin SPRR:Small Proline-Rich Proteins, LINE: Long Interspersed Nuclear Element, SINE: Short Interspersed Nuclear Element, Mya: Million Years Ago, BUSCO: Benchmarking Universal Single-Copy Orthologs, PSMC: *Pairwise Sequentially Markovian Coalescent* and MSMC:multiple sequentially Markovian coalescent

Methods

Genome sequencing and assembly

DNA was extracted from a single *H. maculosa* female collected at Port Phillip Bay, Victoria, Australia. Two types of Illumina libraries were constructed, standard paired end and Illumina mate pairs (Supplementary data 2). Dovetail sequencing, Chicago

libraries improved upon original sequencing resulting in an overall coverage of 71X. Assembly-stats (https://github.com/sanger-pathogens/assembly-stats) was used to ascertain the quality of the assembly and relevant metrics (Supplementary notes 1).

Transcriptome sequencing

The *H. maculosa* transcriptome was generated using 12 tissues (brain, anterior salivary gland, digestive gland, renal, brachial heart, male reproductive tract, systemic heart, eyeballs, gills, posterior salivary gland, dorsal mantle and ventral mantle tissue). RNA was extracted using the Qiagen RNeasy kit. Construction of cDNA libraries was outsourced to AGRF (Australian Genome Research Facility), Melbourne and conducted using their TruSeq mRNA Library Prep with polyA selection and unique dual indexing method. Libraries were constructed using 3 μg of RNA at a concentration of >100 ng/μL. Each tissue was sequenced on 1/12th of an Illumina HiSeq2000 lane with one lane used in total.

De novo transcriptome assembly

De novo assembly of the *H. maculosa* transcriptome was conducted using sequencing data from 11 tissues (as listed above) and Trinity (v10.11.201). Default parameters were used aside from kmer coverage, which was set to three to account for the large data volume. Protein coding sequences were identified using Trinotate[103] and domains assigned by Interpro[104].

Genome annotation

Genes were annotated using a *de novo* predictor supplemented with transcriptomic evidence. Training models were produced by PASA[38] using a transcriptome composed of 12 tissues (as listed above) and supplied to the *de novo* predictor Augustus[39] along with intron, exon and repeat hints (generated by repeatmasker). Alternative splicing of gene models was also predicted using PASA. Methods used for annotation have been documented in the git https://github.com/blwhitelaw/BRO_annotation. Additional genes were predicted by mapping raw expressed reads against the genome. Functional annotation of gene models was achieved using InterPro v72.0[104]. Completeness of genes was assessed using BUSCO v3 Metazoan database[40].

Heterozygosity

JELLYFISH v2.2.1 was used in conjunction with GenomeScope[105] to calculate heterozygosity in *H. maculosa* using a kmer frequency of 21 (Supplementary table 5).

Repetitive and transposable elements

Repetitive and transposable elements were annotated using RepeatModeler v1.0.9 (RepeatScout) and masking performed with RepeatMasker v4.0.8[106](Supplementary notes 3.3). Analysis of gene associated TE was conducted by extracting TE within flanking regions 10K upstream and downstream of genes using Bedtools v2.27.1.

Calibration of sequence divergence with respect to time

Divergence times between the molluscan genomes (*Crassostrea gigas, Lottia gigantea, Aplysia californica, Euprymna scolopes, Octopus bimaculoides, Callistoctopus minor* and *Hapalochlaena maculosa*) and transcriptomes (*Sepia officinalis, idiosepius notoides, Octopus kaurna* and *Octopus vulgaris*) was obtained using a mutual best hit (MBH) approach. Bioprojects for each genome used are as follows: *Crassostera gigas* (PRJNA629593 & PRJEB3535), *Lottia gigantea* (PRJNA259762 & PRJNA175706),

Aplysia californica (PRJNA629593 & PRJNA13635) and (*Euprymna scolopes* PRJNA47095). *Octopus bimaculoides* was obtained from http://octopus.unit.oist.jp/OCTDATA/BASIC/Metazome/Obimaculoides_280.fa.gz. The *, Idiosepius notoides (*BioProject: PRJNA302677) transcriptome was sequenced and assembled using the same method previously described for the *H. maculosa* transcriptome. Whole genomes and transcriptomes were BLASTed against *Octopus bimaculoides*. The resulting hits were filtered, and alignments shared between all species extracted. A maximum likelihood phylogeny was generated using RAxML v8.0[107]. Phylobayes v3.3[108] was used to calculate divergence times (Supplementary 4.1).

Effective population size (PSMC)

Historical changes in effective population size were estimated using PSMC implemented in the software MSMC[109,110]. To generate inputs for MSMC we selected a subset of the reads used for genome assembly corresponding to 38x coverage of reads from libraries with short (500bp) insert sizes. These were pre-processed according to GATK best practices; briefly, adapters were marked with Picard 2.2.1, reads were mapped to the *H. maculosa* genome using bwa mem v 0.7.17 and PCR duplicates

identified using Picard v2.2.1. In order to avoid inaccuracies due to poor coverage or ambiguous read mapping we masked regions where short reads would be unable to find unique matches using SNPable (http://lh3lh3.users.sourceforge.net/snpable.shtml) and where coverage was more than double or less than half the genome wide average of 38x. Variant sites were called within unmasked regions and results converted to MSMC input format using msmc-tools https://github.com/stschiff/msmc-tools. All data for *H. maculosa* scaffolds of length greater than 1Mb was then used to generate 100 bootstrap replicates by dividing data into 500kb chunks and assembling them into 20 chromosomes with 100 chunks each. We then ran msmc2 on each bootstrap replicate and assembled imported the resulting data into R for plotting. A mutation rate of 2.4e-9 per base per year and a generation time of 1 year were assumed in order to set a timescale in years and convert coalescence rates to effective population size.

Mutation rate

Mutation rate was calculated by extracting orthologous genes from *O. bimaculoides* and *H. maculosa.* Neutrality was assumed for genes with very low expression (>10 TMP across all tissues). Neutral genes were aligned using MAFFT v7.407[111] and codeml[112]

was used to calculate substitution metrics (dS). Per base neutral substitution between lineages was determined using the mean dS value divided by divergence time (refer to *Calibration of sequence divergence with respect to time*) over the number of generations. As octopus are diploid the rate was divided by two. Divergence between species was calculated using Phylobayes v3.3[108].

Quantifying gene expression/ specificity

Gene expression within individual tissues was calculated using Kallisto[113] for the transcriptomic data sets of *H. maculosa*, *O. bimaculoides* and *C. minor*. Defaults were used and counts were calculated as TPM. Gene specificity was defined as any gene with a tau value > 0.80.

Gene model expression dynamics

Patterns of gene expression and loss were assessed across octopod genomes at differing taxonomic/organismal levels. Gene models were classified as lineage-specific, octopod specific or non-specific (orthologous to a gene outside of octopods). Expression at each level was determined using whole transcriptomes from all tissues of each species. Genes

with expression within one or more tissues were determined to be expressed, loss of expression was classified as a gene with a single ortholog in each species, which is expressed in one or more species and not expressed in the remaining species.

Dynamics of PSG gene expression

In order to identify patterns of PSG specific gene expression (losses and shifts) between the three available octopod genomes, genes with expression specific to the PSG of each species were examined separately. Specific gene expression was defined as a tau value > 0.8. Orthologous groups were identified between species using Orthovenn2[114] and sequences which were identified as lineage-specific were confirmed using BLAST. Types of expressions were categorized as follows: A loss of expression requires a gene to be present in all three octopods and expressed in one or more species while having no detectable expression in at least one species. A shift in expression occurs when an ortholog present in all species is expressed in different tissues.

The role of the Nav in TTX resistance

Sodium channels for the three octopus genomes along with all available in-house cephalopod transcriptomes were extracted manually using a series of BLAST searches against the nr database. Annotation was achieved using Interpro v72.0[104] and identification and extraction of p-loop regions of the sodium channel alpha subunit were manually performed. Where sodium channels were incomplete alignment against related complete channels were used to extract the p-loop regions. Individual mutations with potential to confer resistance were identified manually in Geneious v10.1 (https://www.geneious.com).

Microbiome of PSG

A single ribo-depleted RNA sample of *H. maculosa* PSG was examined using the SAMSA2 pipeline[115] to identify the bacterial composition and corresponding molecular functions. Two databases were used Subsys and NCBI RefBac. The Krona package[116] was used to produce visualizations of each dataset.

Supplementary Information

Supplementary Notes 1-8, Supplementary Tables 1-8, Supplementary Figs 1-10

Supplementary Data 2: Table of genomic Illumina library insert sizes

References

1. Ritschard, E. A. *et al.* Coupled Genomic Evolutionary Histories as Signatures of Organismal Innovations in Cephalopods. *BioEssays* (2019).
2. Tanner, A. R. *et al.* Molecular clocks indicate turnover and diversification of modern coleoid cephalopods during the Mesozoic Marine Revolution. *Proc. R. Soc. B Biol. Sci.* **284**, 20162818 (2017).
3. Jereb Roper, C. F. E., Norman, M. D., and Finn, J. K., P. Cephalopods of the world. An annotated and illustrated catalogue of cephalopod species known to date," in Octopods and Vampire Squids, Vol. 3,. *Rome FAO* (2014).
4. Graziadei, P. P. C. & Gagne, H. T. Sensory innervation in the rim of the octopus sucker. *J. Morphol.* **150**, 639–679 (1976).
5. Froesch, D. Projection of chromatophore nerves on the body surface of Octopus vulgaris. *Mar. Biol.* **19**, 153–155 (1973).
6. Budelmann, B. U. The cephalopod nervous system: what evolution has made of the molluscan design. in *The nervous systems of invertebrates: An evolutionary and comparative approach* 115–138 (Springer, 1995).
7. Gray, E. G. & Young, J. Z. Electron microscopy of synaptic structure of Octopus brain. *J. Cell Biol.* **21**, 87–103 (1964).
8. Fingerhut, L. C. H. W. *et al.* Shotgun proteomics analysis of saliva and salivary gland tissue from the common octopus Octopus vulgaris. *J. Proteome Res.* **17**, 3866–3876 (2018).
9. Whitelaw, B. L., Cooke, I. R., Finn, J., Zenger, K. & Strugnell, J. M. The evolution and origin of tetrodotoxin acquisition in the blue-ringed octopus (genus Hapalochlaena). *Aquat. Toxicol.* **206**, (2019).
10. Cooke, I. R., Whitelaw, B., Norman, M., Caruana, N. & Strugnell, J. M. Toxicity in

cephalopods. *Evol. Venom. Anim. Their Toxins* 1–15 (2015).

11. Whitelaw, B. L. *et al.* Combined Transcriptomic and Proteomic Analysis of the Posterior Salivary Gland from the Southern Blue-Ringed Octopus and the Southern Sand Octopus. *J. Proteome Res.* **15**, (2016).
12. Fry, B. G., Roelants, K. & Norman, J. A. Tentacles of venom: toxic protein convergence in the Kingdom Animalia. *J. Mol. Evol.* **68**, 311–321 (2009).
13. Ruder, T. *et al.* Molecular phylogeny and evolution of the proteins encoded by coleoid (cuttlefish, octopus, and squid) posterior venom glands. *J. Mol. Evol.* **76**, 192–204 (2013).
14. Fry, B. G. *et al.* Functional and structural diversification of the anguimorpha lizard venom system. *Mol. Cell. Proteomics* (2010) doi:10.1074/mcp.M110.001370.
15. Hendon, R. A. & Tu, A. T. Biochemical Characterization of the Lizard Toxin Gilatoxin. *Biochemistry* (1981) doi:10.1021/bi00515a033.
16. Matsui, T., Fujimura, Y. & Titani, K. Snake venom proteases affecting hemostasis and thrombosis. *Biochimica et Biophysica Acta - Protein Structure and Molecular Enzymology* (2000) doi:10.1016/S0167-4838(99)00268-X.
17. Mitreva, M. *et al.* Novel venom gene discovery in the platypus. *Genome Biol.* **11**, 1–13 (2010).
18. Choo, Y. M. *et al.* Antifibrinolytic role of a bee venom serine protease inhibitor that acts as a plasmin inhibitor. *PLoS One* (2012) doi:10.1371/journal.pone.0032269.
19. Veiga, S. S. *et al.* Identification of high molecular weight serine-proteases in Loxosceles intermedia (brown spider) venom. *Toxicon* (2000) doi:10.1016/S0041-0101(99)00197-X.
20. Modica, M. V., Lombardo, F., Franchini, P. & Oliverio, M. The venomous cocktail of the vampire snail Colubraria reticulata (Mollusca, Gastropoda). *BMC Genomics* **16**, 1–21 (2015).
21. von Reumont, B. M., Undheim, E. A. B., Jauss, R. T. & Jenner, R. A. Venomics of remipede crustaceans reveals novel peptide diversity and illuminates the venom's biological role. *Toxins (Basel).* (2017) doi:10.3390/toxins9080234.
22. Jaimes-Becerra, A. *et al.* Comparative proteomics reveals recruitment patterns of some protein families in the venoms of Cnidaria. *Toxicon* **137**, 19–26 (2017).

23. Crone, H. D., Leake, B., Jarvis, M. W. & Freeman, S. E. On the nature of "Maculotoxin", a toxin from the blue-ringed octopus (Hapalochlaena maculosa). *Toxicon* **14**, 423–426 (1976).

24. Geffeney, S. L. *et al.* Convergent and parallel evolution in a voltage-gated sodium channel underlies TTX-resistance in the Greater Blue-ringed Octopus: Hapalochlaena lunulata. *Toxicon* (2019).

25. Wu, Z. *et al.* Toxicity and distribution of tetrodotoxin-producing bacteria in puffer fish Fugu rubripes collected from the Bohai Sea of China. *Toxicon* **46**, 471–476 (2005).

26. Tsuruda, K. *et al.* Secretory glands of tetrodotoxin in the skin of the Japanese newt Cynops pyrrhogaster. *Toxicon* **40**, 131–136 (2002).

27. Wakely, J. F., Fuhrman, G. J., Fuhrman, F. A., Fischer, H. G. & Mosher, H. S. The occurrence of tetrodotoxin (tarichatoxin) in Amphibia and the distribution of the toxin in the organs of newts (Taricha). *Toxicon* **3**, 195–203 (1966).

28. Du, Y., Nomura, Y., Liu, Z., Huang, Z. Y. & Dong, K. Functional expression of an arachnid sodium channel reveals residues responsible for tetrodotoxin resistance in invertebrate sodium channels. *J. Biol. Chem.* **284**, 33869–33875 (2009).

29. McGlothlin, J. W. *et al.* Parallel evolution of tetrodotoxin resistance in three voltage-gated sodium channel genes in the garter snake Thamnophis sirtalis. *Mol. Biol. Evol.* **31**, 2836–2846 (2014).

30. Cheng, C. A. *et al.* Microflora and tetrodotoxin-producing bacteria in a gastropod, Niotha clathrata. *Food Chem. Toxicol.* **33**, 929–934 (1995).

31. Williams, B. L. & Caldwell, R. L. Intra-organismal distribution of tetrodotoxin in two species of blue-ringed octopuses (Hapalochlaena fasciata and H. lunulata). *Toxicon* **54**, 345–353 (2009).

32. Yotsu-Yamashita, M., Mebs, D. & Flachsenberger, W. Distribution of tetrodotoxin in the body of the blue-ringed octopus (Hapalochlaena maculosa). *Toxicon* **49**, 410–412 (2007).

33. Mäthger, L. M., Bell, G. R. R., Kuzirian, A. M., Allen, J. J. & Hanlon, R. T. How does the blue-ringed octopus (Hapalochlaena lunulata) flash its blue rings? *J. Exp. Biol.* **215**, 3752–3757 (2012).

34. Williams, B. L., Stark, M. R. & Caldwell, R. L. Microdistribution of tetrodotoxin in two species of blue-ringed octopuses (Hapalochlaena lunulata and Hapalochlaena

fasciata) detected by fluorescent immunolabeling. *Toxicon* **60**, 1307–1313 (2012).
35. Savage, I. V. E. & Howden, M. E. H. Hapalotoxin, a second lethal toxin from the octopus Hapalochlaena maculosa. *Toxicon* **15**, 463–466 (1977).
36. Kanda, A., Iwakoshi-Ukena, E., Takuwa-Kuroda, K. & Minakata, H. Isolation and characterization of novel tachykinins from the posterior salivary gland of the common octopus Octopus vulgaris. *Peptides* **24**, 35–43 (2003).
37. Ruder, T. *et al.* Functional characterization on invertebrate and vertebrate tissues of tachykinin peptides from octopus venoms. *Peptides* **47**, 71–76 (2013).
38. Haas, B. J. *et al.* Improving the Arabidopsis genome annotation using maximal transcript alignment assemblies. *Nucleic Acids Res.* **31**, 5654–5666 (2003).
39. Stanke, M. Gene prediction with a hidden Markov model. (University of Göttingen, 2004).
40. Waterhouse, R. M. *et al.* BUSCO applications from quality assessments to gene prediction and phylogenomics. *Mol. Biol. Evol.* **35**, 543–548 (2017).
41. Zarrella, I. *et al.* The survey and reference assisted assembly of the Octopus vulgaris genome. *Sci. data* **6**, 13 (2019).
42. Albertin, C. B. *et al.* The octopus genome and the evolution of cephalopod neural and morphological novelties. *Nature* **524**, 220–224 (2015).
43. Cai, H. *et al.* A draft genome assembly of the solar-powered sea slug Elysia chlorotica. *Sci. data* **6**, 190022 (2019).
44. Liu, C. *et al.* The genome of the golden apple snail Pomacea canaliculata provides insight into stress tolerance and invasive adaptation. *Gigascience* **7**, giy101 (2018).
45. Du, X. *et al.* The pearl oyster Pinctada fucata martensii genome and multi-omic analyses provide insights into biomineralization. *Gigascience* **6**, gix059 (2017).
46. Powell, D. *et al.* The genome of the oyster Saccostrea offers insight into the environmental resilience of bivalves. *DNA Res.* **25**, 655–665 (2018).
47. Sun, J. *et al.* Adaptation to deep-sea chemosynthetic environments as revealed by mussel genomes. *Nat. Ecol. Evol.* **1**, 121 (2017).
48. Uliano-Silva, M. *et al.* A hybrid-hierarchical genome assembly strategy to sequence the invasive golden mussel, Limnoperna fortunei. *Gigascience* **7**, gix128 (2017).

49. Zhang, G. *et al.* The oyster genome reveals stress adaptation and complexity of shell formation. *Nature* **490**, 49 (2012).
50. Jiao, W. *et al.* High-resolution linkage and quantitative trait locus mapping aided by genome survey sequencing: building up an integrative genomic framework for a bivalve mollusc. *DNA Res.* **21**, 85–101 (2013).
51. Garton, D. W. & Haag, W. R. Heterozygosity, shell length and metabolism in the European mussel, Dreissena polymorpha, from a recently established population in Lake Erie. *Comp. Biochem. Physiol. Part A Physiol.* **99**, 45–48 (1991).
52. Simmons, M. J. & Crow, J. F. Mutations affecting fitness in Drosophila populations. *Annu. Rev. Genet.* **11**, 49–78 (1977).
53. Kumar, S. & Subramanian, S. Mutation rates in mammalian genomes. *Proc. Natl. Acad. Sci.* **99**, 803–808 (2002).
54. Hayward, B. W., Kawagata, S., Grenfell, H. R., Sabaa, A. T. & O'Neill, T. Last global extinction in the deep sea during the mid-Pleistocene climate transition. *Paleoceanogr. Paleoclimatology* **22**, (2007).
55. Hofreiter, M. & Stewart, J. Ecological change, range fluctuations and population dynamics during the Pleistocene. *Curr. Biol.* **19**, R584–R594 (2009).
56. Reynolds, T. V, Matthee, C. A. & Von Der Heyden, S. The influence of Pleistocene climatic changes and ocean currents on the phylogeography of the southern African barnacle, Tetraclita serrata (Thoracica; Cirripedia). *PLoS One* **9**, e102115 (2014).
57. Huber, C. D., DeGiorgio, M., Hellmann, I. & Nielsen, R. Detecting recent selective sweeps while controlling for mutation rate and background selection. *Mol. Ecol.* **25**, 142–156 (2016).
58. Mao, Y., Economo, E. P. & Satoh, N. The roles of introgression and climate change in the rise to dominance of Acropora Corals. *Curr. Biol.* **28**, 3373-3382. e5 (2018).
59. Kim, B.-M. *et al.* Antarctic blackfin icefish genome reveals adaptations to extreme environments. *Nat. Ecol. Evol.* **3**, 469 (2019).
60. Guzik, M. T., Norman, M. D. & Crozier, R. H. Molecular phylogeny of the benthic shallow-water octopuses (Cephalopoda: Octopodinae). *Mol. Phylogenet. Evol.* **37**, 235–248 (2005).
61. Strugnell, J., Jackson, J., Drummond, A. J. & Cooper, A. Divergence time

estimates for major cephalopod groups: evidence from multiple genes. *Cladistics* **22**, 89–96 (2006).
62. Strugnell, J. M., Rogers, A. D., Prodöhl, P. A., Collins, M. A. & Allcock, A. L. The thermohaline expressway: the Southern Ocean as a centre of origin for deep-sea octopuses. *Cladistics* **24**, 853–860 (2008).
63. Lindgren, A. R. & Anderson, F. E. Assessing the utility of transcriptome data for inferring phylogenetic relationships among coleoid cephalopods. *Mol. Phylogenet. Evol.* **118**, 330–342 (2018).
64. Styfhals, R., Seuntjens, E., Simakov, O., Sanges, R. & Fiorito, G. In silico Identification and Expression of Protocadherin Gene Family in Octopus vulgaris. *Frontiers in Physiology* (2019) doi:10.3389/fphys.2018.01905.
65. Zipursky, S. L. & Sanes, J. R. Chemoaffinity revisited: dscams, protocadherins, and neural circuit assembly. *Cell* **143**, 343–353 (2010).
66. Tepass, U., Truong, K., Godt, D., Ikura, M. & Peifer, M. Cadherins in embryonic and neural morphogenesis. *Nat. Rev. Mol. Cell Biol.* **1**, 91 (2000).
67. Peek, S. L., Mah, K. M. & Weiner, J. A. Regulation of neural circuit formation by protocadherins. *Cellular and Molecular Life Sciences* (2017) doi:10.1007/s00018-017-2572-3.
68. De Wit, J. & Ghosh, A. Specification of synaptic connectivity by cell surface interactions. *Nature Reviews Neuroscience* (2016) doi:10.1038/nrn.2015.3.
69. Layden, M. J., Meyer, N. P., Pang, K., Seaver, E. C. & Martindale, M. Q. Expression and phylogenetic analysis of the zic gene family in the evolution and development of metazoans. *Evodevo* **1**, 12 (2010).
70. Liu, H., Chang, L.-H., Sun, Y., Lu, X. & Stubbs, L. Deep vertebrate roots for mammalian zinc finger transcription factor subfamilies. *Genome Biol. Evol.* **6**, 510–525 (2014).
71. Young, J. Z. The number and sizes of nerve cells in Octopus. in *Proceedings of the Zoological Society of London* vol. 140 229–254 (Wiley Online Library, 1963).
72. Belcaid, M. *et al.* Symbiotic organs shaped by distinct modes of genome evolution in cephalopods. *Proc. Natl. Acad. Sci.* 201817322 (2019) doi:10.1073/pnas.1817322116.
73. Kim, B.-M. *et al.* The genome of common long-arm octopus Octopus minor. *Gigascience* **7**, giy119 (2018).

74. Kapitonov, V. V & Jurka, J. Rolling-circle transposons in eukaryotes. *Proc. Natl. Acad. Sci.* **98**, 8714–8719 (2001).
75. Oulion, B. *et al.* Factor X activating Atractaspis snake venoms and the relative coagulotoxicity neutralising efficacy of African antivenoms. *Toxicol. Lett.* (2018) doi:10.1016/j.toxlet.2018.02.020.
76. Rogalski, A. *et al.* Differential procoagulant effects of saw-scaled viper (Serpentes: Viperidae: Echis) snake venoms on human plasma and the narrow taxonomic ranges of antivenom efficacies. *Toxicol. Lett.* **280**, 159–170 (2017).
77. Sousa, L. F. *et al.* Coagulotoxicity of Bothrops (lancehead pit-vipers) venoms from Brazil: Differential biochemistry and antivenom efficacy resulting from prey-driven venom variation. *Toxins (Basel).* (2018) doi:10.3390/toxins10100411.
78. Fox, J. W. & Serrano, S. M. T. Structural considerations of the snake venom metalloproteinases, key members of the M12 reprolysin family of metalloproteinases. *Toxicon* **45**, 969–985 (2005).
79. Grisley, M. S. & Boyle, P. R. Bioassay and proteolytic activity of digestive enzymes from octopus saliva. *Comp. Biochem. Physiol. Part B Comp. Biochem.* **88**, 1117–1123 (1987).
80. Key, L. N., Boyle, P. R. & Jaspars, M. Novel activities of saliva from the octopus Eledone cirrhosa (Mollusca; Cephalopoda). *Toxicon* **40**, 677–683 (2002).
81. McDonald, N. M. & Cottrell, G. A. Purification and mode of action of toxin from Eledone cirrosa. *Comp. Gen. Pharmacol.* **3**, 243–248 (1972).
82. Bordon, K. C. F., Perino, M. G., Giglio, J. R. & Arantes, E. C. Isolation, enzymatic characterization and antiedematogenic activity of the first reported rattlesnake hyaluronidase from Crotalus durissus terrificus venom. *Biochimie* **94**, 2740–2748 (2012).
83. Frank, H. Y. & Catterall, W. A. Overview of the voltage-gated sodium channel family. *Genome Biol.* **4**, 207 (2003).
84. How, C.-K., Chern, C.-H., Huang, Y.-C., Wang, L.-M. & Lee, C.-H. Tetrodotoxin poisoning. *Am. J. Emerg. Med.* **21**, 51–54 (2003).
85. Brodie III, E. D. & Brodie Jr, E. D. Tetrodotoxin resistance in garter snakes: an evolutionary response of predators to dangerous prey. *Evolution (N. Y).* **44**, 651–659 (1990).
86. Nakamura, M. & Yasumoto, T. Tetrodotoxin derivatives in puffer fish. *Toxicon* **23**,

271–276 (1985).
87. Jost, M. C. *et al.* Toxin-resistant sodium channels: parallel adaptive evolution across a complete gene family. *Mol. Biol. Evol.* **25**, 1016–1024 (2008).
88. Feldman, C. R., Brodie, E. D. & Pfrender, M. E. Constraint shapes convergence in tetrodotoxin-resistant sodium channels of snakes. *Proc. Natl. Acad. Sci.* **109**, 4556–4561 (2012).
89. Jeziorski, M. C., Greenberg, R. M. & Anderson, P. A. V. Cloning of a putative voltage-gated sodium channel from the turbellarian flatworm Bdelloura candida. *Parasitology* **115**, 289–296 (1997).
90. Geffeney, S. L., Fujimoto, E., Brodie III, E. D., Brodie Jr, E. D. & Ruben, P. C. Evolutionary diversification of TTX-resistant sodium channels in a predator–prey interaction. *Nature* **434**, 759 (2005).
91. Flachsenberger, W. & Kerr, D. I. B. Lack of effect of tetrodotoxin and of an extract from the posterior salivary gland of the blue-ringed octopus following injection into the octopus and following application to its brachial nerve. *Toxicon* **23**, 997–999 (1985).
92. Lopes, V. M., Baptista, M., Repolho, T., Rosa, R. & Costa, P. R. Uptake, transfer and elimination kinetics of paralytic shellfish toxins in common octopus (Octopus vulgaris). *Aquat. Toxicol.* **146**, 205–211 (2014).
93. Ulbricht, W., Wagner, H. & Schmidtmayer, J. Kinetics of TTX-STX Block of Sodium Channels. *Ann. N. Y. Acad. Sci.* **479**, 68–83 (1986).
94. Monteiro, A. & Costa, P. R. Distribution and selective elimination of paralytic shellfish toxins in different tissues of Octopus vulgaris. *Harmful Algae* **10**, 732–737 (2011).
95. Li, S.-C., Wang, W.-X. & Hsieh, D. Feeding and absorption of the toxic dinoflagellate Alexandriumtamarense by two marine bivalves from the South China Sea. *Mar. Biol.* **139**, 617–624 (2001).
96. Braid, H. E. *et al.* Preying on commercial fisheries and accumulating paralytic shellfish toxins: a dietary analysis of invasive Dosidicus gigas (Cephalopoda Ommastrephidae) stranded in Pacific Canada. *Mar. Biol.* **159**, 25–31 (2012).
97. Shea, E. K., Ziegler, A., Faber, C. & Shank, T. M. Dumbo octopod hatchling provides insight into early cirrate life cycle. *Curr. Biol.* **28**, R144–R145 (2018).
98. Lee, M.-J. *et al.* A tetrodotoxin-producing Vibrio strain, LM-1, from the puffer fish

Fugu vermicularis radiatus. *Appl. Environ. Microbiol.* **66**, 1698–1701 (2000).
99. Yotsu, M. *et al.* Production of tetrodotoxin and its derivatives by Pseudomonas sp. isolated from the skin of a pufferfish. *Toxicon* **25**, 225–228 (1987).
100. Chau, R., Kalaitzis, J., Wood, S. & Neilan, B. Diversity and biosynthetic potential of culturable microbes associated with toxic marine animals. *Mar. Drugs* **11**, 2695–2712 (2013).
101. Hwang, D. F. *et al.* Tetrodotoxin-producing bacteria from the blue-ringed octopus Octopus maculosus. *Mar. Biol.* **100**, 327–332 (1989).
102. Magarlamov, T., Melnikova, D. & Chernyshev, A. Tetrodotoxin-producing bacteria: Detection, distribution and migration of the toxin in aquatic systems. *Toxins (Basel).* **9**, 166 (2017).
103. Bryant, D. M. *et al.* A tissue-mapped axolotl de novo transcriptome enables identification of limb regeneration factors. *Cell Rep.* **18**, 762–776 (2017).
104. Jones, P. *et al.* InterProScan 5: genome-scale protein function classification. *Bioinformatics* **30**, 1236–1240 (2014).
105. Vurture, G. W. *et al.* GenomeScope: fast reference-free genome profiling from short reads. *Bioinformatics* **33**, 2202–2204 (2017).
106. Smit Hubley, R & Green, P., A. F. A. RepeatMasker Open-4.0.
107. Stamatakis, A. RAxML-VI-HPC: maximum likelihood-based phylogenetic analyses with thousands of taxa and mixed models. *Bioinformatics* **22**, 2688–2690 (2006).
108. Guindon, S. & Gascuel, O. A simple, fast, and accurate algorithm to estimate large phylogenies by maximum likelihood. *Syst. Biol.* **52**, 696–704 (2003).
109. Li, H. & Durbin, R. Inference of human population history from whole genome sequence of a single individual. *Nature* **475**, 493 (2012).
110. Schiffels, S. & Durbin, R. Inferring human population size and separation history from multiple genome sequences. *Nat. Genet.* **46**, 919 (2014).
111. Rozewicki, J., Li, S., Amada, K. M., Standley, D. M. & Katoh, K. MAFFT-DASH: integrated protein sequence and structural alignment. *Nucleic Acids Res.* **47**, W5–W10 (2019).
112. Adachi, J. & Hasegawa, M. *MOLPHY version 2.3: programs for molecular phylogenetics based on maximum likelihood*. (Institute of Statistical Mathematics Tokyo, 1996).
113. Bray, N. L., Pimentel, H., Melsted, P. & Pachter, L. Near-optimal probabilistic

RNA-seq quantification. *Nat. Biotechnol.* **34**, 525–527 (2016).
114. Xu, L. *et al.* OrthoVenn2: a web server for whole-genome comparison and annotation of orthologous clusters across multiple species. *Nucleic Acids Res.* **47**, W52–W58 (2019).
115. Westreich, S. T., Korf, I., Mills, D. A. & Lemay, D. G. SAMSA: a comprehensive metatranscriptome analysis pipeline. *BMC Bioinformatics* **17**, 399 (2016).
116. Ondov, B. D., Bergman, N. H. & Phillippy, A. M. Interactive metagenomic visualization in a Web browser. *BMC Bioinformatics* **12**, 385 (2011).

Figure1

Click here to access/download;Figure;Fig1.pdf

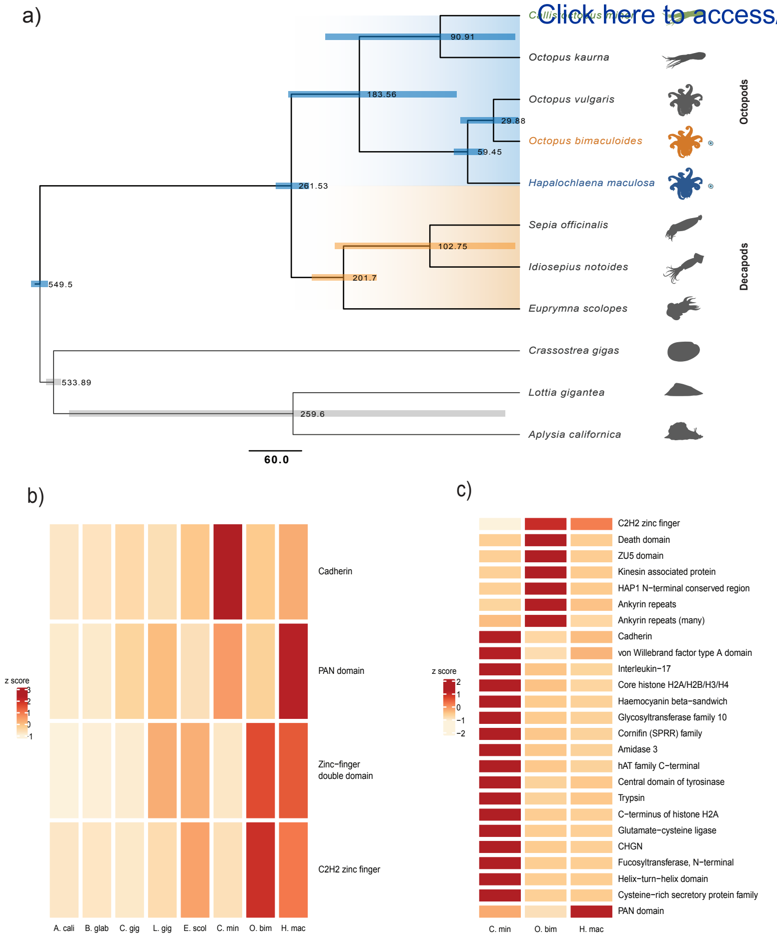

Revised Figure 2

Click here to access/download;Figure;Fig2_LSP.pdf

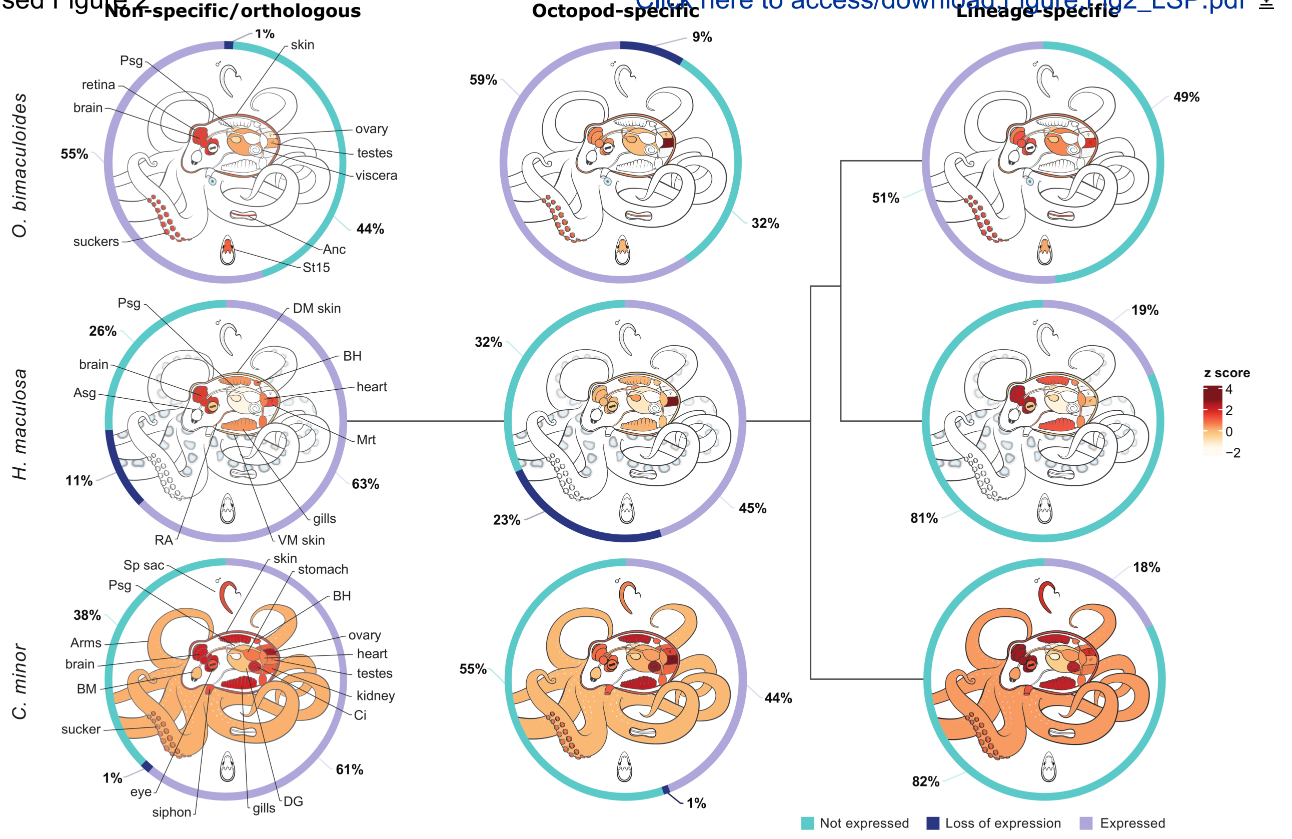

Figure3

Click here to access/download;Figure;Fig3_V2.pdf

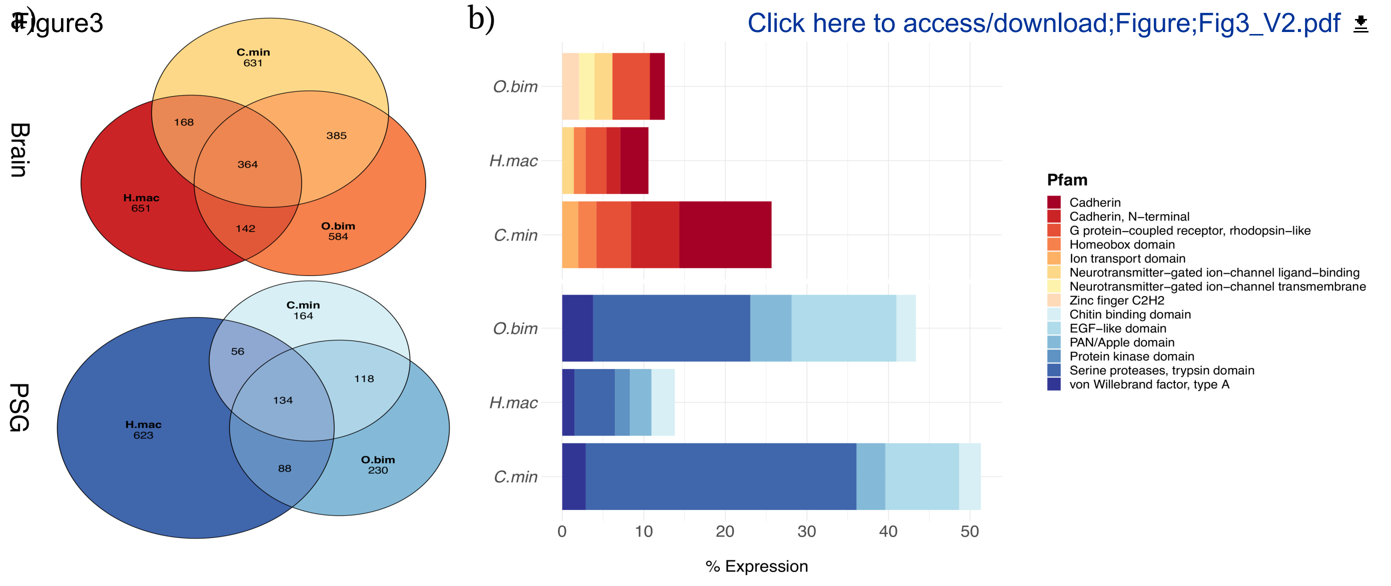

Figure4

Click here to access/download;Figure;Fig4.pdf

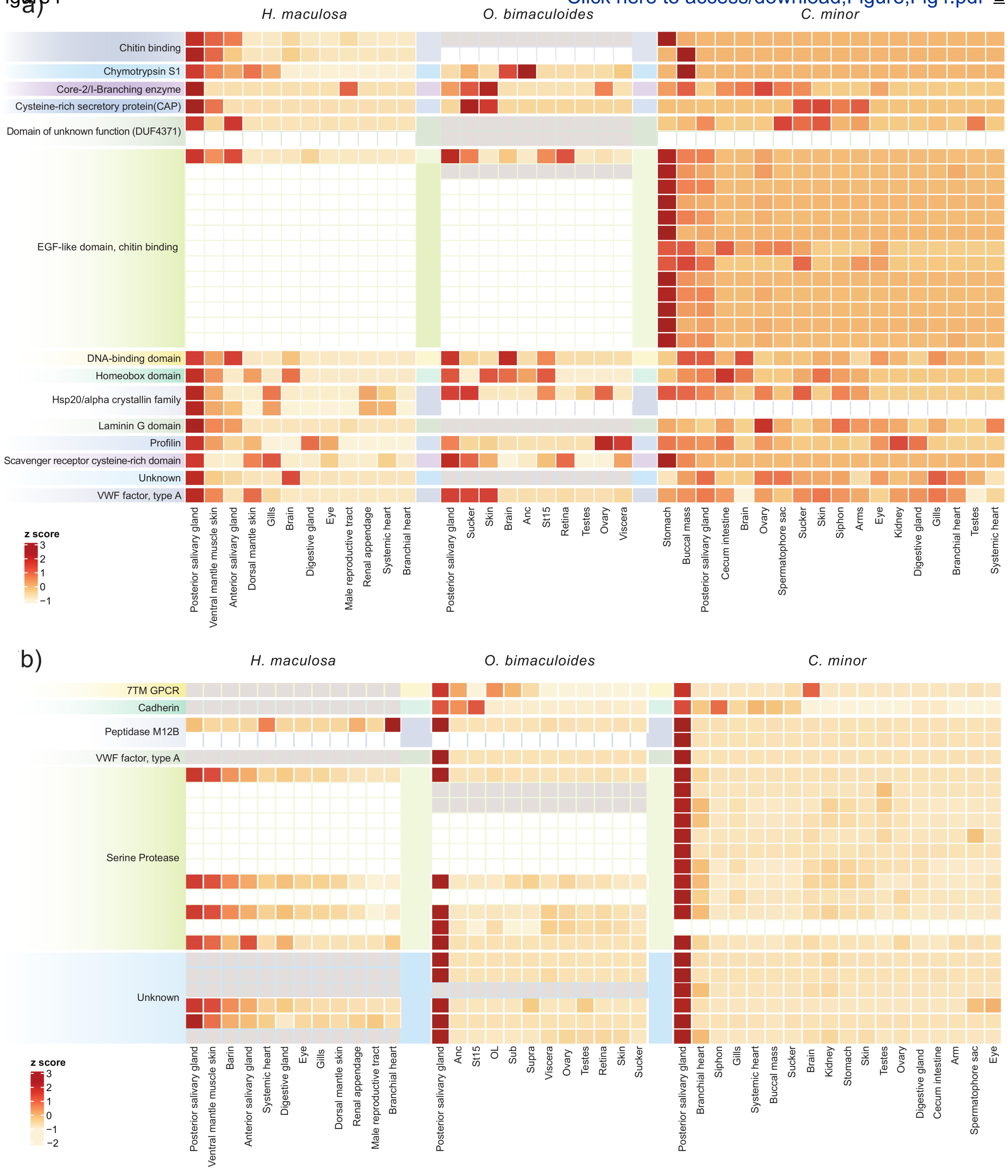

Click here to access/download;Figure;Fig5_RTR_Au

a)

Revised Figure 5

consensus	DI<br>DYWEN	DII<br>EWIES	DIII<br>KGWXD	DIV<br>AGWDG
Mya arenaria	-----	---D-	---I-	-----
Crassostrea gigas	F----	---Q-	---IE	-----
Mizuhopecten yessoensis	-----	-----	---TV	----S
Lottia gigantea	---S-	-----	---V-	-----
Aplysia californica	F--S-	-----	---I-	---SD
Euprymna scolopes	-----	-----	---IN	-----
Doryteuthis pealeii	-----	-----	---IN	-----
Doryteuthis opalescens		-----	---IN	-----
Dosidicus gigas	-----	-----	---IN	
Grimpoteuthis	-----	-----	---M-	-----
Callistoctopus minor	-----	-----		-----
Octopus bimaculoides	-----	-----	---I-	-----
Octopus vulgaris	-----	-----	---I-	-----
Hapalochlaena maculosa	-----	-----	---TE	---HS ☠
Hapalochlaena lunulata	-----	-----	---TE	---HS ☠
Taricha granulosa	-----	----T	---T-	---SD ☠
Tetraodon nigroviridis	C----	----N	---TA	GG--Q ☠
Homo sapiens	-----	----T	---M-	----G

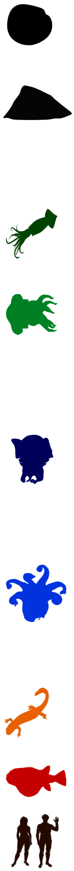

b)

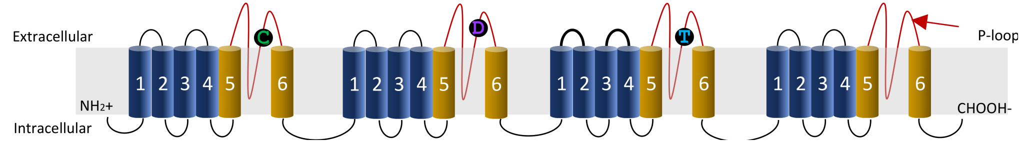

Figure6

Click here to access/download;Figur

a)

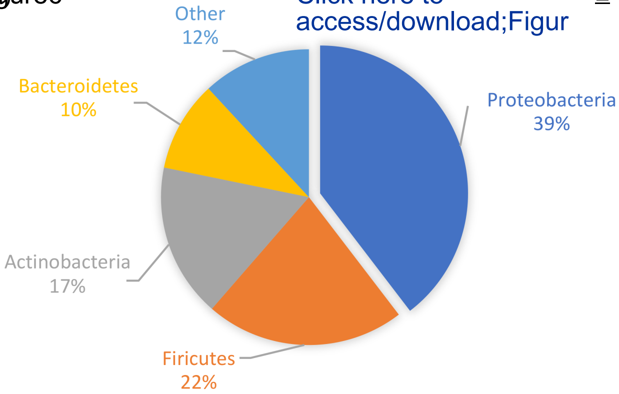

b)

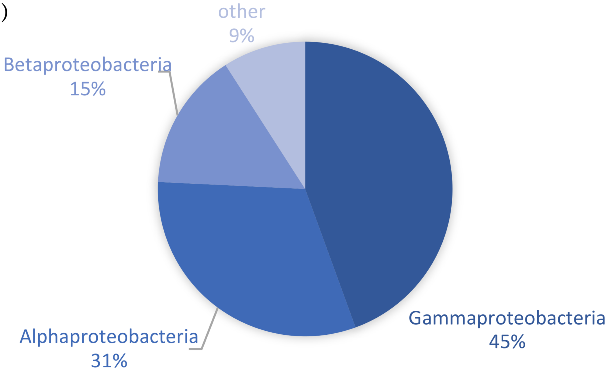

Click here to access/download
Supplementary Material
SUPPLEMENTARY MATERIALS_GS2_5_8_2020.docx

Supplementary Material(insert sizes)

Click here to access/download
Supplementary Material
BRO Sequencing Insert Sizes.xlsx

Cover Letter

Click here to access/download;Personal Cover;Cover letter_GS.docx

Dear Dr. Goodman

I am pleased to submit an original research piece titled "**Adaptive venom evolution and toxicity in octopods is driven by extensive novel gene formation, expansion and loss** " for consideration to be published in *GigaScience*.

Much of cephalopod evolution remains unknown due to sparseness of their genomic sampling. Cephalopod genomes are some of the largest and most repetitive animal genomes and exhibit drastically different evolutionary trajectories relative to other better documented lineages. A more focused genomic study to reveal how individual genomic changes are associated with the evolution of novel organs, tissues, or adaptations, within a single group of cephalopods has been missing so far. We present such a study, focussing on adaptations in the toxic blue-ringed octopus the *Hapalochlaena maculosa*, for which we provide a high quality genome assembly based on multiple technologies. Members of the genus *Hapalochlaena* are the only octopods to contain the lethal neurotoxin, tetrodotoxin (TTX), within their venom and tissues and are a prime example of the origin of evolutionary novelties within octopods.

Using global comparative genomics approaches and focused study on TTX evolution, we report key findings:

- Gene family expansions crucial for the development of complex neural networks are present in cephalopods, yet are differentially expanding in all three octopod species
- Novel gene formation at different phylogenetic levels can be associated with evolution in a specific set of cephalopod tissues
- Changes in Posterior Salivary Gland composition (PSG) between TTX bearing and non-TTX bearing species
- Convergently evolved mutations consistent with TTX resistance detected in *H. maculosa*

We firmly believe that our manuscript is suited for publication by *GigaScience* as one of the first to explore the evolutionary genomic basis for novelties within octopods and cephalopods in general. Our whole genomic comparisons provide insight into the defining structure/features of octopod genomes at the species-specific level. Additionally, we

examine the impact of TTX on the evolution of venom in *H. maculosa* relative to non-TTX bearing octopods.

Yours sincerely,

Brooke L. Whitelaw
James Cook University,
Townsville, QLD 4810
Mobile: 0424642621
brooke.whitelaw@my.jcu.edu.au

Assoc. Prof. Jan Strugnell
James Cook University,
Townsville, QLD 4810
jan.strugnell.jcu.edu.au

and

Prof. Oleg Simakov
University of Vienna
Universitätsring 1, 1010 Wien, Austria
oleg.simakov@univie.ac.at

Click here to access/download;Personal Cover;Cover letter_GS_RTR.docx

Dear Dr. Hongling Zhou

On behalf of my co-authors, I would like to thank you for the opportunity to revise and resubmit our research piece titled "*Adaptive venom evolution and toxicity in octopods is driven by extensive novel gene formation, expansion and loss*" (GIGA-D-20-00135).

We would also like thank all three reviewers for their constructive criticism and advice on how to improve our manuscript. All suggestions have been taken into account and incorporated resulting in a much-improved manuscript.

A response to reviewers has been provided in which we address each point providing the original text followed by the revised text, including line numbers where changes have been implemented. We also included a low resolution version of all corrected figures in the response.

Thank you again for your consideration of our revised manuscript

Yours sincerely,

Brooke L. Whitelaw
James Cook University,
Townsville, QLD 4810
Mobile: 0424642621
brooke.whitelaw@my.jcu.edu.au

Assoc. Prof. Jan Strugnell
James Cook University,
Townsville, QLD 4810
jan.strugnell.jcu.edu.au

and

Prof. Oleg Simakov
University of Vienna
Universitätsring 1, 1010 Wien, Austria
oleg.simakov@univie.ac.at
