# Supplementary material for: Adaptive venom evolution and toxicity in octopods is driven by extensive novel gene formation, expansion, and loss
Source: Gigascience. 2020 Nov 10;9(11):giaa120. doi: 10.1093/gigascience/giaa120 (PMC7656900; doi:10.1093/gigascience/giaa120)
Supplement: giaa120_Supplemental_Files [file giaa120_supplemental_files.zip › SUPPLEMENTARY MATERIALS_GS2_5_8_2020.docx]

**SUPPLEMENTARY MATERIALS FOR** “*Adaptive venom evolution and toxicity in octopods is driven by extensive novel gene formation, expansion and loss*”

**1. GENOME SEQUENCING, ASSEMBLY AND ANALYSES**

**1.1. Library preparation**

**1.2. Sequencing and assembly**

**1.3. Assembly statistics**

**1.4. Genome heterozygosity estimate**

**1.5. Mutation rate**

**1.6. Effective population size/Pairwise Sequentially Markovian Coalescent (PSMC) analysis**

**2. TRANSCRIPTOME SEQUENCING AND ANALYSIS**

**2.1. Tissues sampled, RNA preparation and sequencing**

**2.2. Mapping reads to the genome for expression analysis**

**2.3. *de novo* transcriptome assembly using *Trinity***

**3. ANNOTATION OF TRANSPOSABLE ELEMENTS AND PROTEIN CODING GENES**

**3.1. Annotation of protein coding genes**

**3.2. Annotation completeness**

**3.3. Transposable element annotation and expansions**

**4. MULTI-GENE PHYLOGENY AND GENE FAMILY EXPANSION ANALYSES**

**4.1. Multi-gene cephalopod phylogeny and dating**

**4.2. Genome-wide gene family expansions**

**5. ANALYSIS OF NEURAL ASSOCIATED GENE FAMILIES**

**5.1. Zinc finger C2H2**

**5.2. Cadherin/Protocadherin**

**6. EVOLUTION OF THE VENOM/POSTERIOR SALIVARY GLAND IN OCTOPODS**

**6.1. Extraction of tissue specific genes**

**6.2. Examination of gene families and orthology**

**6.3. Gene loss, shift**

**6.4. Examination of selection and evolutionary rates in octopod serine proteases**

**7. EVOLUTION OF TETRODOTOXIN RESISTANCE IN *H. MACULOSA***

**7.1. Extraction and identification of Nav channels and p-loop regions**

**7.2. Identification of tetrodotoxin(TTX) resistance mutation**

**8. MICROBIOME OF THE *H. MACULOSA* POSTERIOR SALIVARY GLAND**

**8.1. SAMSA**

**1. GENOME SEQUENCING, ASSEMBLY AND ANALYSES**

**1.1. Library preparation**

A single female specimen of *Hapalochlaena maculosa* was collected at Beaumaris Sea Scout Boat Shed, Beaumaris, Port Phillip bay, Australia (37°59'43.70"S 145°21.17"E) at a depth of 3m. The whole animal was stored at -80°C immediately after sampling. DNA was extracted from muscle tissue using QIAmp DNA mini kit. Illumina library preparation was conducted at the genome institute in Washington University, USA.

In order to increase coverage and improve the continuity of contig assembly a total of 4g of arm and mantle tissue was submitted to DovetailGenomics which was then used to generate a Chicago™ library. Resulting Dovetail library concentration was 13.8nM and 4.2ng/ul with a mean size of 488nt and a total volume of 30ul.

**1.2. Sequencing and assembly**

The Illumina library was sequenced using Illumina HiSeq 2000 at the genome institute in Washington University. Insert sizes for mate pair libraries can be found in the attached Supplementary data 2. Reads were trimmed with trimgalore (<https://www.bioinformatics.babraham.ac.uk/projects/trim_galore/>) to remove adapters. Several runs of Meraculous (meraculoususing) different kmer sizes were used to determine the optimal kmer (51). Dovetail sequencing was used to improve on the Illumina assembly. A HiRise ™ assembly was conducted by Dovetail and compared against the original assembly (Table 2 & 3, Figure 1).

Table 1. Summary for Illumina libraries.

| Read Type | Coverage at 4.3GB |
| --- | --- |
| All | 39X |
| 700bp insert | 13X |
| 3-8kb insert | 19X |

Table 2. Comparison of original Illumina and Dovetail augmented assemblies.


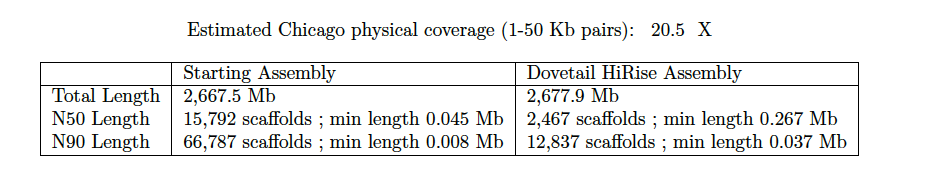


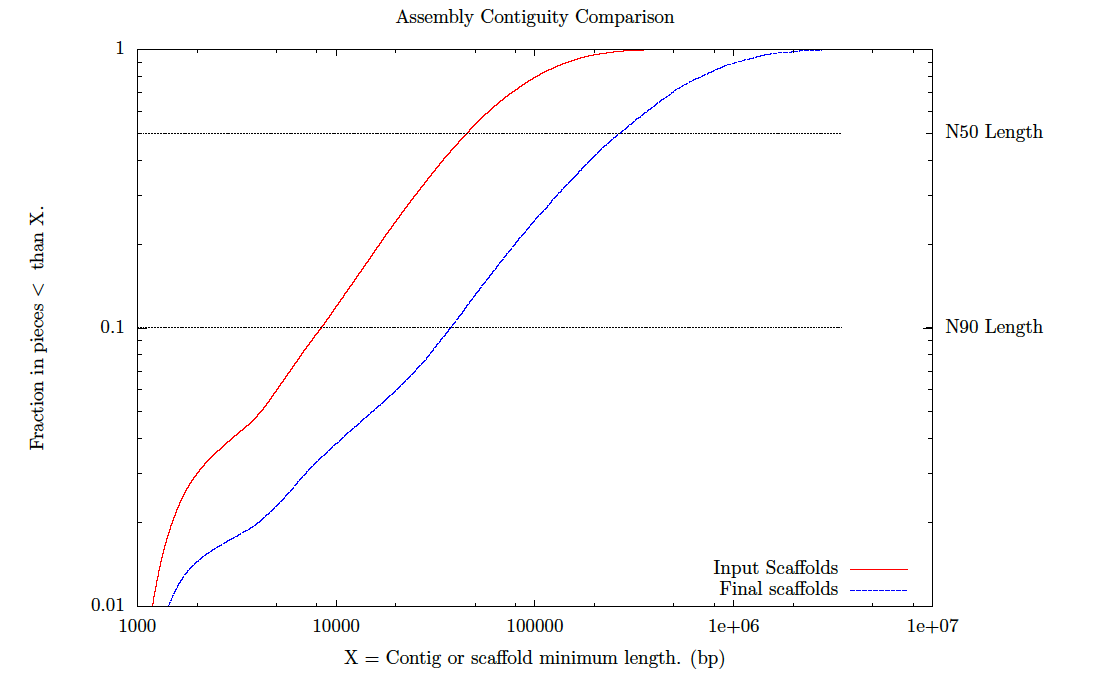


Figure 1. Comparison of assembly continuity between original Illumina (input scaffolds) and Dovetail (Final scaffolds) augmented assemblies.

Table 3. Statistical comparisons between original Illumina and Dovetail augmented assemblies.


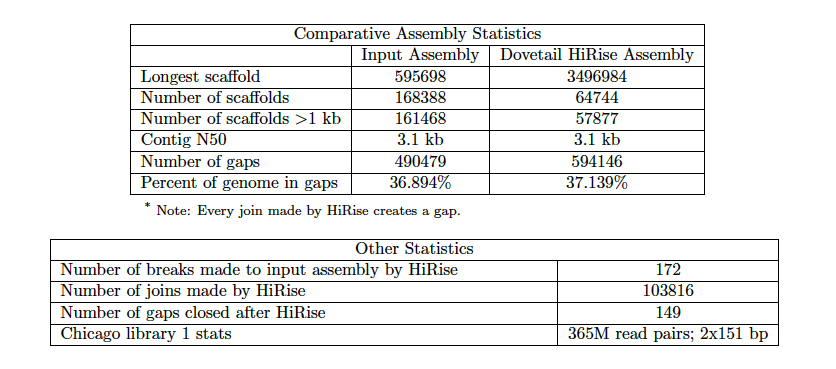


**1.3. Assembly statistics**

The *H. maculosa* genome assembly was assessed using assembly-stats (https://github.com/sanger-pathogens/assembly-stats), *O. bimaculoides* and *C. minor* assemblies were similarly examined.

Table 4. Assembly statistics for the three octopod genomes used in this study.

| **Species** | ***H. maculosa*** | ***C. minor*** | ***O. bimaculoides*** |
| --- | --- | --- | --- |
| total_length | 4009.6Mb | 5090.35Mb | 2371.52Mb |
| number | 48,285 | 41,584 | 379,696 |
| mean_length | 0.083040Mb | 0.122411Mb | 0.006245Mb |
| longest | 11.01Mb | 3.03Mb | 4.06Mb |
| shortest | 771bp | 4876bp | 56bp |
| Gaps | 4.32Mb | 0 | 0.56Mb |
| N50 | 0.93Mb | 0.29Mb | 0.47Mb |
| N50n | 1,044 | 3,696 | 1,369 |
| N70 | 0.44Mb | 0.2Mb | 0.25Mb |
| N70n | 2,299 | 6,735 | 2,776 |
| N90 | 0.12Mb | 0.09Mb | 0.07Mb |
| N90n | 5,607 | 11,789 | 6,239 |

**1.4 Genome heterozygosity estimate**

*JELLYFISH* was used in conjunction with GenomeScope^1^ to calculate heterozygosity in *H. maculosa* using a kmer frequency of 21.

Table 5. GenomeScope version 1.0 *H. maculosa* results.

| Property | Min | Max |
| --- | --- | --- |
| Heterozygosity | 0.92% | 0.97% |
| Genome haploid length | 5,141.97 Mb | 5,165.40 Mb |
| Genome repeat length | 1,840.19 Mb | 1,848.57 Mb |
| Genome unique length | 3,301.78 Mb | 3,316.82 Mb |
| Model fit | 95.23% | 99.35% |
| Read error rate | 0.48% | 0.48% |

Table 6. Heterozygosity for published molluscan genomes.

| Classification | Species | Heterozygosity | Publication |
| --- | --- | --- | --- |
| Cephalopod | *Hapalochlaena maculosa* | 0.98% | This study |
|  | *Octopus bimaculoides* | 0.08% | ^2^ |
|  | *Octopus vulgaris* | 1.10% | ^3^ |
| Gastropod | *Elysia chlorotica* | 3.66% | ^4^ |
|  | *Pomacea canaliculata* | 1-2% | ^5^ |
| Bivalve | *Pinctada fucata martensii* | 2.5-3% | ^6^ |
|  | *Saccostrea glomerata* | 0.51% | ^7^ |
|  | *Modiolus philippinarum* | 2.02% | ^8^ |
|  | *Bathymodiolus platifrons* | 1.24% | ^8^ |
|  | *Limnoperna fortunei* | 2.30% | ^9^ |
|  | *Chlamys farreri* | 1.40% | ^10^ |
|  | *Crassostrea gigas* | 0.73% | ^11^ |
|  | *Dreissena polymorpha* | 2.13% | ^12^ |

**1.5 Mutation rate**

Base neutral mutation rate was calculated between the *H. maculosa* and *O. bimaculoides* lineages with the assumption that the rate of mutations is equal to the rate of fixed differences between the two populations^13^. Orthologous genes from *O. bimaculoides* and *H. maculosa* were used. Neutrality was assumed for genes with very low expression (>10 TMP across all tissues). Neutral genes were aligned using *MAFFT^14^* and *codeml^15^* was used to calculate substitution metrics (dS). Per base neutral substitution between lineages was determined using the mean dS value divided by divergence time usually over number of generations, however *H. maculosa* is a single generation species. As octopus are diploid the rate was divided by two.

**1.6 Effective population size (PSMC)**

Historical changes in effective population size were estimated using Pairwise Sequentially Markovian Coalescent (PSMC) implemented in the software MSMC^16,17^. To generate inputs for MSMC we selected reads from libraries with short (500bp) insert sizes which provided 38x coverage of the genome. These were pre-processed according to GATK best practices; briefly, adapters were marked with Picard 2.2.1, reads were mapped to the *H. maculosa* genome using bwa mem (version 0.7.17) and PCR duplicates identified using Picard 2.2.1. In order to avoid inaccuracies due to poor coverage or ambiguous read mapping we masked regions where short reads would be unable to

find unique matches using SNPable (<http://lh3lh3.users.sourceforge.net/snpable.shtml>**)** and where coverage was more than double or less than half the genome wide average of 38x. Variant sites were called within unmasked regions and results converted to MSMC input format using msmc-tools<https://github.com/stschiff/msmc-tools>. All data for *H. maculosa* scaffolds of length greater than 1Mb was then used to generate 100 bootstrap replicates by dividing data into 500kb chunks and assembling them into 20 chromosomes with 100 chunks each. We then ran msmc2 on each bootstrap replicate and imported the resulting data into R for plotting. A mutation rate of 2.4e-9 per base per year and a generation time of 1 year were assumed in order to set a timescale in years and convert coalescence rates to effective population size.


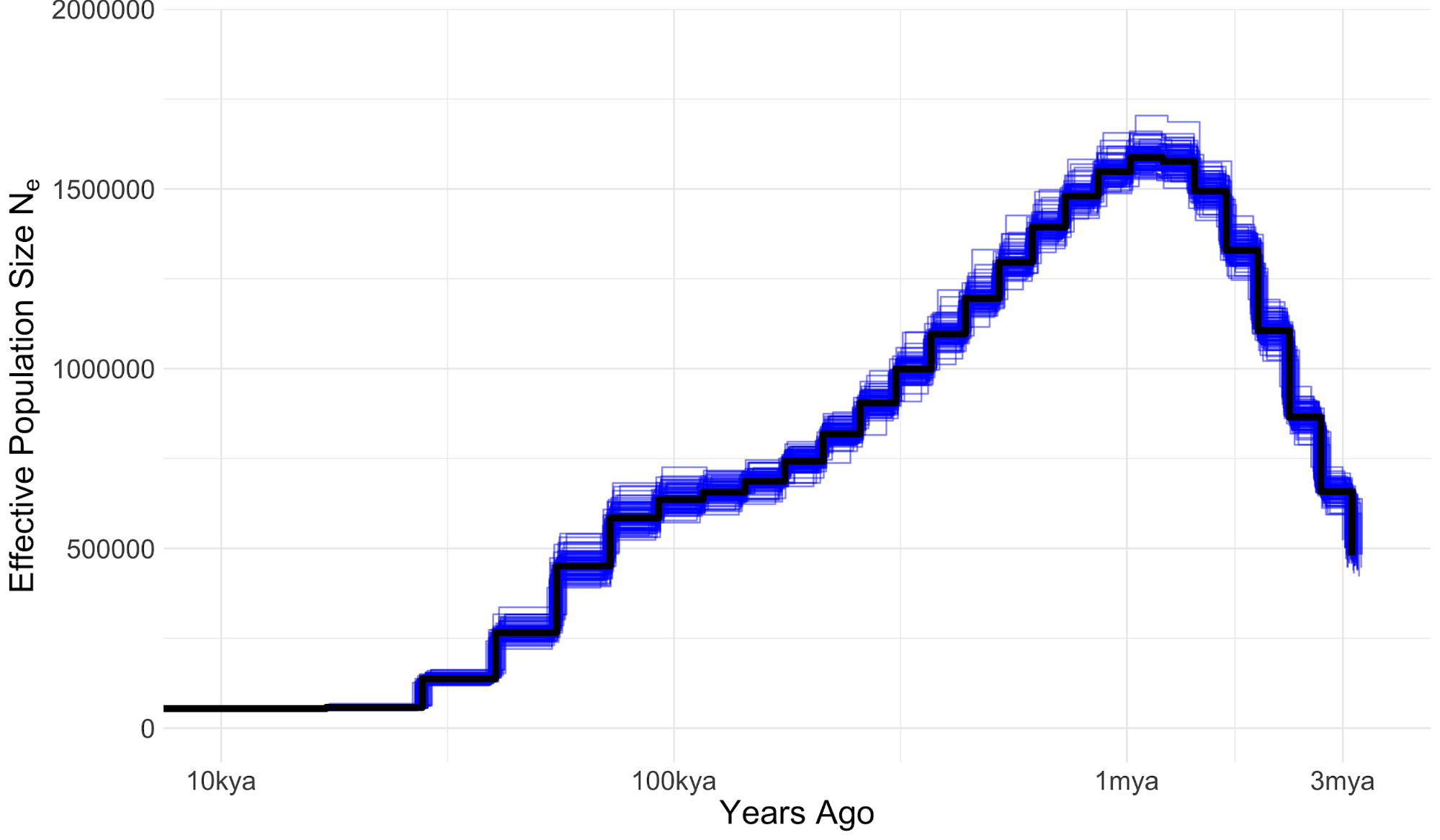


Figure 2. PSMC estimation of effective population size in *H. maculosa.*

**2. TRANSCRIPTOME SEQUENCING AND ANALYSIS**

**2.1. Tissues sampled, RNA preparation and sequencing**

Tissues were extracted from 12 tissues (brain, anterior salivary gland, digestive gland, renal, brachial heart, male reproductive tract, systemic heart, eyeballs, gills, posterior salivary gland, dorsal mantle and ventral mantle tissue) taken from a single *H. maculosa* individual collected at Beaumaris Sea Scout Boat Shed, Beaumaris, Port Phillip Bay, Australia (37°59'43.70"S 145°21.17"E). RNA was extracted using the Qiagen RNeasy mini kit according to the manufacturer's protocol. Extracted tissues were homogenised using a desktop homogeniser (IKA). Quality and quantity of samples was assessed using a Bioanalyser (nanochip)(Agilent). Sequencing was conducted on a single lane of an Illumina HiSeq2000, with each forming 1/12th of a lane. Libraries were constructed using 3 μg of RNA at a concentration of >100 ng/μL.

**2.2. Mapping reads to the genome for expression analysis**

Expression of gene models was determined using *kallisto^18^*, which performs pseudoalignments to determine the compatibility of transcripts. Transcripts used were filtered for low quality and adaptors removed prior to quantification using the *kallisto* package (https://pachterlab.github.io/kallisto/). Each tissue was examined separately, and abundances calculated in transcripts per million(TPM). Expression was calculated for all three octopus in this study using this method.

**2.3. *de novo* transcriptome assembly using Trinity**

*H. maculosa* transcriptome assembly and annotation is described in a previous

publication^19^. *De novo* assembly of the *H. maculosa* transcriptome was conducted using sequencing data from 11 tissues (brain, anterior salivary gland, digestive gland, renal, brachial heart, male reproductive tract, eyeballs, gills, posterior salivary gland, dorsal mantle and ventral mantle tissue) and Trinity (v10.11.201). Default parameters were used aside from kmer coverage, which was set to three to account for the large data volume. Protein coding sequences were identified using Trinotate^15^ and domains assigned by Interpro^20^.

**3. ANNOTATION OF TRANSPOSABLE ELEMENTS AND PROTEIN CODING GENES**

**3.1. Annotation of protein coding genes**

The first step in the annotation pipeline was identification of a training dataset suitable for training the *augustus* algorithm^21^. This data was derived using transcriptomic evidence via the *PASA* pipeline^22^. Transcripts were cleaned and filtered prior to mapping against the genome with both *BLAT^23^* and *GMAP^24^*. Validation of alignments required a percent identity of > 95% along 90% of the transcript length (default settings). ORFs were identified and extracted from the alignments using Transdecoder within the *PASA* package. ORF.gff extracted from *PASA* was converted to genbank format and split into a training subset( 3391 sequences) and a test subset (200 sequences). eTraining the augustus algorithm was performed using the training set of sequences and the results tested for sensitivity and specificity. The results were compared with gene prediction conducted using the gene sets for human and *Drosophila*. Optimization was then conducted using the *H. maculosa* training set for 12 rounds, after which the sensitivity and specificity were re-evaluated. Hint files were created for exons, introns and repeats to assist in gene model prediction by augustus^21^. Repeat hits were derived from the repeatmasker output, exons. Exon hints were identified by aligning transcripts against the genome and extracting alignments with a min identity of 92%, these results were filtered further using pslCDnaFilter to extract the best alignments (-localNearBest=0.005). Intron hints were extracted initially from *TRINITY* accepted_hits.bam output using bam2hints. Augustus was run using these preliminary hints and an exon-exon junction database generated from the output. The pipeline has been documented in the git:<https://github.com/blwhitelaw/BRO_annotation>.

Table 6. *H. maculosa* annotation statistics.

| ***H. maculosa* annotation** |  |
| --- | --- |
| Primary transcripts (augustus) | 22,530 |
| Primary  Transcripts (transcriptome based) | 6,798 |
| Alternative transcripts | 4,031 |
| total | 33,359 |
|  |  |
| **Primary transcript (augustus)** |  |
| Average number of exons | 6 |
| Median exon length | 116 |
| Median intron length | 3,671 |
|  |  |
| **Gene model support** |  |
| transcriptome support | 25,257 |
| Pfam annotation | 11,578 |
| PANTHER annotation | 13,219 |

**3.2 Annotation completeness (BUSCO)**

Completeness of the genome was estimated using BUSCO^25^, which identified 87.7% complete and 7.5% fragmented genes against the metazoan database of 978 groups (Table 7).

Table 7. *H. maculosa* assembly assessed for completeness against the

BUSCO Metazoan database.

| Complete BUSCOs (C) | 858 | 0.88% |
| --- | --- | --- |
| Complete and single-copy  BUSCOs (S) | 760 | 0.78% |
| Complete and duplicated  BUSCOs (D) | 98 | 0.10% |
| Fragmented BUSCOs (F) | 73 | 0.08% |
| Missing BUSCOs (M) | 47 | 0.05% |
| Total BUSCO groups searched | 978 |  |

**3.3 Transposable element annotation and expansions**

*Repratmodeller^26^* was used to create a *de novo* library of repeats for each of the three genomes (*H. maculosa, O. bimaculoides* and *C. minor*). Genomes were masked using the repeatmodeller library via *Repeatmasker^26^*.

| Elements |  | Number of elements | Length | percentage of sequence |
| --- | --- | --- | --- | --- |
| SINEs |  | 1968743 | 301771845bp | 7.53% |
|  | ALUs | 4 | 194bp | 0.00% |
|  | MIRs | 118334 | 18379936bp | 0.46% |
| LINEs |  | 1099904 | 339826118bp | 8.48% |
|  | LINE1 | 37733 | 10860451bp | 0.27% |
|  | LINE2 | 159621 | 18598760bp | 0.46% |
|  | L3/CR1 | 89080 | 38967232bp | 0.97% |
| LTR |  | 162516 | 39307347bp | 0.98% |
|  | ERVL | 42 | 2749bp | 0.00% |
|  | ERVL-MaLRs | 3 | 176bp | 0.00% |
|  | ERV_classI | 19710 | 1655988bp | 0.04% |
|  | ERV_classII | 3265 | 161438bp | 0.00% |
| DNA |  | 2901024 | 400984061bp | 10.00% |
|  | hAT-Charlie | 274907 | 40257595bp | 1.00% |
|  | TcMar-Tigger | 16417 | 4508191bp | 0.11% |
| Unclassified |  | 1606419 | 314382396bp | 7.84% |
|  |  |  |  |  |
| Total interspersed repeats |  | 34.82% | 1396271767bp | 34.82% |
| Small RNA |  | 343070 | 50368808bp | 1.26% |
| Satellites |  | 22693 | 5497063bp | 0.14% |
| Simple repeats |  | 2141863 | 107567262bp | 2.68% |
| Low complexity |  | 121842 | 8404129bp | 0.21% |

Table 8. Summary for *H. maculosa* repeat annotation.

**4. MULTI-GENE PHYLOGENY AND GENE FAMILY EXPANSION ANALYSES**

**4.1 Multi-gene cephalopod phylogeny and dating**

The cephalopod phylogeny was constructed using the genomes (*Aplysia californica^27^, Lottia gigantea, Crassostrea gigas, Octopus bimaculoides, Callistoctopus minor and Hapalochlaena maculosa*) and transcriptomes (*Octopus kaurna, Octopus vulgaris, Sepia officinalis^28^* and *Idiosepius notoides*) where genomes were not available. Of the transcriptomes used, *Octopus kaurna* and *Octopus vulgaris* were sequenced and assembled in house using the same method previously described (Supplementary 2.1 & 2.3 ). Proteome pairs were identified using BLASTp in a mutual-best-hit approach with *O. bimaculoides* acting as the reference. We grouped together proteins from separate proteomes belonging to the same gene to form a cluster. Clusters were aligned individually with *MUSCLE^29^* and gapless alignments were ascertained by trimming using *Gblocks* ^30^ with default parameters. A total of 2,108 clusters were obtained. Phylogenies were constructed using *RAxML v8.0^31^* and divergence times estimated by *Phylobayes v4.1^32^*. *RAxML v8.0^31^* was run using the GTR+G+I model ascertained from *JmodelTest v2.1.10*. using the cAIC criterion for 100 bootstraps. *Phylobayes* estimated divergence times under a strict clock with a mixture model of F81 + G with a burn-in of 10%. Calibrations were used as follows : divergence between *H. maculosa* and *E. scolopes* 275mya & divergence between *C. gigas* and *E. scolopes* 500mya. Two runs were performed and convergence verified using *bpcomp,* which confirmed a maximum difference of < 0.1 and *tracecomp,* which also indicated convergence with an effective sample size(EES*) of* > 200 for all parameters. Both programs used were from the *Phylobayes package.*


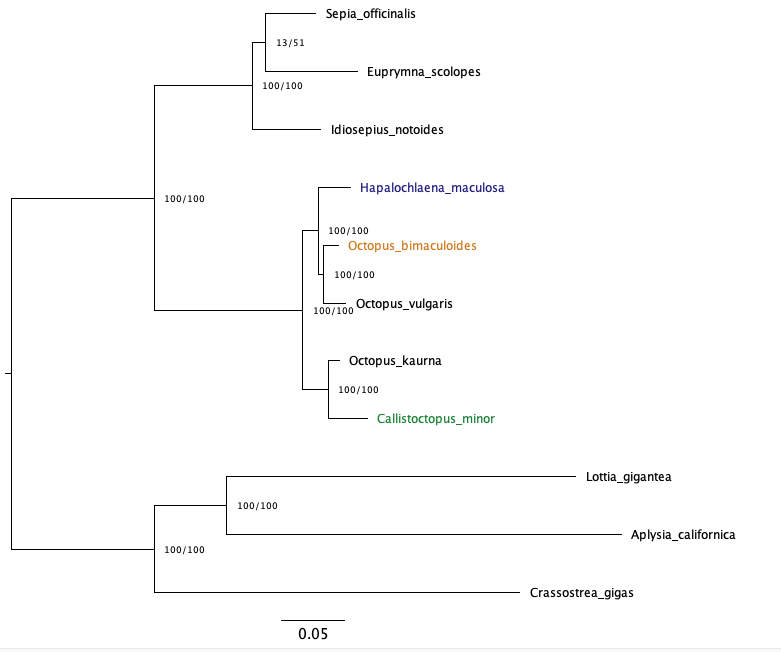


Figure 3. QI-TREE Maximum-likelihood tree.

**4.2 Genome-wide gene family expansions**

In order to examine genome-wide gene family expansions Pfam^31^ annotations were used to first categorize genes. Each gene was annotated using InterPro^32^ and annotations were filtered to remove duplicates among each gene, which may contain several copies of a single domain. A total of 5565 Pfams were identified among six molluscan genomes (*Aplysia californica, Lottia gigantea, Crassostrea gigas, Octopus bimaculoides, Callistoctopus minor and Hapalochlaena maculosa*). Significant expansions were identified by conducting an iterative Fisher's exact test in R^33^ on a counts table. Counts of Pfams were compared between a select group (octopods) and the background (average of remaining molluscs). In addition, each of the three octopods were tested against the background to identify species specific expansions. Expansions were deemed significant if a minimum of half of the species showed a significant p-value (0.01) for the Pfam.

**5. ANALYSIS OF NEURAL ASSOCIATED GENE FAMILIES**

**5.1 zinc finger C2H2**

The zinc-finger gene family was examined separately due to issues with de novo annotation using a single method i.e augustus. Zinc fingers were previously dismissed as repetitive and required additional annotation methods in the previous *O. bimaculoides* genome publication^1^.

Members of the family were extracted from gene models annotated with the following Pfams (PF13894, PF09337, PF13912, PF00096, PF12874). However, to capture zinc fingers missed in the initial gene annotation in *H. maculosa* we examined the transcriptome for zinc finger proteins and mapped them back to the genome to identify exons. This approach could not be used in *C. minor* as all transcripts had a corresponding gene model. As an alternative, exonerate was used to identify potentially missed zinc finger genes in the *C. minor* annotation. zinc fingers identified in the previously published *O. bimaculoides* genome were aligned against the *C. minor* genome and filtered to attain matches which were > 80% identity.

**5.2 Cadherin/Protocadherin**

The cadherin family was found to be more complete than the zinc fingers and additional annotation steps were not conducted as it was not necessary. Cadherin genes were identified using the Pfams (PF08266,PF00028, PF08374). In order to identify members of the subfamily, protocadherin domains genes were examined for the number of domains. Genes with between 4-7 domains were manually classified into the protocadherin family (Figure 4). A total of 77, 164 and 116 were identified for *H. maculosa*, *C. minor* and *O. bimaculoides,* respectively. Alignments were made for the cadherin and protocadherin families using MAFFT^14^ and Fastree^34^ was used to generate a phylogeny.


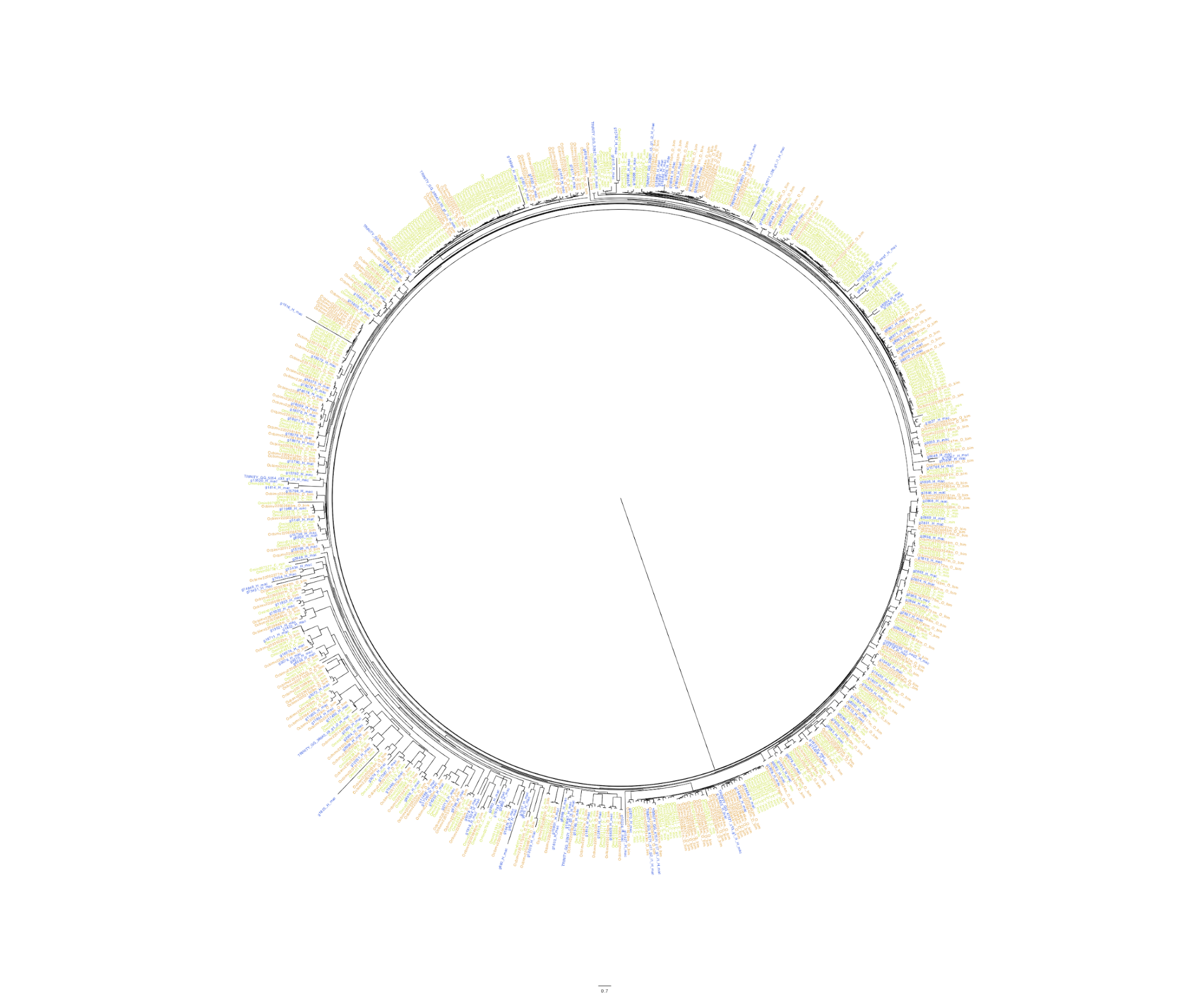


Figure 4. Phylogenetic tree of cadherins in *H. maculosa* (blue), *O. bimaculoides* (orange) and *C. minor* (green).


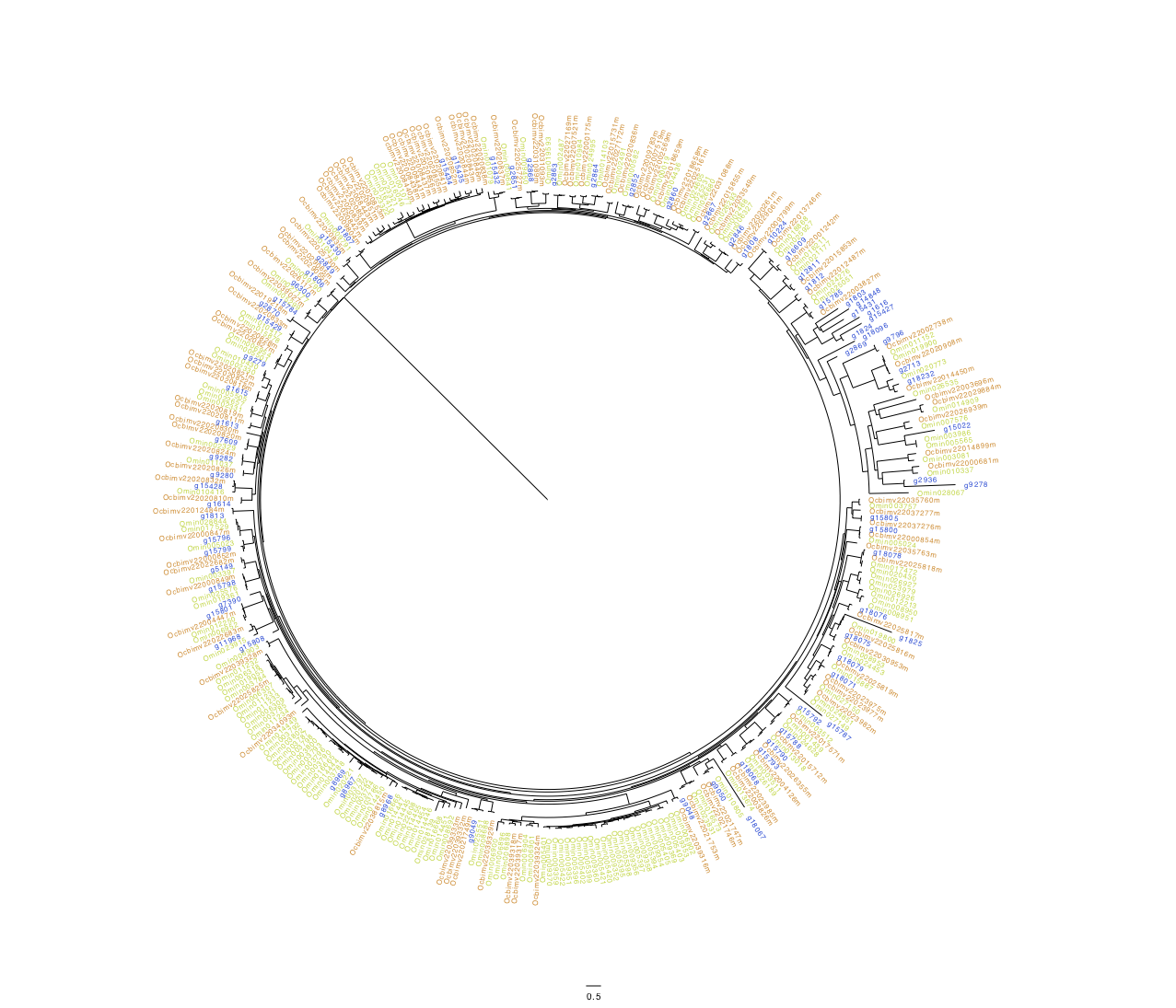


Figure 5. Phylogenetic tree of protocadherins in *H. maculosa* (blue), *O. bimaculoides* (orange) and *C. minor* (green).

**6. EVOLUTION OF THE VENOM/POSTERIOR SALIVARY GLAND (PSG) IN OCTOPODS**

**6.1 Extraction of tissue specific genes**

Specificity of gene expression was examined for all genes in *H. maculosa, C. minor* and *O. bimaculoides.* Expression of transcripts for each gene model within each tissue was calculated using Kallisto^18^. Expression was normalised prior to calculation of tau in R. A gene was deemed to be specifically expressed in a tissue if the tau value was > 0.8. This cutoff has been previously used by ^35,36^ to classify a gene as specifically expressed or promiscuous. Overall trends shows peaks at low tau/highly generalised expression and high tau/specifically expressed within one tissue.


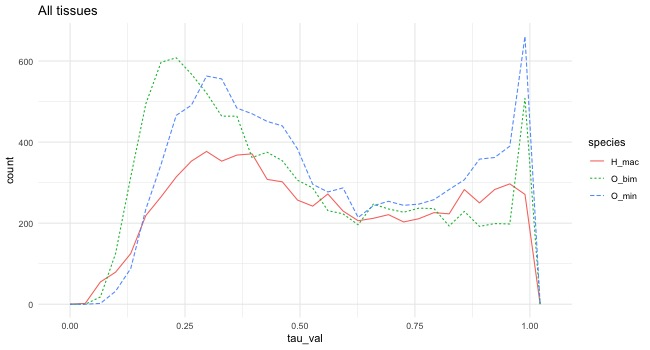


Figure 6. Distribution of tau values for genes in *H. maculosa, C. minor* and *O. bimaculoides.*

**6.2 Examination of gene families and orthology**

In order to identify orthologous groups between the three octopod genomes a combination of proteinortho and Orthovenn2^37^ was used. Tau values for each gene were calculated in R to determine their level of fidelity to a tissue (>0.8 = tissue specific) and Pfams used to categorise families. Specific tissues of interest shared between species (Brain and PSG) were then examined for their gene composition and expression.

**6.3 Gene loss, shift**

Patterns of gene expression, expansion and loss were examined between species. Using the defined orthologous groups, criteria were created to classify genes as shared/orthologous, specific to a species and loss of expression (expression must occur in one species and its ortholog unexpressed in another). The PSG was of particular interest in order to examine the impact of tetrodotoxin on PSG gene expression and composition. Genes with PSG specific expression in both *O. bimaculoides* and *C. minor* (non-TTX bearing) which had orthologs not specific to PSG in *H. maculosa* were examined. Additionally, the inverse of genes specific to the *H. maculosa* PSG and non-specific to the PSG of both *O. bimaculoides* and *C. minor* were compared.


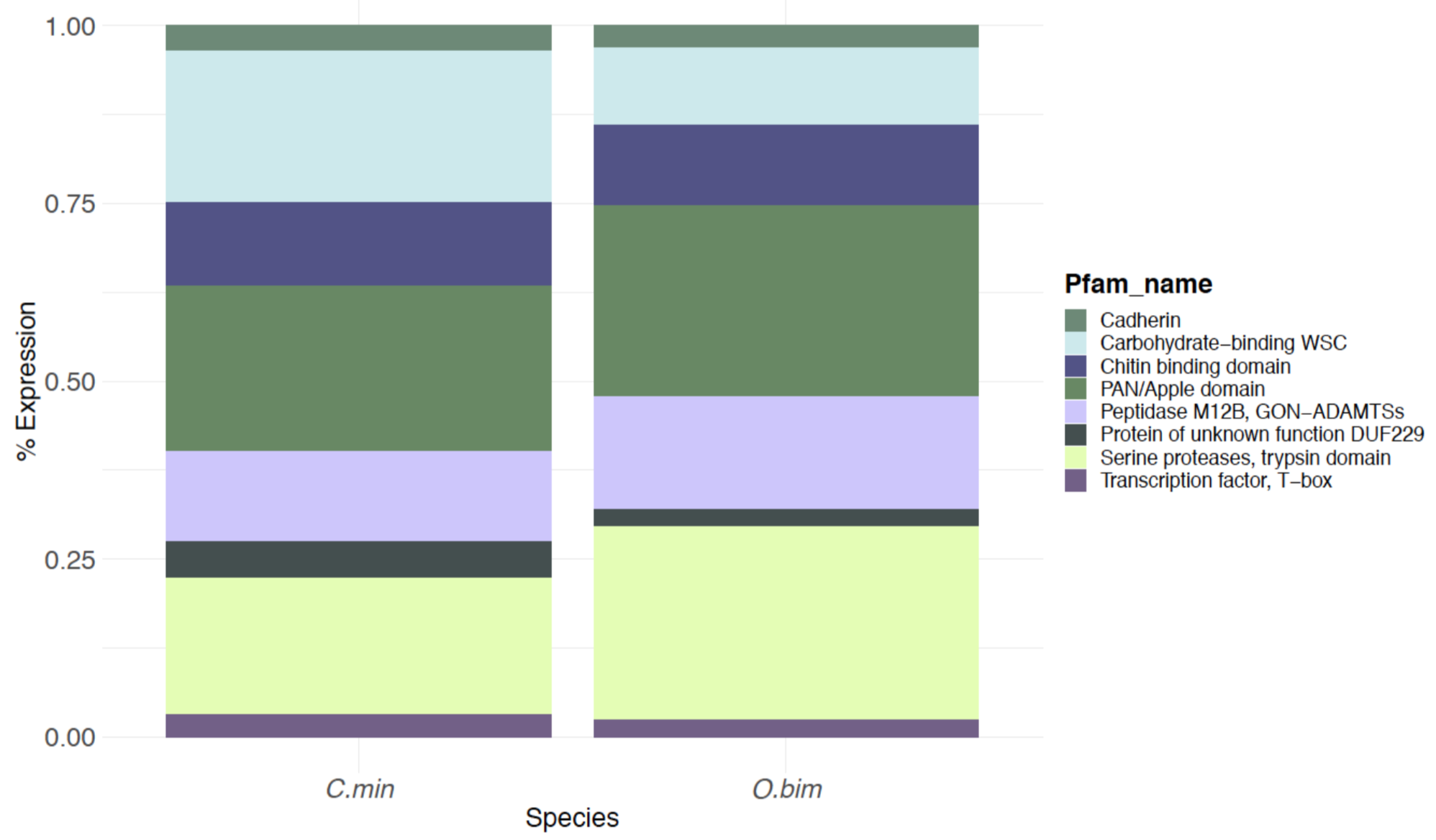


Figure 7. Orthologous genes specifically expressed in the PSG of *O. bimaculoides* and *C. minor* which have no ortholog in *H. maculosa*.

***6.4 Examination of selection and evolutionary rates in octopod serine proteases***

Gene models (aa) from the three octopod genomes (*H. maculosa, O. bimaculoides* and *C. minor*) were annotated with Interproscan and serine proteases with the Pfam PF00089 extracted for examination. Gene models and their corresponding CDS sequences were imported into *Geneious v10.2.6* and selected for a single trypsin (PF00089) domain greater than 200aa/600bp long. The region containing the trypsin domain was then extracted from the nucleic acid sequences and *MAFFT v7.407* was used to align sequences using Translation align in *Geneious v10.2.6*, which interpreted the first codon as the start of the codon region and used the first translation frame. The resulting alignment was tested for an appropriate substitution model in *jModelTest v2.2.10* and a tree was generated with *RAxML v8.0* using the GTR +G+I model and 100 bootstraps. The resulting tree and alignment were examined using codeml via *EasyCodeml v1.21* from the PAML package to examine non-synonymous to synonymous substitution rates for evidence of positive selection. We first used a site-based model which allows for *ω* values to vary between sites along the protein. Comparison of the nested models (M1a-M2a) and (M7-M8) did not reveal any sites under positive selection (p >0.05). In order to access the potential for different rates of evolution within specific lineages we used a branch site model which allows for *ω* values to vary between sites and branches. For the foreground a large clade of genes, majority of which were specifically expressed in the posterior salivary gland (PSG) was selected and compared to all other non-PSG specific genes. No sites among the foreground branches were significantly accelerated relative to the background. The last method implemented is similar to the branch site model, however, the rate along sites is constant and the rate between the background and foreground can differ. This also found no evidence of positive selection between the background and foreground lineages. It should be noted that serine proteases are a large and complex family and are due a more in-depth analysis in coleoid cephalopods, which could form a complete stand-alone study.

**7. EVOLUTION OF TETRODOTOXIN RESISTANCE IN *H. MACULOSA***

**7.1 Extraction and identification of Nav channels and p-loop regions**

Sodium channels (Na_v_) for the three octopus genomes along with all available in-house cephalopod transcriptomes were extracted manually using a series of BLAST searches against the nr database. Annotation was achieved using interproscan^20^ and identification and extraction of p-loop regions of the sodium channel alpha subunit were manually performed. Where sodium channels were incomplete alignment against related complete channels were used to extract the p-loop regions.

**7.2 Identification of TTX resistance mutation**

An assessment of current literature detailing the binding affinity of various mutations to the Na_v_ were used to assess TTX resistant mutations^38-44^. Two Na_v_ genes were identified in *H. maculosa* and found to contain mutations shared with the recently published *Hapalochlaena lunulata^39^*, which have potential to inhibit TTX binding and provide resistance. Three substitutions (M1406T, D1669H and H1670S) were identified in Na_v_1(Fig 8), the former of which is consistent with TTX resistance in pufferfish and garter snakes. A Met-Thr substitution in a TTX sensitive Na_v_1.4 rat channel was found to decreased binding affinity in pufferfish by 15-fold^41^. The latter two substitutions occur in the fourth p-loop at known TTX binding sites. Similar mutations at the same site in the turbellarian flatworm *Bdelloura candida*(BcNa_v_1)^41,45^ are predicted to inhibit TTX binding by preventing formation of a hydrogen bond^38^.

**7.3 Patterns of resistance and expression in the Nav gene family**

A total of two Nav genes were recovered for all three octopods (Na_v_1, Na_v_2), however Na_v_2 for *O. bimaculoides* and *C. minor* were incomplete. Function of the Na_v_2 gene remains unknown in cephalopods and is not believed to contribute to activation of the action potential^39^. However, expression data suggests it plays an important role in neural tissue with similar expression to Na_v_1 in the brain of all octopods species examined (Fig 10). Unlike the Nav1 gene Na_v_2 lacks the Met-Thr mutation believed to provide TTX resistance (Fig 9).


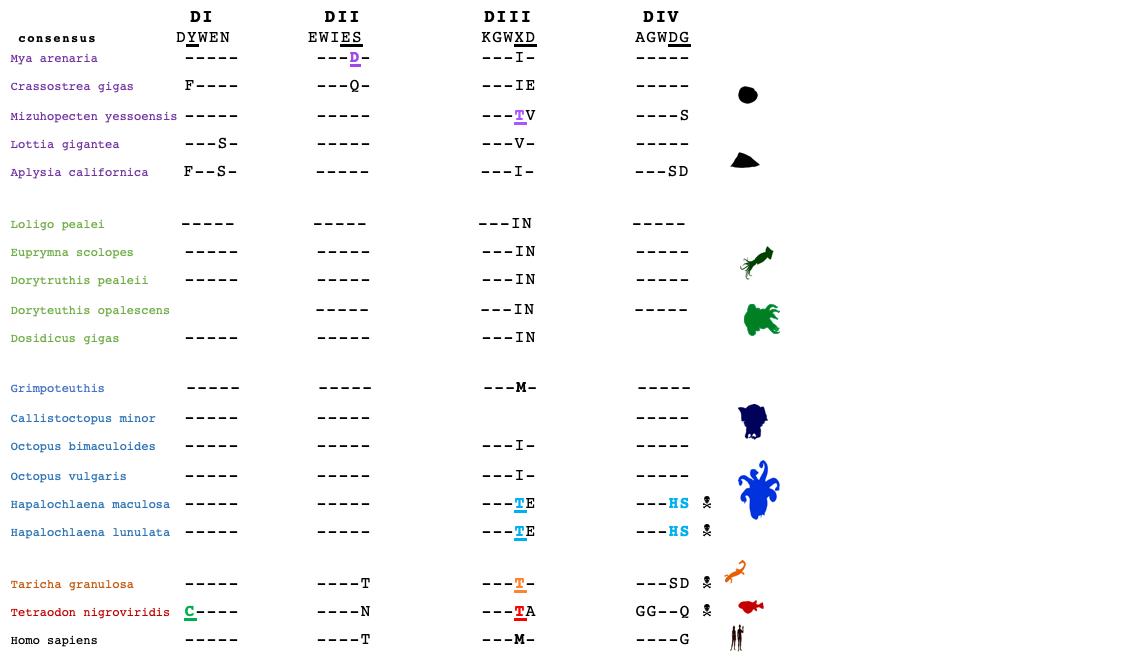


Figure 8. Alignment of Nav1 p-loop regions


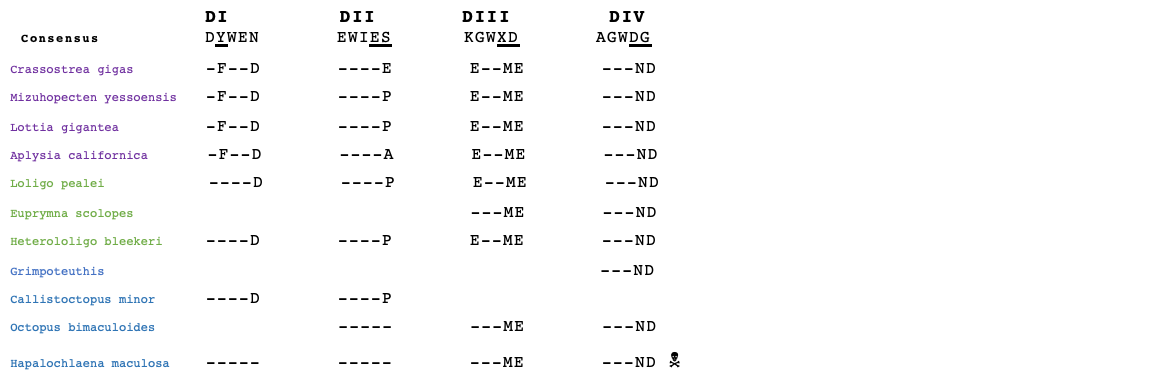


Figure 9. Alignment of Nav2 p-loop regions


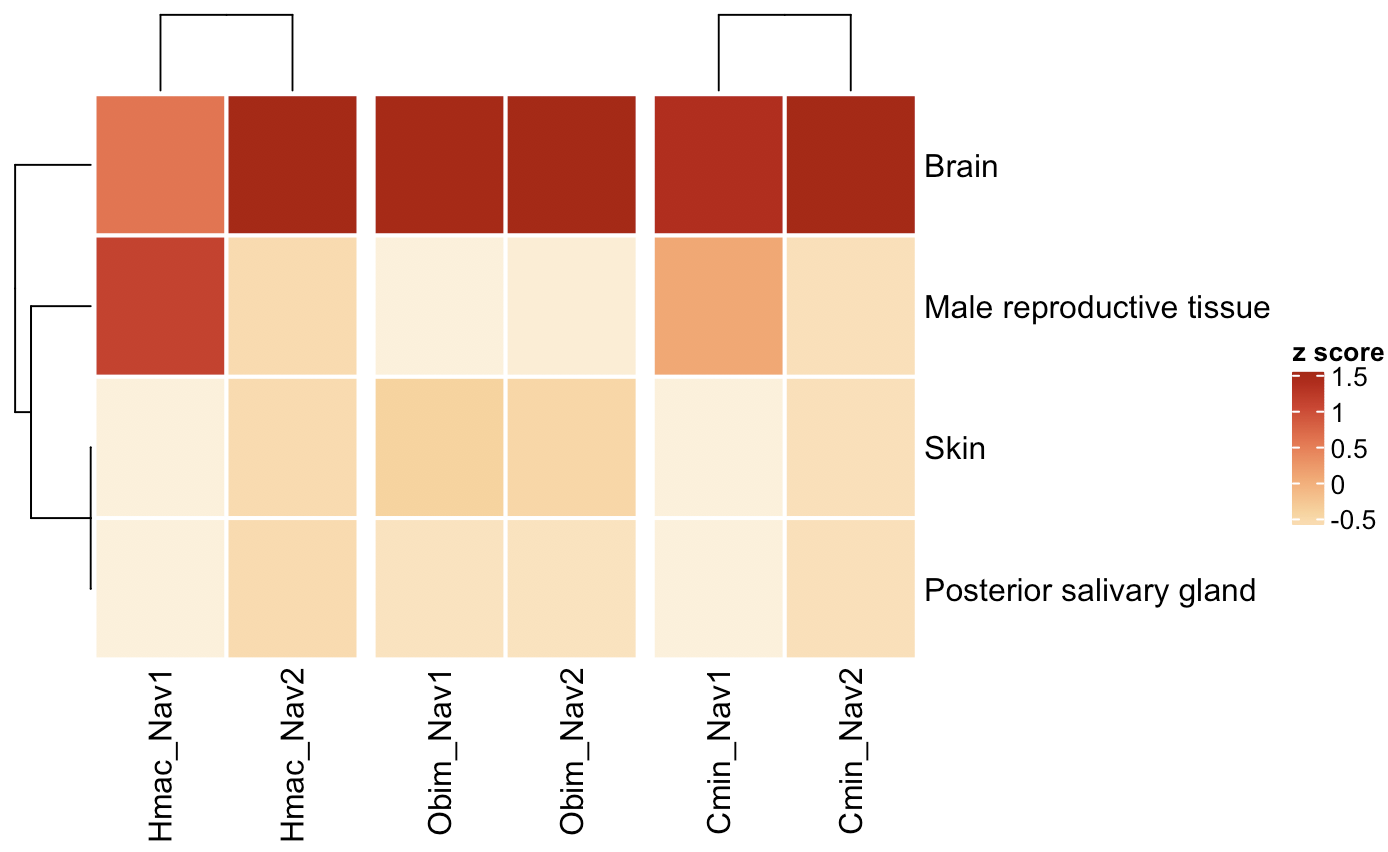


Figure 10. Expression of Nav1 and Nav2 channels across shared tissues of *H. maculosa* (Hmac), *O. bimaculoides* (Obim) and *C. minor* (Cmin).

**8. MICROBIOME OF THE *H. MACULOSA* POSTERIOR SALIVARY GLAND**

**8.1 SAMSA**

Bacterial composition in the PSG of *H. maculosa* was examined using a single ribo-depleted RNA sample. The SAMSA2 pipeline^46^ was used to identify and quantify bacterial species. Reads were trimmed and filtered using trimmomatic^47^ and sortMeRNA^48^ was used to remove ribosomal DNA. As the reads were paired end PEAR^49^ was used to merge reads prior to annotation. Annotation using DIAMOND in conjunction with the RefSeq(ftp://ftp.ncbi.nlm.nih.gov/refseq/release/complete/) and Subsystems(ftp://ftp.theseed.org/subsystems/) databases. Annotation and analyses using these databases was conducted in parallel. Analysis was limited by sample size however diversity indices were calculated in R using the SAMSA2 script “diversity_graphs_mod.R”.

**References**

1 Vurture, G. W. *et al.* GenomeScope: fast reference-free genome profiling from short reads. *Bioinformatics* **33**, 2202-2204 (2017).

2 Albertin, C. B. *et al.* The octopus genome and the evolution of cephalopod neural and morphological novelties. *Nature* **524**, 220-224, doi:10.1038/nature14668 (2015).

3 Zarrella, I. *et al.* The survey and reference assisted assembly of the Octopus vulgaris genome. *Scientific data* **6**, 13 (2019).

4 Cai, H. *et al.* A draft genome assembly of the solar-powered sea slug Elysia chlorotica. *Scientific data* **6**, 190022 (2019).

5 Liu, C. *et al.* The genome of the golden apple snail Pomacea canaliculata provides insight into stress tolerance and invasive adaptation. *GigaScience* **7**, giy101 (2018).

6 Du, X. *et al.* The pearl oyster Pinctada fucata martensii genome and multi-omic analyses provide insights into biomineralization. *GigaScience* **6**, gix059 (2017).

7 Powell, D. *et al.* The genome of the oyster Saccostrea offers insight into the environmental resilience of bivalves. *DNA Research* **25**, 655-665 (2018).

8 Sun, J. *et al.* Adaptation to deep-sea chemosynthetic environments as revealed by mussel genomes. *Nature ecology & evolution* **1**, 0121 (2017).

9 Uliano-Silva, M. *et al.* A hybrid-hierarchical genome assembly strategy to sequence the invasive golden mussel, Limnoperna fortunei. *GigaScience* **7**, gix128 (2017).

10 Jiao, W. *et al.* High-resolution linkage and quantitative trait locus mapping aided by genome survey sequencing: building up an integrative genomic framework for a bivalve mollusc. *DNA research* **21**, 85-101 (2013).

11 Zhang, G. *et al.* The oyster genome reveals stress adaptation and complexity of shell formation. *Nature* **490**, 49 (2012).

12 Garton, D. W. & Haag, W. R. Heterozygosity, shell length and metabolism in the European mussel, Dreissena polymorpha, from a recently established population in Lake Erie. *Comparative Biochemistry and Physiology Part A: Physiology* **99**, 45-48 (1991).

13 Kimura, M. Evolutionary rate at the molecular level. *Nature* **217**, 624-626 (1968).

14 Rozewicki, J., Li, S., Amada, K. M., Standley, D. M. & Katoh, K. MAFFT-DASH: integrated protein sequence and structural alignment. *Nucleic acids research* **47**, W5-W10 (2019).

15 Adachi, J. & Hasegawa, M. *MOLPHY version 2.3: programs for molecular phylogenetics based on maximum likelihood*. (Institute of Statistical Mathematics Tokyo, 1996).

16 Li, H. & Durbin, R. Inference of human population history from whole genome sequence of a single individual. *Nature* **475**, 493 (2012).

17 Schiffels, S. & Durbin, R. Inferring human population size and separation history from multiple genome sequences. *Nature genetics* **46**, 919 (2014).

18 Bray, N. L., Pimentel, H., Melsted, P. & Pachter, L. Near-optimal probabilistic RNA-seq quantification. *Nature biotechnology* **34**, 525-527 (2016).

19 Whitelaw, B. L. *et al.* Combined transcriptomic and proteomic analysis of the posterior salivary gland from the southern blue-ringed octopus and the southern sand octopus. *Journal of proteome research* **15**, 3284-3297 (2016).

20 Jones, P. *et al.* InterProScan 5: genome-scale protein function classification. *Bioinformatics* **30**, 1236-1240 (2014).

21 Stanke, M. *Gene prediction with a hidden Markov model*, University of Göttingen, (2004).

22 Haas, B. J. *et al.* Improving the Arabidopsis genome annotation using maximal transcript alignment assemblies. *Nucleic acids research* **31**, 5654-5666 (2003).

23 Kent, W. J. BLAT—the BLAST-like alignment tool. *Genome research* **12**, 656-664 (2002).

24 Wu, T. D. & Watanabe, C. K. GMAP: a genomic mapping and alignment program for mRNA and EST sequences. *Bioinformatics* **21**, 1859-1875 (2005).

25 Waterhouse, R. M. *et al.* BUSCO applications from quality assessments to gene prediction and phylogenomics. *Molecular biology and evolution* **35**, 543-548 (2017).

26 Smit, A., Hubley, R & Green, P. *RepeatMasker Open-4.0.*, <<<http://www.repeatmasker.org>>. > (2013-2015).

27 Moroz, L. L. *et al.* The ctenophore genome and the evolutionary origins of neural systems. *Nature* **510**, 109 (2014).

28 Bassaglia, Y. *et al.* ESTs library from embryonic stages reveals tubulin and reflectin diversity in Sepia officinalis (Mollusca—Cephalopoda). *Gene* **498**, 203-211 (2012).

29 Edgar, R. C. MUSCLE: a multiple sequence alignment method with reduced time and space complexity. *BMC bioinformatics* **5**, 113 (2004).

30 Talavera, G. & Castresana, J. Improvement of phylogenies after removing divergent and ambiguously aligned blocks from protein sequence alignments. *Systematic biology* **56**, 564-577 (2007).

31 Finn, R., Mistry, J., Tate, J., Coggill, P. & Heger, A. Pfam: the protein families database. Nuclei. Acids Re. (2014).

32 Finn, R. D. *et al.* InterPro in 2017—beyond protein family and domain annotations. *Nucleic Acids Research* **45**, D190-D199, doi:10.1093/nar/gkw1107 (2016).

33 Team, R. C. R: A language and environment for statistical computing. (2013).

34 Price, M. N., Dehal, P. S. & Arkin, A. P. FastTree: computing large minimum evolution trees with profiles instead of a distance matrix. *Molecular biology and evolution* **26**, 1641-1650 (2009).

35 Kryuchkova-Mostacci, N. & Robinson-Rechavi, M. A benchmark of gene expression tissue-specificity metrics. *Briefings in bioinformatics* **18**, 205-214 (2017).

36 Kryuchkova-Mostacci, N. & Robinson-Rechavi, M. Tissue-specificity of gene expression diverges slowly between orthologs, and rapidly between paralogs. *PLoS computational biology* **12**, e1005274 (2016).

37 Xu, L. *et al.* OrthoVenn2: a web server for whole-genome comparison and annotation of orthologous clusters across multiple species. *Nucleic acids research* **47**, W52-W58 (2019).

38 Geffeney, S. L., Fujimoto, E., Brodie III, E. D., Brodie Jr, E. D. & Ruben, P. C. Evolutionary diversification of TTX-resistant sodium channels in a predator–prey interaction. *Nature* **434**, 759 (2005).

39 Geffeney, S. L. *et al.* Convergent and parallel evolution in a voltage-gated sodium channel underlies TTX-resistance in the Greater Blue-ringed Octopus: Hapalochlaena lunulata. *Toxicon* (2019).

40 Ulbricht, W., Wagner, H. H. & Schmidtmayer, J. Kinetics of TTX‐STX Block of Sodium Channels. *Annals of the New York Academy of Sciences* **479**, 68-83 (1986).

41 Jost, M. C. *et al.* Toxin-resistant sodium channels: parallel adaptive evolution across a complete gene family. *Molecular biology and evolution* **25**, 1016-1024 (2008).

42 Du, Y., Nomura, Y., Liu, Z., Huang, Z. Y. & Dong, K. Functional expression of an arachnid sodium channel reveals residues responsible for tetrodotoxin resistance in invertebrate sodium channels. *Journal of Biological Chemistry* **284**, 33869-33875 (2009).

43 Feldman, C. R., Brodie, E. D. & Pfrender, M. E. Constraint shapes convergence in tetrodotoxin-resistant sodium channels of snakes. *Proceedings of the National Academy of Sciences* **109**, 4556-4561 (2012).

44 Frank, H. Y. & Catterall, W. A. Overview of the voltage-gated sodium channel family. *Genome biology* **4**, 207 (2003).

45 Jeziorski, M., Greenberg, R. & Anderson, P. Cloning of a putative voltage-gated sodium channel from the turbellarian flatworm Bdelloura candida. *Parasitology* **115**, 289-296 (1997).

46 Westreich, S. T., Korf, I., Mills, D. A. & Lemay, D. G. SAMSA: a comprehensive metatranscriptome analysis pipeline. *BMC bioinformatics* **17**, 399 (2016).

47 Bolger, A. M., Lohse, M. & Usadel, B. Trimmomatic: a flexible trimmer for Illumina sequence data. *Bioinformatics* **30**, 2114-2120 (2014).

48 Kopylova, E., Noé, L. & Touzet, H. SortMeRNA: fast and accurate filtering of ribosomal RNAs in metatranscriptomic data. *Bioinformatics* **28**, 3211-3217 (2012).

49 Zhang, J., Kobert, K., Flouri, T. & Stamatakis, A. PEAR: a fast and accurate Illumina Paired-End reAd mergeR. *Bioinformatics* **30**, 614-620 (2014).

52 Gao, F., Chen, C., Arab, D.A., Du, Z., He, Y. and Ho, S.Y., 2019. EasyCodeML: A visual tool for analysis of selection using CodeML. *Ecology and evolution*, *9*(7), pp.3891-3898.

53. Darriba, D., Taboada, G.L., Doallo, R. and Posada, D., 2012. jModelTest 2: more models, new heuristics and parallel computing. *Nature methods*, *9*(8), pp.772-772.
