# Supplementary material for: Adaptive venom evolution and toxicity in octopods is driven by extensive novel gene formation, expansion, and loss
Source: Gigascience. 2020 Nov 10;9(11):giaa120. doi: 10.1093/gigascience/giaa120 (PMC7656900; doi:10.1093/gigascience/giaa120)
Supplement: giaa120_Response_to_Reviewer_Comments_Original_Submission [file giaa120_response_to_reviewer_comments_original_submission.pdf]

Author's Response To Reviewer Comments

Close

Dear Dr. Hongling Zhou

On behalf of my co-authors, I would like to thank you for the opportunity to revise and resubmit our research piece titled “Adaptive venom evolution and toxicity in octopods is driven by extensive novel gene formation, expansion and loss” (GIGA-D-20-00135).

We would also like thank all three reviewers for their constructive criticism and advice on how to improve our manuscript. All suggestions have been taken into account and incorporated resulting in a much-improved manuscript.

A response to reviewers has been provided in which we address each point providing the original text followed by the revised text, including line numbers where changes have been implemented. We also included a low resolution version of all corrected figures in the response.

Thank you again for your consideration of our revised manuscript

Yours sincerely,
Brooke L. Whitelaw

Reviewer reports:

Reviewer #1: The manuscript describes the genome of the southern blue-ringed octopus and provides comparisons between this genome and previously published octopus genomes, with a particular focus on venom. I did not see any major flaws in the paper, which I think will be a valuable contribution to the cephalopod genomics literature. The blue-ringed octopuses are of major interest due to their highly toxic venom, and I found the comparisons between this species and others in terms of venom production and resistance to be quite illuminating (though somewhat unsurprising, given what we already knew about the use of TTX by this species).

I found the paper to be fairly well written, though I do have several questions and comments that I hope will help clarify some issues. I will list those below, in the order I encountered them as I read the manuscript and supplementary materials. My only somewhat substantive concern first struck me as I read page 17 of the manuscript: "…suggesting a species-specific expansion of this cluster in C. minor". I think the authors should be a bit more careful with how they use the phrase "species-specific". They have included only three octopod species out of 300+ species in this study. Yes, any differences they detect between these species could be species specific, but I think it is more likely that the differences arose in ancestral lineages. For example, expansion of the serine protease cluster may have occurred only in C. minor, but it could also have occurred in the ancestor of Callistoctopus, or in some other ancestor. At present, the authors do not have sufficient sampling to know if any of the expansions, losses, shifts in expression, etc., they are seeing are truly species specific. Similarly, sentences like "Loss of serine protease genes can also be observed in H. maculosa". The authors can certainly state that H. maculosa has fewer serine protease genes than O. bimaculoides and C. minor, but the *loss* of these genes may have occurred in H. maculosa *or* in any ancestral lineage after the divergence of Hapalochlaena from Octopus. I urge the authors to go through their manuscript carefully to find instances where they have evidence of differences among these species and to check that their descriptions of differences among these species are clear.

We agree with the reviewer and have carefully read through the manuscript and corrected sections where species-specific inferences were made to prevent miscommunication of the findings.

Original text:
“Loss of serine protease genes can also be observed in H. maculosa”

Revised text: (pg. 18, line: 315)
“Fewer serine protease genes can also be observed in H. maculosa”

Original text:
“The greatest proportion of genes in each species examined were not specific to octopods or an octopus species (ancient genes) (Fig 2a). Expression of these genes were enriched in neural tissues across all species, indicating the core conservation of neural development and function. However, we also find that genes specific to each octopod species also show this expression pattern”

Revised text: (pg. 13-14 , lines: 231-235)
“The greatest proportion of genes in each species examined were not specific to octopods or an octopus lineage (ancient genes) (Fig 2a). Expression of these genes were enriched in neural tissues across all species, indicating the core conservation of neural development and function. However, we also find that genes specific to each octopod lineage also show this expression pattern”

Original text:
“Absence of gene expression for genes whose orthologs have retained expression in one or more other species suggests a unique evolutionary trajectory from other octopods. It should be noted that differences in tissue sampling may in part influence these values.”

Revised text: (pg. 14, lines: 244-249)
“Absence of gene expression for genes whose orthologs have retained expression in one or more other species suggests a unique evolutionary trajectory from other octopods. It should be noted that differences in tissue sampling may in part influence these values and due to the limited sampling of species, loss of expression cannot be inferred at a species level and may have occurred at any point in the lineage.”

We have also removed the term ‘species-specific’ and replaced it with the more accurate term ‘lineage-specific’ to avoid confusion. : (pg. 2, line: 45) , (pg. 10, line: 178), (pg. 13, line:229, (pg. 13, line: 224), (pg. 14, line: 239), (pg. 15, line: 255), (pg. 18, line: 314), (pg. 34, line: 589) & (pg. 35, line: 601)

Figure 2 has also been corrected to replace ‘species specific’ with ‘lineage specific’.
Low quality version shown here. The corrected version has been uploaded to replace the original

Minor points and suggestions

Check for subject-verb agreement in the abstract. For example, it should be "This diverse group of specilised (sic) predators has evolved…" (the subject is "group", not "predators").

We have corrected the spelling of ‘specialised’ and have replaced ‘have’ with ‘has’ (pg 1, lines: 24).

Abstract: Last sentence might be better as "This genome, along with other recently published cephalopod genomes, represents a valuable resource from which future work could advance our understanding of the evolution of genomic novelty in this family"

We agree that this sentence would fit better at the end of the abstract and have corrected this.

Original sentence:
“This genome along with other recently published cephalopod genomes represent a valuable resource from which future work could advance the evolution of genomic novelty within the family.‟

Revised sentence (pg. 2, lines: 45-48):
"This genome, along with other recently published cephalopod genomes, represents a valuable resource from which future work could advance our understanding of the evolution of genomic novelty in this family"

Pg. 3: "underrepresented" - Underrepresented in what sense? In terms of genomic resources?

When we used the term underrepresented, we were referring to the lack of published genomes for cephalopods. This has been slowly changing since the publication of the first cephalopod genome in 2015 by Albertin et al. The sentence has been modified to clarify this.

Original sentence:
“The recent publication of octopod genomes provides an opportunity to investigate the link between genomic and organismal evolution in this unique and underrepresented lineage”

Revised sentence: (pg. 3 ,lines: 52-55).
“The recent publication of octopod genomes provides an opportunity to investigate the link between genomic and organismal evolution in this unique lineage for which genomic resources have been lacking.1”

Pg. 3: (FAO,) - Looks like a typo? Or incomplete switch to a different citation format?

Corrected (pg. 3 ,line: 57)

Pg. 3: "soft bodied" should be "soft-bodied"

Corrected (pg. 3 ,line: 58)

Pg. 3: Should be "that are well adapted" (no hyphen)

Corrected (pg. 3 ,line: 58)

Pg. 4: "How resistance to TTX" - This is not totally clear as written. Resistance in what? I think the authors are referring to how Hapalochlaena avoids being killed by its own TTX, but this could be rephrased to make it crystal clear. Also "remains a large unknown" is a little awkward…maybe "remains largely unknown" would be better?

We agree that this sentence was unclear. Reviewer 3 also suggested that due the recent publication by Geffeney et al (2019) this sentence was no longer reflective of the current literature. In order to correct this we have updated this sentence to reflect the latest literature and taken care to ensure the sentences are clear.

Original sentence:
“How resistance to TTX has been acquired at the genetic level, remains a large unknown, with TTX resistance studied in only in a few select species (i.e. pufferfish13, newts14,15 and gastropods16).”

Revised sentences (pg. 4, lines: 74-79):
“The mechanism of TTX resistance, which allows for safe sequestration of TTX, has been attributed to several substitutions in the p-loop regions of voltage-gated sodium channels(Nav) in H. lunulata24. However, these channels have yet to be examined in H. maculosa and H. fasciata. TTX resistance has also been studied in a range of other genera including, pufferfish25, newts26,27 arachnids28, snakes29 and gastropods30”

Pg. 4: "Primarily used for defense…" - Awkward sentence with a dangling modifier, which makes it read as though Hapalochlaena is primarily used for defense in other species.

We agree that this was a poor choice of wording and this sentence has been clarified as follows:

Original sentence:
“Primarily used for defense in other unrelated TTX-bearing species, Hapalochlaena is the only known taxa to utilise TTX in venom”

Revised sentence (pg. 4-5,lines: 82-84):
“While other unrelated TTX-bearing species primarily use TTX for defense, Hapalochlaena is the only known taxa to utilise TTX in venom23,35.”

Pg. 5: "for example at the evolution of venoms" - Somewhat awkward, I think?

We agree and have modified the sentence to improve flow as follows:

Original sentence:
"By using a comparative genomic approach we are able to examine the emergence of octopod novelties, for example at the evolution of venoms, at a molecular level between H. maculosa and the two non-TTX bearing octopods: the California two-spot octopus (O. bimaculoides) and the long-armed octopus (Callistoctopus minor), while also addressing the species-specific evolution of tetrodotoxin acquisition and resistance in H. maculosa"

Revised sentence (pg. 5 ,lines: 93-98):
"By using a comparative genomic approach we are able to examine the emergence of octopod novelties, at a molecular level between H. maculosa and the two non-TTX bearing octopods: the California two-spot octopus (O. bimaculoides) and the long-armed octopus (Callistoctopus minor). We also address unique features of venom evolution in octopods while also addressing the species-specific evolution of tetrodotoxin acquisition and resistance in H. maculosa"

Pg. 6: Capitalize "bay"? (Port Phillip Bay)

Corrected (pg. 6, line: 108)

Pg. 7: Should be "shallow-water marine organisms".

Corrected (pg. 8, lines: 139)

Pg. 8: Should be "Southern Hemisphere".

Corrected (pg. 8, line: 142-143)

Pg. 8: Also here, how did they do their divergence time estimation?

Tanner et al. (2017) used a Bayesian approach to estimate divergence times, analyzing a concatenated alignment of 197 genes with Phylobayes. These details have been incorporated into the manuscript.

Original sentence:
"Previous phylogenies using a combination of a small number of mitochondrial and nuclear genes46-48 and orthologs derived from transcriptomes49 support this topology. Likewise, divergence of the H. maculosa from Abdopus aculeatus has been previously estimated to be ~59 mya2"

Modified sentence (pg. 9, lines: 151-155) :
"Previous phylogenies using a combination of a small number of mitochondrial and nuclear genes49–51 and orthologs derived from transcriptomes52 support this topology. Likewise, estimates by Tanner et al.2, using a concatenated alignment of 197 genes with a Bayesian approach, placed divergence of H. maculosa from Abdopus aculeatus at ~59 mya2."

Pg. 8: "maculosa from Abdopus" - "from" should not be italicized.

Corrected (pg. 9, line: 154)

Pg. 9: "sporadic occurrence" - I think the authors mean sporadic taxonomically here (i.e., some species have them, some do not), but this should be clarified (surely the authors don't mean that sometimes a given species has them and sometimes they don't!).

In order to prevent confusion the sentence has been modified to include the term "taxonomically sporadic".

Original sentence
"Morphological variations of ocelli structure and colour, along with their sporadic occurrence within Octopus and Amphioctopus3, limits our interpretation as to the evolutionary history of this trait in octopods"

Revised sentence: (pg. 9, lines: 160-163):

“Morphological variations of ocelli structure and colour, in conjunction with the taxonomically sporadic occurrence of this trait across species within Octopus and Amphioctopus, limits our interpretation as to the evolutionary history of this trait in octopods.”

Pg. 11: Just a suggestion here: "splicing, embryonic and neural development" is clear, but it looks odd. How about "splicing and embryonic and neural development" or "splicing as well as embryonic and neural development".

We have modified the sentence as suggested:

Original sentence:
“H. maculosa also shows expansions in the C2H2-type zinc finger family. Zinc fingers form an ancient family of transcription factors, which among other roles serve to regulate transposon splicing, embryonic and neural development 45,46.”

Revised sentence: (pg. 12 ,lines: 207-09)
“H. maculosa also shows expansions in the C2H2-type zinc finger family. Zinc fingers form an ancient family of transcription factors, which among other roles serve to regulate transposon splicing as well as embryonic and neural development69,70.”

Pg. 11: Should be "this type of zinc finger in O. bimaculoides"

Corrected (pg. 12, line: 210)

Pg. 12: "High level examination" should be "High-level examination", "large scale expression patterns" should be "large-scale expression patterns", and "lineage specific loss" should be "lineage-specific loss".

Corrected (pg. 13, lines: 227 & 230)

Pg. 12: Unnecessary comma in "we also find that, genes specific to each octopod"

Corrected (pg.13, line: 234)

Pg. 13: "in tandem with overall reduction in genes number relative to the octopods" - This is unclear and poorly worded. I assume this is referring still to H. maculosa relative to other octopods?

In order to improve the clarity and wording of this sentence it has been rephrased as follows:

Original sentence:
“In order to understand the implications of gene expression loss, in tandem with overall reduction in genes number relative to the octopods, further investigation is required.”

Revised sentence: (pg. 14, lines: 249-251)
“In order to fully understand the implications of the gene family contractions and loss of expression in H. maculosa, relative to other octopods, further investigation is required.”

Pg. 15: Unnecessary comma in "More notable, were differences"

Corrected (pg. 17, line: 288)

Pg. 16: Should be "primary venom-producing gland".

Corrected (pg. 17, line: 296)

Pg. 16: Haplochlaena should be italicized in "hypothesized that the Hapalochlaena PSF…" Also, who has hypothesized that the Hapalochlaena PSF will exhibit a loss of redundant proteinaceous toxins? This seems to call for a citation.

This statement was poorly worded and has been corrected to reflect that we proposed the hypothesis.

Hapalochlaena has also been italicized.

Original sentence:
"It has been hypothesized that the Hapalochlaena PSG will exhibit a loss of redundant proteinaceous toxins due to the presence of TTX."

Revised sentence: (pg. 17, lines: 299-300)
"We hypothesize that the Hapalochlaena PSG will exhibit a loss of redundant proteinaceous toxins due to the presence of TTX."

Pg. 16: "A total of 623 genes were exclusive to H. maculosa PSF…exclusive to O. bimaculoides and C. minor, respectively". Should this be "exclusive to the O. bimaculoides and C. minor PSGs, respectively"?

Yes the reviewer is correct. This sentence has been corrected as suggested.

Original sentence:
"A total of 623 genes were exclusive to H. maculosa PSG compared to only 230 and 164 exclusive to O. bimaculoides and C. minor, respectively"

Revised sentence (pg.17-18, lines 302-304)
"A total of 623 genes were exclusive to H. maculosa PSG compared to only 230 and 164 exclusive to O. bimaculoides and C. minor PSGs, respectively."

Pg. 16: "Additionally, H. maculosa PSG is predicted to be" - Predicted by whom? The authors? I think so, so they should make that clear, e.g., "we predict that the H. maculosa PSG is functionally more diverse…"

This sentence has been corrected as suggested:

Revised sentence (pg. 18, lines: 304-306)
"Additionally, we predict that the H. maculosa PSG is functionally more diverse based on the number of Pfam families detected, 532 in total."

Pg. 17: The sentence about reprolysin doesn't make sense to me. The authors describe shifting expression in this species (see my comment above) but then note that there is a complete loss of orthologs from the genome. This latter comment suggests that reprolysin doesn't even exist in the H. maculosa genome, but it must, if it is showing different expression patterns than the other species. Can the authors clarify this?

The sentence that "there is a complete loss of orthologs from the genome" was incorrect. It should have read "there is a complete loss of paralogs from the genome". This has been corrected in the sentence below.

Revised sentence (pg. 18, lines: 316-318)
"Similarly, reprolysin (M12B) exhibits shifting expression in H. maculosa, presumably from the PSG to the branchial heart, and a complete loss of paralogs from the genome."

Pg. 18: "the cephalopod specific clade" should be "cephalopod-specific clade".

Corrected (pg. 19 ,line: 324)

Pg. 18: "…hyaluronidase, which often serve as dispersal factors" seems odd. Should this be "hyaluronidase, which often serves as a dispersal factor"?

Yes and we have modified the sentence as suggested.

Original sentence:
"Previous proteomic analysis of the H. maculosa PSG revealed high expression of hyaluronidase, which often serve as dispersal factors within snake venom, facilitating the spread of toxin while not being directly toxic to their prey9,72"

Revised sentence: (pg. 19, lines: 330-333)

“Previous proteomic analysis of the H. maculosa PSG revealed high expression of hyaluronidase, which often serves as a dispersal factor within snake venom, facilitating the spread of toxin while not being directly toxic to their prey9,72.”

Pg. 19: "Two Nav genes"…should the "v" be a subscript here?

Corrected (pg. 20, line: 352)

Pg. 19: "latter regions in DIII and DIV" - Is "latter" the best adjective here?

“Latter” is not an ideal word choice and the sentence has been modified.

Original sentence:
“The latter regions in DIII and DIV”

Revised sentence: (pg. 21, line: 356)
“The regions DIII and DIV closer to the C-terminal end of the protein”

Pg. 20: "In previous studies, when examined individually, the Met- Thr substitution in a TTX sensitive Nav1.4 rat channel decreased binding affinity in pufferfish by 15-fold" - This could be more clear. A "rat channel"? As in, a channel in rats? But the sentence says "in pufferfish". Please clarify this.

Jost et al 2008 found a Met-Thr substitution in the third p-loop region of a pufferfish sodium channel. This substitution was induced in a TTX sensitive rat channel through site-directed mutagenesis and the rat channel was then expressed in the oocytes of the African clawed frog (Xenopus). In our original sentence some of these taxonomical/methodological details obscured the ultimate finding which is the introduction of a Met-Thr substitution, in an otherwise TTX-sensitive channel, inhibits TTX binding by 15-fold. Our revised sentence has removed reference to taxonomy to convey the main finding more clearly.

Original sentence:
"In previous studies, when examined individually, the Met- Thr substitution in a TTX sensitive Nav1.4 rat channel decreased binding affinity in pufferfish by 15-fold"

Revised sentence (pg. 21,lines: 365-366):
“In a previous study a Met to Thr substitution into a TTX sensitive Nav1.4 channel decreased binding affinity to TTX by 15-fold87.”

Pg. 21: "It has yet to be established if these mutations are derived from a shared ancestor or have occurred independently" - Excellent. This is exactly the clarity I think the authors can bring to their other statements about gains, losses, etc. that I pointed out in a previous comment.

As detailed in the first response to reviewers we have clarified these statements throughout.

Pg. 21: I would write "While Hapalochlaena remains" here.

Corrected (pg. 22 ,line: 379)

Pg. 21: Should be "STX-contaminated bivalves" (and "STX-contaminated fish" and "STX-contaminated food sources" below…and "TTX-producing bacteria" and "TTX-producing strains" on pg. 23).

Corrected (pg. 22-24, lines: 383, 391-392, 394, 416, 422)

Pg. 21: "Humboldt" should be capitalized, as it is a proper name.

Corrected (pg. 18, line: 389, 391)

Pg. 23: "Sequestration of TTX is not exclusive to the blue-ringed octopus among molluscs. Gastropods such as Pleurobranchaea maculata and Niotha clathrata, as well as some bivalves, are capable of sequestering the similar toxin STX " - TTX and STX are similar, but not the same. Are there other examples of actual TTX sequestration in molluscs, outside of Hapalochlaena? If not, this should be rephrased.

Yes, sequestration of TTX does occur in molluscs aside from Hapalochlaena, including but not necessarily limited to the gastropods Pleurobranchaea maculata and Niotha clathrata. In the original sentence we mistakenly said these species were capable of sequestering STX as opposed to TTX. This has been corrected in the revised sentence.

Original sentence:
“Gastropods such as Pleurobranchaea maculata and Niotha clathrata, as well as some bivalves, are capable of sequestering the similar toxin STX”

Revised sentence: (pg. 24 ,lines: 409-420)
“Gastropods such as Pleurobranchaea maculata and Niotha clathrata, as well as some bivalves, are also capable of sequestering TTX”

Pg. 23: "highly diverse composition of genera" may be better as "highly diverse composition of bacterial genera"

Corrected (pg. 24, lines: 413)

Pg. 23: "Diversity of bacterial genera much like the H. maculosa in this study was high" - This seems awkward and unclear."

This sentence was unclear and has been modified to:

Original sentence:
“Diversity of bacterial genera much like the H. maculosa in this study was high and this may complicate identification of species responsible for TTX production”

Revised sentence (pg. 25 , lines: 423-425):
“Congruent with our findings the diversity of bacterial genera was high and this may complicate identification of species responsible for TTX production”

Pg. 24: "TTX bearing mollusk genome" - Hmm…does the genome bear TTX? The mollusk does, I suppose, but it's produced by bacteria as described above. This could be rephrased.

The sentence has been rephrased to improve clarity to:

Original sentence:
“This work describes the genome of a unique TTX bearing mollusc genome, the southern blue-ringed octopus (Hapalochlaena maculosa).”

Revised sentence (pg. 25 ,lines: 434-435):
“This work describes the genome of a unique TTX bearing mollusc, the southern blue-ringed octopus (Hapalochlaena maculosa).”

Pg. 28: What kit or method was used to construct the cDNA libraries for transcriptome sequencing?

Construction of cDNA libraries was outsourced to AGRF (Australian Genome Research Facility), Melbourne, and conducted using their TruSeq mRNA Library Prep with polyA selection and unique dual indexing method. This information has been included in the methods section “Transcriptome sequencing”

Revised text: (pg. 29, lines: 499-504)
“Construction of cDNA libraries was outsourced to AGRF (Australian Genome Research Facility), Melbourne and conducted using their TruSeq mRNA Library Prep with polyA selection and unique dual indexing method. Libraries were constructed using 3 µg of RNA at a concentration of >100 ng/µ L. Each tissue was sequenced on 1/12th of an Illumina HiSeq2000 lane with one lane used in total.”

Pg. 30: Which assemblies of the molluscan genomes (Crassostrea, etc.) were used? Where did the transcriptomes for Sepia and Idiosepius come from? Were reads downloaded from the NCBI SRA and assembled in Trinity? If so, what were the BioProejct numbers for the transcriptome data?

We have modified the methods section “Calibration of sequence divergence with respect to time” to include details as to the origin of both the transcriptomes and the genomes used in this study.

Revised text: (pg. 31-32, lines: 539-546)
Bioprojects for each genome used are as follows:Crassostera gigas (PRJNA629593 & PRJEB3535), Lottia gigantea (PRJNA259762 & PRJNA175706), Aplysia californica
(PRJNA629593 & PRJNA13635) and (Euprymna scolopes PRJNA47095). Octopus bimaculoides was obtained from http://octopus.unit.oist.jp/OCTDATA/BASIC/Metazome/Obimaculoides_280.fa.gz. The , Idiosepius notoides (BioProject: PRJNA302677) transcriptome was sequenced and assembled using the same method previously described for the H. maculosa transcriptome.

Pg. 32: I think when the authors write "H. maculosa is a single generation species", they mean that it is semelparous, but I don't see why that is relevant for mutation rate calculations.

We completely agree that this has little impact on mutation rate (measured per generation). This was included in an early draft (that discussed the coalescent demographic analyses in more detail) and was kept in by mistake. The text has been revised to remove "H. maculosa is a single generation species"

Original sentence:
"Per base neutral substitution between lineages was determined using the mean dS value divided by divergence time (refer to Calibration of sequence divergence with respect to time) usually over number of generations, however H. maculosa is a single generation species"

Revised sentence: (pg. 34, lines: 575-577)
"Per base neutral substitution between lineages was determined using the mean dS value divided by divergence time (refer to Calibration of sequence divergence with respect to time) over the number of generations."

Pg. 33: "genes with expression within one or more tissues was determined" should be "…were determined".

Corrected (pg. 35, line: 592).

Pg. 34: Something is strange in this sentence - "A loss of expression requires a gene to be present in all three octopods with and expressed in one or more species while having no detectable expression in at least one species" (an extra "with"?)

Corrected (pg. 35, lines: 602-604).

Pg. 34: Individual mutation with potential" should be "mutations"

Corrected (pg. 36, line: 613-614).

Figure 5: Loligo pealei and Doryteuthis pealeii are the same species. The latter name is the correct one.

Figure 5 has been corrected by removing the duplicate sequence and retaining the correct name Doryteuthis pealeii

Low quality version shown here. The corrected version has been uploaded to replace the original

Supplementary Material

4.1: a)What models and settings were used in RAxML and PhyloBayes?
b)How were those models chosen?
c)What calibrations were used for the divergence time analysis?
d)How was convergence inferred for the PhyloBayes run?
e)Also, Supplementary Figure 3 is a a "QITREE" tree…do the authors mean IQ-TREE? If so, why is IQ-TREE not mentioned in the text? This tree also differs slightly from the tree presented in Figure 1a, though I doubt the difference is of any consequence for this paper.

In order to clearly address the points raised by Reviewer1, we have answered each question separately.

a)The models chosen for RaxML and Phylobayes were GTR+G+I and strict clock with a mixture model of

F81 + G respectively.
b) These models were selected based on results from JmodelTest.
c) Calibrations were used on two nodes for the Phylobayes run : divergence between H. maculosa and E. scolopes 275mya & divergence between C. gigas and E. scolopes 500mya
d) Convergence was ascertained using tracecomp from the Phylobayes package
e) Two trees were run using the same alignment, the first was run with RAXML and the resulting tree used to inform Phylobayes, the second was run with IQTREE and included as a supplementary as it does not differ from the previous tree in any significant capacity. We have corrected the typo QITREE in the text.

We have added the details mentioned above to the supplementary materials section "4.1 Multi-gene cephalopod phylogeny and dating".

Original text:
"A total of 2,108 clusters were obtained. Phylogenies were constructed using RAxML31 and Phylobayes32. Divergence times were calculated using Phylobayes, calibrations, setting and model used"

Revised text: (pg. 15-16, lines: 222-232)
"A total of 2,108 clusters were obtained. Phylogenies were constructed using RAxML v8.031 and divergence times estimated by Phylobayes v4.132. RAxML v8.031 was run using the GTR+G+I model ascertained from JmodelTest v2.1.10. using the cAIC criterion for 100 bootstraps. Phylobayes estimated divergence times under a strict clock with a mixture model of F81+G with a burn-in of 10%. Calibrations were used as follows : divergence between H. maculosa and E. scolopes 275mya & divergence between C. gigas and E. scolopes 500mya. Two runs were performed and convergence verified using bpcomp, which confirmed a maximum difference of < 0.1 and tracecomp, which also indicated convergence with an effective sample size(EES) of > 200 for all parameters. Both programs used were from the Phylobayes package."

Reviewer #2: Review
Manuscript Number: GIGA-D-20-00135
Title: Adaptive venom evolution and toxicity in octopods is driven by extensive novel gene formation, expansion and loss
submitted to: GigaScience

This manuscript presents interesting data sets of both the genomic sequence of the TTX-bearing octopus Hapalochlaena maculosa as well as transcriptomes from twelve different tissues. The methods used were appropriate for the aims of the study including the use of two different methods to prepare (Illumina and Chicago), sequence (Illumina HiSeq 2000 and Dovetail) and assemble the genome (Illumina and HiRise). The authors do a good job reporting the statistical analysis of their assembly and comparing their statistics to two other octopus genomes, Callistoctopus minor and Octopus bimaculoides. Their methods of transcriptome sequencing, analysis and assembly were appropriate. Finally, their analysis of the completeness of their genome was appropriate and indicate that their genome is well constructed.
Their further analysis of the assembled genome and transcriptome are interesting and appropriate including the examination of the expansion of the zinc finger and cadherin/protocadherin gene families that have previously been identified in octopuses. Their analysis of expression differences in genes expressed in the posterior salivary gland between non-TTX bearing octopuses and the TTX-bearing H. maculosa is informative and suggests that the expression of serine protease venoms found in non TTX-bearing octopuses is reduced in H. maculosa. Finally, the authors confirm that H. maculosa has the same set of amino acid substitutions that are found in the voltage-gated sodium channel NaV1 of Hapalochlaena lunalata. In both species, these changes in channel structure are likely to impart TTX resistance and explain the genetic mechanism underlying TTX resistance in the genus.
The authors appear to have met the minimum standard of reporting for the journal. However, the authors have not done an adequate job of reviewing the scientific literature that would contextualize their work and this has led to inaccurate statements in the manuscript. The manuscript requires editing for clarity. I will highlight several of the problem sections below.

1) In the abstract/background the authors state "Cephalopods represent a rich system for investigating the genetic basis underlying organismal novelties. This diverse group of specilised predators have evolved many unique adaptations including proteinaceous venom." Proteinaceous venoms are not unique to cephalopods. Snakes have evolved the use of proteinaceous venoms that function as enzymes including serine proteases that the authors suggest are unique to cephalopods. For an example, see a review in Toxicon from 2013 by Solange and Serrano. The authors could strengthen this manuscript by

discussing their work in the context of the independent evolution in vertebrates and invertebrate lineages of the use of this enzyme class. The author's interesting report that serine protease expression is reduced in a tetrodotoxin (TTX) bearing cephalopod compared to non-TTX bearing cephalopods is overshadowed by this mischaracterization of the uniqueness of this character in cephalopods.

It was not our intention to convey that cephalopods are the only taxa to have evolved proteinaceous venom and to rectify this we have modified the sentence in the abstract to more accurately represent the literature. Additionally, we clarified the independent evolution between invertebrates and vertebrates in the introduction.

Abstract:
Original text:
"Cephalopods represent a rich system for investigating the genetic basis underlying organismal novelties. This diverse group of specilised predators have evolved many unique adaptations including proteinaceous venom."

Revised text: (pg.1, lines:23-25)
“Cephalopods represent a rich system for investigating the genetic basis underlying organismal novelties. This diverse group of specialized predators has evolved many adaptations including proteinaceous venom.”

Background:
Original text:
“Furthermore, proteinaceous venom is produced and stored within a specialised gland in cephalopods known as the posterior salivary gland (PSG)”

Revised text: (pg.3-4, lines:63-71)
“Furthermore, the cephalopods have independently evolved proteinaceous venom, which is produced and stored within a specialised gland in known as the posterior salivary gland (PSG). All octopods are believed to possess a form of proteinaceous venom used to subdue prey8–10. Serine proteases are a common component of cephalopod venoms and have been observed in the PSG of squids, cuttlefish and octopods10–13. Convergent recruitment of serine proteases has been observed between many vertebrate (Squamata14–16 and Monotremata17) and invertebrate (Hymenoptera18, Arachnida19, Gastropoda20, Remipedia21 and Cnidarian22) venomous lineages.”

2) In the abstract/findings description the authors state "…voltage-gated sodium channels in H. maculosa contain a resistance mutation found in pufferfish and garter snakes, which is absent in other octopods." Hapalochlaena maculosa has the same amino acid sequences encoded in the voltage-gated sodium channel genes NaV1 and NaV2 as previously reported for the Greater Blue-ringed octopus Hapalochlaena lunulata, in Toxicon from 2019 by Geffeney and colleagues.

This sentence has been corrected to reflect the recent finding of the resistance mutations by Gefferny et al 2019 in H. lunulata.

Original sentence:
“Moreover, voltage-gated sodium channels in H. maculosa contain a resistance mutation found in pufferfish and garter snakes, which is absent in other octopods”

Modified sentence: (pg.2, lines:38-40)
“Moreover, voltage-gated sodium channels in H. maculosa contain a resistance mutation found in pufferfish and garter snakes, which is exclusive to the genus.”

3) In the background section of the main body the authors state "How resistance to TTX has been acquired at the genetic level, remains a large unknown, with TTX resistance studied in only in a few select species (i.e. pufferfish13, newts14,15 and gastropods16)." The changes in voltage-gated sodium channel genes that lead to TTX-resistance are well understood. Genetic changes that lead to TTX resistance have been examined in groups not included in the authors list including other invertebrates (e.g. insects and blue-ringed octopuses) as well as snakes. The authors statement mischaracterizes the body of literature examining the evolution of TTX resistance.

This section has been modified to more accurately depict the current literature by providing more

examples of taxa which exhibit resistance to TTX. We now included an example of an arachnid whose channels have been examined for TTX resistance, however we were unable to find an example of an insect as suggested above by reviewer 2. We would be happy to include an example of an insect if the reference could be provided.

Original text:
"How resistance to TTX has been acquired at the genetic level, remains a large unknown, with TTX resistance studied in only in a few select species (i.e. pufferfish13, newts14,15 and gastropods16)."

Revised text: (pg. 4, lines: 74-89)
"The mechanism of TTX resistance, which allows for safe sequestration of TTX, has been attributed to several substitutions in the p-loop regions of voltage-gated sodium channels(Nav) in H. lunulata24. However, these channels have yet to be examined in H. maculosa and H. fasciata. TTX resistance has also been studied in a range of other genera including, pufferfish25, newts26,27 arachnids28, snakes29 and gastropods30."

4) In the data description the authors discuss their work to identify the expansion of genes in the cadherin/protocadherin gene family. This section requires citations as well as correction of existing citations.

a. The authors state "H. maculosa and C. minor exhibit expansions in the cadherin gene family, characteristic of other octopod genomes, including O. bimaculoides (Fig1b)." without including a reference. This statement requires a reference and the discussion of their data would be improved by comparing their findings to other articles that have examined the expansion of the cadherin gene family and specifically protocadherins, for example Styfhals et al. (2019) in Frontiers in Physiology.

We agree with the reviewer that further discussion was required for this section and have restructured the paragraph to the following:

Original text:
"Organismal impact of novel genes and gene family expansions
Gene family expansions between octopods (O. bimaculoides, C. minor and H. maculosa) and three other molluscan genomes (Aplysia californica, Lottia gigantea and Crassostrea gigas) were examined using Pfam annotations. A total of 5565 Pfam domains were identified among six molluscan genomes. H. maculosa and C. minor exhibit expansions in the cadherin gene family, characteristic of other octopod genomes, including O. bimaculoides (Fig1b). C. minor, in particular, shows the greatest expansion of this family within octopods. Expansions of protocadherins, a subset of the cadherin family, have also occurred independently in squid 20, with the octopod expansions occuring post divergence ~135 mya 20. Cadherins, specifically protocadherins, are essential mediators of short-range neuronal connections in mammals42 43. Due to the absence of a myelin sheath in octopods, short-range connections are integral to maintaining signal fidelity over distance44."

Revised text: (pg. 10-12, lines: 183-206)
"Organismal impact of novel genes and gene family expansions
Gene family expansions between octopods (O. bimaculoides, C. minor and H. maculosa) and three other molluscan genomes (Aplysia californica, Lottia gigantea and Crassostrea gigas) were examined using Pfam annotations. A total of 5565 Pfam domains were identified among six molluscan genomes. H. maculosa and C. minor exhibit expansions in the cadherin gene family, characteristic of other octopod genomes, including O. bimaculoides (Fig1b)42,64. C. minor, in particular, shows the greatest expansion of this family within octopods. Expansions of protocadherins, a subset of the cadherin family, have also occurred independently in squid42, with the octopod expansions occurring post divergence ~135 mya42. The shared ancestry of octopod cadherins was also documented by Styfhals et al64 using phylogenetic inference between O. bimaculoides and O. vulgaris.Cadherins, specifically protocadherins, play crucial roles in synapse formation, elimination and axon targeting within mammals and are essential mediators of short-range neuronal connections65–68. It should be noted that octopods lack a myelin sheath, as a result short-range connections are integral to maintaining signal fidelity over distance6. The independent expansions of protocadherins within chordate and cephalopod lineages are believed to be associated with increased neuronal complexity42,64. Elevated expression of protocadherins within neural tissues have been observed in O. vulgaris and O. bimaculoides by both Styfhals et al64 and Albertin et al42 respectively. In particular Styfhals et al64 noted differential expression across neural tissues including

supra-esophageal mass, sub-esophageal mass, optic lobe and the stellate ganglion64. However, functional implications of observed expression patterns remain speculative without further study.”

b. The next sentence ("Expansions of protocadherins, a subset of the cadherin family, have also occurred independently in squid 20, with the octopod expansions occuring post divergence ~135 mya 20.") incorrectly references Williams et al. (2012, reference 20) but should reference Albertin et al. (2015, reference 29).

This reference was corrected as suggested: (pg. 11, line:190-192)

“Expansions of protocadherins, a subset of the cadherin family, have also occurred independently in squid42, with the octopod expansions occurring post divergence ~135 mya42”

c. The authors state "Cadherins, specifically protocadherins, are essential mediators of short-range neuronal connections in mammals42 43. Due to the absence of a myelin sheath in octopods, short-range connections are integral to maintaining signal fidelity over distance44." None of the citations in these two sentences are correct and no correct references can be found in the list of citations. These final statements should include the fact that expansion in the number of protocadherin genes also occurs in chordates (for example, Hulpiau & van Roy, 2010 from Molecular Biology and Evolution). There is good evidence that protocadherins have roles in multiple aspects of proper synapse formation in mammals including synapse generation, synapse elimination and axon targeting (for example see reviews by de Wit and Ghosh from 2016 in Nature Reviews Neuroscience as well as Peek et al. from 2017 in Cellular and Molecular Life Sciences). Though synapses are "short-range connections", proper synapse formation is important for vertebrates and invertebrates with complex nervous systems whether or not that have myelinated axons. The expansion of protocadherin genes in both cephalopods and chordates independently is thought to be linked to increased neuronal circuit complexity.

We have corrected the miscitations and restructured the paragraph to incorporate the reviewers suggestions and more accurately describe the evolution and role of protocadherins in vertebrates and invertebrates.

Original text:
“Cadherins, specifically protocadherins, are essential mediators of short-range neuronal connections in mammals42 43. Due to the absence of a myelin sheath in octopods, short-range connections are integral to maintaining signal fidelity over distance44.”

Revised text: (pg. 11-12, lines: 194-206)
“Cadherins, specifically protocadherins, play crucial roles in synapse formation, elimination and axon targeting within mammals and are essential mediators of short-range neuronal connections65–68. It should be noted that octopods lack a myelin sheath, as a result short-range connections are integral to maintaining signal fidelity over distance6. The independent expansions of protocadherins within chordate and cephalopod lineages are believed to be associated with increased neuronal complexity42,64. Elevated expression of protocadherins within neural tissues have been observed in O. vulgaris and O. bimaculoides by both Styfhals et al64 and Albertin et al42 respectively. In particular Styfhals et al64 noted differential expression across neural tissues including supra-esophageal mass, sub-esophageal mass, optic lobe and the stellate ganglion64. However, functional implications of observed expression patterns remain speculative without further study.”

5) In the data description the authors discuss their work to identify the expansion of genes in the zinc finger gene family. The author state "H. maculosa also shows expansions in the C2H2-type zinc finger family. Zinc fingers form an ancient family of transcription factors, which among other roles serve to regulate transposon splicing, embryonic and neural development 45,46." These references are not correct for this statement. The manuscript would be strengthened by proper citations in this section, for example Fedotova and colleagues (2017) have a review in Acta Naturae. Additionally, there is evidence that these proteins have roles in both transposon suppression and alternative splicing.

Citations were corrected as suggested: (pg. 12, lines: 207-209)

“H. maculosa also shows expansions in the C2H2-type zinc finger family. Zinc fingers form an ancient family of transcription factors, which among other roles serve to regulate transposon splicing as well as embryonic and neural development69,70.”

6) The authors state "It has been hypothesized that the Hapalochlaena PSG will exhibit a loss of redundant proteinaceous toxins due to the presence of TTX." This sentence should have a citation or the authors should explain that this statement is their hypothesis.

This statement is a hypothesis by the authors and the sentence has been modified to reflect this.

Original sentence:
“It has been hypothesized that the Hapalochlaena PSG will exhibit a loss of redundant proteinaceous toxins due to the presence of TTX.”

Revised sentence: (pg.17, lines: 299-300)
“We hypothesize that the Hapalochlaena PSG will exhibit a loss of redundant proteinaceous toxins due to the presence of TTX.”

7) There are minor errors in the sequences presented in Figure 5. In multiple invertebrate species, phenylalanine (F) replaces tyrosine (Y) in the D1 pore. In pufferfish cysteine (C) replaces tyrosine (Y). The figure is constructed in a way that suggests that these amino acids replace a neighboring aspartic acid (D).

Figure 5 has been modified so all sequences align correctly.
Low quality version shown here. The corrected version has been uploaded to replace the original

Reviewer #3
A truly excellent paper that was a pleasure to read. My comments are very minor:
- TTX resistance in Thamnophis species of snakes should be referenced in the sentence "How resistance to TTX has been acquired at the genetic level, remains a large unknown, with TTX resistance studied in only in a few select species (i.e. pufferfish13, newts14,15 and gastropods16)". This is cited later (ref 58) but it would be appropriate for inclusion in this sentence too.
This citation has been added as suggested to a modified version of this sentence as suggested by reviewer 1:

Original text:
"How resistance to TTX has been acquired at the genetic level, remains a large unknown, with TTX resistance studied in only in a few select species (i.e. pufferfish13, newts14,15 and gastropods16)"
Revised text: (pg. 4, lines: 74-79)
“The mechanism of TTX resistance, which allows for safe sequestration of TTX, has been attributed to several substitutions in the p-loop regions of voltage-gated sodium channels(Nav) in H. lunulata13. However, these channels have yet to be examined in H. maculosa and H. fasciata. TTX resistance has also been studied in a range of other genera including, pufferfish14, newts15,16 arachnids17, snakes18 and gastropods19.

- For the PSG specific genes, calculations of the relative rates of evolution would be informative as this would be suggestive of adaptive evolution eg are the abundant serine proteases C. minor showing signs of accelerated evolution seen in other venomous lineages such as snakes? Previous work has shown that the sites on the molecular surface are undergoing episodic diversification when compared across a wide range of lineages. In this case, it would be interesting to see what the evolutionary patterns are for C. minor, in that is the extensive duplication accompanied by signs of diversification?
We investigated the potential of positive selection within serine proteases with a focus on genes specifically expressed in the posterior salivary gland (venom gland). Unfortunately, we did not find strong evidence of accelerated evolution in these genes with the method described below. In the future we look forward to conducting a more in-depth analysis of this interesting family with a more comprehensive sampling across coleoid cephalopods.
This section below, describing our additional analyses, has been added to the supplementary materials: (pg. 24-25, lines: 355-382)
6.4 Examination of selection and evolutionary rates in octopod serine proteases
Gene models (aa) from the three octopod genomes (H. maculosa, O. bimaculoides and C. minor) were annotated with Interproscan and serine proteases with the Pfam PF00089 extracted for examination. Gene models and their corresponding CDS sequences were imported into Geneious v10.2.6 and selected for a single trypsin (PF00089) domain greater than 200aa/600bp long. The region containing the trypsin domain was then extracted from the nucleic acid sequences and MAFFT v7.407 was used to align

sequences using Translation align in Geneious v10.2.6, which interpreted the first codon as the start of the codon region and used the first translation frame. The resulting alignment was tested for an appropriate substitution model in jModelTest v2.2.10 and a tree was generated with RAxML v8.0 using the GTR +G+I model and 100 bootstraps. The resulting tree and alignment were examined using codeml via EasyCodeml v1.21 from the PAML package to examine non-synonymous to synonymous substitution rates for evidence of positive selection. We first used a site-based model which allows for ω values to vary between sites along the protein. Comparison of the nested models (M1a-M2a) and (M7-M8) did not reveal any sites under positive selection ($p > 0.05$). In order to access the potential for different rates of evolution within specific lineages we used a branch site model which allows for ω values to vary between sites and branches. For the foreground a large clade of genes, majority of which were specifically expressed in the posterior salivary gland (PSG) was selected and compared to all other non-PSG specific genes. No sites among the foreground branches were significantly accelerated relative to the background. The last method implemented is similar to the branch site model, however, the rate along sites is constant and the rate between the background and foreground can differ. This also found no evidence of positive selection between the background and foreground lineages. It should be noted that serine proteases are a large and complex family and are due a more in-depth analysis in coleoid cephalopods, which could form a complete stand-alone study.
- The M12B metalloprotease type in snake venom has a wide range of demonstrated activities, both anticoagulant (fibrinogenolytic) but also procoagulant (Factor X activating [Atractaspis and Daboia venoms] and prothrombin activating (Bothrops, Echis, and Dispholidus/Thelatornis venoms]) prothrombin activating metalloproteases from Dispholidus typus (boomslang) and Thelotornis mossambicanus (twig snake).
" Comp Biochem Physiol C Toxicol Pharmacol: 108625. Oulion, B., J. S. Dobson, C. N. Zdenek, K. Arbuckle, C. Lister, F. C. P. Coimbra, B. Op den Brouw, J. Debono, A. Rogalski, A. Violette, R. Fourmy, N. Frank and B. G. Fry (2018). "Factor X activating Atractaspis snake venoms and the relative coagulotoxicity neutralising efficacy of African antivenoms." Toxicol Lett 288: 119-128.
Rogalski, A., C. Soerensen, B. Op den Brouw, C. Lister, D. Dashevsky, K. Arbuckle, A. Gloria, C. N. Zdenek, N. R. Casewell, J. M. Gutierrez, W. Wuster, S. A. Ali, P. Masci, P. Rowley, N. Frank and B. G. Fry (2017). "Differential procoagulant effects of saw-scaled viper (Serpentes: Viperidae: Echis) snake venoms on human plasma and the narrow taxonomic ranges of antivenom efficacies." Toxicol Lett 280: 159-170.
Sousa, L. F., C. N. Zdenek, J. S. Dobson, B. Op den Brouw, F. Coimbra, A. Gillett, T. H. M. Del-Rei, H. M. Chalkidis, S. Sant'Anna, M. M. Teixeira-da-Rocha, K. Grego, S. R. Travaglia Cardoso, A. M. Moura da Silva and B. G. Fry (2018). "Coagulotoxicity of Bothrops (Lancehead Pit-Vipers) Venoms from Brazil: Differential Biochemistry and Antivenom Efficacy Resulting from Prey-Driven Venom Variation." Toxins (Basel) 10(10): 411.
We agree that these references should be included in the manuscript and have added them as appropriate. (pg. 18, lines:316-319)

“Similarly, reprolysin (M12B) exhibits shifting expression in H. maculosa, presumably from the PSG to the branchial heart, and a complete loss of paralogs from the genome. While the function of this protein has not been assessed in octopus, members of this protein family exhibit anticoagulant properties in snake venom75–78.

Close
