# Supplementary material for: Adaptive venom evolution and toxicity in octopods is driven by extensive novel gene formation, expansion, and loss
Source: Gigascience. 2020 Nov 10;9(11):giaa120. doi: 10.1093/gigascience/giaa120 (PMC7656900; doi:10.1093/gigascience/giaa120)
Supplement: giaa120_Response_to_Reviewer_Comments_Revision_1 [file giaa120_response_to_reviewer_comments_revision_1.pdf]

Author's Response To Reviewer Comments

Close

We would like to thank the reviewers for their helpful comments, which have been addressed below and the editor for considering our manuscript for publication.

Reviewer reports:
Reviewer #1: The authors have made the changes that I requested. I confess that I did not carefully read through the revision to verify that all of the minor grammatical edits I proposed were made, but I "spot checked" several and they look pretty good.

Reviewer #2: The authors have adequately revised their manuscript in response to my review and addressed the comments in my review with their letter. I have just two minor suggestions after reading the revised manuscript.

1) The authors should choose one convention for spelling the word specialized either specialised (British) or specialized (American).

We have chosen to use the British spelling and corrected the one American spelling at pg. 1, line 24.

2) In lines 372-374, the authors comment on the interactions between aspartic acid and TTX, "While it has yet to be assessed for TTX resistance, the replacement of Asp in B. candida with a neutral amino acid has been predicted to disrupt TTX binding by preventing formation of a hydrogen bond." A better reference for this statement is Shen et al. (2018). In addition, the cryo-EM structure data from this paper suggest that either a hydrogen bond or a salt bridge could form between TTX and that aspartic acid at this position of the protein.

We agree that this sentence required clarification, specifically that both hydrogen and salt bridges could be disrupted. This information has been added to the modified sentence as well as the suggested reference.

Original sentence:
“While it has yet to be assessed for TTX resistance, the replacement of Asp in B. candida with a neutral amino acid has been predicted to disrupt TTX binding by preventing formation of a hydrogen bond90”

Revised sentence: (pg. 17, lines : 396-398)
“While it has yet to be assessed for TTX resistance, the replacement of Asp in B. candida with a neutral amino acid has been predicted to disrupt TTX binding by preventing formation of a salt bridge or hydrogen bond89,91”

Close
