# Supplementary material for: Adaptive venom evolution and toxicity in octopods is driven by extensive novel gene formation, expansion, and loss
Source: Gigascience. 2020 Nov 10;9(11):giaa120. doi: 10.1093/gigascience/giaa120 (PMC7656900; doi:10.1093/gigascience/giaa120)
Supplement: giaa120_Reviewer_1_Report_Original_Submission — Frank Anderson -- 6/3/2020 Reviewed [file giaa120_reviewer_1_report_original_submission.pdf]

Reviewer Report

Title: Adaptive venom evolution and toxicity in octopods is driven by extensive novel gene formation, expansion and loss

Version: Original Submission **Date:** 6/3/2020

Reviewer name: Frank Anderson

Reviewer Comments to Author:

The manuscript describes the genome of the southern blue-ringed octopus and provides comparisons between this genome and previously published octopus genomes, with a particular focus on venom. I did not see any major flaws in the paper, which I think will be a valuable contribution to the cephalopod genomics literature. The blue-ringed octopuses are of major interest due to their highly toxic venom, and I found the comparisons between this species and others in terms of venom production and resistance to be quite illuminating (though somewhat unsurprising, given what we already knew about the use of TTX by this species).

I found the paper to be fairly well written, though I do have several questions and comments that I hope will help clarify some issues. I will list those below, in the order I encountered them as I read the manuscript and supplementary materials. My only somewhat substantive concern first struck me as I read page 17 of the manuscript: "…suggesting a species-specific expansion of this cluster in C. minor". I think the authors should be a bit more careful with how they use the phrase "species-specific". They have included only three octopod species out of 300+ species in this study. Yes, any differences they detect between these species could be species specific, but I think it is more likely that the differences arose in ancestral lineages. For example, expansion of the serine protease cluster may have occurred only in C. minor, but it could also have occurred in the ancestor of Callistoctopus, or in some other ancestor. At present, the authors do not have sufficient sampling to know if any of the expansions, losses, shifts in expression, etc., they are seeing are truly species specific. Similarly, sentences like "Loss of serine protease genes can also be observed in H. maculosa". The authors can certainly state that H. maculosa has fewer serine protease genes than O. bimaculoides and C. minor, but the *loss* of these genes may have occurred in H. maculosa *or* in any ancestral lineage after the divergence of Hapalochlaena from Octopus. I urge the authors to go through their manuscript carefully to find instances where they have evidence of differences among these species and to check that their descriptions of differences among these species are clear.

Minor points and suggestions

Check for subject-verb agreement in the abstract. For example, it should be "This diverse group of specilised (sic) predators has evolved…" (the subject is "group", not "predators").

Abstract: Last sentence might be better as "This genome, along with other recently published cephalopod genomes, represents a valuable resource from which future work could advance our understanding of the evolution of genomic novelty in this family"

Pg. 3: "underrepresented" - Underrepresented in what sense? In terms of genomic resources?

Pg. 3: (FAO,) - Looks like a typo? Or incomplete switch to a different citation format?

Pg. 3: "soft bodied" should be "soft-bodied"
Pg. 3: Should be "that are well adapted" (no hyphen)
Pg. 4: "How resistance to TTX" - This is not totally clear as written. Resistance in what? I think the authors are referring to how Hapalochlaena avoids being killed by its own TTX, but this could be rephrased to make it crystal clear. Also "remains a large unknown" is a little awkward…maybe "remains largely unknown" would be better?
Pg. 4: "Primarily used for defense…" - Awkward sentence with a dangling modifier, which makes it read as though Hapalochlaena is primarily used for defense in other species.
Pg. 5: "for example at the evolution of venoms" - Somewhat awkward, I think?
Pg. 6: Capitalize "bay"? (Port Philip Bay)
Pg. 7: Should be "shallow-water marine organisms".
Pg. 8: Should be "Southern Hemisphere".
Pg. 8: Also here, how did they do their divergence time estimation?
Pg. 8: "maculosa from Abdopus" - "from" should not be italicized.
Pg. 9: "sporadic occurrence" - I think the authors mean sporadic taxonomically here (i.e., some species have them, some do not), but this should be clarified (surely the authors don't mean that sometimes a given species has them and sometimes they don't!).
Pg. 11: Just a suggestion here: "splicing, embryonic and neural development" is clear, but it looks odd. How about "splicing and embryonic and neural development" or "splicing as well as embryonic and neural development".
Pg. 11: Should be "this type of zinc finger in O. bimaculoides"
Pg. 12: "High level examination" should be "High-level examination", "large scale expression patterns" should be "large-scale expression patterns", and "lineage specific loss" should be "lineage-specific loss".
Pg. 12: Unnecessary comma in "we also find that, genes specific to each octopod"
Pg. 13: "in tandem with overall reduction in genes number relative to the octopods" - This is unclear and poorly worded. I assume this is referring still to H. maculosa relative to other octopods?
Pg. 15: Unnecessary comma in "More notable, were differences"
Pg. 16: Should be "primary venom-producing gland".
Pg. 16: Haplochlaena should be italicized in "hypothesized that the Hapalochlaena PSF…" Also, who has hypothesized that the Hapalochlaena PSF will exhibit a loss of redundant proteinaceous toxins? This seems to call for a citation.
Pg. 16: "A total of 623 genes were exclusive to H. maculosa PSF…exclusive to O. bimculoides and C. minor, respectively". Should this be "exclusive to the O. bimaculoides and C. minor PSGs, respectively"?
Pg. 16: "Additionally, H. maculosa PSG is predicted to be" - Predicted by whom? The authors? I think so, so they should make that clear, e.g., "we predict that the H. maculosa PSG is functionally more diverse…"
Pg. 17: The sentence about reprolysin doesn't make sense to me. The authors describe shifting expression in this species (see my comment above) but then note that there is a complete loss of orthologs from the genome. This latter comment suggests that reprolysin doesn't even exist in the H. maculosa genome, but it must, if it is showing different expression patterns than the other species. Can the authors clarify this?
Pg. 18: "the cephalopod specific clade" should be "cephalopod-specific clade".

Pg. 18: "…hyaluronidase, which often serve as dispersal factors" seems odd. Should this be "hyaluronidase, which often serves as a dispersal factor"?
Pg. 19: "Two Nav genes"…should the "v" be a subscript here?
Pg. 19: "latter regions in DIII and DIV" - Is "latter" the best adjective here?
Pg. 20: "In previous studies, when examined individually, the Met- Thr substitution in a TTX sensitive Nav1.4 rat channel decreased binding affinity in pufferfish by 15-fold" - This could be more clear. A "rat channel"? As in, a channel in rats? But the sentence says "in pufferfish". Please clarify this.
Pg. 21: "It has yet to be established if these mutations are derived from a shared ancestor or have occurred independently" - Excellent. This is exactly the clarity I think the authors can bring to their other statements about gains, losses, etc. that I pointed out in a previous comment.
Pg. 21: I would write "While Hapalochlaena remains" here.
Pg. 21: Should be "STX-contaminated bivalves" (and "STX-contaminated fish" and "STX-contaminated food sources" below…and "TTX-producing bacteria" and "TTX-producing strains" on pg. 23).
Pg. 21: "Humboldt" should be capitalized, as it is a proper name.
Pg. 23: "Sequestration of TTX is not exclusive to the blue-ringed octopus among molluscs. Gastropods such as Pleurobranchaea maculata and Niotha clathrata, as well as some bivalves, are capable of sequestering the similar toxin STX " - TTX and STX are similar, but not the same. Are there other examples of actual TTX sequestration in molluscs, outside of Hapalochlaena? If not, this should be rephrased.
Pg. 23: "highly diverse composition of genera" may be better as "highly diverse composition of bacterial genera"
Pg. 23: "Diversity of bacterial genera much like the H. maculosa in this study was high" - This seems awkward and unclear."
Pg. 24: "TTX bearing mollusk genome" - Hmm…does the genome bear TTX? The mollusk does, I suppose, but it's produced by bacteria as described above. This could be rephrased.
Pg. 28: What kit or method was used to construct the cDNA libraries for transcriptome sequencing?
Pg. 30: Which assemblies of the molluscan genomes (Crassostrea, etc.) were used? Where did the transcriptomes for Sepia and Idiosepius come from? Were reads downloaded from the NCBI SRA and assembled in Trinity? If so, what were the BioProejct numbers for the transcriptome data?
Pg. 32: I think when the authors write "H. maculosa is a single generation species", they mean that it is semelparous, but I don't see why that is relevant for mutation rate calculations.
Pg. 33: "genes with expression within one or more tissues was determined" should be "…were determined".
Pg. 34: Something is strange in this sentence - "A loss of expression requires a gene to be present in all three octopods with and expressed in one or more species while having no detectable expression in at least one species" (an extra "with"?)
Pg. 34: Individual mutation with potential" should be "mutations"
Figure 5: Loligo pealei and Doryteuthis pealeii are the same species. The latter name is the correct one.
Supplementary Material
4.1: What models and settings were used in RAxML and PhyloBayes? How were those models chosen? What calibrations were used for the divergence time analysis? How was convergence inferred for the PhyloBayes run? Also, Supplementary Figure 3 is a a "QITREE" tree…do the authors mean IQ-TREE? If so,

why is IQ-TREE not mentioned in the text? This tree also differs slightly from the tree presented in Figure 1a, though I doubt the difference is of any consequence for this paper.

Methods

Are the methods appropriate to the aims of the study, are they well described, and are necessary controls included? Choose an item.

Conclusions

Are the conclusions adequately supported by the data shown? Choose an item.

Reporting Standards

Does the manuscript adhere to the journal's guidelines on [minimum standards of reporting?]() Choose an item.

Choose an item.

Statistics

Are you able to assess all statistics in the manuscript, including the appropriateness of statistical tests used? Choose an item.

Quality of Written English

Please indicate the quality of language in the manuscript: Choose an item.

Declaration of Competing Interests

Please complete a declaration of competing interests, considering the following questions:

- Have you in the past five years received reimbursements, fees, funding, or salary from an organisation that may in any way gain or lose financially from the publication of this manuscript, either now or in the future?
- Do you hold any stocks or shares in an organisation that may in any way gain or lose financially from the publication of this manuscript, either now or in the future?
- Do you hold or are you currently applying for any patents relating to the content of the manuscript?
- Have you received reimbursements, fees, funding, or salary from an organization that holds or has applied for patents relating to the content of the manuscript?
- Do you have any other financial competing interests?
- Do you have any non-financial competing interests in relation to this paper?

If you can answer no to all of the above, write 'I declare that I have no competing interests' below. If your reply is yes to any, please give details below.

I declare that I have no competing interests.

I agree to the open peer review policy of the journal. I understand that my name will be included on my report to the authors and, if the manuscript is accepted for publication, my named report including any attachments I upload will be posted on the website along with the authors' responses. I agree for my report to be made available under an Open Access Creative Commons CC-BY license (http://creativecommons.org/licenses/by/4.0/). I understand that any comments which I do not wish to be included in my named report can be included as confidential comments to the editors, which will not be published.

Choose an item.

To further support our reviewers, we have joined with Publons, where you can gain additional credit to further highlight your hard work (see: https://publons.com/journal/530/gigascience). On publication of this paper, your review will be automatically added to Publons, you can then choose whether or not to claim your Publons credit. I understand this statement.

Yes Choose an item.
