# Supplementary material for: Adaptive venom evolution and toxicity in octopods is driven by extensive novel gene formation, expansion, and loss
Source: Gigascience. 2020 Nov 10;9(11):giaa120. doi: 10.1093/gigascience/giaa120 (PMC7656900; doi:10.1093/gigascience/giaa120)
Supplement: giaa120_Reviewer_2_Report_Original_Submission — Shana Geffeney -- 6/8/2020 Reviewed [file giaa120_reviewer_2_report_original_submission.pdf]

Reviewer Report

Title: Adaptive venom evolution and toxicity in octopods is driven by extensive novel gene formation, expansion and loss

Version: Original Submission **Date:** 6/8/2020

Reviewer name: Shana Geffeney

Reviewer Comments to Author:

Review
Manuscript Number: GIGA-D-20-00135
Title: Adaptive venom evolution and toxicity in octopods is driven by extensive novel gene formation, expansion and loss
submitted to: GigaScience
This manuscript presents interesting data sets of both the genomic sequence of the TTX-bearing octopus Hapalochlaena maculosa as well as transcriptomes from twelve different tissues. The methods used were appropriate for the aims of the study including the use of two different methods to prepare (Illumina and Chicago), sequence (Illumina HiSeq 2000 and Dovetail) and assemble the genome (Illumina and HiRise). The authors do a good job reporting the statistical analysis of their assembly and comparing their statistics to two other octopus genomes, Callistoctopus minor and Octopus bimaculoides. Their methods of transcriptome sequencing, analysis and assembly were appropriate. Finally, their analysis of the completeness of their genome was appropriate and indicate that their genome is well constructed.

Their further analysis of the assembled genome and transcriptome are interesting and appropriate including the examination of the expansion of the zinc finger and cadherin/protocadherin gene families that have previously been identified in octopuses. Their analysis of expression differences in genes expressed in the posterior salivary gland between non-TTX bearing octopuses and the TTX-bearing H. maculosa is informative and suggests that the expression of serine protease venoms found in non TTX-bearing octopuses is reduced in H. maculosa. Finally, the authors confirm that H. maculosa has the same set of amino acid substitutions that are found in the voltage-gated sodium channel NaV1 of Hapalochlaena lunalata. In both species, these changes in channel structure are likely to impart TTX resistance and explain the genetic mechanism underlying TTX resistance in the genus.

The authors appear to have met the minimum standard of reporting for the journal. However, the authors have not done an adequate job of reviewing the scientific literature that would contextualize their work and this has led to inaccurate statements in the manuscript. The manuscript requires editing for clarity. I will highlight several of the problem sections below.

1) In the abstract/background the authors state "Cephalopods represent a rich system for investigating the genetic basis underlying organismal novelties. This diverse group of specilised predators have evolved many unique adaptations including proteinaceous venom." Proteinaceous venoms are not unique to cephalopods. Snakes have evolved the use of proteinaceous venoms that function as enzymes including serine proteases that the authors suggest are unique to cephalopods. For an example, see a review in Toxicon from 2013 by Solange and Serrano. The authors could strengthen

this manuscript by discussing their work in the context of the independent evolution in vertebrates and invertebrate lineages of the use of this enzyme class. The author's interesting report that serine protease expression is reduced in a tetrodotoxin (TTX) bearing cephalopod compared to non-TTX bearing cephalopods is overshadowed by this mischaracterization of the uniqueness of this character in cephalopods.

2) In the abstract/findings description the authors state "…voltage-gated sodium channels in H. maculosa contain a resistance mutation found in pufferfish and garter snakes, which is absent in other octopods." Hapalochlaena maculosa has the same amino acid sequences encoded in the voltage-gated sodium channel genes NaV1 and NaV2 as previously reported for the Greater Blue-ringed octopus Hapalochlaena lunulata, in Toxicon from 2019 by Geffeney and colleagues.

3) In the background section of the main body the authors state "How resistance to TTX has been acquired at the genetic level, remains a large unknown, with TTX resistance studied in only in a few select species (i.e. pufferfish13, newts14,15 and gastropods16)." The changes in voltage-gated sodium channel genes that lead to TTX-resistance are well understood. Genetic changes that lead to TTX resistance have been examined in groups not included in the authors list including other invertebrates (e.g. insects and blue-ringed octopuses) as well as snakes. The authors statement mischaracterizes the body of literature examining the evolution of TTX resistance.

4) In the data description the authors discuss their work to identify the expansion of genes in the cadherin/protocadherin gene family. This section requires citations as well as correction of existing citations.

a. The authors state "H. maculosa and C. minor exhibit expansions in the cadherin gene family, characteristic of other octopod genomes, including O. bimaculoides (Fig1b)." without including a reference. This statement requires a reference and the discussion of their data would be improved by comparing their findings to other articles that have examined the expansion of the cadherin gene family and specifically protocadherins, for example Styfhals et al. (2019) in Frontiers in Physiology.

b. The next sentence ("Expansions of protocadherins, a subset of the cadherin family, have also occurred independently in squid 20, with the octopod expansions occuring post divergence ~135 mya 20.") incorrectly references Williams et al. (2012, reference 20) but should reference Albertin et al. (2015, reference 29).

c. The authors state "Cadherins, specifically protocadherins, are essential mediators of short-range neuronal connections in mammals42 43. Due to the absence of a myelin sheath in octopods, short-range connections are integral to maintaining signal fidelity over distance44." None of the citations in these two sentences are correct and no correct references can be found in the list of citations. These final statements should include the fact that expansion in the number of protocadherin genes also occurs in chordates (for example, Hulpiau & van Roy, 2010 from Molecular Biology and Evolution). There is good evidence that protocadherins have roles in multiple aspects of proper synapse formation in mammals including synapse generation, synapse elimination and axon targeting (for example see reviews by de Wit and Ghosh from 2016 in Nature Reviews Neuroscience as well as Peek et al. from 2017 in Cellular and Molecular Life Sciences). Though synapses are "short-range connections", proper synapse formation is important for vertebrates and invertebrates with complex nervous systems whether or not that have myelinated axons. The expansion of protocadherin genes in both cephalopods and chordates independently is thought to be linked to increased neuronal circuit complexity.

5) In the data description the authors discuss their work to identify the expansion of genes in the zinc finger gene family. The author state "H. maculosa also shows expansions in the C2H2-type zinc finger family. Zinc fingers form an ancient family of transcription factors, which among other roles serve to regulate transposon splicing, embryonic and neural development 45,46." These references are not correct for this statement. The manuscript would be strengthened by proper citations in this section, for example Fedotova and colleagues (2017) have a review in Acta Naturae. Additionally, there is evidence that these proteins have roles in both transposon suppression and alternative splicing.

6) The authors state "It has been hypothesized that the Hapalochlaena PSG will exhibit a loss of redundant proteinaceous toxins due to the presence of TTX." This sentence should have a citation or the authors should explain that this statement is their hypothesis.

7) There are minor errors in the sequences presented in Figure 5. In multiple invertebrate species, phenylalanine (F) replaces tyrosine (Y) in the D1 pore. In pufferfish cysteine (C) replaces tyrosine (Y). The figure is constructed in a way that suggests that these amino acids replace a neighboring aspartic acid (D).

Methods

Are the methods appropriate to the aims of the study, are they well described, and are necessary controls included? Choose an item.

Conclusions

Are the conclusions adequately supported by the data shown? Choose an item.

Reporting Standards

Does the manuscript adhere to the journal's guidelines on [minimum standards of reporting?]() Choose an item.

Choose an item.

Statistics

Are you able to assess all statistics in the manuscript, including the appropriateness of statistical tests used? Choose an item.

Quality of Written English

Please indicate the quality of language in the manuscript: Choose an item.

Declaration of Competing Interests

Please complete a declaration of competing interests, considering the following questions:

- Have you in the past five years received reimbursements, fees, funding, or salary from an organisation that may in any way gain or lose financially from the publication of this manuscript, either now or in the future?
- Do you hold any stocks or shares in an organisation that may in any way gain or lose financially from the publication of this manuscript, either now or in the future?
- Do you hold or are you currently applying for any patents relating to the content of the manuscript?
- Have you received reimbursements, fees, funding, or salary from an organization that holds or has applied for patents relating to the content of the manuscript?
- Do you have any other financial competing interests?
- Do you have any non-financial competing interests in relation to this paper?

If you can answer no to all of the above, write 'I declare that I have no competing interests' below. If your reply is yes to any, please give details below.

I declare that I have no competing interests.

I agree to the open peer review policy of the journal. I understand that my name will be included on my report to the authors and, if the manuscript is accepted for publication, my named report including any attachments I upload will be posted on the website along with the authors' responses. I agree for my report to be made available under an Open Access Creative Commons CC-BY license (http://creativecommons.org/licenses/by/4.0/). I understand that any comments which I do not wish to be included in my named report can be included as confidential comments to the editors, which will not be published.

Choose an item.

To further support our reviewers, we have joined with Publons, where you can gain additional credit to further highlight your hard work (see: https://publons.com/journal/530/gigascience). On publication of this paper, your review will be automatically added to Publons, you can then choose whether or not to claim your Publons credit. I understand this statement.

Yes Choose an item.
