# Supplementary material for: Adaptive venom evolution and toxicity in octopods is driven by extensive novel gene formation, expansion, and loss
Source: Gigascience. 2020 Nov 10;9(11):giaa120. doi: 10.1093/gigascience/giaa120 (PMC7656900; doi:10.1093/gigascience/giaa120)
Supplement: giaa120_Reviewer_2_Report_Revision_1 — Shana Geffeney -- 8/25/2020 Reviewed [file giaa120_reviewer_2_report_revision_1.pdf]

Reviewer Report

Title: Adaptive venom evolution and toxicity in octopods is driven by extensive novel gene formation, expansion and loss

Version: Revision 1 **Date:** 8/25/2020

Reviewer name: Shana Geffeney

Reviewer Comments to Author:

The authors have adequately revised their manuscript in response to my review and addressed the comments in my review with their letter. I have just two minor suggestions after reading the revised manuscript. 1) The authors should choose one convention for spelling the word specialized either specialised (British) or specialized (American). 2) In lines 372-374, the authors comment on the interactions between aspartic acid and TTX, "While it has yet to be assessed for TTX resistance, the replacement of Asp in B. candida with a neutral amino acid has been predicted to disrupt TTX binding by preventing formation of a hydrogen bond." A better reference for this statement is Shen et al. (2018). In addition, the cryo-EM structure data from this paper suggest that either a hydrogen bond or a salt bridge could form between TTX and that aspartic acid at this position of the protein.

Methods

Are the methods appropriate to the aims of the study, are they well described, and are necessary controls included? Choose an item.

Conclusions

Are the conclusions adequately supported by the data shown? Choose an item.

Reporting Standards

Does the manuscript adhere to the journal's guidelines on minimum standards of reporting? Choose an item.

Choose an item.

Statistics

Are you able to assess all statistics in the manuscript, including the appropriateness of statistical tests used? Choose an item.

Quality of Written English

Please indicate the quality of language in the manuscript: Choose an item.

Declaration of Competing Interests

Please complete a declaration of competing interests, considering the following questions:

- Have you in the past five years received reimbursements, fees, funding, or salary from an organisation that may in any way gain or lose financially from the publication of this manuscript, either now or in the future?
- Do you hold any stocks or shares in an organisation that may in any way gain or lose financially from the publication of this manuscript, either now or in the future?
- Do you hold or are you currently applying for any patents relating to the content of the manuscript?
- Have you received reimbursements, fees, funding, or salary from an organization that holds or has applied for patents relating to the content of the manuscript?
- Do you have any other financial competing interests?
- Do you have any non-financial competing interests in relation to this paper?

If you can answer no to all of the above, write 'I declare that I have no competing interests' below. If your reply is yes to any, please give details below.

I declare that I have no competing interests.

I agree to the open peer review policy of the journal. I understand that my name will be included on my report to the authors and, if the manuscript is accepted for publication, my named report including any attachments I upload will be posted on the website along with the authors' responses. I agree for my report to be made available under an Open Access Creative Commons CC-BY license (http://creativecommons.org/licenses/by/4.0/). I understand that any comments which I do not wish to be included in my named report can be included as confidential comments to the editors, which will not be published.

Choose an item.

To further support our reviewers, we have joined with Publons, where you can gain additional credit to further highlight your hard work (see: https://publons.com/journal/530/gigascience). On publication of this paper, your review will be automatically added to Publons, you can then choose whether or not to claim your Publons credit. I understand this statement.

Yes Choose an item.
