# Supplementary material for: Adaptive venom evolution and toxicity in octopods is driven by extensive novel gene formation, expansion, and loss
Source: Gigascience. 2020 Nov 10;9(11):giaa120. doi: 10.1093/gigascience/giaa120 (PMC7656900; doi:10.1093/gigascience/giaa120)
Supplement: giaa120_Reviewer_3_Report_Original_Submission — Bryan Fry -- 6/10/2020 Reviewed [file giaa120_reviewer_3_report_original_submission.pdf]

Reviewer Report

Title: Adaptive venom evolution and toxicity in octopods is driven by extensive novel gene formation, expansion and loss

Version: Original Submission **Date:** 6/10/2020

Reviewer name: Bryan Fry

Reviewer Comments to Author:

A truly excellent paper that was a pleasure to read. My comments are very minor:
- TTX resistance in Thamnophis species of snakes should be referenced in the sentence "How resistance to TTX has been acquired at the genetic level, remains a large unknown, with TTX resistance studied in only in a few select species (i.e. pufferfish13, newts14,15 and gastropods16)". This is cited later (ref 58) but it would be appropriate for inclusion in this sentence too.
- For the PSG specific genes, calculations of the relative rates of evolution would be informative as this would be suggestive of adaptive evolution eg are the abundant serine proteases C. minor showing signs of accelerated evolution seen in other venomous lineages such as snakes? Previous work has shown that the sites on the molecular surface are undergoing episodic diversification when compared across a wide range of lineages. In this case, it would be interesting to see what the evolutionary patterns are for C. minor, in that is the extensive duplication accompanied by signs of diversification?
- The M12B metalloprotease type in snake venom has a wide range of demonstrated activities, both anticoagulant (fibrinogenolytic) but also procoagulant (Factor X activating [Atractaspis and Daboia venoms] and prothrombin activating (Bothrops, Echis, and Dispholidus/Thelatornis venoms]) prothrombin activating metalloproteases from Dispholidus typus (boomslang) and Thelotornis mossambicanus (twig snake)." Comp Biochem Physiol C Toxicol Pharmacol: 108625. Oulion, B., J. S. Dobson, C. N. Zdenek, K. Arbuckle, C. Lister, F. C. P. Coimbra, B. Op den Brouw, J. Debono, A. Rogalski, A. Violette, R. Fourmy, N. Frank and B. G. Fry (2018). "Factor X activating Atractaspis snake venoms and the relative coagulotoxicity neutralising efficacy of African antivenoms." Toxicol Lett 288: 119-128. Rogalski, A., C. Soerensen, B. Op den Brouw, C. Lister, D. Dashevsky, K. Arbuckle, A. Gloria, C. N. Zdenek, N. R. Casewell, J. M. Gutierrez, W. Wuster, S. A. Ali, P. Masci, P. Rowley, N. Frank and B. G. Fry (2017). "Differential procoagulant effects of saw-scaled viper (Serpentes: Viperidae: Echis) snake venoms on human plasma and the narrow taxonomic ranges of antivenom efficacies." Toxicol Lett 280: 159-170. Sousa, L. F., C. N. Zdenek, J. S. Dobson, B. Op den Brouw, F. Coimbra, A. Gillett, T. H. M. Del-Rei, H. M. Chalkidis, S. Sant'Anna, M. M. Teixeira-da-Rocha, K. Grego, S. R. Travaglia Cardoso, A. M. Moura da Silva and B. G. Fry (2018). "Coagulotoxicity of Bothrops (Lancehead Pit-Vipers) Venoms from Brazil: Differential Biochemistry and Antivenom Efficacy Resulting from Prey-Driven Venom Variation." Toxins (Basel) 10(10): 411.

Methods

Are the methods appropriate to the aims of the study, are they well described, and are necessary controls included? Choose an item.

Conclusions

Are the conclusions adequately supported by the data shown? Choose an item.

Reporting Standards

Does the manuscript adhere to the journal's guidelines on minimum standards of reporting? Choose an item.

Choose an item.

Statistics

Are you able to assess all statistics in the manuscript, including the appropriateness of statistical tests used? Choose an item.

Quality of Written English

Please indicate the quality of language in the manuscript: Choose an item.

Declaration of Competing Interests

Please complete a declaration of competing interests, considering the following questions:

- Have you in the past five years received reimbursements, fees, funding, or salary from an organisation that may in any way gain or lose financially from the publication of this manuscript, either now or in the future?
- Do you hold any stocks or shares in an organisation that may in any way gain or lose financially from the publication of this manuscript, either now or in the future?
- Do you hold or are you currently applying for any patents relating to the content of the manuscript?
- Have you received reimbursements, fees, funding, or salary from an organization that holds or has applied for patents relating to the content of the manuscript?
- Do you have any other financial competing interests?
- Do you have any non-financial competing interests in relation to this paper?

If you can answer no to all of the above, write 'I declare that I have no competing interests' below. If your reply is yes to any, please give details below.

I declare that I have no competing interests

I agree to the open peer review policy of the journal. I understand that my name will be included on my report to the authors and, if the manuscript is accepted for publication, my named report including any attachments I upload will be posted on the website along with the authors' responses. I agree for my

report to be made available under an Open Access Creative Commons CC-BY license (http://creativecommons.org/licenses/by/4.0/). I understand that any comments which I do not wish to be included in my named report can be included as confidential comments to the editors, which will not be published.

Choose an item.

To further support our reviewers, we have joined with Publons, where you can gain additional credit to further highlight your hard work (see: https://publons.com/journal/530/gigascience). On publication of this paper, your review will be automatically added to Publons, you can then choose whether or not to claim your Publons credit. I understand this statement.

Yes Choose an item.
